# Hydroquinone Oxidation by Redox‐active Guanidines and Thioguanidines: Faster Conversion to High‐Potential Than to Low‐Potential Quinones

**DOI:** 10.1002/chem.70981

**Published:** 2026-04-10

**Authors:** Ute Wild, Olaf Hübner, Sebastian Jaworski, Elisabeth Kaifer, Hans‐Jörg Himmel

**Affiliations:** ^1^ Anorganisch‐Chemisches Institut Ruprecht‐Karls‐Universität Heidelberg Heidelberg Germany

**Keywords:** dehydrogenative coupling, guanidines, hydroquinone oxidation, proton‐coupled electron transfer, redox‐active molecules

## Abstract

Quinones are important oxidants in biology and synthetic chemistry. Herein, we study the kinetics of the oxidation of hydroquinones to quinones with redox‐active guanidines and thioguanidines. In the first section, we report the synthesis and characterization of the first redox‐active aromatic compounds with two, three, and four thioguanidino groups and compare their redox properties with the corresponding oligoguanidines. The stable salts obtained upon chemical two‐electron oxidation of the tetra‐thioguanidine were then applied in dehydrogenative P–P coupling reactions at room temperature, demonstrating their superior proton‐coupled electron‐transfer reactivity compared with previously used oligoguanidines. Oxidation of hydroquinones and halogenated derivatives to the 1,4‐benzoquinones is also much faster with the new tetrathioguanidine than with oligoguanidines. Seemingly paradoxically, for tetraguanidines and tetrathioguanidines, oxidations to high‐potential halogenated quinones are faster than those to low‐potential quinones (due to proton‐coupled electron transfer (PCET)), motivating the use of redox‐active guanidines as redox mediators.

## Graphical abstract

New redox‐active oligothioguanidines are developed and used in dehydrogenative P–P coupling reactions and hydroquinone oxidations. Tetrathioguanidino‐ and tetraguanidinobenzenes oxidize high‐potential halogenated hydroquinones faster than relatively low‐potential hydroquinone. The interplay between electron and proton transfer that is responsible for this seemingly paradoxical trend is analysed.

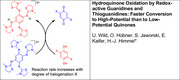

## 1 Introduction

Proton‐coupled electron transfer (PCET) is an extremely important reaction in modern synthetic chemistry and in biology [1, 2]. Nature uses low‐potential quinones such as 1,4‐benzoquinone derivatives in a variety of PCET reactions. In synthetic organic chemistry, high‐potential 1,4‐benzoquinone derivatives such as tetrachloro‐1,4‐benzoquinone (chloranil, CA) or 2,3‐dichloro‐5,6‐dicyano‐1,4‐benzoquinone (DDQ) are widely used [3].

Several studies demonstrated that low‐potential 1,4‐benzoquinones could readily be regenerated from the reduced 1,4‐hydroquinone redox states in an environmentally friendly way by catalytic oxidation with dioxygen. For example, catalytic hydroquinone oxidation with O_2_ was achieved with a Co(salophen) catalyst, which does not lead to the accumulation of the H_2_O_2_ intermediate [4]. Kochi and coworkers reported the oxidation of 1,4‐hydroquinone to benzoquinone in an O_2_ atmosphere with NO_2_ as a catalyst in CH_2_Cl_2_ solution at −10°C, or more conveniently with NaNO_2_ / HCl as a catalyst at room temperature [5, 6]. The regeneration of high‐potential 1,4‐benzoquinone derivatives from their reduced hydroquinone states by oxidation with dioxygen as terminal oxidant is much less developed, but highly desirable, since it allows selective substrate oxidations with catalytic amounts of 2,3‐dichloro‐5,6‐dicyano‐p‐benzoquinone (DDQ) or tetrachloro‐p‐benzoquinone (chloranil) and O_2_ as terminal oxidant. Apart from electrochemical methods [7, 8, 9], NaNO_2 _/ acid or tert‐butyl nitrite (*t*BuONO, TBN) in combination with DDQ as a catalyst was used in oxidation reactions with O_2_ as a terminal oxidant [10, 11, 12, 13]. Also, DDQ together with HNO_3_ [14] or with Fe(NO_3_)_3_ [15] as a catalyst pair was applied. In these reactions, NO is oxidized by dioxygen to NO_2_. Substrate oxidation by the quinone (DDQ) produces the hydroquinone, which is re‐oxidized to the quinone by NO_2_. The NO_2_ is converted to NO, which is again re‐oxidized by dioxygen to NO_2_. DDQ can be photochemically excited to the triplet state to increase its reduction potential [16, 17, 18]. In the case of chloranil (CA), polymer‐incarcerated platinum was reported as a catalyst for the re‐oxidation of the corresponding hydroquinone to CA with O_2_ [19]. This method also allows the development of in situ coupled oxidation cycles for catalytic oxidations with *o*‐CA as catalyst and dioxygen as terminal oxidant [20]. Despite these encouraging results, there is still a huge demand for alternative redox mediators for the oxidation of high‐potential hydroquinones. The use of TBN can cause side reactions (e.g., nitration of organic groups in the substrate), and polymer‐incarcerated platinum is not a standard, readily available material and is hardly suitable for larger‐scale processes.

It is well known that the optimization of PCET oxidizing reagents within a structurally‐related family of molecules is hampered by the effect that an increase in the redox potential is accompanied by a decrease in the Brønsted basicity of the reduced form of the PCET reagent [1, 21]. The degree of this thermodynamic compensation at standard conditions can be quantified by a proportionality constant γ between the change of redox potential (Δ*E* ^0^) and Δ*pK*_a_ value of the protonated, reduced species, Δ*E* ^0^ = −γ(0.059 mV) Δ*pK*_a_. Within this context, Hammes–Schiffer and Stahl analyzed the dependence of the one‐electron reduction potential of quinones on the potential for two‐electron‐two‐proton (2e^−^/2H^+^) transfer [22]. In their study, they observed that halogenated 1,4‐benzoquinones, despite differing significantly in their one‐electron reduction potential, exhibit similar potentials for 2e^−^/2H^+^ reduction. This finding supports the idea of a redox mediator‐triggered re‐oxidation of CA and other high‐potential quinones.

**FIGURE 1:**
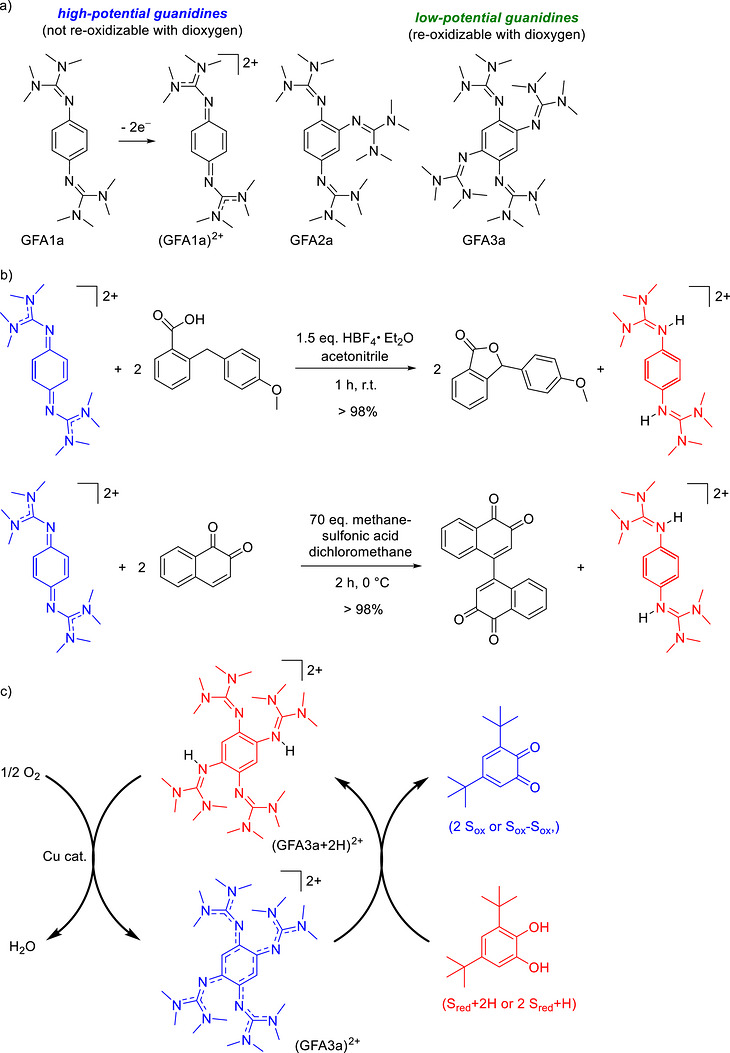
(a) Examples for high‐potential and low‐potential GFA compounds. (b) Examples for dehydrogenative C─O and C─C coupling reactions with GFA1a. (c) Application of GFA3a as redox catalyst for the selective oxidation of organic substrates with dioxygen as terminal oxidant (Cu cat: CuCl_2_+Cu(H_2_O)_6_(BF_4_)_2_). In general, a reduced substrate *S*_red_+2H or two *S*_red_+H is oxidized to two *S*_ox_ or *S*_ox_–*S*_ox_. An example is 3,5‐ditertbutyl‐*o*‐hydroquinone oxidation, which does not work without the addition of (GFA3a)^2+^ as redox mediator.

Starting with the first synthesis of 1,2,4,5‐tetrakis(tetramethylguanidino)benzene [23], our group systematically studied guanidino‐functionalized aromatic compounds (GFAs) and developed several applications for this class of compounds (e.g., as reagents in PCET, as redox‐active ligands for the design of bistable coordination compounds and redox catalysts, and as dopants in material science) [24, 25, 26]. Within this research theme, we studied the PCET reactivity of the redox‐active guanidines GFA1a—GFA3a (Figure 1a), especially the “high‐potential” guanidine 1,4‐bis(tetramethylguanidino)benzene (GFA1a) [27, 28] and the “low‐potential” guanidine 1,2,4,5‐tetrakis(tetramethylguanidino)benzene (GFA3a, Figure 1a) [29] after two‐electron oxidation to the dications. Two examples for the application of GFA1a for C─O and C─C coupling reactions are shown in Figure 1b [27, 28]. Since a variety of interesting chemical oxidation reactions, such as aryl–aryl coupling reactions, are initiated by an electron transfer step, high‐potential guanidines exhibit a larger substrate scope than the low‐potential ones, in similarity to the results obtained for quinones.

Interestingly, the reduced, twofold protonated compound (GFA3a+2H)^2+^ can be efficiently re‐oxidized with dioxygen at room temperature in CH_3_CN solution in the presence of CuCl_2_ + Cu(H_2_O)_6_(BF_4_)_2_ as a catalyst (Figure 1c) [30]. In this reaction, O_2_ is converted to H_2_O; no intermediate formation of H_2_O_2_ is observed. Therefore, salts of (GFA3a)^2+^ can be applied as catalysts for the selective 2e^−^, 2H^+^ oxidation of organic substrates (e.g., 3,5‐ditertbutyl‐*o*‐hydrobenzoquinone) [30]. However, the scope is limited to substrates with relatively low redox potential. On the other hand, the known high‐potential guanidine GFA1a (or its protonated version (GFA1a+2H)^2+^), while being a more capable PCET reagent, cannot be re‐oxidized with dioxygen in such a simple way. Hence, the situation is comparable to that found for hydroquinones/quinones.

**FIGURE 2:**
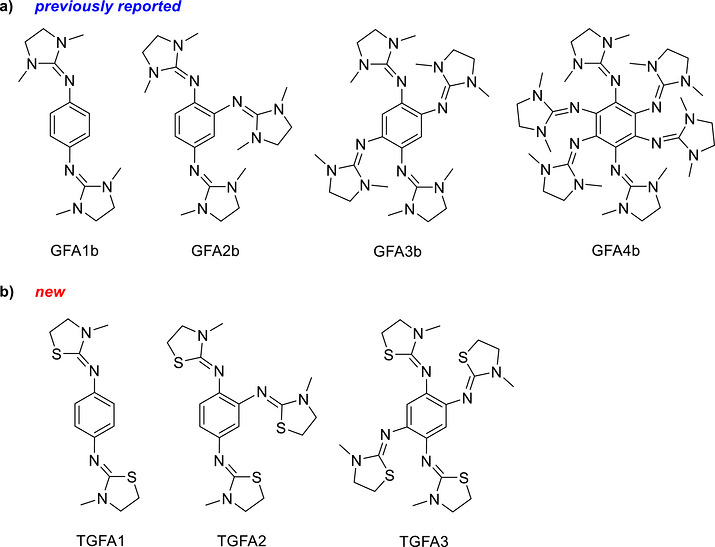
Structures of the previously studied compounds GFA1b‐GFA4b and the new compounds TGFA1‐TGFA3.

The redox potential and other properties of GFAs can be tuned by modifications of the size and substituents at the aromatic core, and the number and nature of the guanidino groups. A rather simple modification is the replacement of the tetramethylguanidino group by the cyclic *N*,*N*’‐dimethyl(ethylene)guanidino group, leading to compounds GFA1b–GFA3b, and also the first hexaguanidinobenzene, GFA4b [29, 31, 32, 33]. GFA4b is the strongest known organic four‐electron donor for which the reduced and oxidized redox states (dication or tetracation) are stable and fully characterized [33]. In this work, we report the first redox‐active thioguanidine molecules, in which two (TGFA1), three (TGFA2), or four (TGFA3) thioguanidino groups are attached to a benzene ring (Figure 2b). The substitution of a guanidino NMe group by sulfur destabilizes the oxidized redox state and increases the redox potential. Then, the performance of these compounds in PCET reactions, especially the oxidation of hydroquinones, is tested.

## 2 Results and Discussion

**FIGURE 3:**
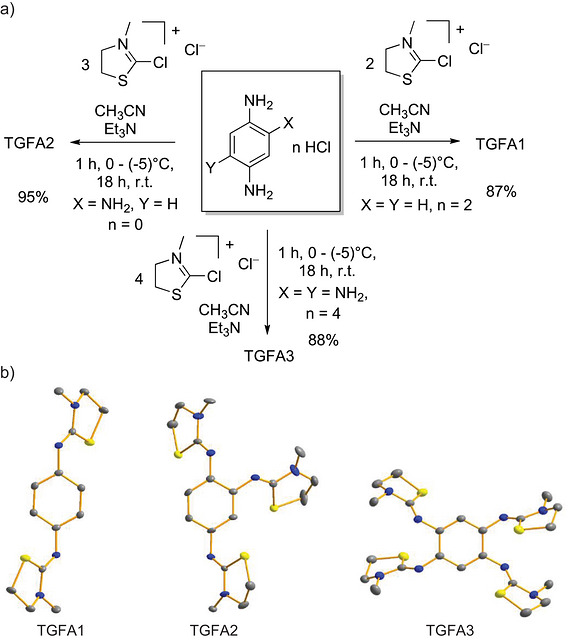
(a) Synthesis of the new redox‐active thioguanidines TGFA1–TGFA3. (b) Illustration of the solid‐state structures. Hydrogen atoms are omitted. Displacement ellipsoids drawn at the 50% probability level. Color code: C gray, N blue, S yellow.

The synthesis of the three new redox‐active thioguanidines was accomplished by reaction of the corresponding amino‐substituted benzenes with the required equivalents of 2‐chloro‐4,5‐dihydro‐3‐methylthiazolium chloride (Figure 3a). The compounds were obtained in isolated yields of 87% (TGFA1), 95% (TGFA2), and 88% (TGFA3). Crystals of all three molecules suitable for SC‐XRD analysis were grown from CH_2_Cl_2_ solutions layered with *n*‐hexane (Figure 3b). The thioguanidino groups are tilted with respect to the central aromatic C_6_ ring plane; the corresponding dihedral angles measure ca. 50°–70°.

In further experiments, we protonated the new thioguanidines with two equivalents of NH_4_PF_6_ in CH_3_CN solution (1 h at 50 °C). In this way, the di‐protonated compounds (TGFA2+2H)(PF_6_)_2_ and (TGFA3+2H)(PF_6_)_2_ were synthesized in yields of 72% and 67%, respectively, and fully characterized. In the case of TGFA1, protonation with NH_4_PF_6_ leads to a mixture of mono‐ and di‐protonated compounds. Crystal structures were obtained for all three di‐protonated compounds (see Supporting Information for details).

**FIGURE 4:**
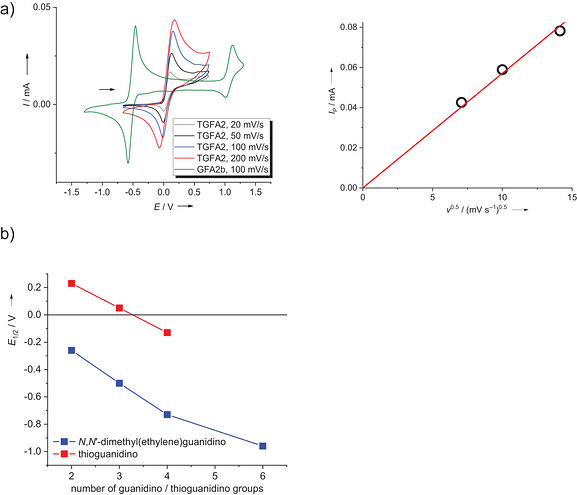
(a) Left: CV curves for TGFA2 in CH_2_Cl_2_ solutions, recorded with different scan rates. For comparison, the CV curve for GFA2b is also included. Right: Plot of the peak current (I_P_) versus the square root of the scan rate (v^1/2^) (Randles–Ševčik plot). (b) Trend of the redox potentials (*E*_1/2_ values, versus the reference redox couple ferrocenium/ferrocene, GFA4b in CH_3_CN, all others in CH_2_Cl_2_) with the number of guanidino groups in the series GFA1b‐GFA4b (blue curve) and in the series TGFA1‐TGFA3 (red curve).

Cyclic voltammetry (CV) measurements for TGFA1‐TGFA3 were carried out in CH_2_Cl_2_ and CH_3_CN solutions (see Supporting Information for details). All compounds show a (quasi‐)reversible two‐electron redox event. For example, Figure 4a shows the CV curves of TGFA2 in CH_2_Cl_2_, recorded with different scan rates, together with the CV curve of GFA2b. The *E*_1/2_ value is significantly increased with respect to the value for GFA2b, from −0.50 V for GFA2b to 0.07 V for TGFA2 (Figure 4a). A Randles–Ševčik plot indicates the validity of the Butler–Volmer equation [34, 35].

In Figure 4b, the *E*_1/2_ values are plotted as a function of the number of thioguanidino / guanidino groups. It can be seen that for oligothioguanidines and oligoguanidines, the *E*_1/2_ values steadily decrease with the number of thioguanidino / guanidino groups. In all cases, the compounds with thioguanidino groups exhibit significantly higher *E*_1/2_ values than the corresponding molecules with an equal number of guanidino groups. This could be rationalized by the less effective charge delocalization in the dicationic redox state of the thioguanidines.

### 2.1 Chemical Oxidation

**FIGURE 5:**
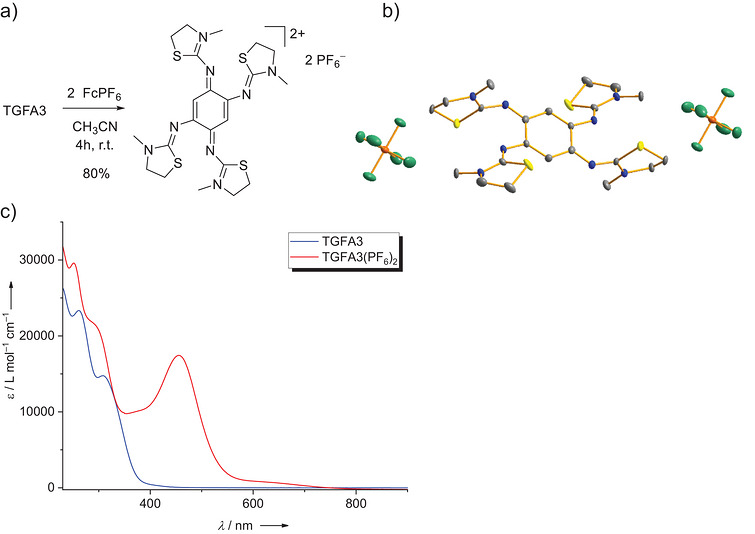
(a) Synthesis of the salt TGFA3(PF_6_)_2_. (b) Illustration of the solid‐state structure of TGFA3(PF_6_)_2_. Only one of the two slightly different dications in the unit cell is shown. Hydrogen atoms are omitted. Displacement ellipsoids drawn at the 50% probability level. Colour code: C gray, N blue, S yellow, P orange, F green. (c) UV–vis spectra of TGFA3 (CH_2_Cl_2_) and TGFA3(PF_6_)_2_ (CH_3_CN).

Oxidation of TGFA3 with two equivalents of FcPF_6_ in CH_3_CN at room temperature led to the brown salt TGFA3(PF_6_)_2_ in an isolated yield of 80% (Figure 5a). Crystals were grown at 3°C from a concentrated CH_3_CN solution layered with Et_2_O (Figure 5b). Selected bond parameters of the neutral and dicationic redox state of the tetrathioguanidine are compared in Table 2. The unit cell contains two dications with different structural parameters (Mol1 and Mol2 in Table 2). As expected, the bond lengths of the former imino bonds in the guanidino groups (termed a in Table 1) increase upon oxidation, and the bond lengths of the C─N bond connecting the thioguanidino groups with the central C_6_ ring (termed b in Table 1) decrease. However, the two pairs of guanidino groups in the oxidized species differ significantly in their structural parameters, arguing for the presence of a quinone‐type structure. On the other hand, the three clearly different C─C bond lengths in the C_6_ ring are not really in line with a simple quinone‐type structure. In contrast to the 1,2,4,5‐tetraguanidino‐benzene compounds GFA3a and GFA3b, the charge seems not to be delocalized equally over all four thioguanidino groups.

**TABLE 1:** Potentials (*E*_1/2_ and *E*_ox_ values in V) for two‐electron redox process from CV measurements on TGA1–TGFA3 and GFA1b‐GFA3b in CH_2_Cl_2_ solutions.

	1		2		3	
	*E*_1/2_	*E*_ox_	*E*_1/2_	*E*_ox_	*E*_1/2_	*E*_ox_
TGFA_	0.23	0.32	0.07	0.15	−0.11	−0.03
GFA_b	−0.26	−0.22	−0.50	−0.41	−0.73	−0.62

In the UV–vis spectrum, recorded for a CH_3_CN solution, the dication exhibits a strong band at 456 nm. For comparison, a band at 425 nm is characteristic of (GFA3a)^2+^. Since there is no band in this region in the spectrum of the neutral compound, UV–vis spectroscopy can conveniently be used to record the progress of redox and PCET reactions with the dication.

**SCHEME 1:**
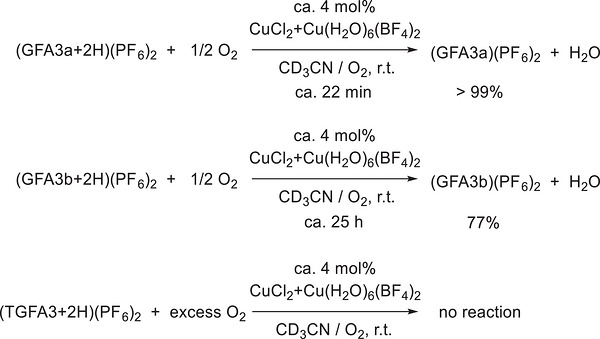
Experiments to oxidize catalytically the protonated tetraguanidines / tetrathioguanidine to the dicationic redox states with dioxygen.

We also tested catalytic oxidation of the di‐protonated compound, (TGFA3 + 2H)(PF_6_)_2_, with dioxygen and CuCl_2_+Cu(H_2_O)_6_(BF_4_)_2_ (ca. 4 mol%) as catalyst (see Scheme 1 and Supporting Information for details). However, no conversion was observed with NMR spectroscopy. By contrast, under similar conditions (GFA3a + 2H)(PF_6_)_2_ was oxidized quantitatively to GFA3a(PF_6_)_2_ within 22 min at room temperature [30]. Oxidation of (GFA3b + 2H)(PF_6_)_2_ with dioxygen was also possible, but proceeded much slower; 77% conversion was reached after 25 h at room temperature (see Supporting Information). In these reactions, O_2_ is converted to H_2_O; the formation of H_2_O_2_ was not observed. This result can be rationalized by the simultaneous donation of two electrons from the tetraguanidine / tetrathioguanidine, facilitating two‐electron oxygen reduction.

**FIGURE 6:**
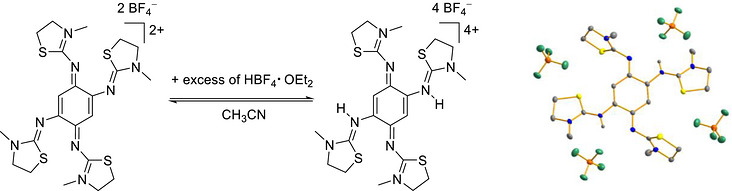
Formation of the oxidized and twofold protonated salt (TGFA3 + 2H)(BF_4_)_4_, and illustration of its solid state structure. Hydrogen atoms are omitted. Displacement ellipsoids drawn at the 50% probability level. Colour code: C gray, N blue, S yellow, P orange, F green.

Interestingly, the oxidized, dicationic compound can be protonated with strong acids. Hence, from a solution containing TGFA3(BF_4_)_2_ together with an excess of HBF_4_·Et_2_O, the salt of the diprotonated, oxidized tetracation, (TGFA3 + 2H)(BF_4_)_4_, was isolated and crystallized (Figure 6). The effect of protonation is mainly a harmonization of the N─C bond lengths *a* and an increase of the differences of *b* (Table 2). Titration experiments show that quantitative di‐protonation requires the use of ca. 60 equivalents of HBF_4_·Et_2_O in the UV–vis experiments (see Supporting Information for details). Due to the higher Brønsted basicity, protonation of GFA3a(BF_4_)_2_ to give (GFA3a + 2H)(BF_4_)_4_ requires only two equivalents of HBF_4_·Et_2_O [36]. In the ^1^H NMR spectra, the two N─H protons show at *δ* = 8.82 ppm. Mono‐ and di‐protonation significantly changes the optical properties. In the UV–vis spectra (CH_3_CN solution), (TGFA3 + H)^3+^ shows bands at 382 and 624 nm (reaching maximum intensity with ca. seven equivalents of HBF_4_·Et_2_O), and (TGFA3+2H)^4+^ shows a band at 362 nm (reaching maximum intensity with ca. 60 equivalents of HBF_4_·Et_2_O).

**TABLE 2:** Selected bond lengths (in Å) for TGFA3, (TGFA3 + 2H)^2+^ (in the salt (TGFA3 + 2H)(PF_6_)_2_), (TGFA3)^2+^ (in the salt TGFA3(PF_6_)_2_) and (TGFA3 + 2H)^4+^ (in the salt TGFA3 + 2H(BF_4_)_4_) from SC‐XRD data.

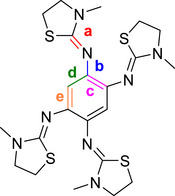				
Bond	TGFA3	(TGFA3 + 2H)^2+^	(TGFA3)^2+^		(TGFA3 + 2H)^4+^
			Mol.1	Mol.2	
*a* (C─N)	1.280(4)/ 1.273(4)	1.289(6)/	1.365(4)/	1.341(4)/	1.370(3)/
		1.288(6)/	1.299(5)	1.330(5)	1.351(2)^a^
		1.326(6)^a^/			
		1.326(6)^a^			
*b* (C─N)	1.413(3)/ 1.416(3)	1.395(5)/	1.292(4)/	1.298(4)/	1.293(2)/
		1.402(5)/	1.381(4)	1.355(4)/	1.395(2)^a^
		1.425(6)^a^/			
		1.426(6)^a^			
*c* (C─C)	1.416(4)	1.402(6)/ 1.406(6)	1.484(5)	1.507(5)	1.472(3)
*d* (C─C)	1.397(4)	1.391(6)/ 1.384(6)	1.448(5)	1.431(5)	1.341(3)
*e* (C─C)	1.389(4)	1.398(6)/ 1.398(6)	1.359(4)	1.356(4)	1.444(3)

^a^ Involving the protonated *N* atoms.

**FIGURE 7:**
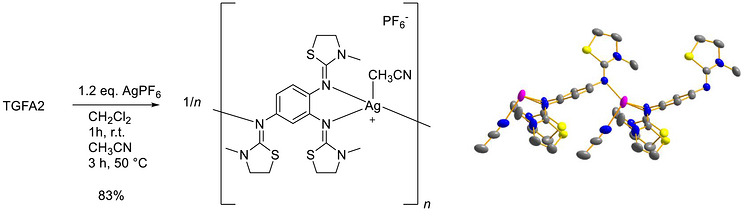
Polycationic chain coordination polymer isolated upon reaction of TGFA2 with AgPF_6_. Hydrogen atoms are omitted. Displacement ellipsoids drawn at the 50% probability level. Colour code: C gray, N blue, S yellow, Ag pink.

Experiments to oxidize TGFA1 or TGFA2 were only partially successful; an isolation of the oxidized redox states in pure form was not possible. Since oxidation with FcPF_6_ is hampered by the relatively high *E*_1/2_ values (*E*_1/2_ = 0.23 V for TGFA1 and 0.07 V for TGFA2), we used stronger oxidizing reagents in these experiments. However, reaction with AgPF_6_ resulted in the formation of coordination polymers with neutral thioguanidines bridged by Ag^+^ ions, which resist heating to a temperature of 50°C. An example is the silver chain polymer crystallizing from a mixture of TGFA2 and AgPF_6_ (Figure 7). The Ag^+^ ions are coordinated in a tetrahedral fashion to the adjacent imino N atoms of a first neutral TGFA2 unit, and to one of the imino N atoms of the next neutral TGFA2 unit, and in addition to an acetonitrile molecule.

### 2.2 Application in P–P Coupling Reactions

**FIGURE 8:**
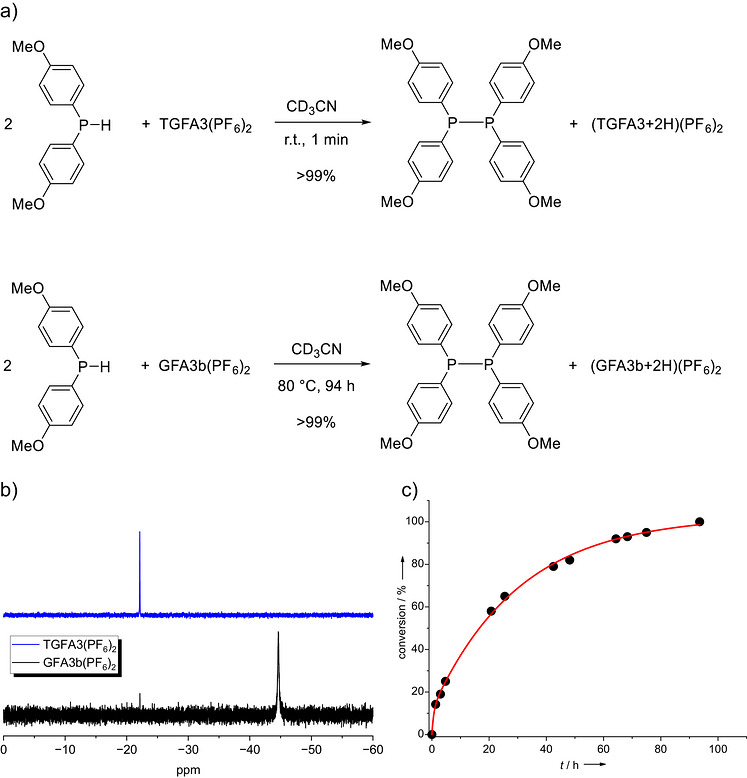
(a) Dehydrogenative P–P coupling reactions of phosphanes with TGFA3(PF_6_)_2_ and GFA3b(PF_6_)_2_. (b) Comparison between the ^31^P NMR spectra (242.95 MHz, CD_3_CN) recorded after mixing together the two reactants at room temperature. (c) Conversion versus time plot from ^31^P NMR experiments for the reaction of GFA3b(PF_6_)_2_ with di(4‐methoxyphenyl)phosphane at 80°C.

The new compound TGFA3(PF_6_)_2_ and the previously reported GFA3b(PF_6_)_2_ were applied in a coupling reaction of two phosphanes, namely di(4‐methoxyphenyl)phosphane, to give the corresponding diphosphane (Figure 8a). In the case of TGFA3(PF_6_)_2_, the reaction was immediately completed at room temperature in quantitative NMR yield (see Figure 8b and Supporting Information for details). On the other hand, almost no conversion was observed for GFA3b(PF_6_)_2_ at room temperature. The reaction was very slow, even at 80°C, requiring ca. 94 h to reach a quantitative yield (see plot in Figure 8c). This result is in line with a previous study on dehydrogenative P–P coupling reactions at 80°C with GFA3a(PF_6_)_2_ [37]. Hence, TGFA3(PF_6_)_2_ is a much more potent reagent for dehydrogenative P–P coupling reactions than salts of (GFA3a)^2+^ or (GFA3b)^2+^.

### 2.3 Hydroquinone Oxidation

Next, we tested oxidation of hydroquinone and the halogenated derivatives 2‐chlorobenzene‐1,4‐diol, 2‐bromobenzene‐1,4‐diol, and 2,5‐dibromobenzene‐1,4‐diol to give the corresponding benzoquinones. Since the reactions were generally too slow with GFA3b(PF_6_)_2_, the experiments focussed on TGFA3(PF_6_)_2_ and GFA3a(PF_6_)_2_.

**SCHEME 2:**
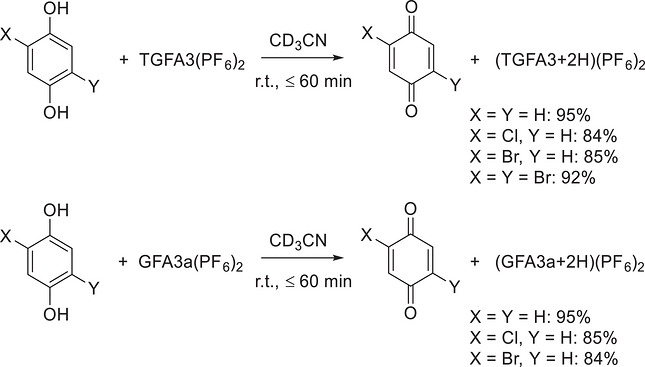
Oxidation of (halogenated) hydroquinones with salts of (TGFA3)^2+^ and (GFA3a)^2+^ in acetonitrile solutions, together with the NMR yields.

First, we studied the reactions in NMR experiments (see Supporting Information for details). These experiments (carried out in CD_3_CN solutions) showed that oxidation of hydroquinone to benzoquinone proceeds according to Scheme 2 without formation of by‐products. (We generally used a small excess of TGFA3(PF_6_)_2_ or GFA3a(PF_6_)_2_, see Supporting Information.) With TGFA3(PF_6_)_2_, 1,4‐benzoquinone was obtained in 95% NMR yield (after 25 min) at room temperature. In the case of GFA3a(PF_6_)_2_, a yield of 95% was obtained, too (reaction time of 45 min at room temperature). Please note that for both reactions, quantitative conversion can be achieved upon later addition of some more TGFA(PF_6_)_2 _/ GFA3a(PF_6_)_2_ (see Supporting Information). Also, the halogenated hydroquinones were efficiently converted to the quinones (Scheme 2); only for the combination GFA3a(PF_6_)_2_ and 2,5‐dibromobenzene‐1,4‐diol, the formation of some side products was observed (therefore, this reaction was not further analyzed).

### 2.4 Quantum‐Chemical Calculations

In the dehydrogenative P–P coupling and hydroquinone oxidation reactions presented in the previous two sections, electron transfer is coupled with proton transfer. In this section, the interplay between electron and proton transfer is inspected. Quantum‐chemical calculations were carried out with the B3LYP functional (with D3 dispersion correction and Becke–Johnson damping) in combination with the def2‐TZVP basis set. The most important results are included in Table 3 (see Supporting Information for details). First, we compared the electron transfer between thioguanidines and guanidines by calculating the Gibbs free energy changes of some comparative reactions. In their dicationic, oxidized redox state, the compounds with *N*,*N*’‐dimethyl(ethylene)guanidino groups reduce the corresponding compounds with thioguanidino groups, showing that the thioguanidines are stronger oxidants. The Gibbs free energy change decreases (to more negative values) with increasing number of guanidino/thioguanidino groups. The calculated Δ*G* values fit the experimentally derived ones (Δ*G*_exp_ values included in Table 3).

**TABLE 3:** Calculated Gibbs free energy changes (Δ*G*_calcd_ (298 K, 1 bar, in kJ mol^−1^) from quantum‐chemical calculations for electron transfer, proton transfer, and PCET between the three compounds, as well as for oxidation of hydroquinone to benzoquinone. In the case of electron transfer, Δ*G*_exp_ (in kJ mol^−1^) from CV measurements in CH_2_Cl_2_ solution are included, estimated with Δ*G*_exp_ = −2·F·Δ*E*_1/2_, where F = 96485.3321 s A mol^−1^ is the Faraday constant.

Electron transfer	Δ*G*_calcd_
(TGFA1)^2+^ + GFA1b → TGFA1 + (GFA1b)^2+^	−103 (−90.7 (Δ*G*_exp_))
(TGFA2)^2+^ + GFA2b → TGFA2 + (GFA2b)^2+^	−115 (−106.1 (Δ*G*_exp_))
(TGFA3)^2+^ + GFA3a → TGFA3 + (GFA3a)^2+^	−89.1
(TGFA3)^2+^ + GFA3b → TGFA3 + (GFA3b)^2+^	−122.5 (−121.6 (Δ*G*_exp_))
(GFA3a)^2+^ + GFA3b → GFA3a + (GFA3b)^2+^	−33.4
Proton transfer	Δ*G*_calcd_
(GFA3a+H)^+^ + GFA3b → GFA3a + (GFA3b+H)^+^	6.2
(GFA3a+H)^+^ + TGFA3 → GFA3a + (TGFA3+H)^+^	40.7
(GFA3b+H)^+^ + TGFA3 → GFA3b + (TGFA3+H)^+^	34.5
PCET	Δ*G*_calcd_
(TGFA3)^2+^ + (GFA3a+2H)^2+^ → (TGFA3+2H)^2+^ + (GFA3a)^2+^	−9.5
(TGFA3)^2+^ + (GFA3b+2H)^2+^ → (TGFA3+2H)^2+^ + (GFA3b)^2+^	−53.0
(GFA3a)^2+^ + (GFA3b+2H)^2+^ → (GFA3a+2H)^2+^ + (GFA3b)^2+^	−43.5
hydroquinone oxidation	Δ*G*_calcd_
(TGFA3)^2+^ + p‐C_6_H_4_(OH)_2_ → (TGFA3+2H)^2+^ + p‐C_6_H_4_O_2_	−8.2
(GFA3a)^2+^ + p‐C_6_H_4_(OH)_2_ → (GFA3a+2H)^2+^ + p‐C_6_H_4_O_2_	+1.3
(GFA3b)^2+^ + p‐C_6_H_4_(OH)_2_ → (GFA3b+2H)^2+^ + p‐C_6_H_4_O_2_	44.8

Next, we compared the absolute proton affinities. These calculations focused on the compounds with four guanidino/thioguanidino groups. It can be seen that the TGFA compounds are weaker Brønsted bases compared with the corresponding GFA compounds GFA3a and GFA3b (Table 3). According to the calculations, GFA3a is the strongest Brønsted base, but GFA3b is only slightly weaker.

The data clearly show that there is a compensating effect between compounds with thioguanidino and with guanidino groups. The change from guanidino groups to thioguanidino groups shifts the potentials to higher values, but at the same time decreases the Brønsted basicity. To see if this compensation leads to equal PCET properties, we next inspected PCET (2e^−^, 2H^+^ transfer) reactions between the compounds (Table 3). Here, it can be seen that (TGFA3)^2+^ is the strongest PCET reagent, as it oxidizes both (GFA3a + 2H)^2+^ and (GFA3b + 2H)^2+^. However, there is a huge difference between the two tetraguanidines. While reaction with (GFA3a + 2H)^2+^ is only slightly exergonic (Δ*G* = −9.5 kJ mol^−1^), that with (GFA3b + 2H)^2+^ is highly exergonic (Δ*G* = −71.1 kJ mol^−1^). Interestingly, a compensating effect is not observed between GFA3a and GFA3b (the compounds with guanidino groups), since GFA3a is a stronger oxidant and a slightly stronger Brønsted base.

Finally, oxidation of hydroquinone (*p*‐C_6_H_4_(OH)_2_) to p‐benzoquinone (*p*‐C_6_H_4_O_2_) is compared (see Table 3). There is a clear trend; the Δ*G* value rises in the order (TGFA3)^2+^ < (GFA3a)^2+^ < (GFA3b)^2+^. Reaction with (TGFA3)^2+^ is mildly exergonic (Δ*G* = −8.2 kJ mol^−1^), and that with (GFA3a)^2+^ is almost thermoneutral. By contrast, the reaction with (GFA3b)^2+^ is endergonic. It should be noted that the calculations were deliberately carried out without the inclusion of the solvent and counterion effects to compare the inherent properties. Therefore, the absolute numbers certainly change for reactions in solutions.

### 2.5 Kinetic Analysis (UV–vis)

**FIGURE 9:**
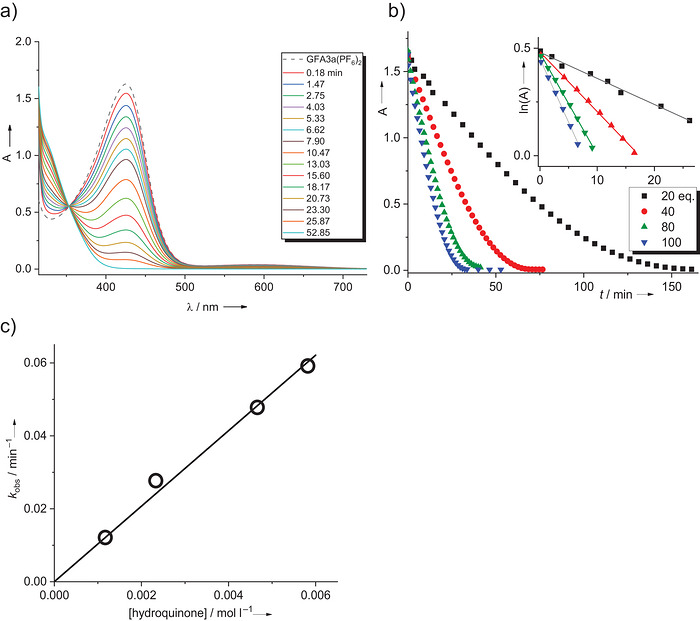
(a) UV–vis spectra recorded for the reaction of (GFA3a)^2+^ with 100 equivalents of hydroquinone in CH_3_CN solution to give (GFA3a + 2H)^2+^ and benzoquinone. (b) Absorption at 425 nm versus time plots to obtain the *k*_obs_ values. c) Plot *k*_obs_ versus the hydroquinone concentration to determine the second‐order rate constant *k* (slope).

The kinetic analysis (see Supporting Information for details) followed the previously reported procedure for PCET reactions with quinones [38] and guanidines [29]. A large excess of the hydroquinone was used to ensure pseudo‐first‐order conditions. UV–vis spectra were recorded as a function of time for CH_3_CN solutions that contain the oxidized tetrathioguanidine, (TGFA3(PF_6_)_2_), or oxidized tetraguanidine, GFA3a(PF_6_)_2_, together with an excess (10–100 equivalents) of the hydroquinone. As an example, Figure 9a shows the UV–vis spectra recorded for GFA3a(PF_6_)_2_ together with 100 equivalents of the hydroquinone. The conversion can easily be followed by the decline of the strong 425 nm band of (GFA3a)^2+^. An isosbestic point at 353 nm indicates clean conversion without formation of side products, being in line with the NMR experiments. The reaction was completed after ca. 53 min. Then, the experiment was repeated with 20, 40, and 80 equivalents of hydroquinone. The absorbance at 425 nm was plotted as a function of time for all applied equivalents of hydroquinone (Figure 9b). Obviously, the rate increases with the excess of the hydroquinone. The equation ln(A) = −*k*_obs_·t + ln(A_0_), where A is the time‐dependent absorbance at 425 nm for GFA3a(PF_6_)_2_ (456 nm for TGFA3(PF_6_)_2_) and A_0_ is the absorbance at t  = 0, was used to obtain the pseudo first‐order rate constant *k*_obs_ as the slope of the ln(A) versus time plot at the first minutes of the reaction. Finally, using the equation *k*_obs_ = *k*·[hydroquinone], the second‐order rate constant *k* was obtained as the slope of the plot *k*_obs_ versus the hydroquinone concentration (Figure 9c).

The UV–vis measurements and their analysis are included in the Supporting Information. The second‐order rate constants *k* obtained from these experiments are collected in Table 4. There are two important results from the kinetic analysis of the reactions with TGFA3(PF_6_)_2_ and GFA3a(PF_6_)_2_.

**TABLE 4:** Second‐order rate constants *k*(TGFA3) and *k*(GFA3a) determined from the UV‐vis spectroscopic experiments for TGFA3(PF_6_)_2_ and GFA3a(PF_6_)_2_, respectively.

Substrate	*k*(TGFA3) / l·s^−1^·mol^−1^	*k*(GFA3a) / l·s^−1^·mol^−1^
Hydroquinone	14.4 ± 0.7	0.17 ± 0.005
2‐chlorobenzene‐1,4‐diol	22.4 ± 0.7	1.50 ± 0.04
2‐bromobenzene‐1,4‐diol	24.9 ± 1.0	1.47 ± 0.09
2,5‐dibromobenzene‐1,4‐diol	39.9 ± 2.1	^a^

^a^ No clean reaction.

1) The reactions (hydroquinone oxidation as well as oxidative P–P coupling of phosphanes) are faster with TGFA3(PF_6_)_2_ than with GFA3a(PF_6_)_2_. This could be explained by the significantly higher potential of the tetrathioguanidine. According to the Marcus theory for electron transfer, the higher potential implies a higher electron‐transfer rate. Hence, this result indicates that the rate for the electron‐transfer step contributes to the overall rate *k*.

2) For both the tetrathioguanidine and the tetraguanidine, the rate increases with halogenation of the hydroquinone substrate. The estimated *E_o_*_x_ values (vs. NHE, in DMSO solution) increase from 1.61 V for hydroquinone to 1.73 V for 2‐chlorobenzene‐1,4‐diol and 2‐bromobenzene‐1,4‐diol [39]. This means that higher‐potential hydroquinones are oxidized faster than lower‐potential ones. At first glance, this result is in seeming contradiction to the first point and the Marcus theory for electron transfer (the reactions are not likely to fall into the Marcus inverted region). On the other hand, electron transfer is coupled to proton transfer in these reactions. The results can be rationalized by the dependence of the overall rate *k* on the rate of proton‐transfer, which is likely to correlate with the Lewis acidity of the hydroquinone derivative. The *pK*_a_ value decreases upon halogenation (e.g., the first *pK*_a_ values in DMSO are 19.1, 14.5, and 16.5 for hydroquinone, 2‐chlorobenzene‐1,4‐diol, and 2‐bromobenzene‐1,4‐diol, respectively [39]). Therefore, proton transfer should be facilitated by the introduction of electron‐withdrawing halides at the aromatic core of the hydroquinones. Due to the compensating effect arising from opposite trends of electron transfer and proton transfer, the 2e^−^/2H^+^ reduction potential does not change much upon halogenation (potentials in H_2_O vs. NHE of 0.69, 0.71, 0.67, and 0.73 V were reported for hydroquinone, 2‐chlorobenzene‐1,4‐diol, 2‐bromobenzene‐1,4‐diol, and 2,5‐dibromobenzene‐1,4‐diol, respectively) [22]. Moreover, similar bond dissociation free energies (BDFE) for hydroquinone and halo‐substituted derivatives were reported (average BDFE values in H_2_O of 283, 286, and 290 kJ mol^−1^ for hydroquinone, 2‐chlorobenzene‐1,4‐diol, and 2‐bromobenzene‐1,4‐diol, respectively [1]). It should be stressed that these thermodynamic considerations are not necessarily sufficient to explain the trends in the kinetics. Moreover, the thermodynamics as well as the kinetics might be affected by the formation of donor–acceptor complexes preceding electron and proton transfer. In this case, arguments that are purely based on the properties of the individual reactants are not adequate. On the other hand, the experiments (e.g., the NMR spectra) gave no indications for the formation of such complexes. Hydrogen bonding between the hydroquinone (hydrogen‐bond donor) and (TGFA3)^2+^ or (GFA3a)^2+^ (hydrogen‐bond acceptor) is hampered due to the positive charge on the guanidino groups. In summary, the results indicate contributions from electron and proton transfer to the overall reaction rate.

**SCHEME 3:**
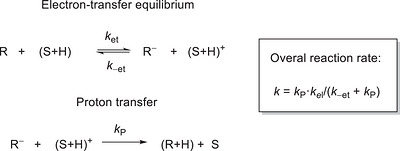
General two‐step reaction sequence for hydrogen transfer between a PCET reagent R and a substrate S‐H, in which an initial electron transfer equilibrium is followed by (irreversible) proton transfer. The overall rate depends on all three rates *k*_et_, *k*_−et_, and *k*_P_. For simplicity, only a (1e^−^,1H^+^) process is formulated.

Previous studies concerning PCET reactivity of quinones indicated that these reactions are often initiated by an electron transfer equilibrium, which is followed by a proton transfer step. Scheme 3 formulates such a two‐step mechanism for the simple case of a (1e^−^,1H^+^) reaction. Thereby, the rate constants *k*_el_ and *k*_−el_ for the electron‐transfer equilibrium and the rate constant *k*_P_ for the following (irreversible) proton transfer enter into the rate equation. There are strong indications that reactions with redox‐active guanidines also generally follow such a mechanism [29]. Hence, substrates with an oxidation potential more than 1 V higher than the reduction potential of the quinone/guanidine typically do not react [29, 40]. Moreover, we observed a strong kinetic isotope effect (*k*_H_/*k*_D_ = 5.6) for the reaction of GFA1a(PF_6_)_2_ with 10‐methyl‐9,10‐dihydroacridane [29].

For reactions of TGFA3 or GFA3a with hydroquinones, a concerted mechanism is also possible (e^−^ and H^+^ transfer in one step). Anyway, for a stepwise mechanism according to Scheme 3 and for a concerted mechanism, the overall rate depends on electron as well as proton transfer. The interplay between these two processes is responsible for the faster oxidation of high‐potential than of low‐potential quinones.

The results indicate that redox‐active guanidines and thioguanidines are of interest as redox mediators in PCET and dehydrogenative coupling reactions with high‐potential quinones, as they can efficiently reoxidize the hydroquinone by‐product. The use of (GFA3a)^2+^ for the oxidation of high‐potential hydroquinones is additionally motivated by its efficient regeneration from (GFA3a+2H)^2+^ by catalytic oxidation with dioxygen. This opens up the possibility to design cascade reactions (see Figure 1c for comparison), in which the quinone and GFA3a are applied in catalytic amounts. The diprotonated tetraguanidine, (GFA3a+2H)^2+^, is oxidized by O_2_ (in the presence of a copper catalyst) to give the dicationic, oxidized (GFA3a)^2+^, that re‐oxidizes the hydroquinone to the quinone. The high‐potential quinone then oxidizes a substrate, *S*_red_ + 2H or 2(*S*_red_ + H), to give S_ox_ or a coupling product S_ox_─S_ox_, and is again converted to the hydroquinone. In this way, new multistep reactions can be designed that could be valuable additions to the aerobic oxidation reactions of organic molecules by multistep electron transfer developed by Bäckvall and others [41].

## 3 Conclusions

In this work, three new redox‐active oligothioguanidines were synthesized, in which two (TGFA1), three (TGFA2), or four (TGFA3) thioguanidino groups are bound to benzene. They exhibit significantly higher redox potentials (*E*_1/2_ values) than the corresponding oligoguanidines. Of these compounds, TGFA3 can be oxidized to give stable salts of the dication (TGFA3)^2+^. Remarkably, these salts can be di‐protonated to give tetracations (TGFA+2H)^4+^, in which two opposite guanidino groups are protonated, and the other two guanidino groups are responsible for charge delocalization. Subsequently, salts of (TGFA3)^2+^ were applied first in dehydrogenative P–P coupling reactions. The results show that dehydrogenative coupling of phosphanes to diphosphanes (accompanied by reduction and di‐protonation of the thioguanidine) proceeds very fast and quantitatively at room temperature; it is much faster than with the corresponding oxidized tetraguanidines (GFA3a)^2+^ (with four tetramethylguanidino groups) and (GFA3b)^2+^ (with four *N*,*N*’‐dimethyl(ethylene)guanidino groups).

Moreover, (TGFA3)^2+^ and (GFA3a)^2+^ both efficiently oxidize hydroquinones and halogenated high‐potential derivatives. The determination of the second‐order rate constants for hydroquinone oxidation showed that oxidation of hydroquinones to quinones is faster with (TGFA3)^2+^ than with (GFA3a)^2+^. Seemingly paradoxically, high‐potential halogenated hydroquinones react faster than low‐potential hydroquinone, both for (TGFA3)^2+^ and (GFA3a)^2+^. These results point to a significant contribution of the rate for proton transfer to the overall reaction rate. Quantum‐chemical calculations give detailed information about the interplay between electron and proton transfer in these reactions. They show that (TGFA3)^2+^ exhibits a higher redox potential than (GFA3a)^2+^, in nice quantitative agreement with the experimental results. On the other hand, TGFA3 is a weaker base than GFA3a, and therefore TGFA3 is only a slightly stronger oxidant in PCET reactions than GFA3a. The higher redox potential of TGFA3 might be responsible for the higher rate of PCET reactions with TGFA3, since these reactions are initiated by electron transfer.

The results of this work motivate the use of redox‐active guanidines and thioguanidines as redox mediators in PCET and dehydrogenative coupling reactions with high‐potential quinones. In such reactions, the redox‐active guanidines or thioguanidines are responsible for the re‐oxidation of the hydroquinones. Especially, the use of (GFA3a)^2+^ is attractive due to its efficient regeneration from (GFA3a + 2H)^2+^ by catalytic oxidation with dioxygen.

## 4 Experimental Details

The synthesis details and analytical data for all compounds and information about the quantum‐chemical calculations are included in the Supporting Information. Deposition Numbers 2505945 for TGFA1, 2505946 for TGFA2, 2505947 for TGFA3, 2505948 for TGFA3(PF_6_)_2_, 2505949 for {[AgCH_3_CN(TGFA2)](PF_6_)}*_n_*, 2505950 (TGFA1+2H)(PF_6_)_2_, 2505951 for (TGFA3+2H)(PF_6_)_2_, 2505952 for (TGFA3+2H)(BF_4_)_4_ contain the supplementary crystallographic data for this paper. These data are provided free of charge by the joint Cambridge Crystallographic Data Centre and Fachinformationszentrum Karlsruhe Access Structure service.4.1 4.2.

## Conflicts of Interest

The authors declare no conflicts of interest.

## Supporting information



The authors have cited additional references within the Supporting Information [42, 43, 44, 45, 46, 47, 48, 49, 50, 51, 52, 53, 54, 55, 56, 57, 58, 59, 60, 61, 62, 63, 64, 65, 66, 67, 68, 69, 70, 71]. [Correction updated on 14 April 2026 ‐ the reaction equations in the Supporting Information were updated.]
