# Supplementary material for: Hydroquinone Oxidation by Redox‐active Guanidines and Thioguanidines: Faster Conversion to High‐Potential Than to Low‐Potential Quinones
Source: Chemistry. 2026 Apr 10;32(24):e70981. doi: 10.1002/chem.70981 (PMC13290418; doi:10.1002/chem.70981)
Supplement: Supplementary file 1 — The authors have cited additional references within the Supporting Information [42, 43, 44, 45, 46, 47, 48, 49, 50, 51, 52, 53, 54, 55, 56, 57, 58, 59, 60, 61, 62, 63, 64, 65, 66, 67, 68, 69, 70, 71]. [Correction updated on 14 April 2026 ‐ the reaction equations in the Supporting Information were updated.] [file CHEM-32-e70981-s001.pdf]

Content

Content 1

General Information 2

Synthesis of TGFA1 4

Attempt to oxidise TGFA1 with diacetyliodobenzene / NH_4PF_6 leading to $(TGFA+2H)(PF_6)_2$ 7

Synthesis of TGFA2 10

Protonation of TGFA2 to give $(TGFA2+2H)(PF_6)_2$ 13

Attempt to oxidise TGFA2 with $AgPF_6$ leading to the coordination polymer $\{[AgCH_3CN(TGFA2)](PF_6)\}_n$ 16

Synthesis of TGFA3 18

Protonation of TGFA3 to give $(TGFA3+2H)(PF_6)_2$ 22

Oxidation of TGFA3 with ferrocenium hexafluorophosphate to give $TGFA3(PF_6)_2$ 24

Oxidation of TGFA3 with acetylferrocenium tetrafluoroborate to give $TGFA3(BF_4)_2$ 27

Protonation experiments of $(TGFA3)^{2+}$ with $HBF_4{\cdot}Et_2O$ 29

Titration of $TGFA3(PF_6)_2$ with $HBF_4{\cdot}Et_2O$: NMR experiment 29

Titration of $TGFA3(PF_6)_2$ with $HBF_4{\cdot}Et_2O$: UV-vis experiment 31

Preparation of $(TGFA3+2H)(BF_4)_4$ 32

Catalytic oxidation of $(GFA3+2H)(PF_6)_2$ with O_2 35

Dehydrogenative P-P coupling of diarylphosphanes: reaction of $TGFA3(PF_6)_2$ with $(MeOPh)_2PH$ to give $(MeOPh)_2P\text{-}P(MeOPh)_2$ 37

Dehydrogenative P-P coupling of diarylphosphanes: reaction of $GFA3b(PF_6)_2$ with $(MeOPh)_2PH$ to give $(MeOPh)_2P\text{-}P(MeOPh)_2$ 39

Oxidation of hydroquinones and derivatives to quinones 42

NMR experiments 42

Hydroquinone oxidation 42

2-Chlorobenzene-1,4-diol oxidation 46

2-Bromobenzene-1,4-diol oxidation 48

2,5-Dibromobenzene-1,4-diol oxidation 50

UV-vis experiments 52

Hydroquinone oxidation 52

2-Chlorobenzene-1,4-diol oxidation 58

2-Bromobenzene-1,4-diol oxidation 63

2,5-Dibromobenzene-1,4-diol oxidation 68

Details for the crystal structure determinations 73

Computational Details 76

General Information

All synthetic work was carried out using standard Schlenk techniques under argon atmosphere. The solvents acetonitrile, dichloromethane, diethyl ether, toluene and tetrahydrofurane were dried with a MBraun MB-SPS-800 Solvent Purification System and stored over molecular sieves. Other solvents were purchased from Acros Organics. The following chemicals were purchased and used as delivered: hydroquinone (> 99.5%, Sigma-Aldrich), 2-chlorobenzene-1,4-diol (90%, BLD pharm, 87.5% according to an NMR experiment with hexamethylbenzene as internal standard), 2-bromobenzene-1,4-diol (90%, BLD pharm, 94% according to an NMR experiment with hexamethylbenzene as internal standard), 2,5-dibromobenzene-1,4-diol (98.38%, BLD pharm, 97% according to an NMR experiment with hexamethylbenzene as internal standard), $AgPF_6$ (99%, abcr), 1,4-diaminobenzene·2 HCl (>99%, Sigma-Aldrich), di(4-methoxyphenyl)phosphine (95%, Sigma-Aldrich), $HBF_4·Et_2O$ (Sigma-Aldrich), 4-nitro-1,2-phenylenediamine (>97%, TCI), diacetyliodobenzene (> 99%, Fluka), NH_4PF_6 (99%, Acros Organics), 1,2,4,5-tetraaminobenzene·4 HCl, Pd on charcoal (10 wt%, Sigma-Aldrich), hydrazinium hydroxide (about 100%, Sigma-Aldrich), copper(II)-tetrafluoroborate hexahydrate (96%, Sigma-Aldrich), copper(II) chloride (Sigma-Aldrich, ≥ 98%) and thiazolin-2-one (97%, BLD pharm). The synthesis of ferrocenium hexafluorophosphate,[1] acetylferrocenium tetrafluoroborate,[2] GFA3a$(PF_6)_2$,[3] GFA3b$(PF_6)_2$,[4] and 2-chloro-3-methyl-2-thiazolidinium chloride[5] (with oxalylchloride instead of phosgene or thionylchloride) followed the literature procedure. Elemental analyses were performed at the Microanalytical Laboratory of Heidelberg University using the vario EL and vario MICRO cube devices from Elementar Analysensysteme GmbH. Please note that the compounds are strong Brønsted bases and redox-active. Therefore, some deviations are caused by reactions with traces of water or dioxygen during the elemental analysis measurements. NMR spectra were recorded on a Bruker Avance II 400, Bruker AVANCE III 600 or Bruker 600 Ultrashield system. Solvent resonances were taken as references for all ^{1}H NMR spectra. UV-Vis spectra were recorded with a Cary 5000 spectrophotometer. CV measurements were carried out with a Metrohm Autolab PGSTAT 204 potentiostate/galvanostate and an Ag/AgCl reference electrode, Pt rod counter electrode and glassy carbon working electrode. All voltammograms were recorded at room temperature. CH_2Cl_2 and CH_3CN were used as solvents for the individual compounds (concentration around 10^{-3} M, if not stated otherwise), whereas $n$$Bu_4N(PF_6)$ (electrochemical grade (≥99.0%), Fluka) was employed as supporting electrolyte (c = 0.1 M). The potentials are given vs. the reference redox pair ferrocenium/ferrocene (Fc^+/Fc), measured at $E_{1/2}$ = 0.45 V in CH_3CN and 0.46 V in CH_2Cl_2. HR-ESI spectra were recorded with a Bruker ApexQe hybrid 9.4 T FT-ICR spectrometer or a Thermo Scientific Q Exactive and HR-MALDI with a Bruker timsTOFfleX.

X-ray crystallography

Suitable crystals for single-crystal structure determination were taken directly from the mother liquor, taken up in per-fluorinated polyether oil and fixed on a cryo loop. Full shells of intensity data were collected at low temperature with a Bruker D8 Venture, dual source (Mo-Kα radiation, microfocus X-ray tube, Photon III detector). Data were processed with the standard Bruker (SAINT, APEX3/4) software package.[6] Multiscan absorption correction was applied using the SADABS program.[7] The structures were solved by intrinsic phasing[8] and refined using the SHELXTL software package (Version 2018/3).[9] Graphical handling of the structural data during solution and refinement were performed with OLEX2.[10] All non-hydrogen atoms were given anisotropic displacement parameters. Hydrogen atoms bound to carbon were input at calculated positions and refined with a riding model. Hydrogen atoms bound to nitrogen were located in difference Fourier syntheses and refined, either fully or with appropriate distance and/or symmetry. Split atom models were used to refine disordered groups and/or solvent molecules. When found necessary, suitable geometry and adp restraints were applied.[11,12] Due to severe disorder and fractional occupancy, electron density attributed to the solvent of crystallization was removed from some of the structures with the BYPASS procedure,[13] as implemented in PLATON (squeeze/hybrid).

Deposition Numbers 2505945 for TGFA1, 2505946 for TGFA2, 2505947 for TGFA3, 2505948 for TGFA3(PF_6)$_2$, 2505949 for {[AgCH$_3$CN(TGFA2)](PF_6)}$_n$, 2505950 (TGFA1+2H)(PF_6)$_2$, 2505951 for (TGFA3+2H)(PF_6)$_2$, and 2505952 for (TGFA3+2H)(BF_4)$_4$ contain the supplementary crystallographic data for this paper. These data are provided free of charge by the joint Cambridge Crystallographic Data Centre and Fachinformationszentrum Karlsruhe Access Structure service.

Synthesis of TGFA1

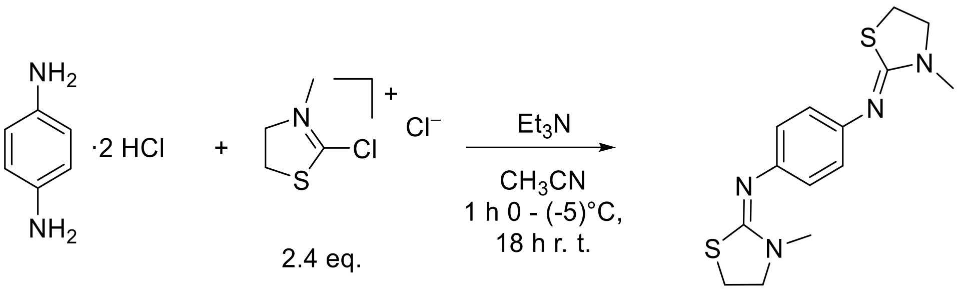

1,4-Diaminobenzene·2 HCl (120 mg, 0.66 mmol) was suspended in 8 ml CH_3CN and then 0.65 ml (4.69 mmol) of triethylamine was added. 2-Chloro-3-methyl-2-thiazolidinium chloride (277 mg, 1.61 mmol) was suspended in 10 ml CH_3CN and cooled to 0 - (-5) °C (ice/ethanol). This suspension was added dropwise to the activated urea using a pasteur pipette, stirred for 1 h at low temperature and overnight at room temperature (during which time the ice bath slowly thaws). The solvent was removed at reduced pressure and the residue was re-dissolved in some water and HCl (10%, pH < 3) and extracted three times with 20 ml CH_2Cl_2. The aqueous phase was adjusted to pH > 12 with NaOH (25%) and extracted 4 times with 20 ml CH_2Cl_2 (until the organic phase is almost colourless). The combined organic phases were dried over K_2CO_3. After filtration and removal of the solvent, 191.5 mg (0.57 mmol, 87%) of a beige-coloured powder was obtained. Crystals were grown from a CH_2Cl_2 solution layered with *n*-hexane.

Elemental analysis calcd. (%) for $C_{14}H_{18}N_4S_2 \cdot 0.33\ CH_2Cl_2$ (334.76 g mol^{-1}): C 51.54, H 5.62, N 16.74; found C 51.99, H 5.92, N 16.88.

^{1}H NMR (600.18 MHz, $CDCl_3$, 295 K): δ = 6.86 (s, 4 H, CH_{arom}), 3.55 (t, J = 6.8 Hz, 4 H, CH_2), 3.12 (t, J = 6.8 Hz, 4 H, CH_2), 3.02 (s, 6 H, CH_3) ppm.

^{13}C NMR (150.92 MHz, $CDCl_3$, 295 K): δ = 159.34 (C_q, C_{gua}), 147.80 (C_q, C_{arom}), 122.31(CH_{arom}), 53.01, 26.77 (CH_2), 33.84 (CH_3) ppm.

HR-MS (HR-ESI$^+$, CH_2Cl_2): m/z (%) = calcd. for $[C_{14}H_{19}N_4S_2]^+$ 307.1046, found 307.1042 (100).

UV-vis (CH_3CN, c = $6.57 \cdot 10^{-5}$ M): λ (ε in l mol^{-1} cm^{-1}) = 286 (18430) nm.
UV-vis (CH_2Cl_2, c = $6.33 \cdot 10^{-5}$ M): λ (ε in l mol^{-1} cm^{-1}) = 289 (16120) nm.

Analytical data for TGFA1

^{1}H NMR spectrum (600.18 MHz, 295 K) of TGFA1 in $CDCl_3$

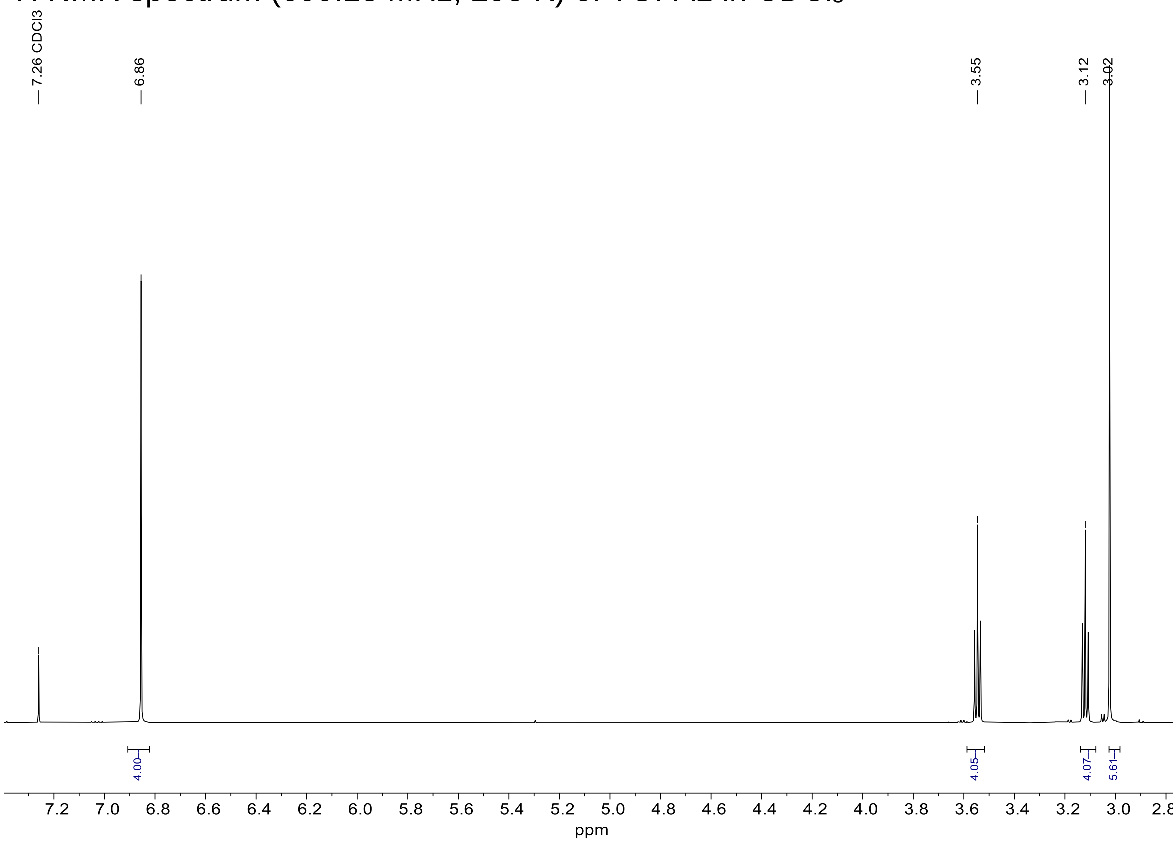

^{13}C NMR spectrum (150.92 MHz, 295 K) of TGFA1 in $CDCl_3$

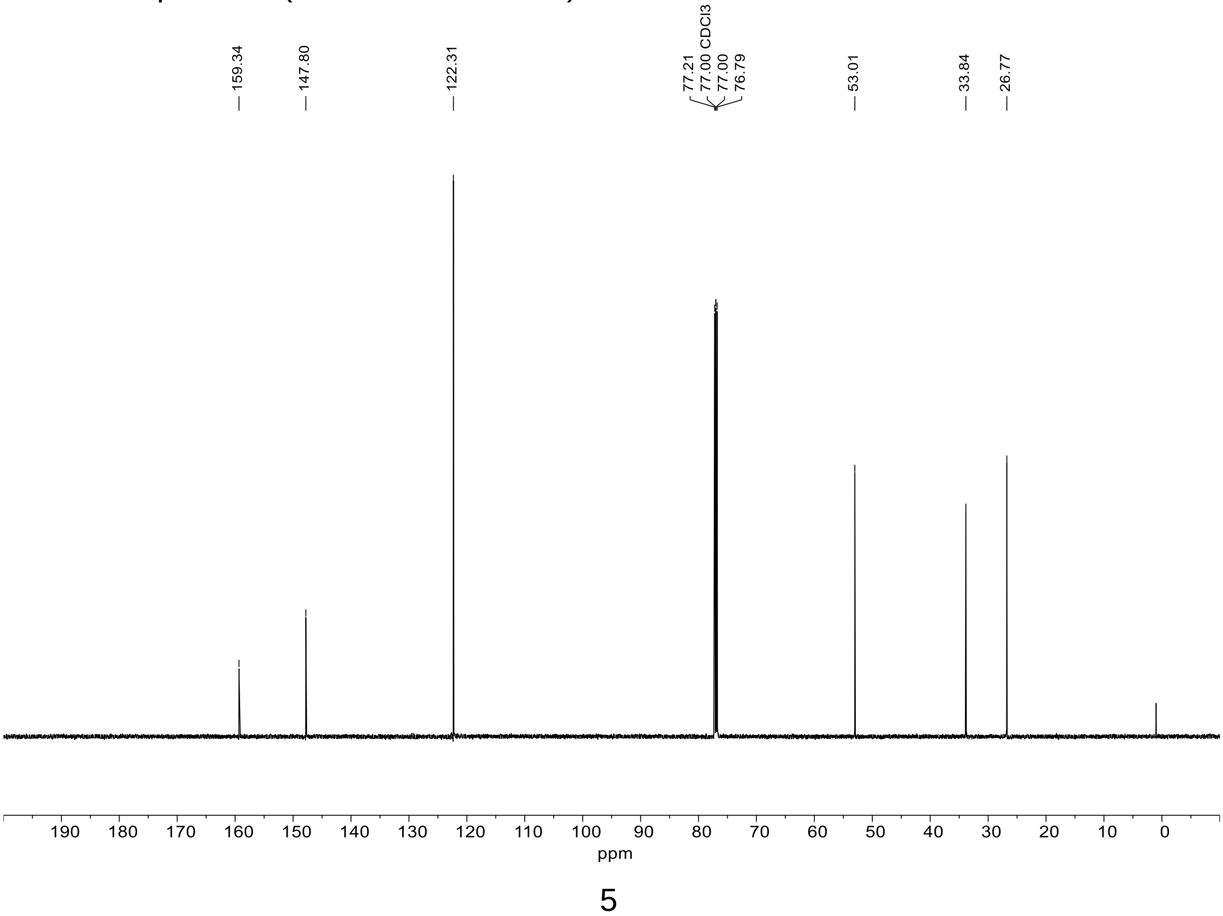

UV-vis spectra of TGFA1 in CH_3CN and CH_2Cl_2

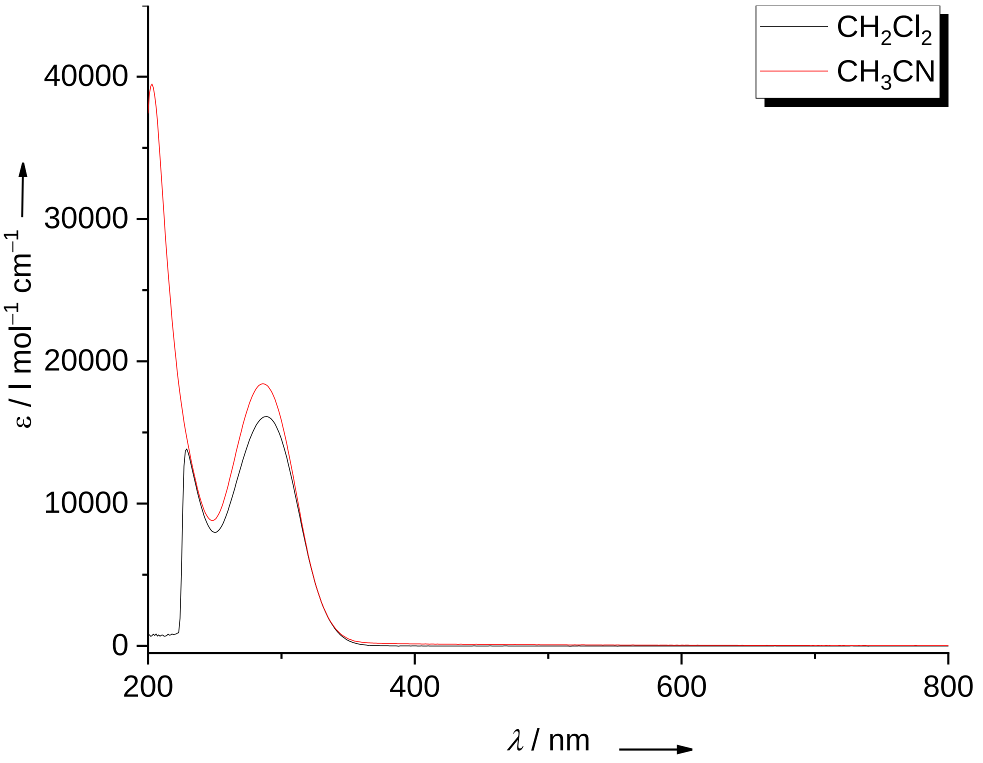

Cyclic voltammetry curves for TGFA1 in CH_2Cl_2 ($^nBu_4NPF_6$ as supporting electrolyte). Potentials given vs. the ferrocenium/ferrocene (Fc^+/Fc) redox couple. For comparison, the CV curve of GFA1b is also included.

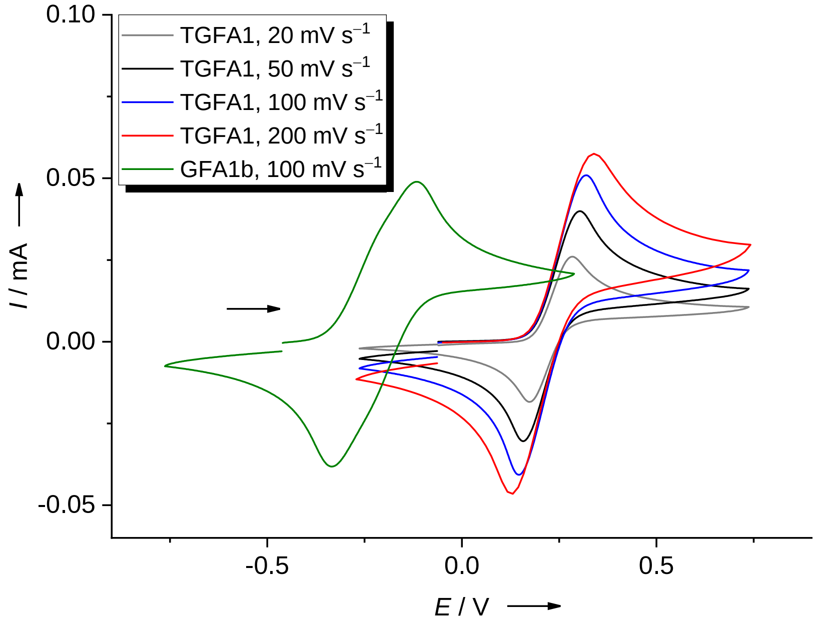

Cyclic voltammetry curves for TGFA1 in CH_3CN ($^nBu_4NPF_6$ as supporting electrolyte). Potentials given vs. the ferrocenium/ferrocene (Fc^+/Fc) redox couple. For comparison, the CV curve of GFA1b is also included.

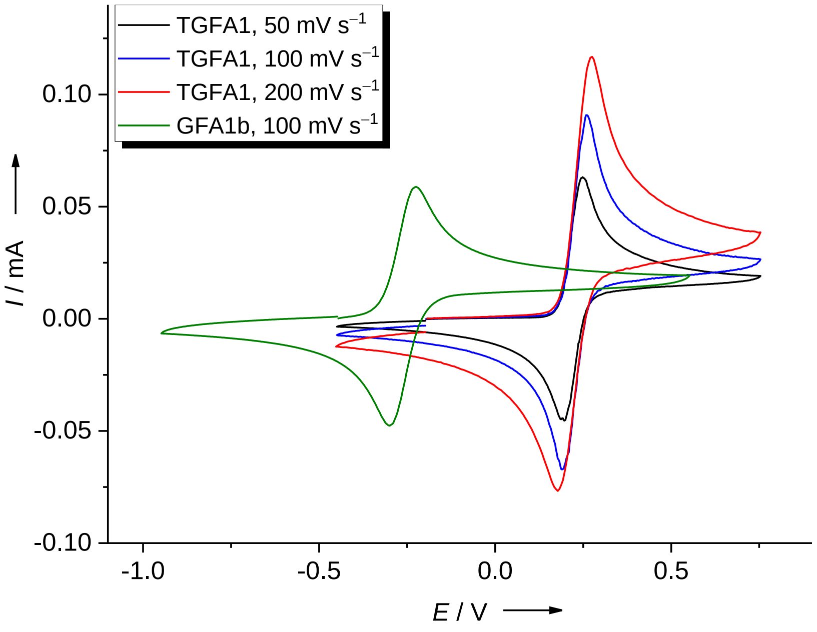

Attempt to oxidise TGFA1 with diacetyliodobenzene / NH_4PF_6 leading to (TGFA+2H)$(PF_6)_2$

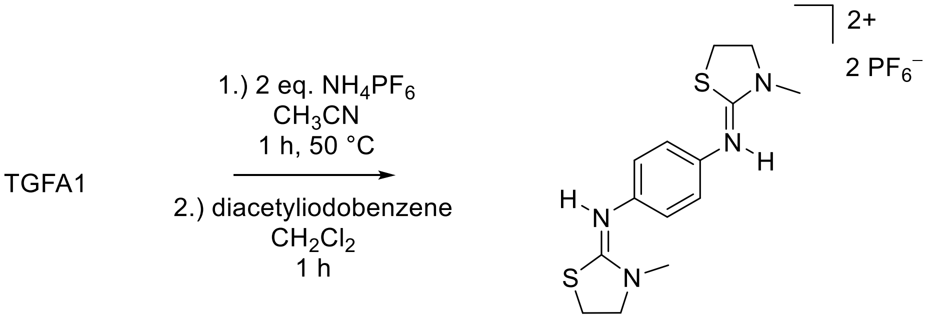

21.6 mg (70.6 µmol) of TGFA1 and 22.9 mg (141 µmol) of NH_4PF_6 were dissolved in 4 ml CH_3CN and heated to 50 °C for 1 h. The solvent was removed under reduced pressure and the residue washed 3 times with 5 ml Et_2O. Then, 22.7 mg (70.5 µmol) diacetyliodobenzene and 5 ml of CH_2Cl_2 were added and the reaction mixture stirred for 1 h at room temperature. The solvent was removed under reduced pressure and the residue washed 2 times with 5 ml toluene and 2 times with 5 ml Et_2O to give 27.4 mg (45.8 µmol, 65%) of a brownish powder. Colourless crystals suitable for structural characterization with SC-XRD were obtained after a few days by re-dissolving the residue in CH_3CN and layering with Et_2O.

Elemental analysis calcd. (%) for $C_{14}H_{20}N_4S_2P_2F_{12}$ (598.39 g mol^{-1}): C 28.10, H 3.37, N 9.36; found C 27.70, H 3.62, N 8.75.

^{1}H NMR (600.18 MHz, CD_3CN, 295 K): δ = 7.36 (s, 4 H, CH_{arom}), 4.06 (*t*, *J* = 7.8 Hz, 4 H, CH_2), 3.44 (dd, *J* = 8.2, 7.3 Hz, 4 H, CH_2), 3.20 (s, 6 H, CH_3) ppm.

^{13}C NMR (150.92 MHz, CD_3CN, 295 K): δ = 170.66 (C_q, C_{gua}), 139.77 (C_q, C_{arom}), 127.16 (CH_{arom}), 57.90, 29.22 (CH_2), 35.51 (CH_3) ppm.

^{31}P NMR (242.96 MHz, CD_3CN, 295 K): δ = –144.62 (sept) ppm.

^{19}F NMR (150.92 MHz, CD_3CN, 295 K): δ = –72.22, –73.48 ppm.

HR-MS (ESI$^+$, CH_3CN): *m/z* (%) = calcd. for $[C_{14}H_{19}N_4S_2]^+$ 307.1046, found 307.1039 (100).

UV-vis (CH_3CN, c = $7.05 \cdot 10^{-5}$ M): λ (ε in l mol^{-1} cm^{-1}) = 247 (16060), 272 (sh, 12990) nm.

Analytical data for (TGFA1+2H)$(PF_6)_2$

^{1}H NMR spectrum (600.18 MHz, 295 K) of (TGFA1+2H)$(PF_6)_2$ in CD_3CN

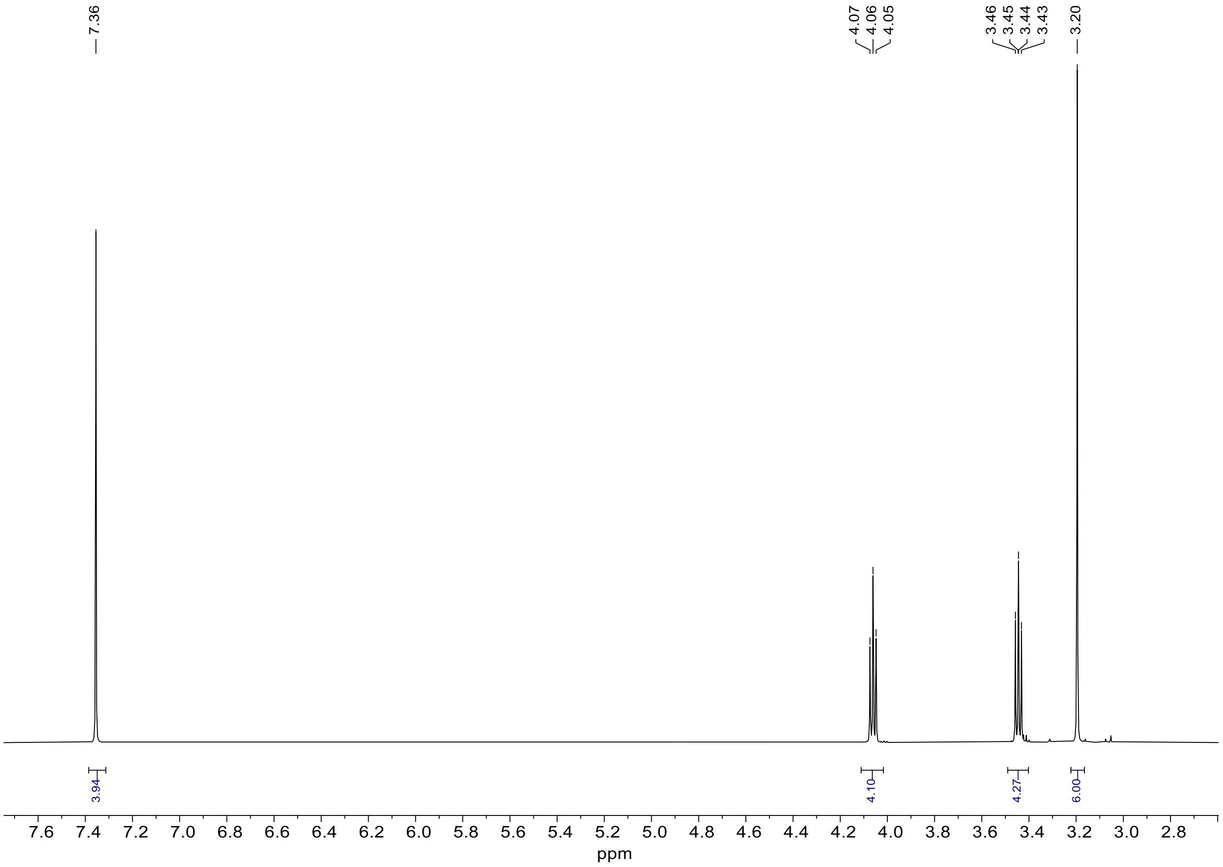

^{13}C NMR spectrum (150.92 MHz, 295 K) of $(TGFA1+2H)(PF_6)_2$ in CD_3CN

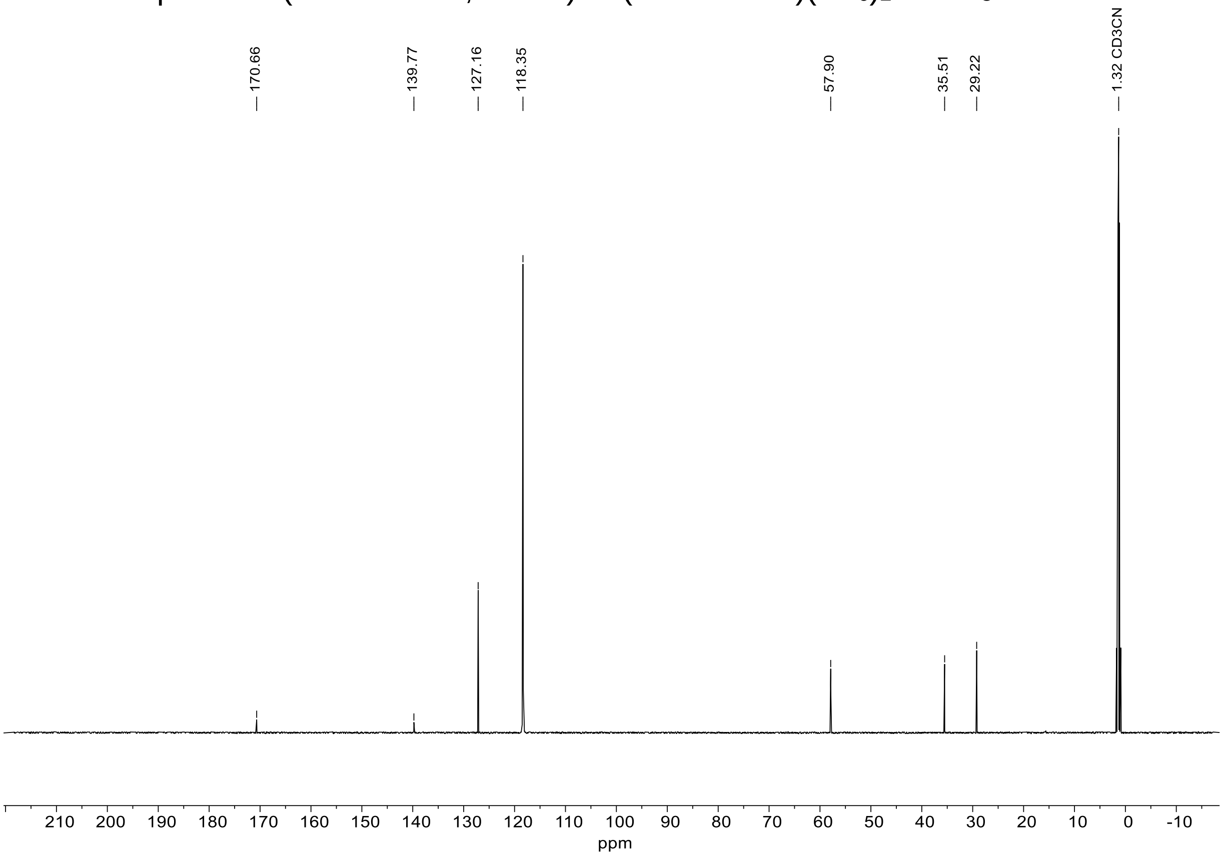

UV-vis spectrum of $(TGFA1+2H)(PF_6)_2$ in CH_3CN

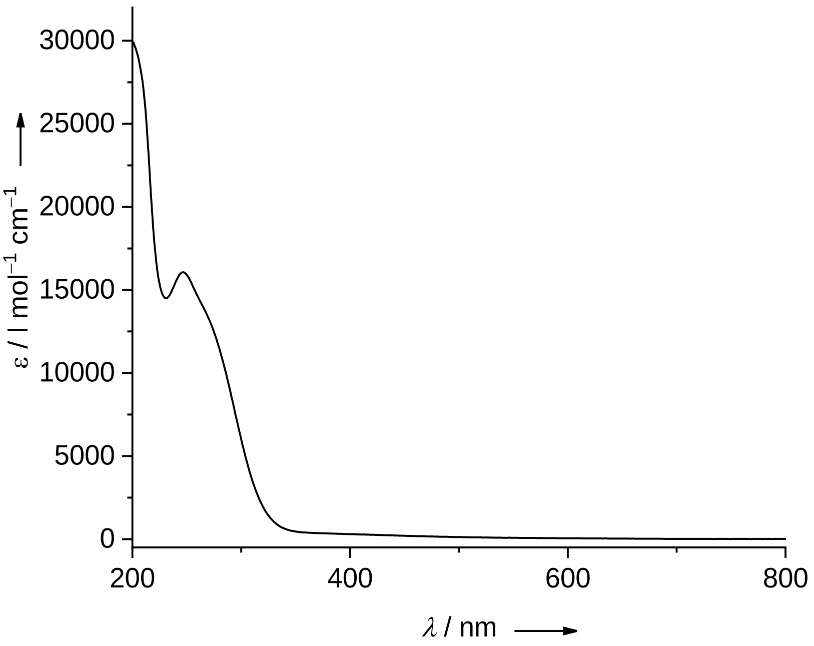

Synthesis of TGFA2

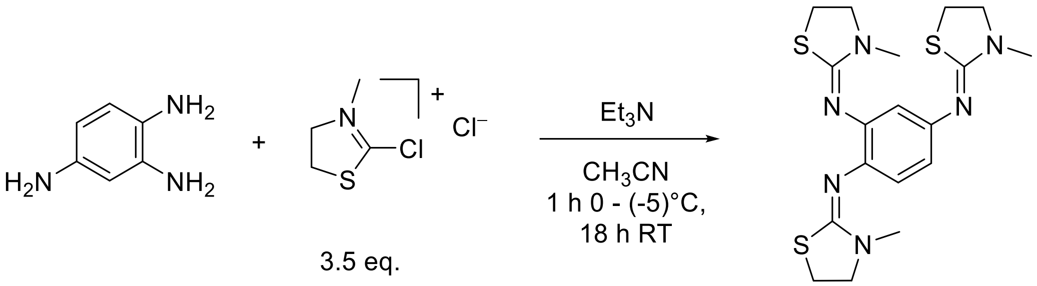

1,2,4-Triamino-benzene. The synthesis followed a literature protocol for the preparation of 2-nitrobenzene-1,4-diamine from 2-methoxy-4-nitroaniline (Dorok, Sascha, Eur. Pat. Appl. 2963010, 2016, EP 2 963 010 A1). 153 mg (1 mmol) of 4-nitro-1,2-diaminobenzene and 45 mg of Pd on charcoal (10 wt%) were suspended/dissolved in 5 ml THF. Then, 0.5 ml (10 mmol) of hydrazine-monohydrate was added slowly and dropwise, and the resulting reaction mixture stirred for 3 h at a temperature of 90 °C. The mixture was filtrated over celite and washed several times with THF. Subsequently, the solvent was removed under vacuum.

Then, the 1,2,4-triaminobenzene was suspended in 15 ml CH_3CN and mixed with 1.5 ml Et_3N (10.8 mmol). The suspension was cooled to 0 - (-5) °C with an ice/ethanol bath and a suspension of 608.4 mg (3.54 mmol) 2-chloro-3-methyl-2-thiazolidinium chloride in 10 ml CH_3CN was slowly added. The reaction solution was stirred overnight (slow thawing to r. t.). The solvent was removed under vacuum. Then, the residue was re-dissolved in some H_2O and HCl (10%, pH < 3) and extracted three times with 20 ml CH_2Cl_2. The aqueous phase was adjusted to pH > 12 with NaOH (25%) and extracted 4 times with 25 ml CH_2Cl_2 (until the CH_2Cl_2 phase was almost colourless). The combined organic phases were dried over K_2CO_3. After filtration and removal of the solvent, 416.3 mg (0.95 mmol, 95%) of a beige-coloured powder was obtained. Crystals suitable for structural characterization with SC-XRD were grown from a CH_2Cl_2 solution layered with *n*-hexane.

Elemental analysis calcd. (%) for $C_{18}H_{24}N_6S_3 \cdot 0.17\ CH_2Cl_2$ (437.60 g mol^{-1}): C 49.95, H 5.62, N 19.21; found C 50.50, H 5.51, N 19.32.

^{1}H NMR (600.18 MHz, $CDCl_3$, 295 K): δ = 6.80 (d, *J* = 8.2 Hz, 1 H, CH_{arom}), 6.57 – 6.53 (m, 1 H, CH_{arom}), 6.51 (d, *J* = 2.4 Hz, 1 H, CH_{arom}), 3.51 (t, *J* = 6.8 Hz, 2 H, CH_2), 3.49 – 3.46 (m, 4 H, CH_2), 3.11 – 3.06 (m, 6 H, CH_2), 3.00 (s, 3 H, CH_3), 2.99 (s, 3 H, CH_3), 2.98 (s, 3 H, CH_3) ppm.

^{13}C NMR (150.92 MHz, $CDCl_3$, 295 K): δ = 159.22, 159.02, 158.88 (C_q, C_{gua}), 148.30, 144.67, 140.07 (C_q, C_{arom}), 121.95, 117.18, 115.02 (CH_{arom}), 53.18, 53.15, 53.01, 26.95, 26.89, 26.81 (CH_2), 34.04, 33.87 (CH_3) ppm.

HR-MS (HR-ESI$^+$, CH_2Cl_2): *m/z* (%) = calcd. for $[C_{18}H_{25}N_6S_3]^+$ 421.1297, found 421.1292 (70).

UV-vis (CH_3CN, c = $6.04 \cdot 10^{-5}$ M): λ (ε in l mol^{-1} cm^{-1}) = 228 (sh, 28230), 255 (sh, 22540), 293 (sh, 17415) nm.

UV-vis (CH_2Cl_2, c = $4.57\cdot10^{-5}$ M): λ (ε in l mol^{-1} cm^{-1}) = 229 (29175), 255 (sh, 23000), 293 (sh, 17370) nm.

Analytical data for TGFA2

^{1}H NMR spectrum (600.18 MHz, 295 K) of TGFA2 in $CDCl_3$

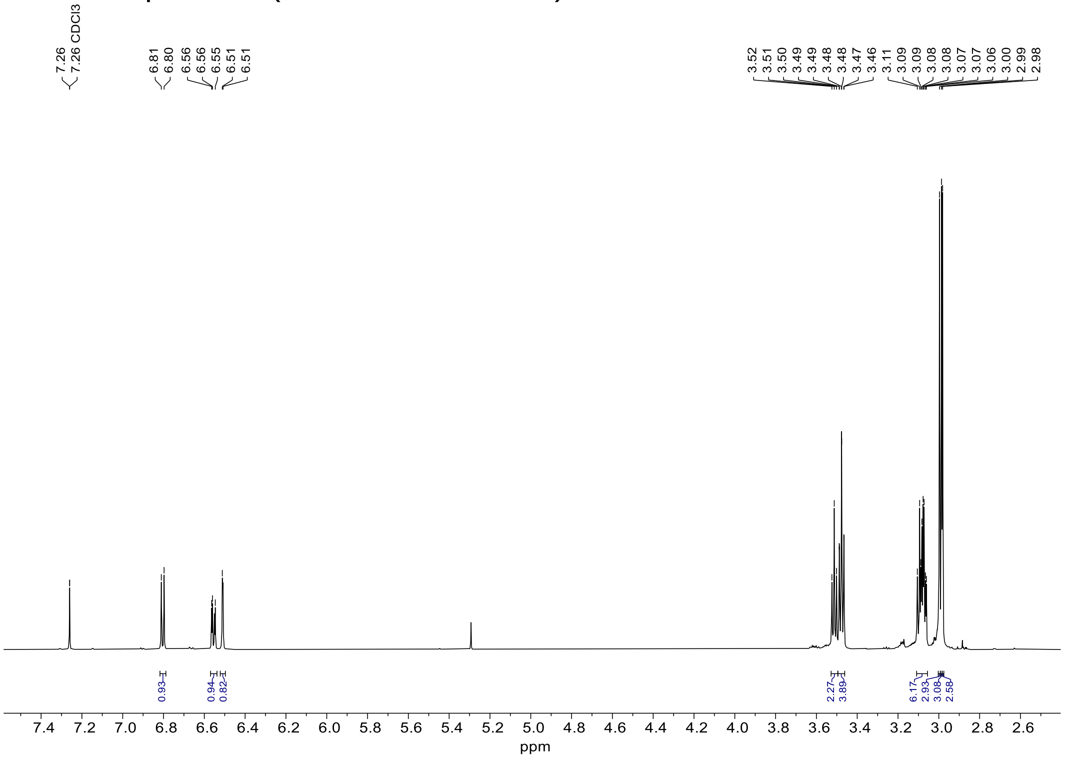

^{13}C NMR spectrum (150.92 MHz, 295 K) of TGFA2 in $CDCl_3$

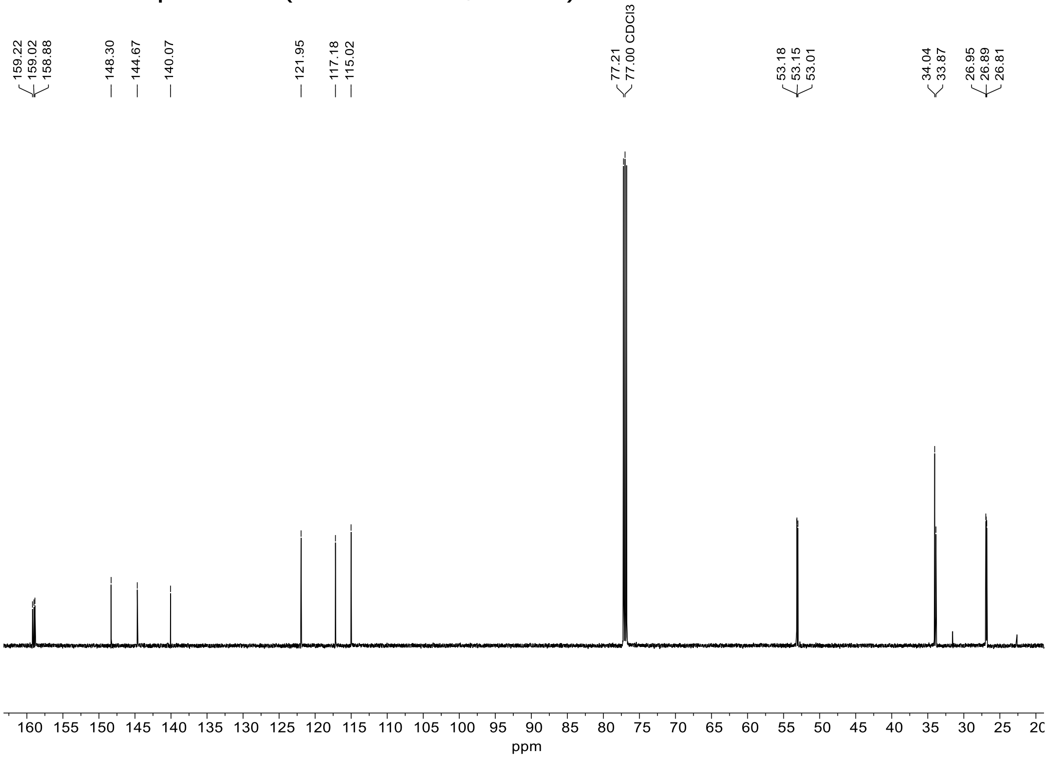

UV-vis spectra of TGFA2 in CH_3CN and CH_2Cl_2

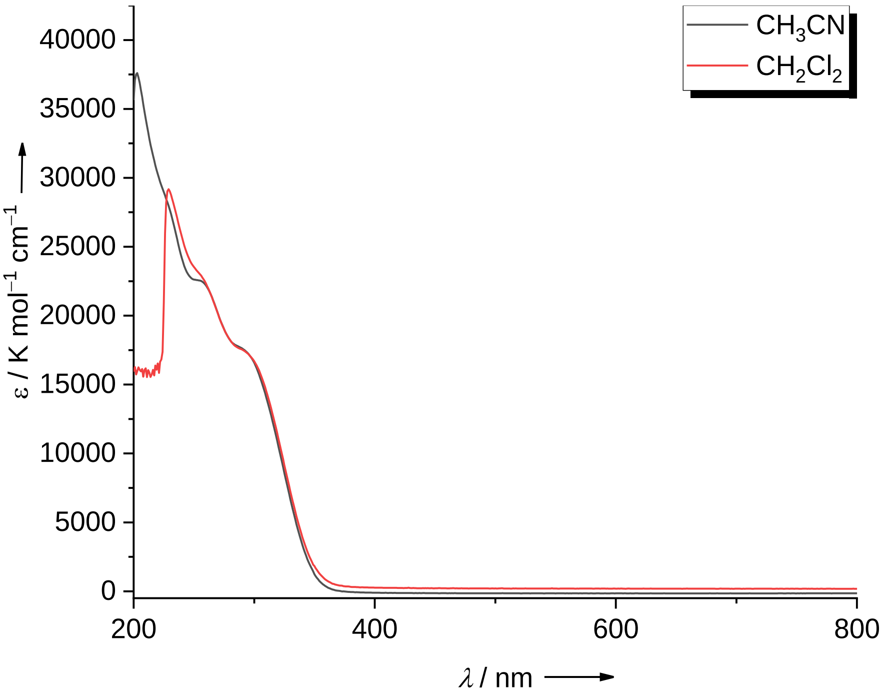

Cyclic voltammetry curves for TGFA2 in CH_2Cl_2 ($^{n}Bu_4NPF_6$ as supporting electrolyte). Potentials given vs. the ferrocenium/ferrocene (Fc^+/Fc) redox couple. For comparison, the CV curve of GFA2b is also included.

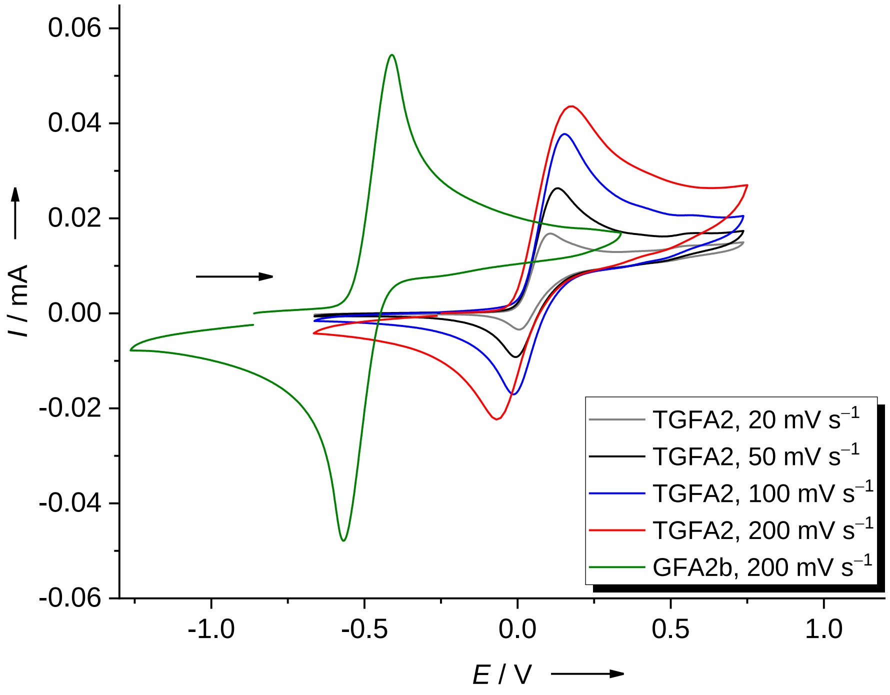

Cyclic voltammetry curves for TGFA2 in CH_3CN ($^nBu_4NPF_6$ as supporting electrolyte). Potentials given vs. the ferrocenium/ferrocene (Fc^+/Fc) redox couple. For comparison, the CV curve of GFA2b is also included.

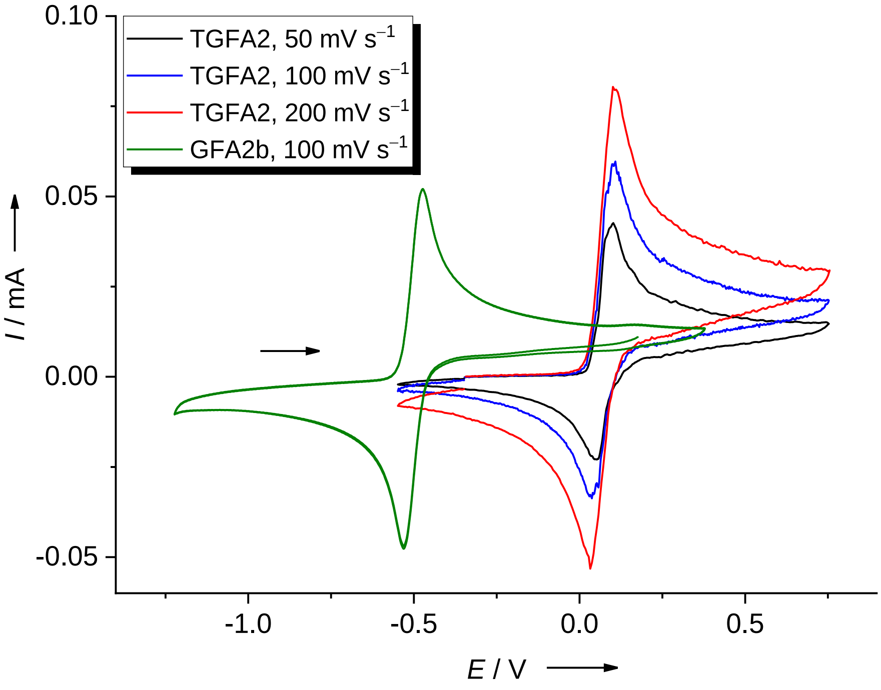

Protonation of TGFA2 to give (TGFA2+2H)(PF_6)$_2$

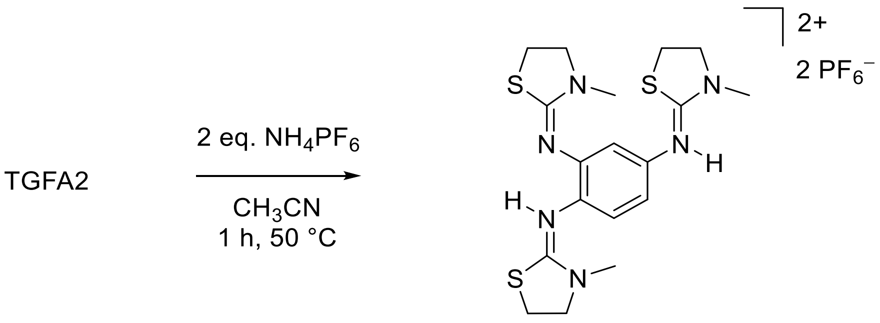

A mixture of 10.8 mg (25.7 µmol) of TGFA2 and 8.4 mg (51.5 µmol) of NH_4PF_6 in 3 ml CH_3CN was heated to 50 °C for 1h. The solvent was removed under reduced pressure and the solid residue washed 3 times with 3 ml Et_2O to give 13.1 mg (18.4 µmol, 72%) of a beige powder.

Elemental analysis calcd. (%) for $C_{18}H_{26}N_6S_3P_2F_{12}$ (712.56 g mol^{-1}): C 30.34, H 3.68, N 11.79; found C 30.39, H 4.40, N 12.60.

^{1}H NMR (399.89 MHz, CD_3CN, 295 K): δ = 7.24 (dd, J = 7.8, 1.1 Hz, 1 H, CH_{arom}), 6.90 – 6.87 (m, 2 H, CH_{arom}), 3.97 (t, J = 7.6 Hz, 2 H, CH_2), 3.88 (t, J = 7.5 Hz, 2 H, CH_2), 3.79 (t, J = 7.3 Hz, 2 H, CH_2), 3.41 – 3.29 (m, 6 H, CH_2), 3.15 (s, 3 H, CH_3), 3.10 (s, 3 H, CH_3), 3.08 (s, 3 H, CH_3) ppm.

^{13}**C NMR** (150.92 MHz, CD_3CN, 298 K): δ = 169.16, 167.67, 165.41 (C_q, C_{gua}), 143.24, 134.17 (C_q, C_{arom}), 125.64, 121.27, 118.74 (CH_{arom}), 56.94, 56.63, 55.21, 28.91, 28.74, 28.41 (CH_2), 35.12, 35.03, 34.60 (CH_3) ppm.

^{31}**P NMR** (161.88 MHz, CD_3CN, 295 K): δ = –144.64 (sept) ppm.

^{19}**F NMR** (376.27 MHz, CD_3CN, 295 K): δ = –71.98, –73.86 ppm.

HR-MS (HR-ESI$^+$, CH_3CN): *m/z* (%) = calcd. for $[C_{18}H_{25}N_6S_3]^+$ 421.1297, found 421.1312 (4); calcd. for $[C_{18}H_{26}N_6S_3]^{2+}$ 211.0685, found 211.0694 (100).

UV-vis (CH_3CN, c = $7.02\cdot10^{-5}$ M): λ (ε in l mol^{-1} cm^{-1}) = 216 (25870), 240 (sh, 22180), 266 (21210) nm.

Analytical data for (TGFA2+2H)(PF_6)$_2$

^{1}H NMR spectrum (399.89 MHz, 295 K) of (TGFA2+2H)(PF_6)$_2$ in CD_3CN

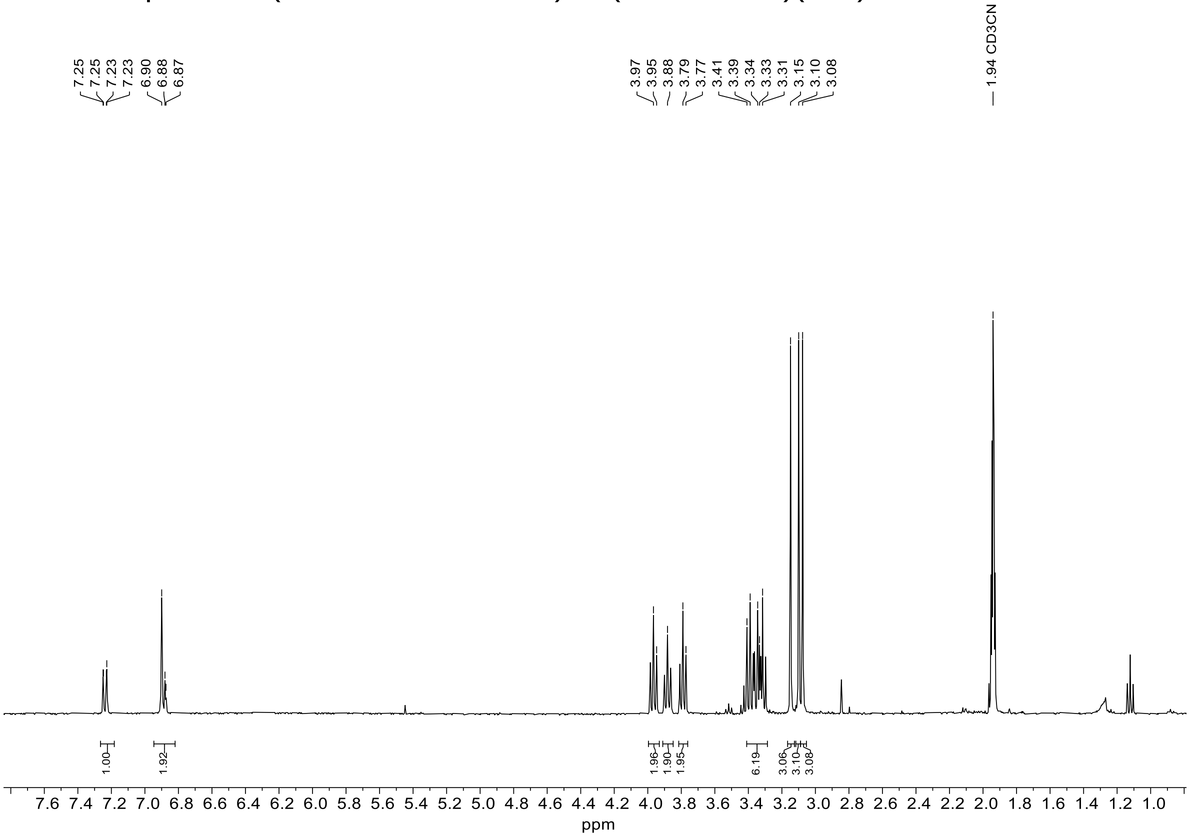

^{13}C NMR spectrum (150.92 MHz, 298 K) of (TGFA2+2H)$(PF_6)_2$ in CD_3CN

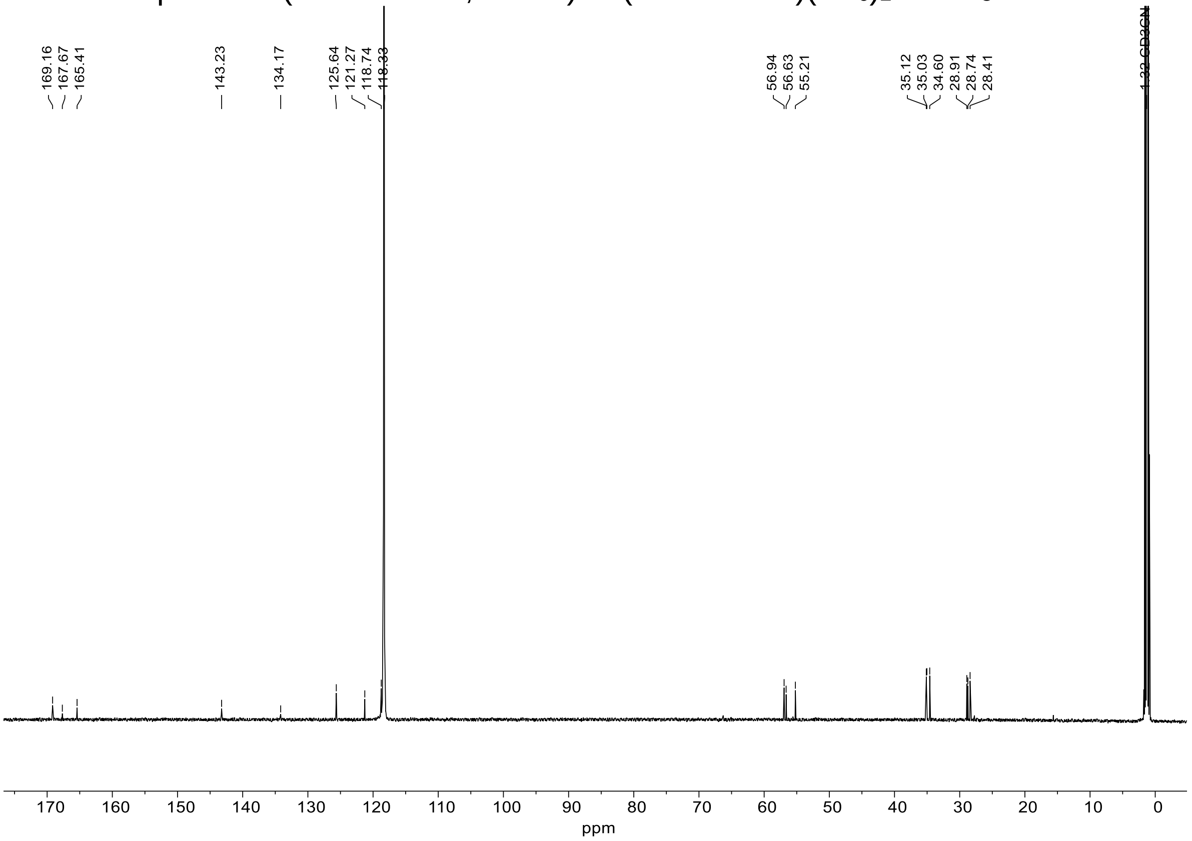

UV-vis spectrum of (TGFA2+2H)$(PF_6)_2$ in CH_3CN

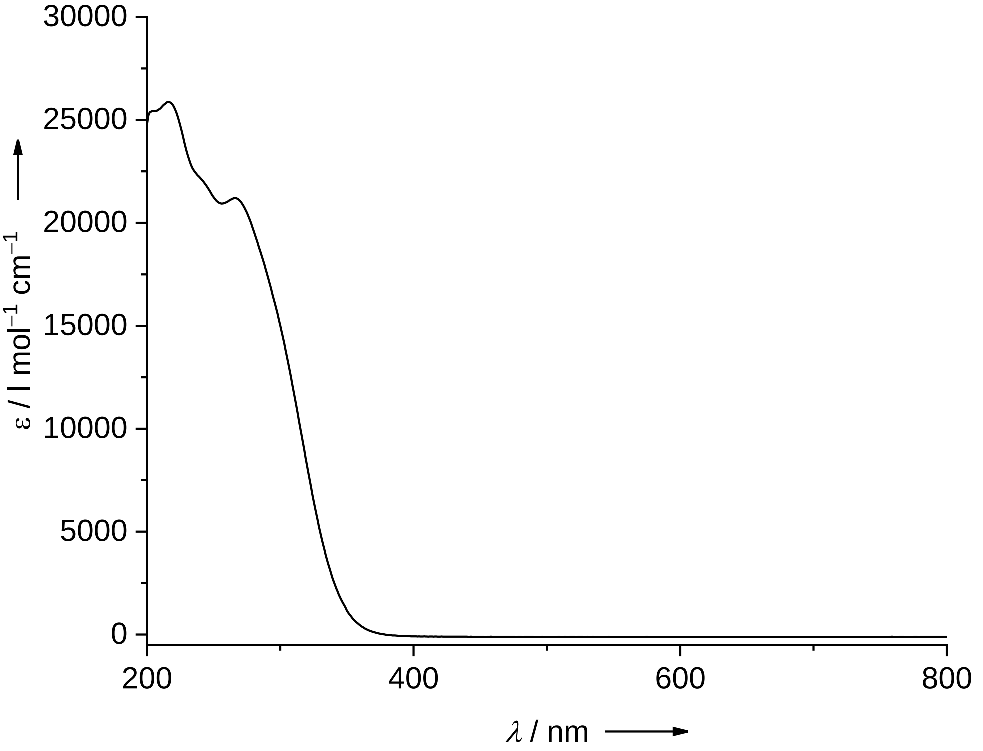

Attempt to oxidise TGFA2 with $AgPF_6$ leading to the coordination polymer $\{[AgCH_3CN(TGFA2)](PF_6)\}_n$

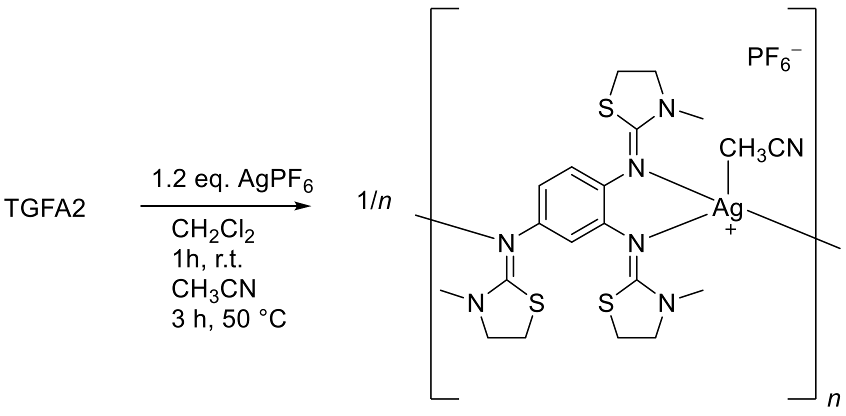

A mixture of 15.8 mg (37.6 µmol) of TGFA2 and 11.4 mg (45.1 µmol) of $AgPF_6$ was dissolved in 2 ml CH_2Cl_2 and stirred for 1h. The solvent was removed under reduced pressure. The solid residue was redissolved in 3 ml CH_3CN and the solution was heated to 50 °C for 3 h. Then, the mixture was cooled to r. t. and filtrated through a syringe filter. The solvent was removed and the colourless residue washed three times with 2 ml Et_2O (22.3 mg, 31.2 µmol, 83%). Colourless crystals suitable for a structural characterization with SC-XRD formed after layering a CH_3CN solution with Et_2O at 3 °C.

^{1}H NMR (399.89 MHz, CD_3CN, 295 K): δ = 6.88 (dd, *J* = 8.1, 1.1 Hz, 1 H, CH_{arom}), 6.56 (ddd, *J* = 8.3, 2.4, 1.1 Hz, 1 H, CH_{arom}), 6.49 (dd, *J* = 2.4, 1.1 Hz, 1 H, CH_{arom}), 3.71 (td, *J* = 7.2, 3.0 Hz, 4 H, CH_2), 3.63 – 3.55 (m, 2 H, CH_2), 3.23 – 3.14 (m, 6 H, CH_2), 3.06 (s, 3 H, CH_3), 3.05 (s, 3 H, CH_3), 2.97 (s, 3 H, CH_3) ppm.

^{13}C NMR (100.56 MHz, CD_3CN, 295 K): δ = 167.08, 161.11 (C_q, C_{gua}), 149.67, 145.11 (C_q, C_{arom}), 124.18, 118.85, 117.20 (CH_{arom}), 55.23, 55.14, 54.01, 27.68, 27.53, 27.51 (CH_2), 34.38, 34.34, 34.08 (CH_3) ppm.

^{31}P NMR (161.88 MHz, CD_3CN, 295 K): δ = –144.64 (sept) ppm.

^{19}F NMR (376.27 MHz, CD_3CN, 295 K): δ = –71.98, –73.86 ppm.

HR-MS (HR-ESI$^+$, CH_3CN): *m/z* (%) = calcd. for $[C_{18}H_{24}N_6S_3Ag]^+$ 527.0270, found 527.0283 (0.5); calcd. for $[C_{18}H_{24}N_6S_3]^{2+}$ 211.0685, found 211.0690 (100); calcd. for $[C_{18}H_{25}N_6S_3]^+$ 421.1297, found 421.1308 (28).

Analytical data for {[AgCH$_3$CN(TGFA2)](PF$_6$)}$_n$

^{1}H NMR spectrum (399.89 MHz, 295 K) of {[AgCH$_3$CN(TGFA2)](PF$_6$)}$_n$ in CD$_3$CN

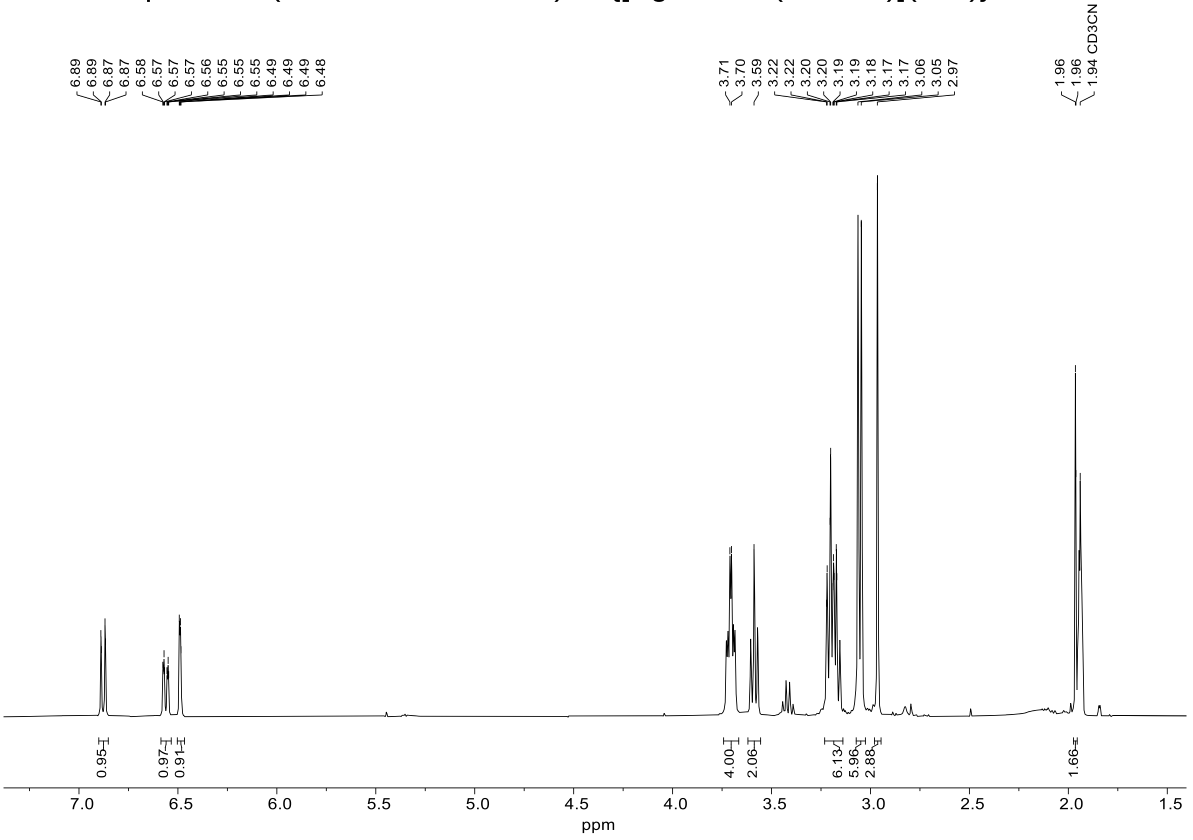

^{13}C NMR spectrum (150.91 MHz, 295 K) of {[AgCH$_3$CN(TGFA2)](PF$_6$)}$_n$ in CD$_3$CN

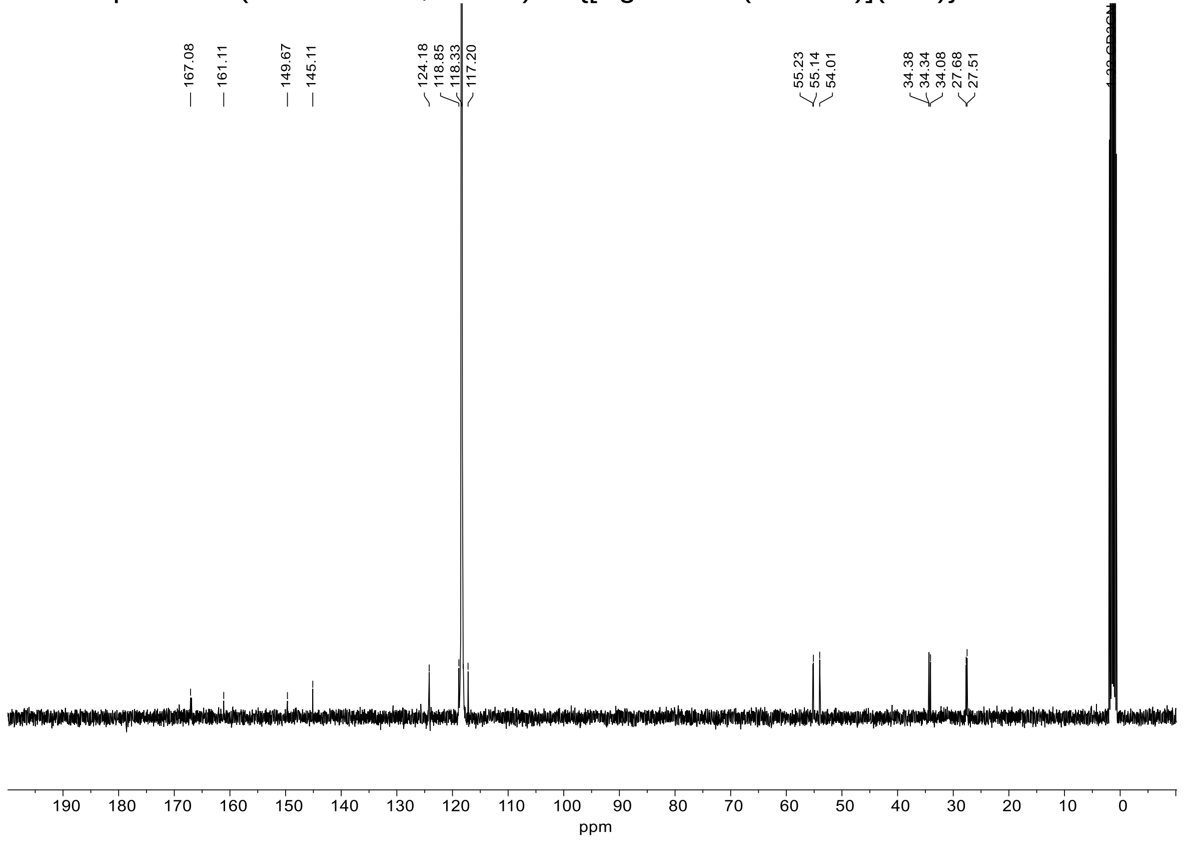

UV-vis spectrum of {[AgCH$_3$CN(TGFA2)](PF$_6$)}$_n$ in CH$_3$CN

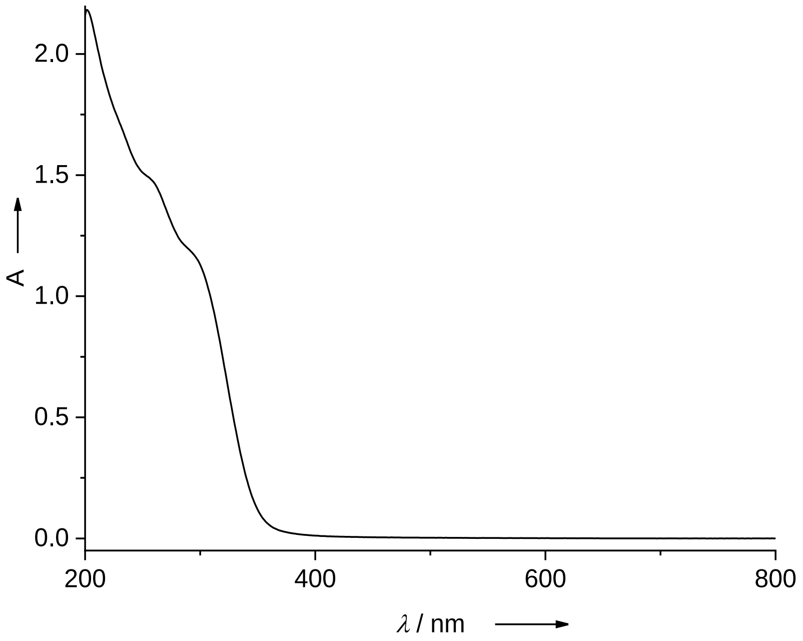

Synthesis of TGFA3

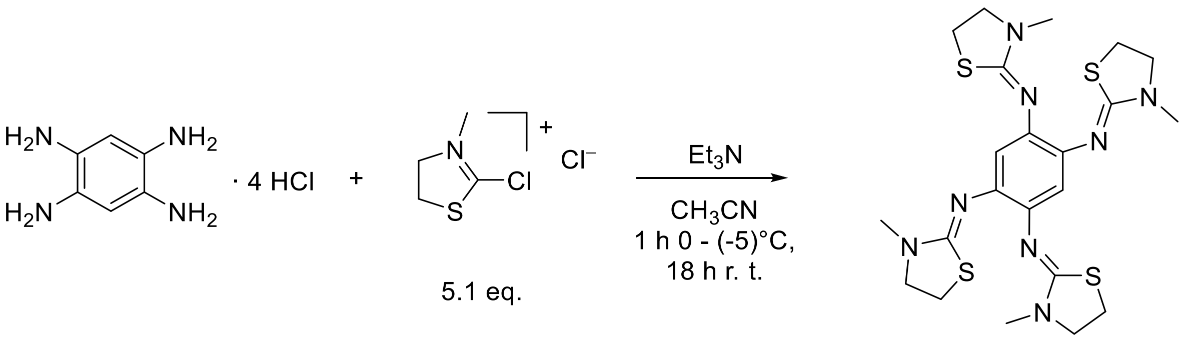

1,2,4,5-Tetraaminobenzene·4 HCl (143 mg, 0.5 mmol) was suspended in 10 ml CH$_3$CN and mixed with 1 ml Et$_3$N (7.2 mmol). The solution was cooled to 0 - (-5) °C (ice/ethanol). Then, to this solution 437.2 mg (2.54 mmol) of 2-chloro-3-methyl-2-thiazolidinium chloride suspended in 10 ml CH$_3$CN was added dropwise and the mixture was stirred for 18 h (during which time the ice bath slowly thaws). The solvent was removed under vacuum and the residue was re-dissolved with some H$_2$O and HCl (10%, pH < 3). The aqueous phase was extracted twice with 15 ml of CH$_2$Cl$_2$ and the collected organic phases were extracted once with brine. Then, the pH value of the collected aqueous phases was adjusted to > 10 with NaOH (25%). After repeated extraction with a total of 75 ml of CH$_2$Cl$_2$, accompanied with re-adjustment of the pH value, the organic phase was dried over K$_2$CO$_3$ and filtrated. Then, the solvent was removed, yielding 298.4 mg of a beige-brown residue. The residue was washed with a small amount of CH$_3$CN and Et$_2$O (245.8 mg, 0.44 mmol, 88%). Crystals suitable for SC-XRD were grown from a CH$_2$Cl$_2$ solution layered with *n*-hexane at 3 °C.

Elemental analysis calcd. (%) for $C_{22}H_{30}N_8S_4 \cdot 0.2\ CH_2Cl_2$ (556.01 g mol^{-1}): C 48.06, H 5.53, N 20.15; found C 48.52, H 5.36, N 20.59.

^{1}H NMR (600.18 MHz, CD_2Cl_2, 295 K): δ = 6.29 (s, 2 H, CH_{arom}), 3.48 (t, J = 6.8 Hz, 8 H, CH_2), 3.10 (t, J = 6.8 Hz, 8 H, CH_2), 2.95 (s, 12 H, CH_3 ppm).

^{13}C NMR (150.92 MHz, CD_2Cl_2, 295 K): δ = 158.72 (C_q, C_{gua}), 140.41 (C_q, C_{arom}), 114.85 (CH_{arom}), 53.50, 27.36 (CH_2), 34.19 (CH_3) ppm.

HR-MS (HR-ESI$^+$, CH_2Cl_2): m/z (%) = calcd. for $[C_{22}H_{31}N_8S_4]^+$ 535.1549, found 535.1548 (100).

UV-vis (CH_2Cl_2, c = 8.35·10^{-5} M): λ (ε in l mol^{-1} cm^{-1}) = 261 (23340), 308 (14750) nm.

Analytical data for TGFA3

^{1}H NMR spectrum (600.18 MHz, 295 K) of TGFA3 in CD_2Cl_2

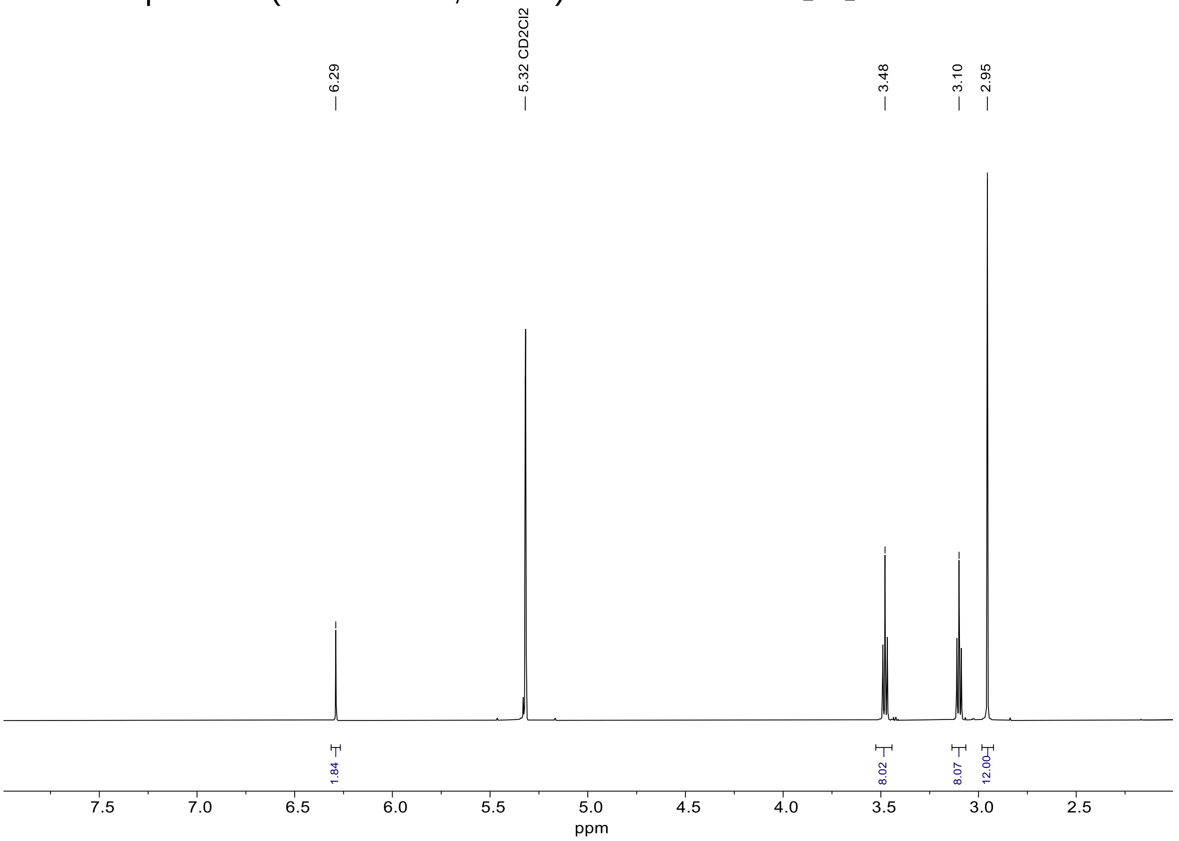

^{13}C NMR spectrum (150.92 MHz, 295 K) of TGFA3 in CD_2Cl_2

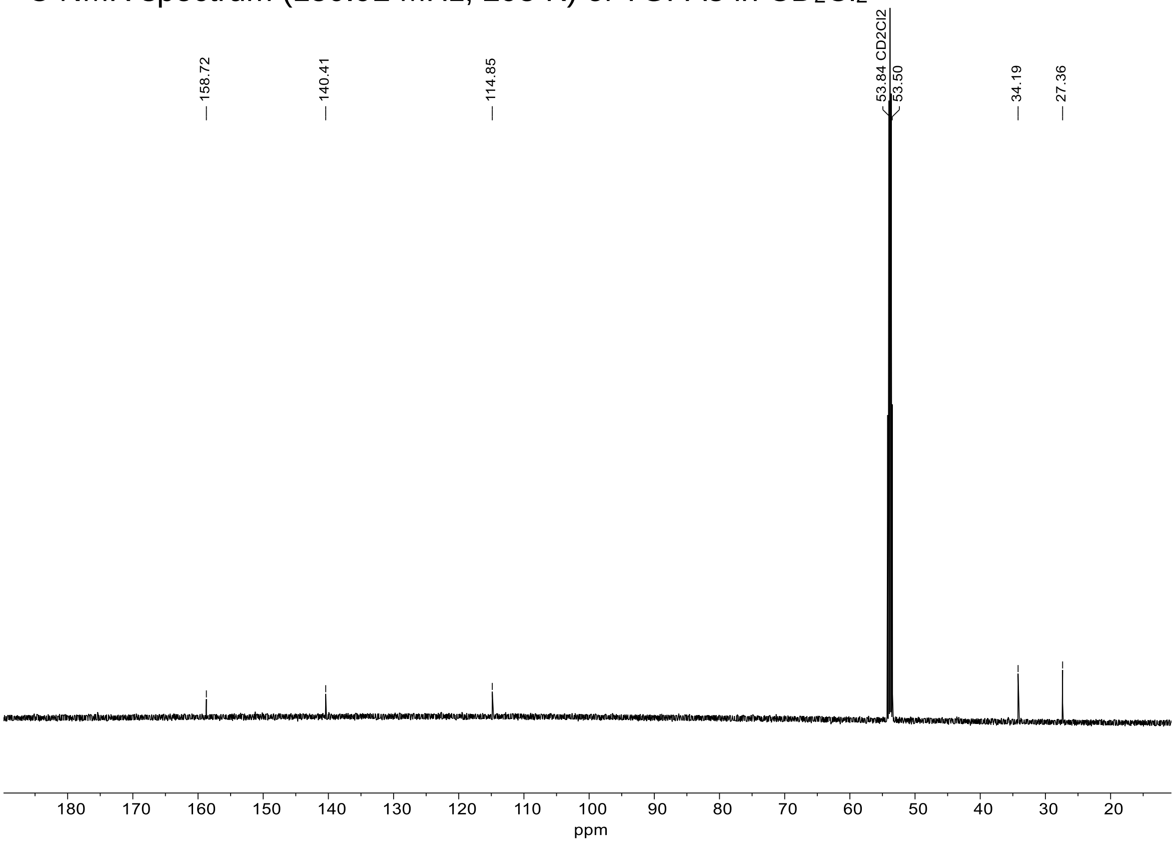

UV-vis spectrum of TGFA3 in CH_2Cl_2

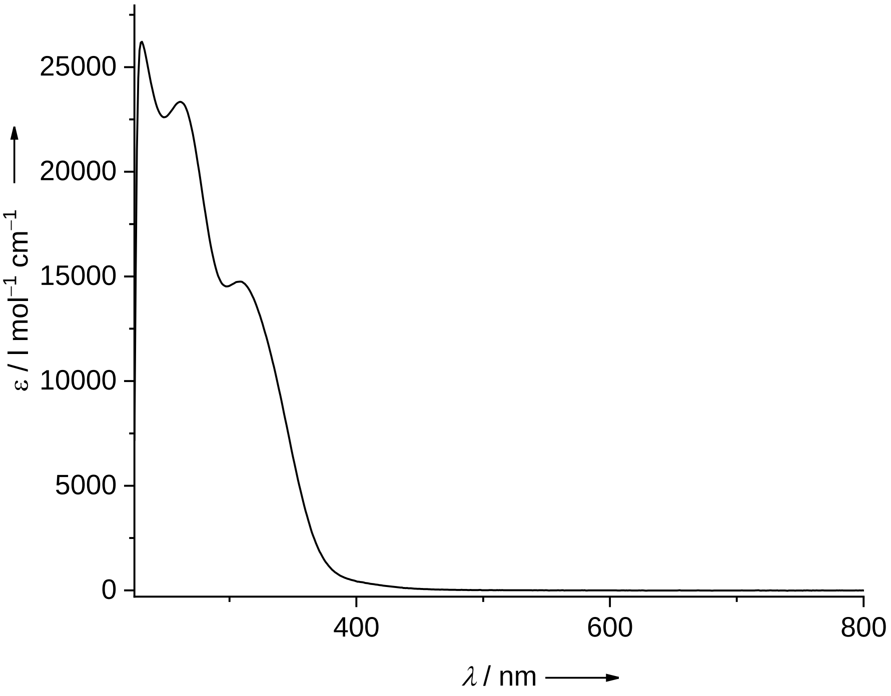

Cyclic voltammetry curves for TGFA3 in CH_2Cl_2 ($^nBu_4NPF_6$ as supporting electrolyte). Potentials given vs. the ferrocenium/ferrocene (Fc^+/Fc) redox couple. For comparison, the cyclic voltammetry curve of GFA3b is also included.

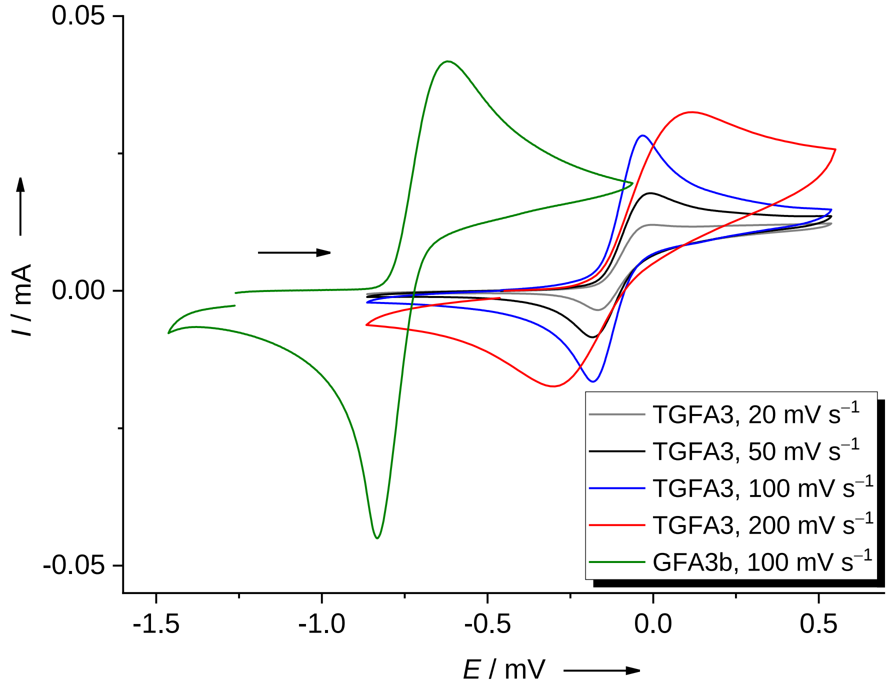

Cyclic voltammetry curves for TGFA3 in CH_3CN ($^nBu_4NPF_6$ as supporting electrolyte). Potentials given vs. the ferrocenium/ferrocene (Fc^+/Fc) redox couple. The measurements were hampered by the low solubility of TGFA3 in CH_3CN. For comparison, the cyclic voltammetry curve of GFA3b is also included.

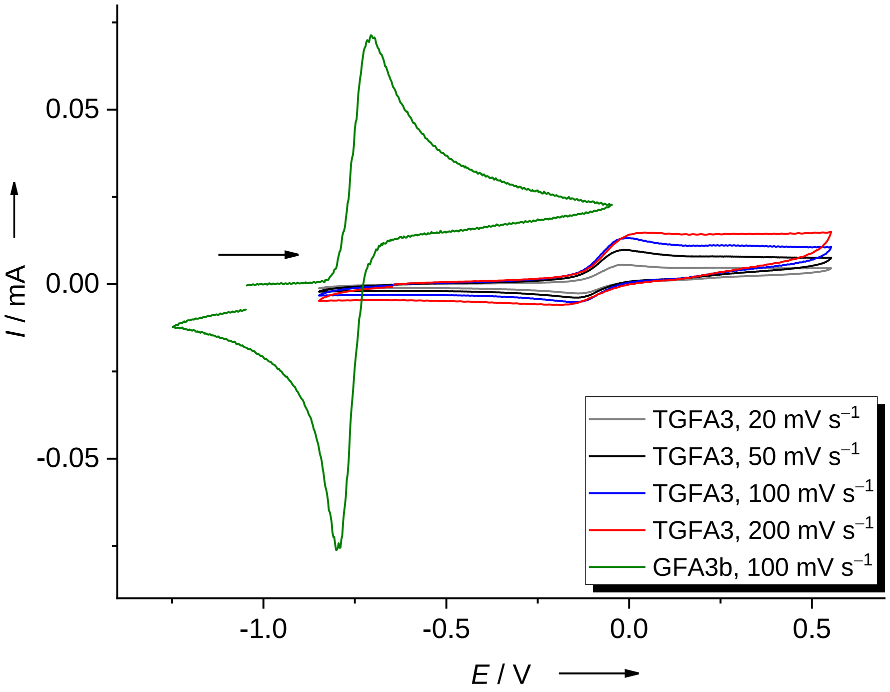

Protonation of TGFA3 to give (TGFA3+2H)(PF_6)$_2$

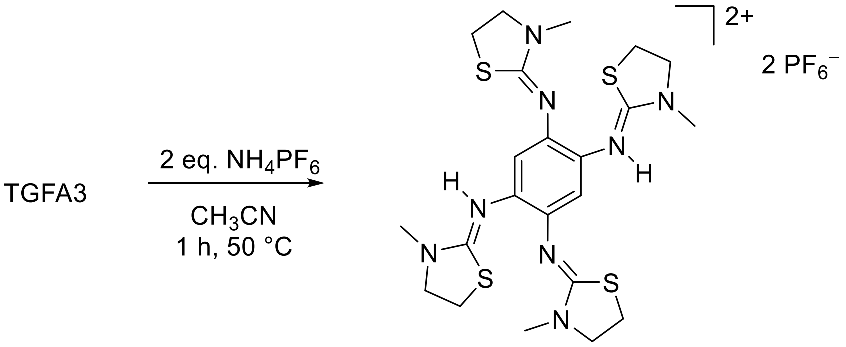

A mixture of 24.6 mg of TGFA3 (46 µmol) and 15 mg of NH_4PF_6 (92 µmol, 2 eq) was dissolved in 10 ml CH_3CN, and heated to 50 °C for 1 h. The solvent was removed under reduced pressure and re-dissolved in 5 ml CH_3CN. Then, 40 mg of activated charcoal were added to the solution and stirred for 30 min. After filtration using a syringe filter and solvent removal, 25.9 mg (31 µmol, 67%) of a beige powder was obtained. Crystals suitable for SC-XRD were grown from a CH_3CN solution layered with Et_2O at 3 °C.

Elemental analysis calcd. (%) for $C_{22}H_{32}N_8S_4P_2F_{12}\cdot H_2O$ (844.74 g mol^{-1}): C 31.28, H 4.06, N 13.27; found C 31.10, H 4.81, N 12.05.

^{1}H NMR (399.99 MHz, CD_3CN, 295 K): δ = 7.04 (s, 2 H, CH_{arom}), 3.90 (t, *J* = 7.5 Hz, 8 H, CH_2), 3.37 (t, *J* = 7.5 Hz, 8 H, CH_2), 3.12 (s, 12 H, CH_3) ppm.

^{13}C NMR (150.91 MHz, CD_3CN, 298 K): δ = 168.15 (C_q, C_{gua}), 137.44 (C_q, C_{arom}), 119.20 (CH_{arom}), 56.78, 29.34 (CH_2), 35.49 (CH_3) ppm.

^{19}F NMR (376.27 MHz, CD_3CN, 295 K): δ = –71.91, –73.79 ppm.

^{31}P NMR (242.95 MHz, CD_3CN, 298 K): δ = –144.61 (sept) ppm.

HR-MS (HR-ESI$^+$, CH_3CN): *m/z* (%) = calcd. for [$C_{22}H_{31}N_8S_4$]$^+$ 535.1549, found 535.1550 (100); calcd. for [$C_{44}H_{62}N_{16}S_8PF_6$]$^+$ 1215.2745, found 1215.2768 (9).

UV-vis (CH_3CN, c = 4.93·10^{-5} M): λ (ε in l mol^{-1} cm^{-1}) = 218 (30310), 243 (sh, 23490), 283 (24550), 316 (sh, 17540) nm.

Analytical data for (TGFA3+2H)$(PF_6)_2$

^{1}H NMR spectrum (600.18 MHz, 295 K) of (TGFA3+2H)$(PF_6)_2$ in CD_3CN

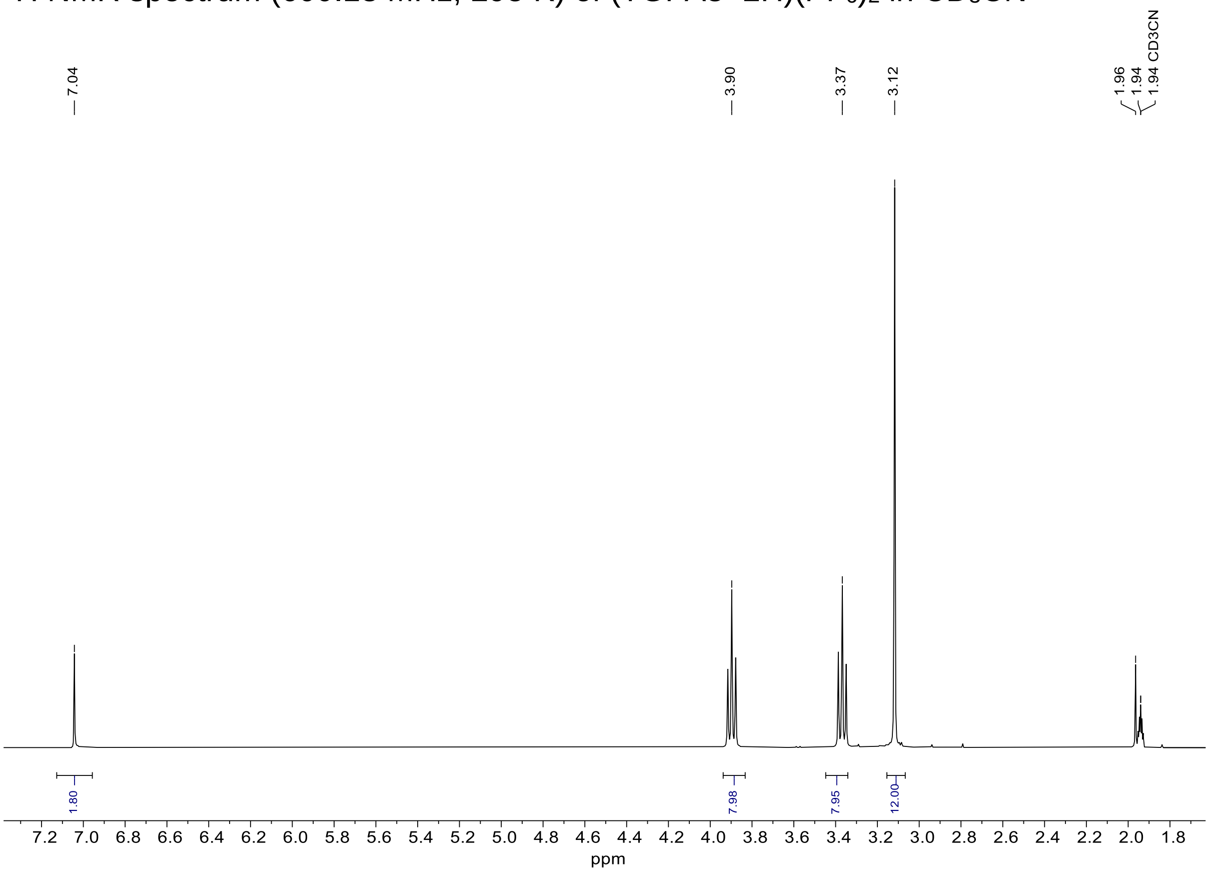

^{13}C NMR spectrum (150.91 MHz, 298 K) of (TGFA3+2H)$(PF_6)_2$ in CD_3CN

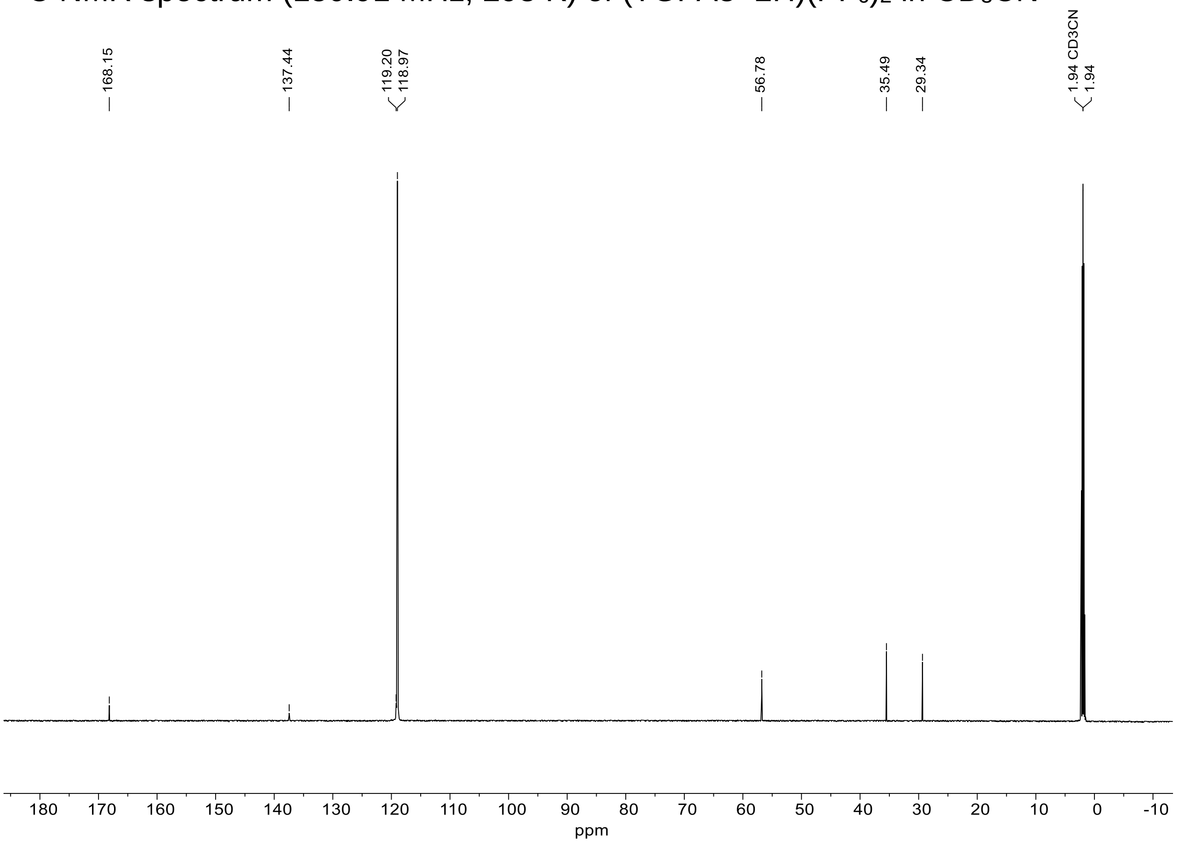

UV-vis spectrum of $(TGFA3+2H)(PF_6)_2$ in CH_3CN

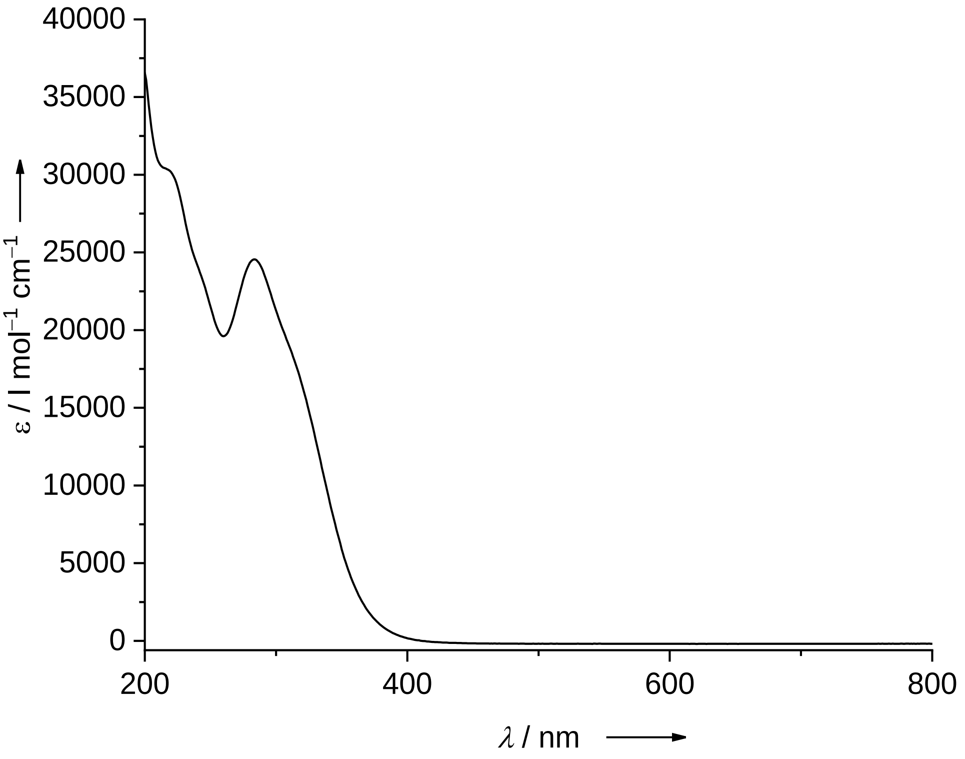

Oxidation of TGFA3 with ferrocenium hexafluorophosphate to give $TGFA3(PF_6)_2$

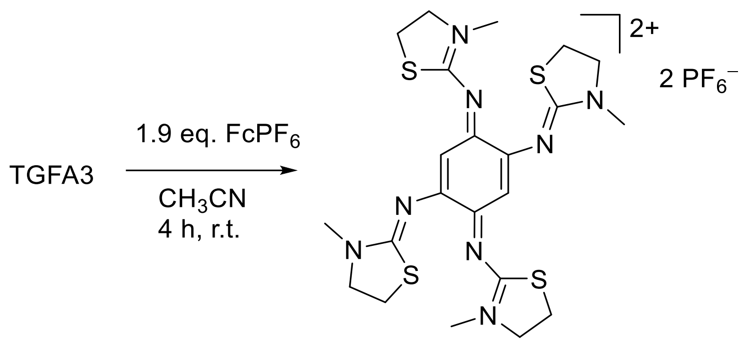

A mixture of 42.2 mg (79 µmol) TGFA3 and 50.0 mg (151 µmol) of ferrocenium hexafluorophosphate was dried at 40 °C under vacuum for 2 h and then dissolved in 10 ml CH_3CN. The reaction mixture was stirred for a period of 4 h at room temperature. Then, the solvent was removed under vacuum, the brown residue washed three times with 10 ml Et_2O (until colourless) and dried. The brown powder was re-dissolved in CH_3CN and layered with Et_2O resulting 51 mg (62 µmol, 80%) of a brown powder. Crystals suitable for SC-XRD were obtained at 3 °C by dissolving the residue in small amounts of CH_3CN and layering this solution with Et_2O.

Elemental analysis calcd. (%) for $C_{22}H_{30}N_8S_4P_2F_{12}$ (824.71 g mol^{-1}): C 32.04, H 3.67, N 13.59; found C 32.07, H 4.48, N 13.76.

^{1}H NMR (600.18 MHz, CD_3CN, 295 K): δ = 6.12 (s, 2 H, CH_{ring}), 4.04 (t, *J* = 7.7 Hz, 8 H, CH_2), 3.53 (t, *J* = 7.9 Hz, 8 H, CH_2), 3.07 (s, 12 H, CH_3) ppm.

^{13}C NMR (150.92 MHz, CD_3CN, 295 K): δ = 173.35 (C_q, C_{gua}), 157.50 (C_q, C_{ring}), 111.48 (CH_{ring}), 56.26, 29.07 (CH_2), 35.42 (CH_3) ppm.

^{31}P NMR (242.96 MHz, CD_3CN, 295 K): δ = –144.63 (sept) ppm.

^{19}F NMR (564.73 MHz, CD_3CN, 295 K): δ = –73.51, –72.26 ppm.

HR-MS (HR-ESI$^+$, CH_2Cl_2): *m/z* (%) = calcd. for $[C_{22}H_{30}N_8S_4]^+$ 534.1471, found 534.1471 (100); calcd. for $[C_{22}H_{30}N_8S_4PF_6]^+$ 679.1113, found 679.1119 (17).

UV-vis (CH_3CN, c = $3.66{\cdot}10^{-5}$ M): λ (ε in l mol^{-1} cm^{-1}) = 226 (32460), 252 (29585), 293 (21460), 455 (17435), 632 (700) nm.

Analytical data for TGFA3(PF_6)$_2$

^{1}H NMR spectrum (600.18 MHz, 295 K) of TGFA3(PF_6)$_2$ in CD_3CN

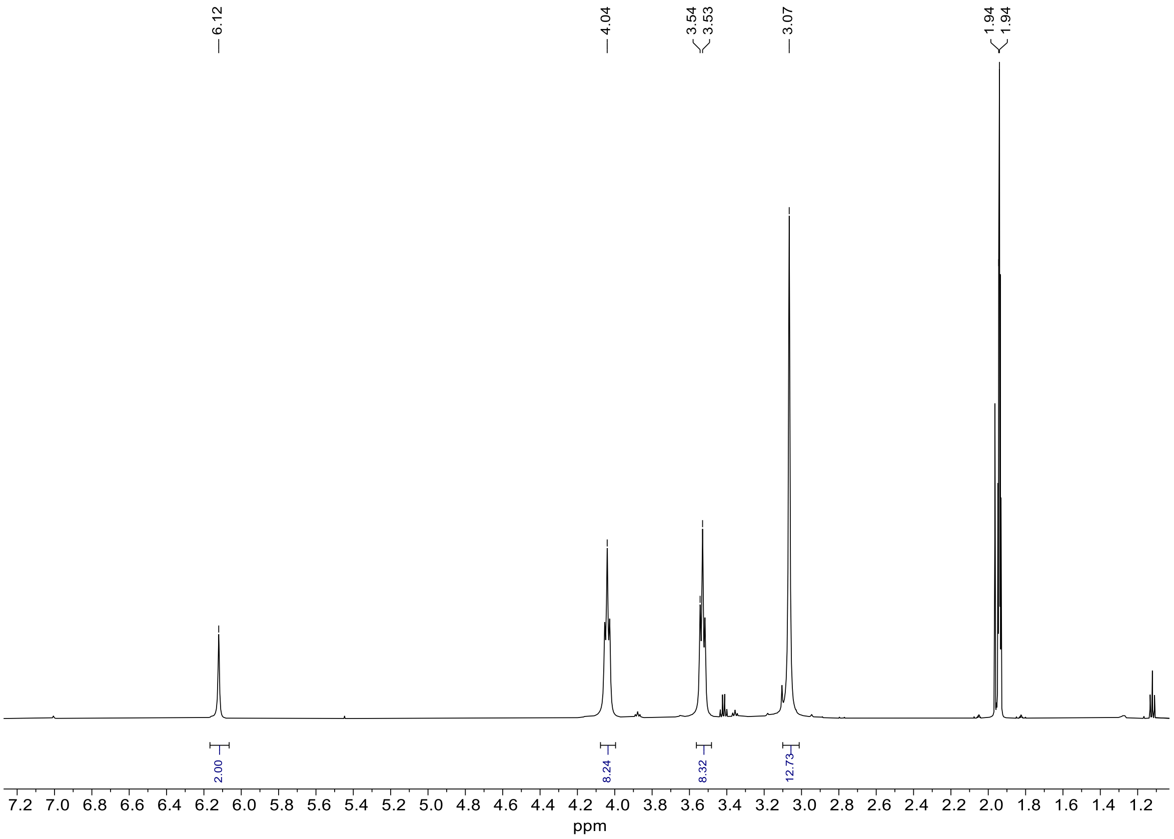

^{13}C NMR spectrum (150.92 MHz, 295 K) of TGFA3$(PF_6)_2$ in CD_3CN

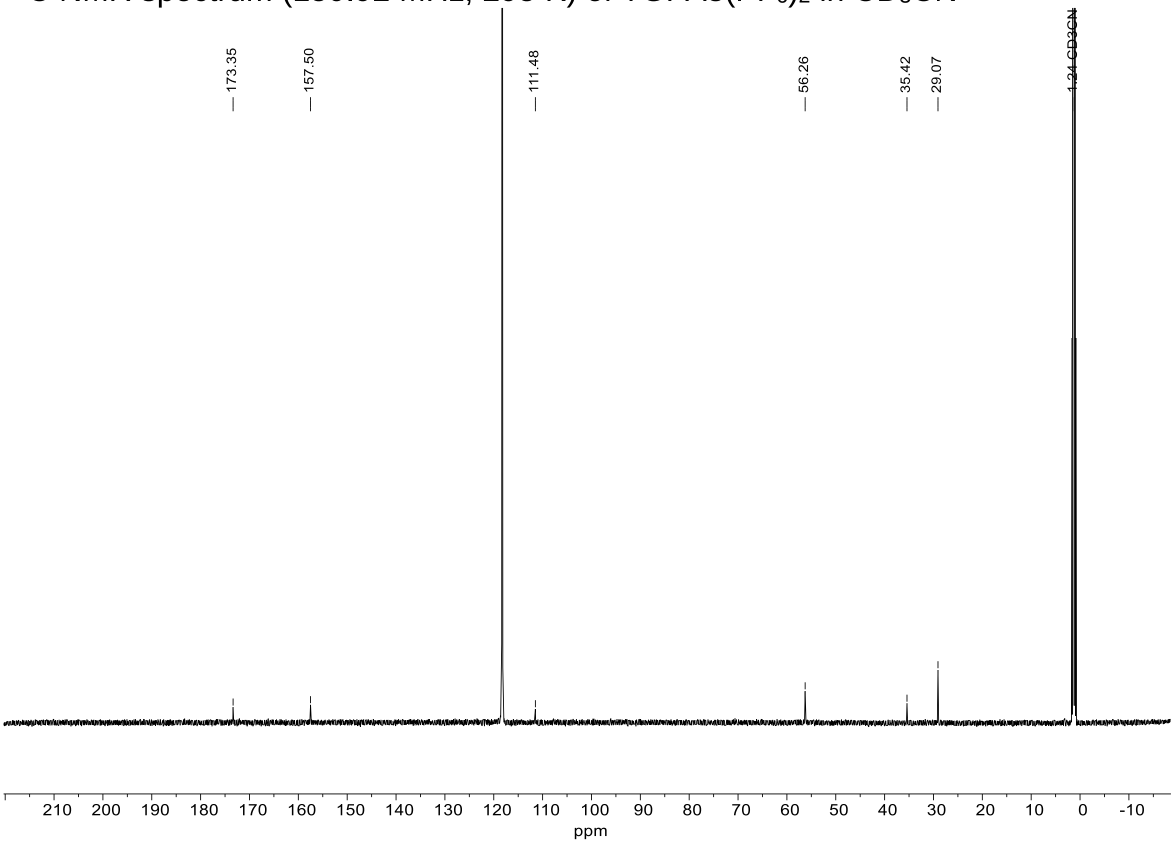

UV-vis spectrum of TGFA3$(PF_6)_2$ in CH_3CN

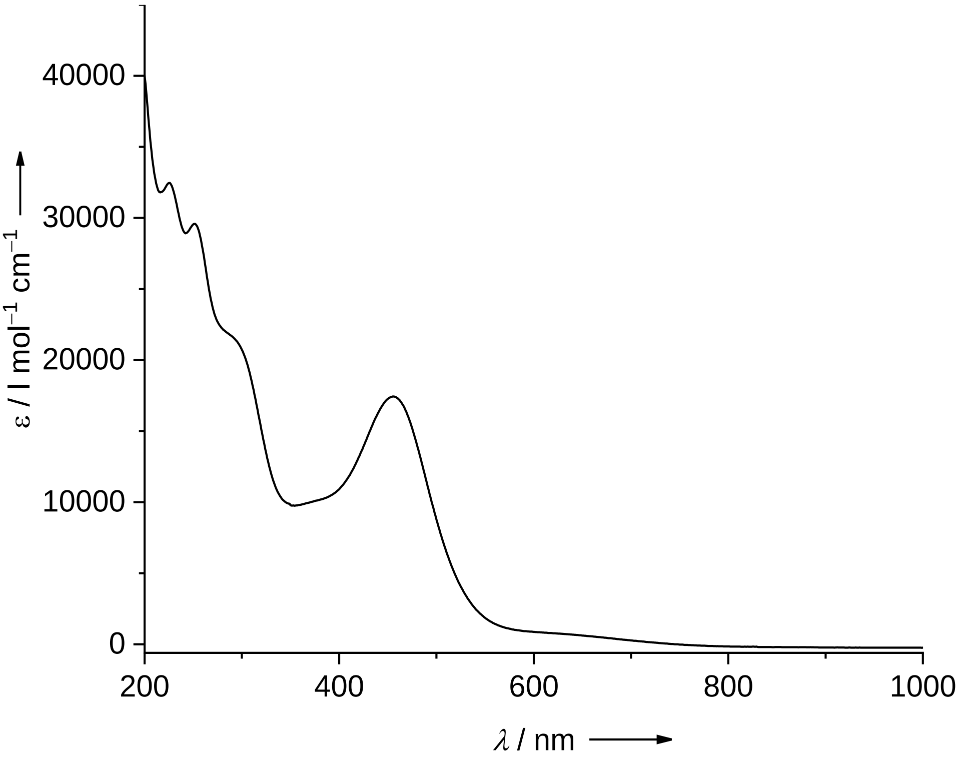

Oxidation of TGFA3 with acetylferrocenium tetrafluoroborate to give TGFA3(BF_4)$_2$

TGFA3

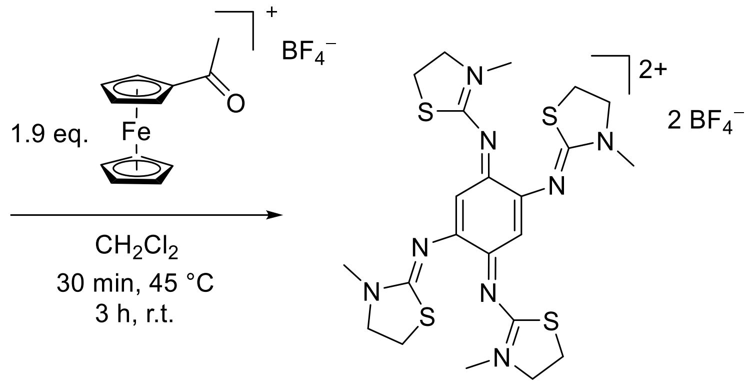

A mixture of 33.5 mg (62.6 µmol) TGFA3 and 37.7 mg (119.7 µmol, 1.9 eq.) of acetylferrocenium tetrafluoroborate was dried at 40 °C under vacuum for 2 h and then dissolved in 5 ml CH_2Cl_2. The reaction mixture was stirred for 30 min at 45 °C and 3.5 h at room temperature. The solvent was removed under vacuum, the brown residue was washed three times with 10 ml Et_2O (until colourless) and dried. The brown powder was re-dissolved in 2 ml CH_3CN and 5.4 mg (16.8 µmol) diacetyliodobenzene was added (to oxidise protonated TGFA3) and stirred 20 min at 45 °C. The solvent was removed under reduced pressure and the residue washed twice with 5 ml toluene and with 10 ml Et_2O. The obtained residue was dissolved in CH_3CN and filtrated via syringe filter. The solvent was partially removed and layered with Et_2O resulting in 25 mg (32 µmol, 54%) product as a brown powder.

Elemental analysis calcd. (%) for $C_{22}H_{30}N_8S_4B_2F_8{\cdot}CH_2Cl_2$ (793.13 g mol^{-1}): C 34.82, H 4.07, N 14.13; found C 35.28, H 4.60, N 13.45.

^{1}H NMR (600.18 MHz, CD_3CN, 295 K): δ = 6.12 (s, 2 H, CH_{ring}), 4.04 (t, J = 8.0 Hz, 8 H, CH_2), 3.53 (t, J = 8.0 Hz, 8 H, CH_2), 3.06 (s, 12 H, CH_3) ppm.

^{13}C NMR (150.92 MHz, CD_3CN, 295 K): δ = 173.35 (C_q, C_{gua}), 157.49 (C_q, C_{ring}), 111.52 (CH_{ring}), 56.28, 29.09 (CH_2), 35.44 (CH_3) ppm.

^{11}B NMR (192.56 MHz, CD_3CN, 295 K): δ = –1.19 ppm.

^{19}F NMR (564.73 MHz, CD_3CN, 295 K): δ = –151.81 ppm.

HR-MS (MALDI$^+$, DCBT): *m/z* (%) = calcd. for $[C_{22}H_{30}N_8S_4]^+$ 534.1471, found 534.1484 (100); calcd. for $[C_{22}H_{30}N_8S_4BF_4]^+$ 621.1500, found 621.1524 (73).

UV-vis (CH_3CN, c = 7.82·10^{-5} M): λ (ε in l mol^{-1} cm^{-1}) = 227 (28160), 250 (27420), 290 (22820), 455 (13075), 632 (930) nm.

Analytical data for TGFA3(BF_4)$_2$

^1H NMR spectrum (600.18 MHz, 295 K) of TGFA3(BF_4)$_2$ in CD_3CN

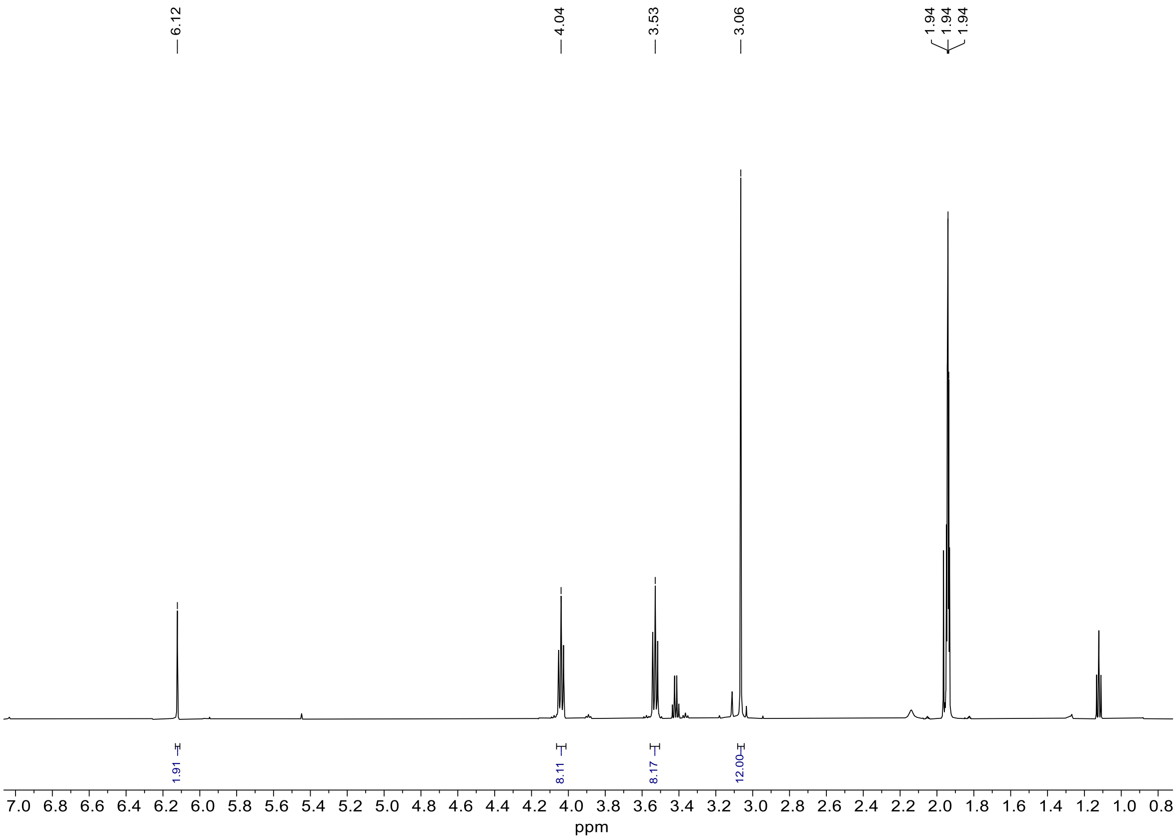

^{13}C NMR spectrum (150.92 MHz, 295 K) of TGFA3(BF_4)$_2$ in CD_3CN

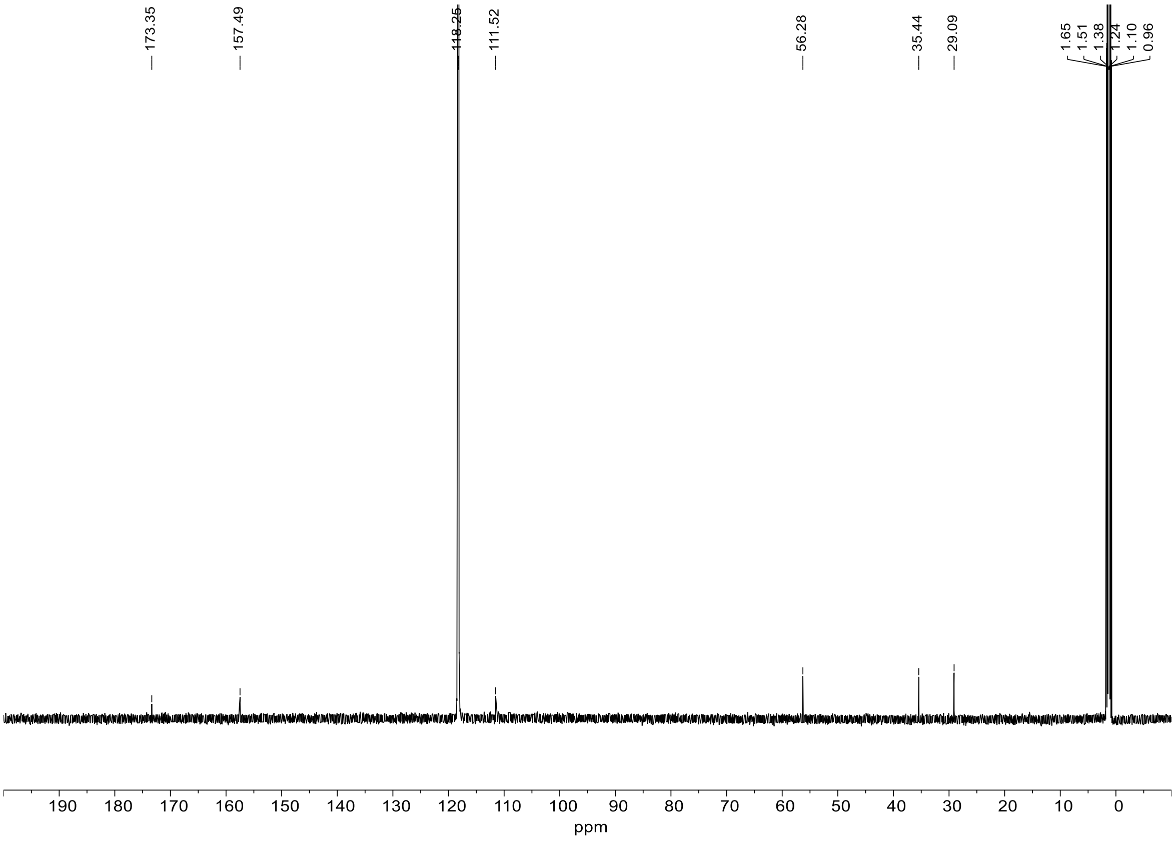

UV-vis spectrum of $TGFA3(BF_4)_2$ in CH_3CN

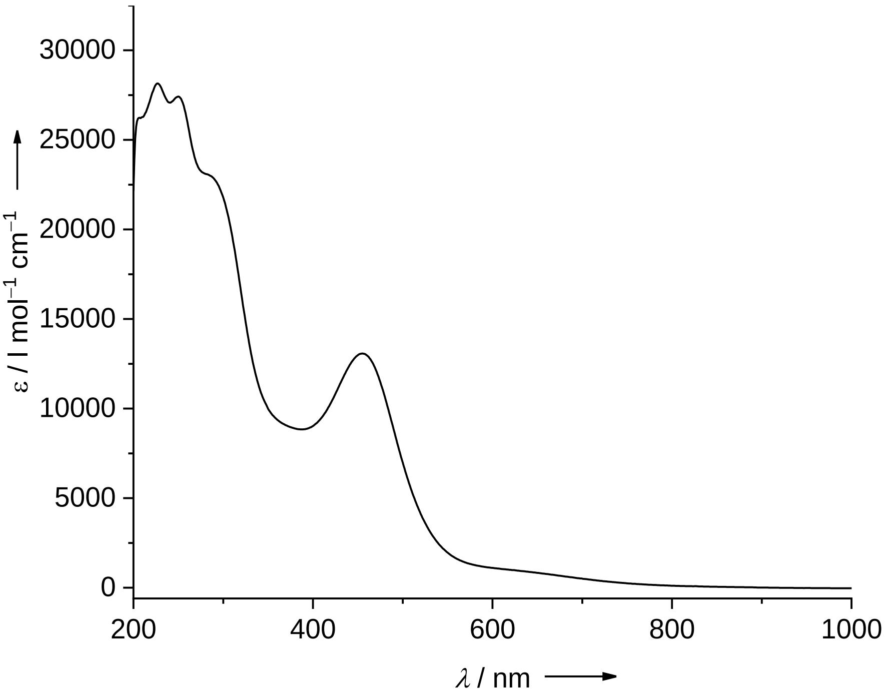

Protonation experiments of $(TGFA3)^{2+}$ with $HBF_4 \cdot Et_2O$

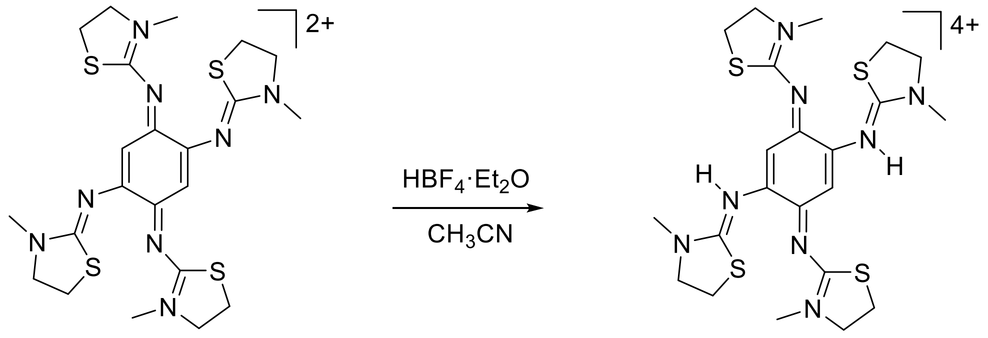

Titration of $TGFA3(PF_6)_2$ with $HBF_4 \cdot Et_2O$: NMR experiment

In an NMR tube, 4.122 mg (5 µmol) of $TGFA3(PF_6)_2$ was dissolved in 0.45 ml CD_3CN and titrated with a $HBF_4 \cdot Et_2O$ solution in CD_3CN (c = 0.36 M). NMR spectra were taken for stepwise addition of portions of ca. 0.5 eq. of the $HBF_4 \cdot Et_2O$ solution (7 µl, 2.5 µmol).

^1H NMR spectra (600.15 MHz, 298 K) for titration of TGFA3(PF_6)$_2$ with $HBF_4 \cdot Et_2O$ in CD_3CN

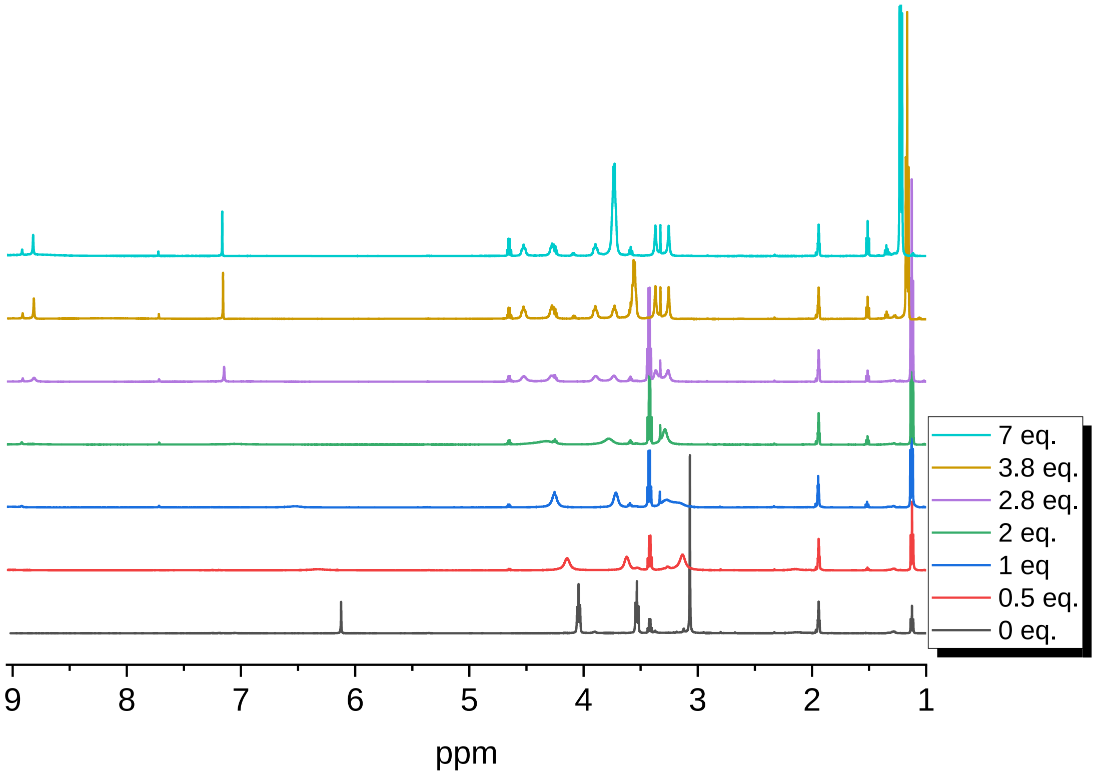

At the concentrations used in the NMR experiments, the doubly-protonated form $(TGFA3{+}2H)^{4+}$ is exclusively present after addition of 7 eq. of $HBF_4 \cdot Et_2O$. The spectra recorded at lower $HBF_4 \cdot Et_2O$ concentration show the monoprotonated and the diprotonated forms together, $(TGFA3{+}H)^{3+}$ and $(TGFA3{+}2H)^{4+}$. The NMR signals for $(TGFA3{+}H)^{3+}$ were deduced from the spectrum recorded after addition of 1 eq. of $HBF_4 \cdot Et_2O$.

^1H NMR data for $(TGFA3{+}H)^{3+}$ (after addition of 1 eq $HBF_4 \cdot Et_2O$ to $TGFA^{2+}$) (600.15 MHz, CD_3CN, 298 K): δ = 6.52 (s, 2 H, CH_{arom}), 4.25 (bs, 8 H, CH_2), 3.71 (bs, 8 H, CH_2), 3.36 – 3.07 (m, 12 H, CH_3) ppm.

Photos of the NMR tubes after addition of various amounts of $HBF_4 \cdot Et_2O$ to a solution of TGFA3(PF_6)$_2$ in CD_3CN

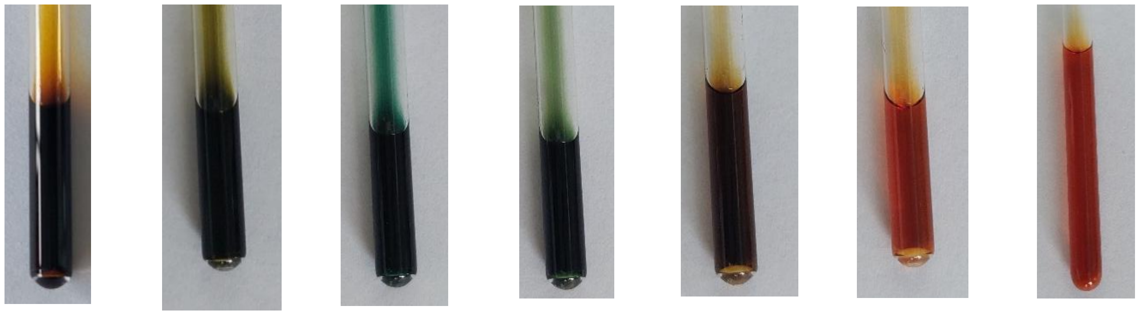

0 eq. | 0.5 eq. | 1 eq. | 2 eq. | 2.8 eq. | 3.8 eq. | 7 eq.

Titration of TGFA3(PF_6)$_2$ with HBF_4·Et_2O: UV-vis experiment

In a 1 cm cuvette, 2.4 ml of a solution of TGFA3(PF_6)$_2$ in CH_3CN ($c = 3.14 \cdot 10^{-4}$ M) was titrated with a HBF_4·Et_2O solution in CH_3CN ($c = 3.5 \cdot 10^{-2}$ M). 1 eq. of HBF_4·Et_2O corresponds to 9 µl ($3.12 \cdot 10^{-4}$ mmol).

UV-vis spectra (CH_3CN) of the titration of TGFA3(PF_6)$_2$ with HBF_4·Et_2O

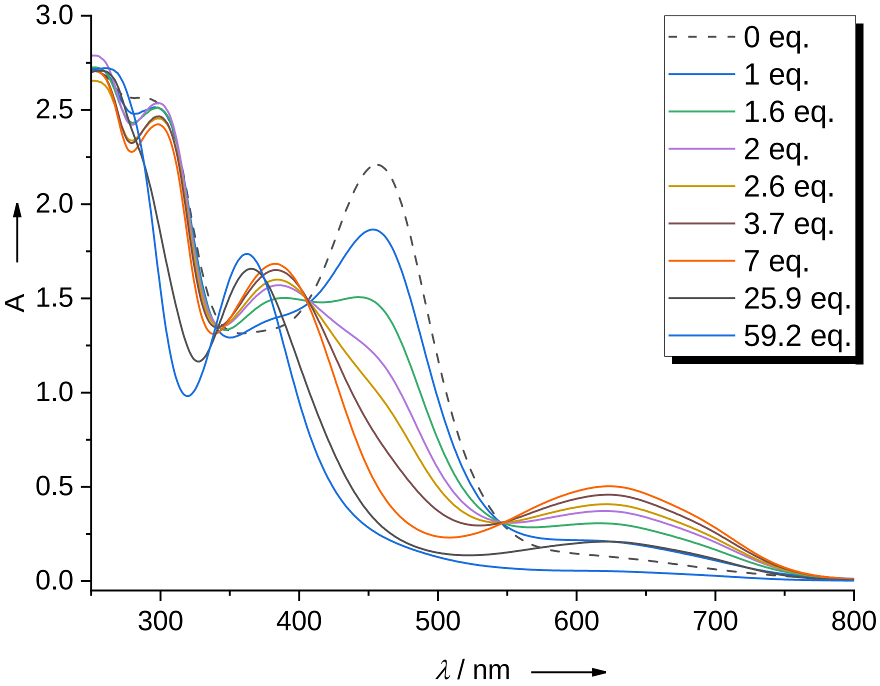

Due to the low concentration used in the UV-vis experiments, more equivalents of HBF_4·Et_2O are necessary for protonation of the dicationic oxidized molecule in the UV-vis experiments compared to the NMR experiments. The bands due to the monoprotonated form (TGFA3+H)$^{3+}$ are fully developed upon addition of 7 eq. of HBF_4·Et_2O.

UV-vis data of (TGFA3+H)$^{3+}$ (after addition of 7 eq. HBF_4·Et_2O to TGFA3^{2+}): λ = 298, 382, 624 nm.

Photos of the UV-vis cuvettes after addition of various amounts of HBF_4·Et_2O to a solution of TGFA3(PF_6)$_2$ in CH_3CN

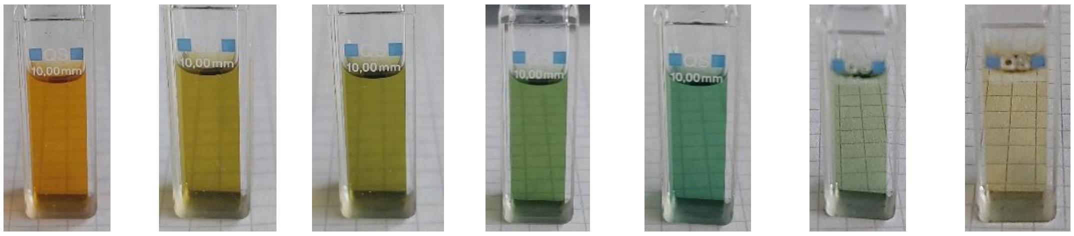

0 eq. 1.6 eq. 2 eq. 3.7 eq. 7 eq. 25.9 eq. 59.2 eq.

Preparation of (TGFA3+2H)(BF_4)$_4$

TGFA3(BF_4)$_2$ (19.8 mg, 28 µmol) was dried at 40 °C under vacuum for 2 h and dissolved in 5 ml CH_3CN. Then, 0.15 ml of a $HBF_4{\cdot}Et_2O$ solution in CH_3CN (10%, 0.73 M, 110 µmol, 4 eq.) was added (the colour of the reaction mixture changes from brown, then green to orange) and stirred for 5 min. The solvent was partially removed under vacuum and the remaining solution was layered with Et_2O to give few orange crystals suitable for a structural characterization with SC-XRD at 3 °C.
In solution, the diprotonated form (TGFA3+2H)$^{4+}$ is stable only in the presence of an excess of a strong acid ($HBF_4{\cdot}Et_2O$).
For comparison, (GFA3a+2H)$^{4+}$ is stable in solution (see U. Wild, O. Hübner, L. Greb, M. Enders, E. Kaifer, H.-J. Himmel, Twofold Oxidized and Twofold Protonated Redox-Active Guanidine: An Ultimate Intermediate in Proton-Coupled Electron-Transfer Reactions, *Eur. J. Org. Chem.* **2018**, 5910–5915).

For NMR measurements:

In an NMR tube, 5.64 mg (7.6 µmol) of TGFA3(BF_4)$_2$ were dried at 45 °C under vacuum and then dissolved in 0.45 ml CD_3CN. 40 µl of a $HBF_4{\cdot}Et_2O$ solution in CD_3CN (c = 0.73 M, 29.2 µmol, 3.8 eq.). The NMR tube was flame-sealed under vacuum.
A small amount of 2-fold protonated TGFA3 from the oxidation of TGFA3 with acetylferrocenium tetrafluoroborate reacts to (TGFA3+4H)(BF_4)$_4$.

^{1}H NMR (600.18 MHz, CD_3CN, 295 K): δ = 8.82 (s, 2 H, NH), 7.17 (s, 2 H, CH_{ring}), 4.51 (t, *J* = 8.5 Hz, 4 H, CH_2), 4.34 – 4.19 (m, 4 H, CH_2), 3.88 (t, *J* = 8.6 Hz, 4 H, CH_2), 3.72 (t, *J* = 7.9 Hz, 4 H, CH_2), 3.36 (s, 6 H, CH_3), 3.24 (s, 6 H, CH_3) ppm.

^{13}C NMR (150.92 MHz, CD_3CN, 295 K): δ = 172.89 (C_q, C_{gua}), 134.59 (C_q, C_{ring}), 115.84 (CH_{ring}), 60.34, 58.78, 31.38, 30.28 (CH_2), 37.80, 37.64 (CH_3) ppm.

^{11}B NMR (242.96 MHz, CD_3CN, 295 K): δ = –1.13 ppm.

^{19}F NMR (564.73 MHz, CD_3CN, 295 K): δ = –150.34 ppm.

UV-vis (CH_3CN, c = $5.71{\cdot}10^{-5}$ M): λ (ε in l mol^{-1} cm^{-1}) = 253 (40040), 362 (11220) nm.

Analytical data for (TGFA3+2H)$(BF_4)_4$

^{1}H NMR spectrum (600.18 MHz, 295 K) of TGFA3$(BF_4)_2$ and 3.8 eq. $HBF_4 \cdot Et_2O$ in CD_3CN

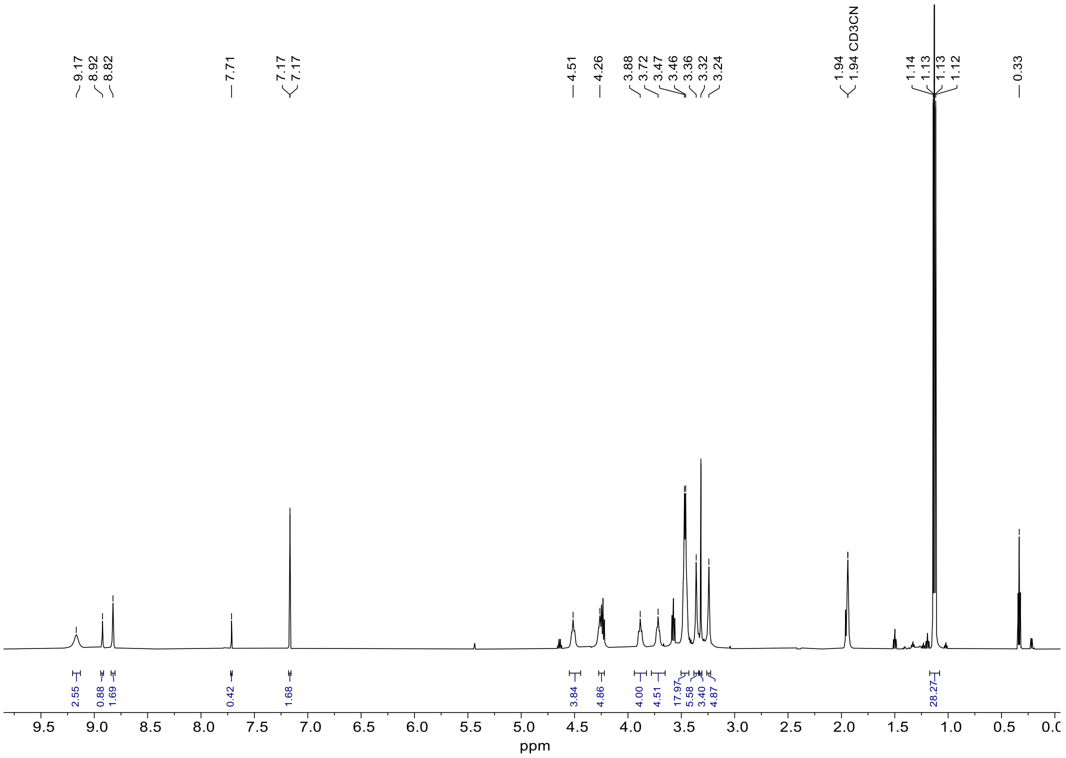

^{1}H NMR spectrum (600.18 MHz) of TGFA3$(BF_4)_2$ and 3.8 eq. $HBF_4{\cdot}Et_2O$ in CD_3CN with assignment of the signals

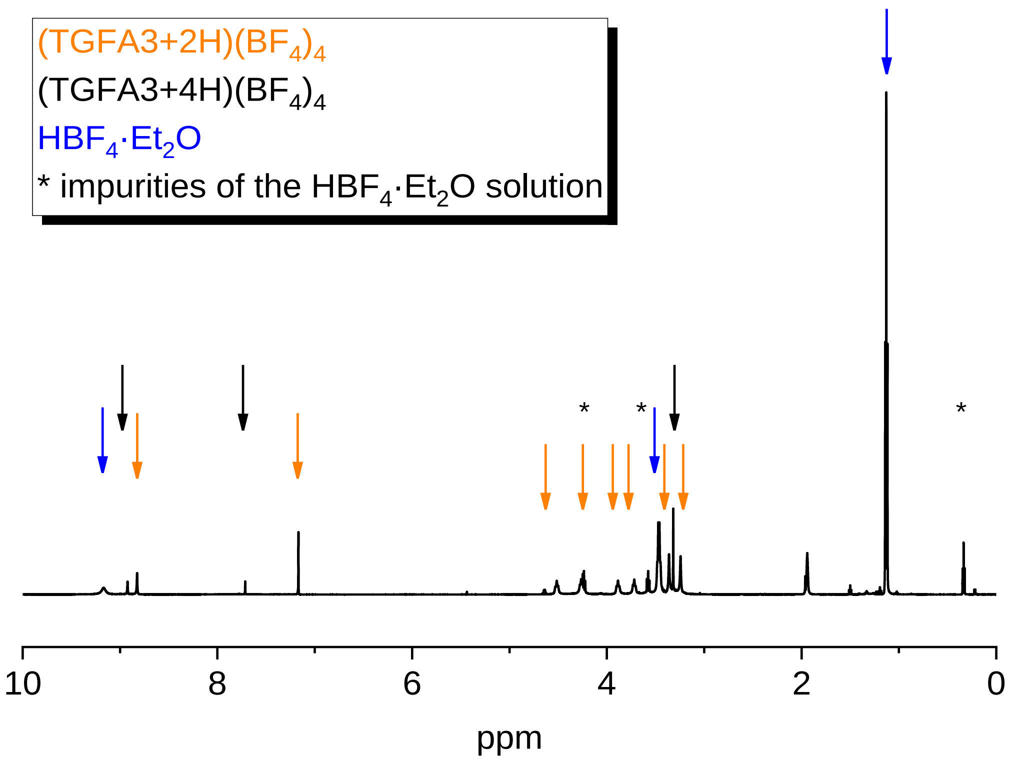

^{13}C NMR spectrum (150.92 MHz, 295 K) of TGFA3$(BF_4)_2$ and 3.8 eq. $HBF_4{\cdot}Et_2O$ in CD_3CN.

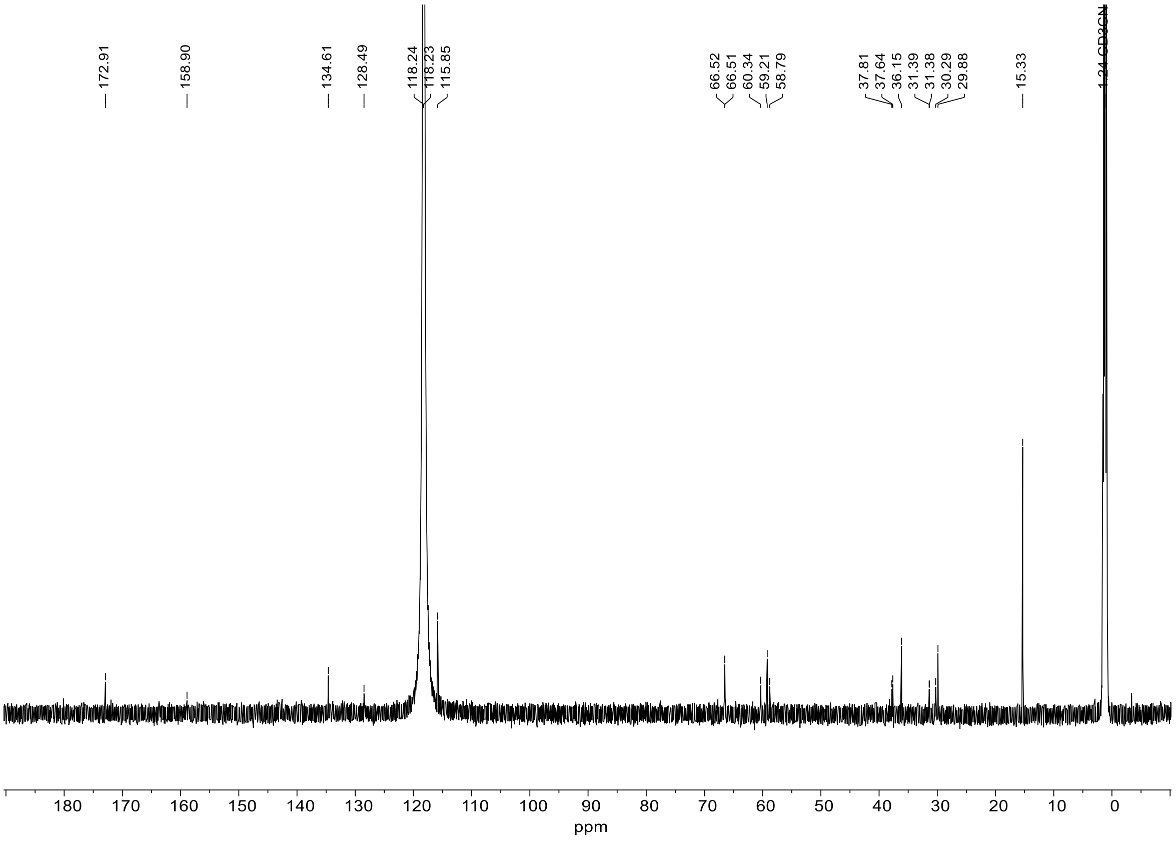

UV-vis spectrum of TGFA3(BF_4)$_2$ with 60 eq. $HBF_4 \cdot Et_2O$ in CH_3CN

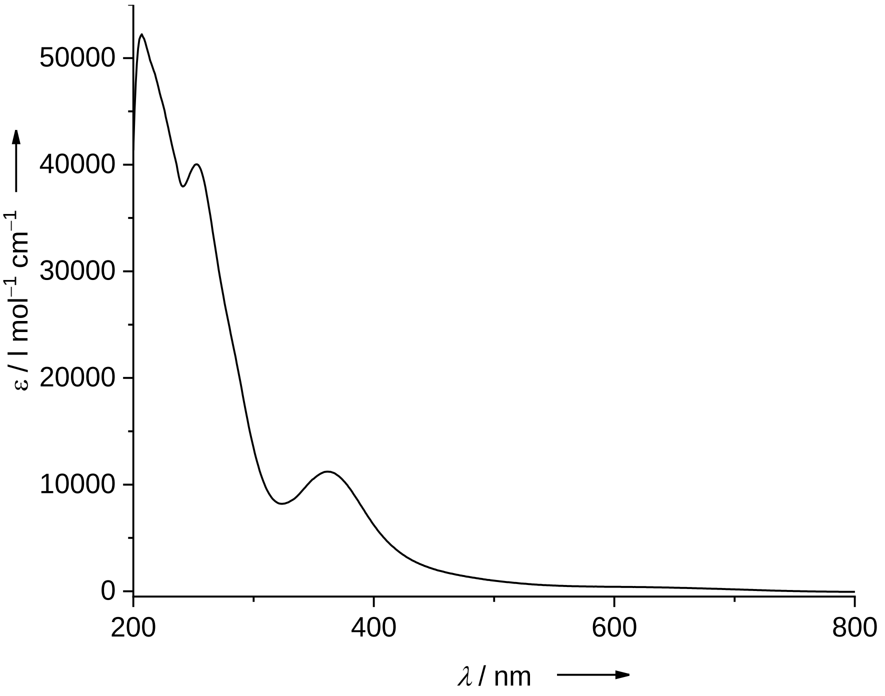

Please note: Due to the low concentration used in the UV-vis experiments, a large acid concentration is necessary to obtain solutions that contain only the double-protonated form, (TGFA3+2H)$^{4+}$.

Catalytic oxidation of (GFA3+2H)(PF_6)$_2$ with O_2

$$\text{(TGFA3+2H)(PF}_6)_2 + \text{excess O}_2 \xrightarrow[\text{CD}_3\text{CN / O}_2\text{, r.t.}]{\text{ca. 4 mol\% CuCl}_2\text{+Cu(H}_2\text{O)}_6\text{(BF}_4)_2} \text{no reaction}$$

$$\text{(GFA3a+2H)(PF}_6)_2 + 1/2\ \text{O}_2 \xrightarrow[\text{CD}_3\text{CN / O}_2\text{, r.t.; ca. 22 min}]{\text{ca. 4 mol\% CuCl}_2\text{+Cu(H}_2\text{O)}_6\text{(BF}_4)_2} \text{(GFA3a)(PF}_6)_2 + \text{H}_2\text{O} \quad (> 99\%)$$

$$\text{(GFA3b+2H)(PF}_6)_2 + 1/2\ \text{O}_2 \xrightarrow[\text{CD}_3\text{CN / O}_2\text{, r.t.; ca. 25 h}]{\text{ca. 4 mol\% CuCl}_2\text{+Cu(H}_2\text{O)}_6\text{(BF}_4)_2} \text{(GFA3b)(PF}_6)_2 + \text{H}_2\text{O} \quad (77\%)$$

General protocol:

Stock solutions of the copper compounds in CH_3CN were prepared and the required portion of the solution transferred with a syringe into a Schlenk flask. After solvent removal under vacuum, the copper catalyst was re-dissolved in CD_3CN (saturated with O_2). Then, the solutions were transferred under an O_2 atmosphere into an NMR tube containing (GFA3+2H)$(PF_6)_2$.

	TGFA3$(PF_6)_2$	GFA3a$(PF_6)_2$[a]	GFA3b$(PF_6)_2$
(GFA3)(+2H)$(PF_6)_2$ [mg / µmol]	6.476 / 7.67	6.338 / 7.70	6.294 / 7.73
$CuCl_2$ [µmol]	0.3 (4.0 mol %)	0.3 (4.1 mol%)	0.3 (3.9 mol %)
$Cu(H_2O)_6(BF_4)_2$ [µmol]	0.2 (2.7 mol %)	0.2 (2.7 mol%)	0.2 (2.6 mol %)
CD_3CN [ml]	0.6	0.6	0.6
Na_2SO_4 [mg]	1.214	1.086	1.642
reaction time	25 h	22 min	25 h
conversion (%)	0	100	77

[a] See ref. [14].

^1H NMR spectrum (600.15 MHz, 298 K, CD_3CN) of the catalytic oxidation of (TGFA3+2H)$(PF_6)_2$ with O_2 after 25 h, showing no conversion

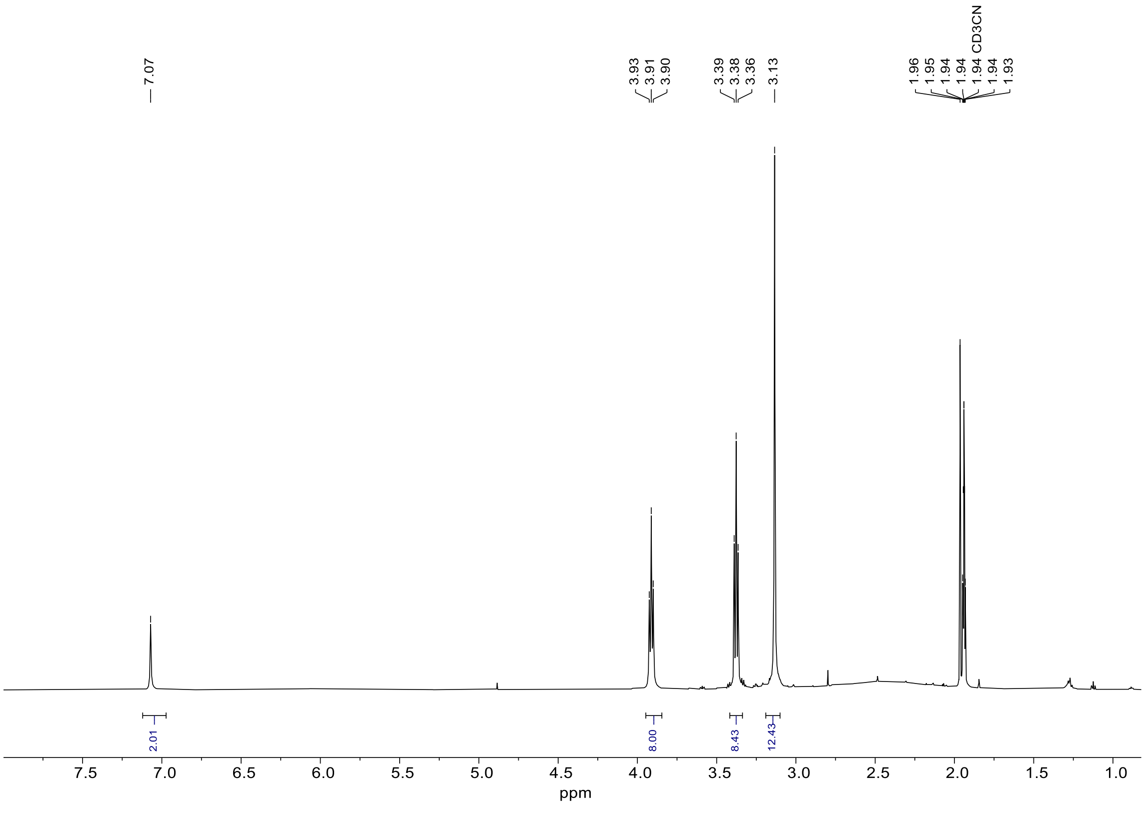

^1H NMR spectrum (600.15 MHz, 298 K, CD_3CN) of the catalytic oxidation of (GFA3b+2H)$(PF_6)_2$ with O_2 after 25 h

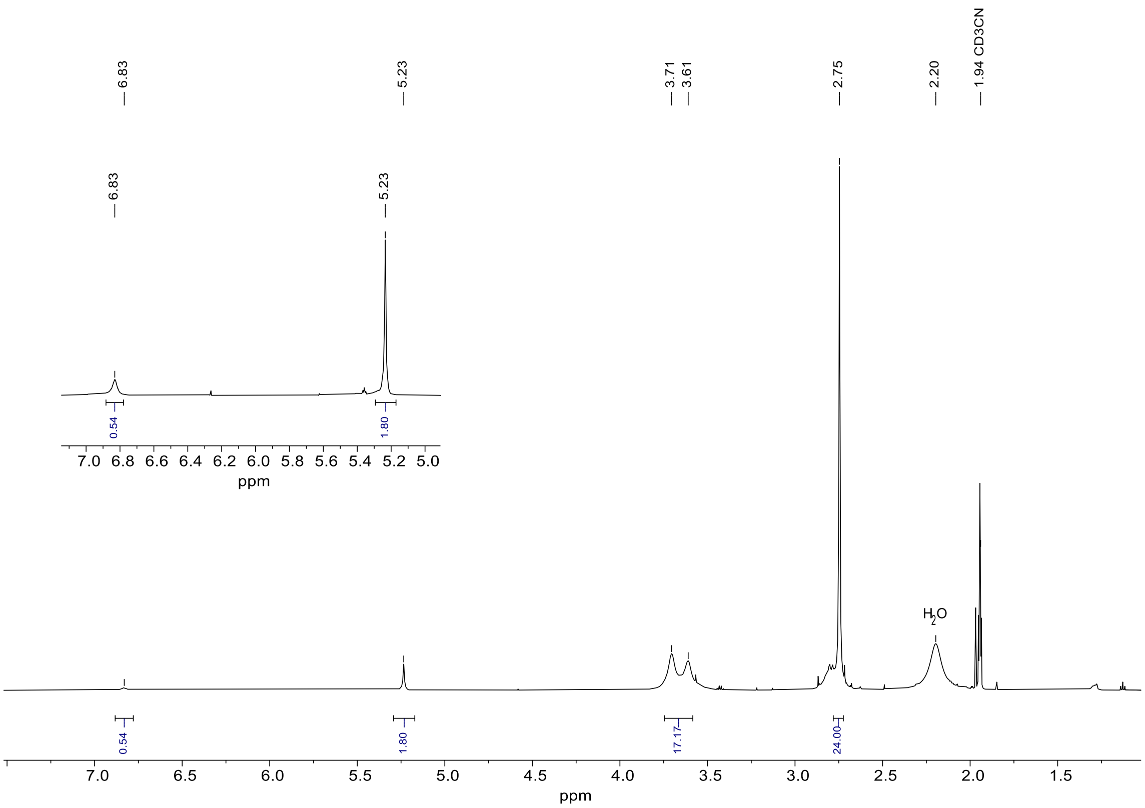

Dehydrogenative P-P coupling of diarylphosphanes: reaction of TGFA3$(PF_6)_2$ with $(MeOPh)_2PH$ to give $(MeOPh)_2P$-$P(MeOPh)_2$

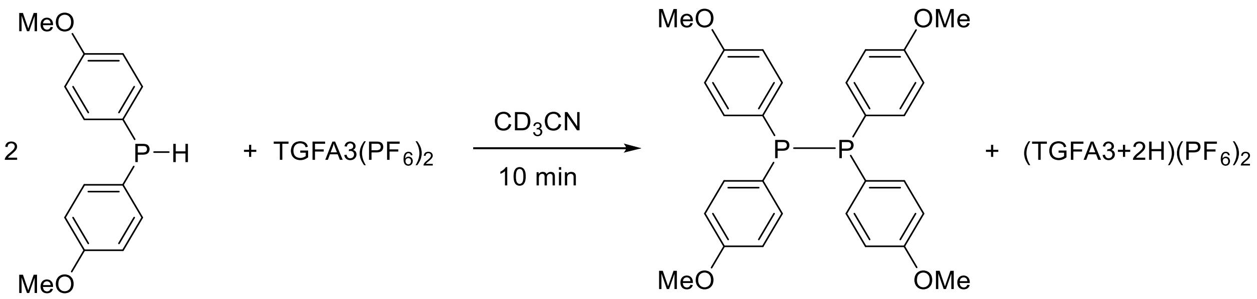

In an NMR tube, 5.306 mg of TGFA3$(PF_6)_2$ were dried at 45 °C under vacuum. Then, 3.2 mg of $(MeOPh)_2PH$ was added and the mixture was dissolved in 0.5 ml CD_3CN. The NMR tube was flame-sealed under vacuum. The color of the reaction mixture brightened up within 10 min.

Conversion (estimated from the ^{31}P NMR spectrum) of $(MeOPh)_2PH$ to give $(MeOPh)_2P$-$P(MeOPh)_2$: quantitative within 10 min

^{31}P NMR spectrum (CD_3CN, 242.94 MHz, 298 K) for the reaction of TGFA3$(PF_6)_2$ with $(MeOPh)_2PH$ in a 1:1.4 ratio (estimated from the signals in the ^1H NMR spectrum) after 10 min

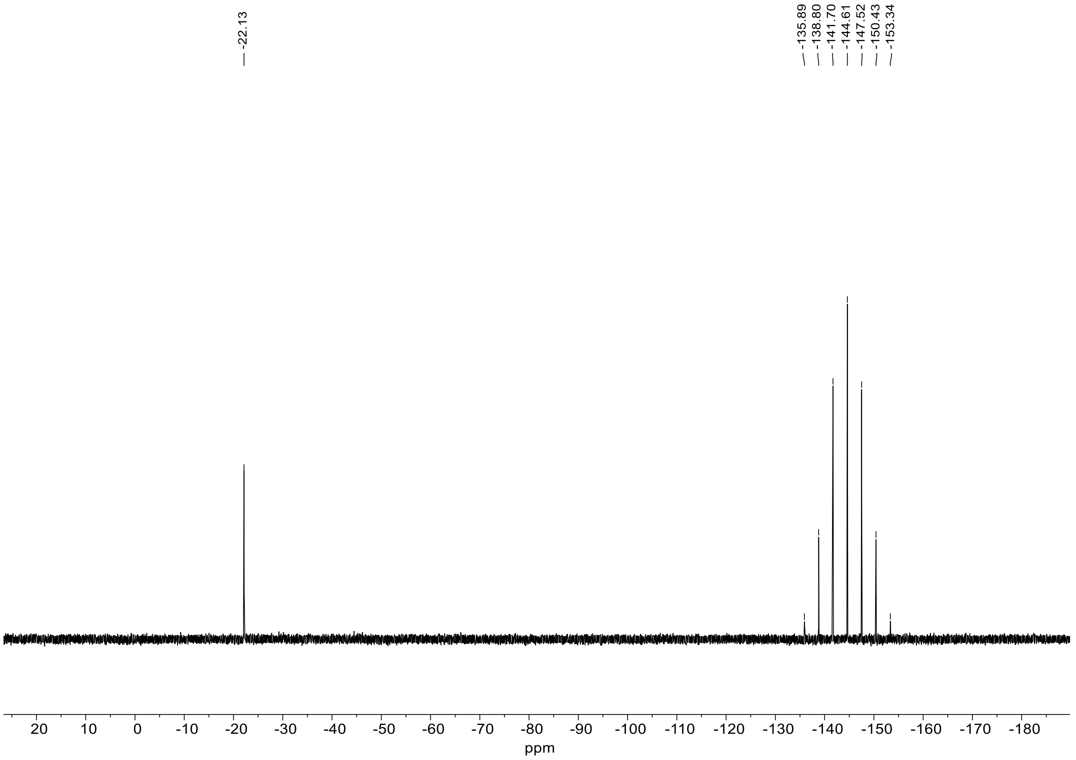

^1H NMR spectrum (CD_3CN, 600.15 MHz, 298 K) for the reaction of TGFA3$(PF_6)_2$ with $(MeOPh)_2PH$ in a 1:1.4 ratio (estimated from the signals in the ^1H NMR spectrum) after 10 min

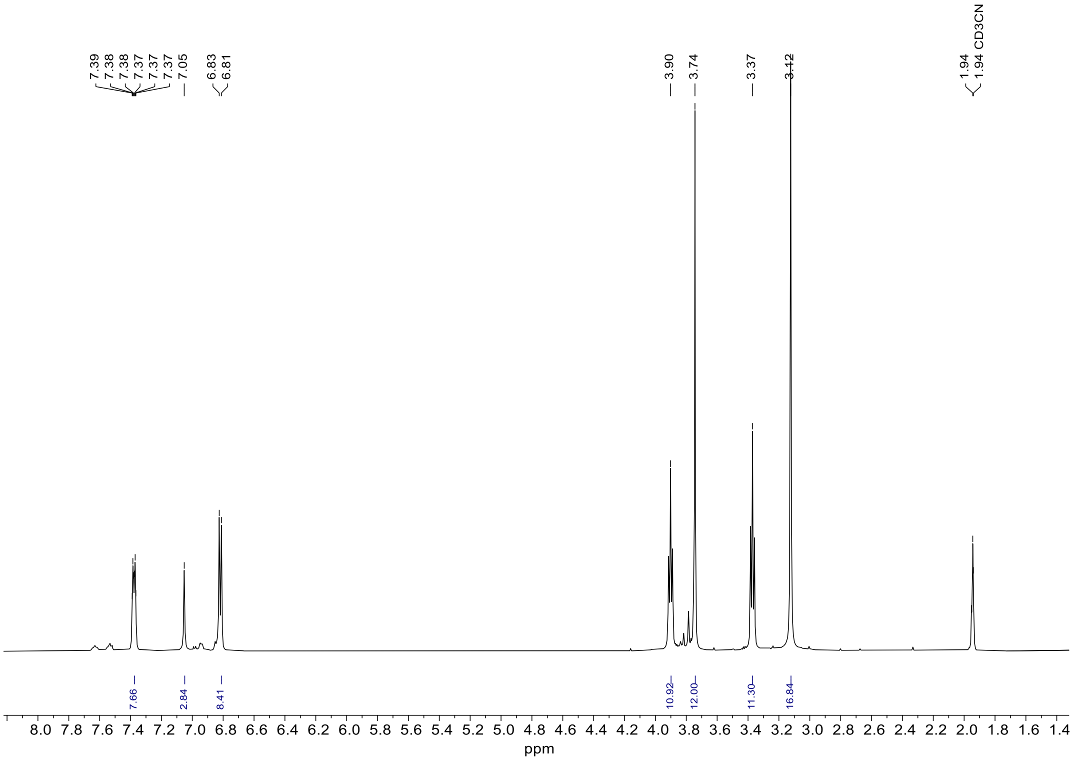

^{1}H NMR for $(MeOPh)_2P$-$P(MeOPh)_2$ (600.15 MHz, CD_3CN, 298 K): δ = 7.38 (dt, J = 8.9, 2.7 Hz, 8 H, CH_{arom}), 6.82 (d, J = 8.4 Hz, 8 H, CH_{arom}), 3.74 (s, 12 H, CH_3) ppm.

^{31}P NMR for $(MeOPh)_2P$-$P(MeOPh)_2$ (242.95 MHz, CD_3CN, 298 K): δ = –22.13 ppm.

^{1}H NMR for (TGFA3+2H)$(PF_6)_2$ (600.15 MHz, CD_3CN, 298 K): δ = 7.05 (s, 2 H, CH_{arom}), 3.90 (t, J = 7.5 Hz, 8 H, CH_2), 3.37 (t, J = 7.5 Hz, 8 H, CH_2), 3.12 (s, 12 H, CH_3) ppm.

^{31}P NMR for (TGFA3+2H)$(PF_6)_2$ (242.95 MHz, CD_3CN, 298 K): δ = –144.61 (sept) ppm.

Dehydrogenative P-P coupling of diarylphosphanes: reaction of GFA3b$(PF_6)_2$ with $(MeOPh)_2PH$ to give $(MeOPh)_2P$-$P(MeOPh)_2$

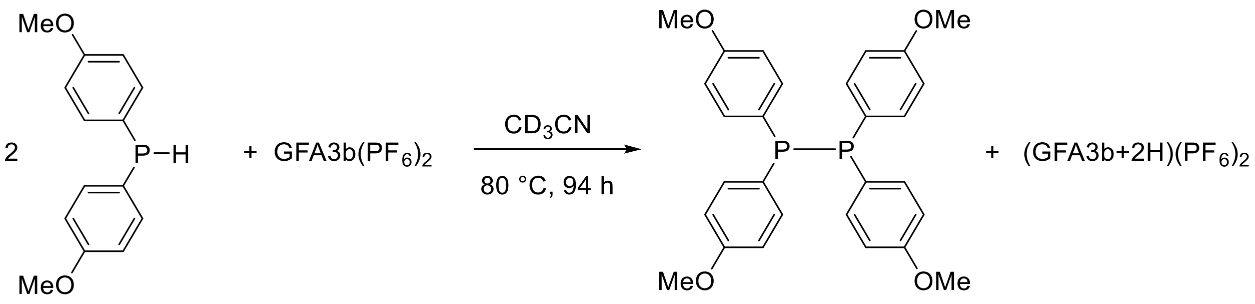

In an NMR tube, 5.074 mg of GFA3b$(PF_6)_2$ were dried at 45 °C under vacuum. Then, 2.6 mg of $(MeOPh)_2PH$ was added and the mixture was dissolved in 0.45 ml CD_3CN. The NMR tube was flame-sealed under vacuum and heated to 80 °C.

Conversion (estimated from the ^{31}P NMR spectrum) of $(MeOPh)_2PH$ to give $(MeOPh)_2P$-$P(MeOPh)_2$: quantitative within 93.6 h at 80 °C

^{31}P NMR spectra (CD_3CN, 242.95 MHz, 298 K) for the reaction of GFA3b(PF_6)$_2$ with ($MeOPh$)$_2$PH in a 1:1.8 ratio (estimated from the signals in the ^1H NMR spectrum)

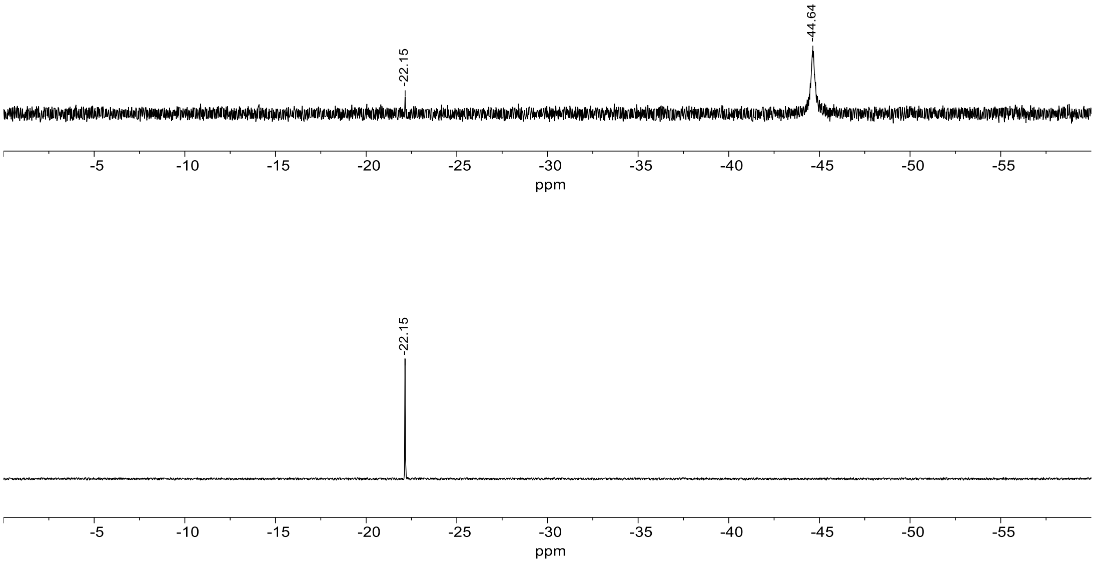

Top: first measurement after 20 min at r.t.; bottom: last measurement after 93.6 h at 80 °C

^{31}P NMR spectrum (CD_3CN, 242.96 MHz, 298 K) for the reaction of GFA3b(PF_6)$_2$ with ($MeOPh$)$_2$PH in a 1:1.8 ratio (estimated from the signals in the ^1H NMR spectrum), last measurement after 93.6 h at 80 °C

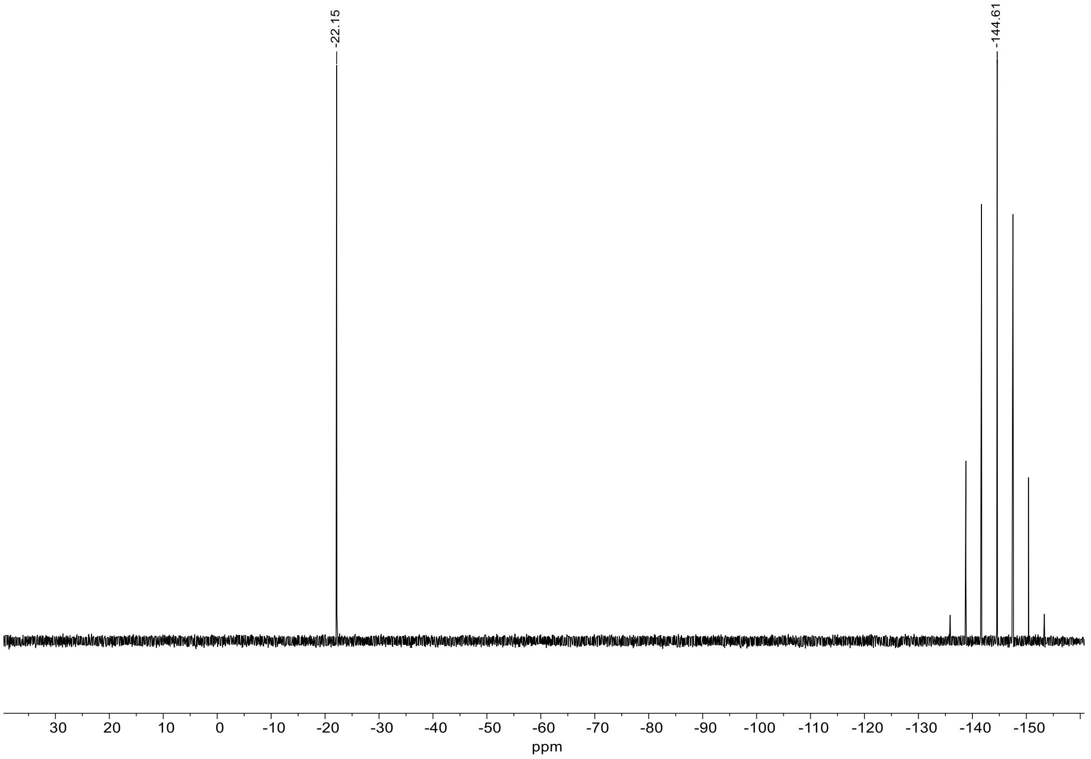

^{1}H NMR spectrum (CD_3CN, 600.15 MHz, 298 K) for the reaction of GFA3b(PF_6)$_2$ with (MeOPh)$_2$PH 1:1.8 ratio (estimated from the signals in the ^{1}H NMR spectrum), last measurement after 93.6 h at 80 °C

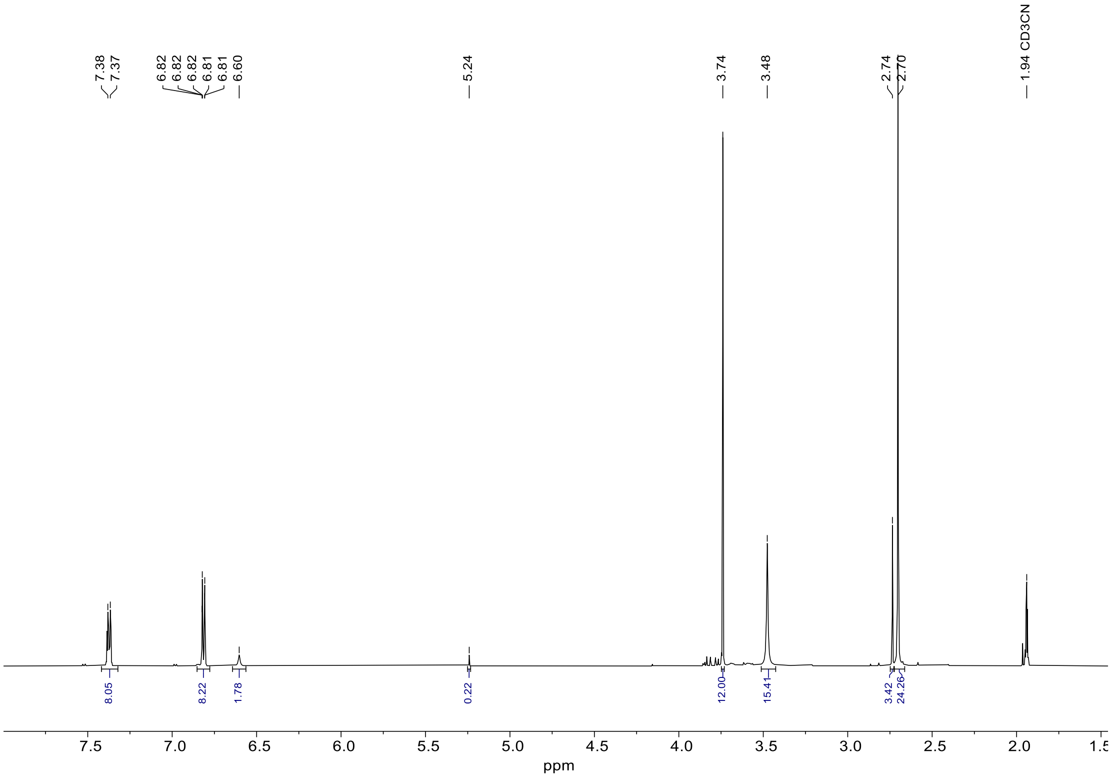

^{1}H NMR for (MeOPh)$_2$P-P(MeOPh)$_2$ (600.15 MHz, CD_3CN, 298 K): δ = 7.37 (dt, *J* = 8.8, 2.8 Hz, 8 H, CH_{arom}), 6.85 – 6.78 (m, 8 H, CH_{arom}), 3.74 (s, 12 H, CH_3) ppm.

^{31}P NMR for (MeOPh)$_2$P-P(MeOPh)$_2$ (242.96 MHz, CD_3CN, 298 K): δ = –22.15 ppm.

^{1}H NMR for (GFA3b+2H)(PF$_6$)$_2$ (600.15 MHz, CD_3CN, 298 K): δ = 6.60 (s, 2 H, CH_{arom}), 3.48 (s, 16 H, CH_2), 2.70 (s, 24 H, CH_3) ppm.

^{31}P NMR for (GFA3b+2H)(PF$_6$)$_2$ (242.95 MHz, CD_3CN, 298 K): δ = –144.61 (sept) ppm.

^{31}P NMR for (MeOPh)$_2$PH (242.95 MHz, CD_3CN, 298 K): δ = –44.64 ppm.

Conversion versus time plot from ^{31}P NMR experiments for the reaction of GFA3b(PF_6)$_2$ with (MeOPh)$_2$PH (1:1.8 ratio, estimated from the signals in the ^1H NMR spectrum)

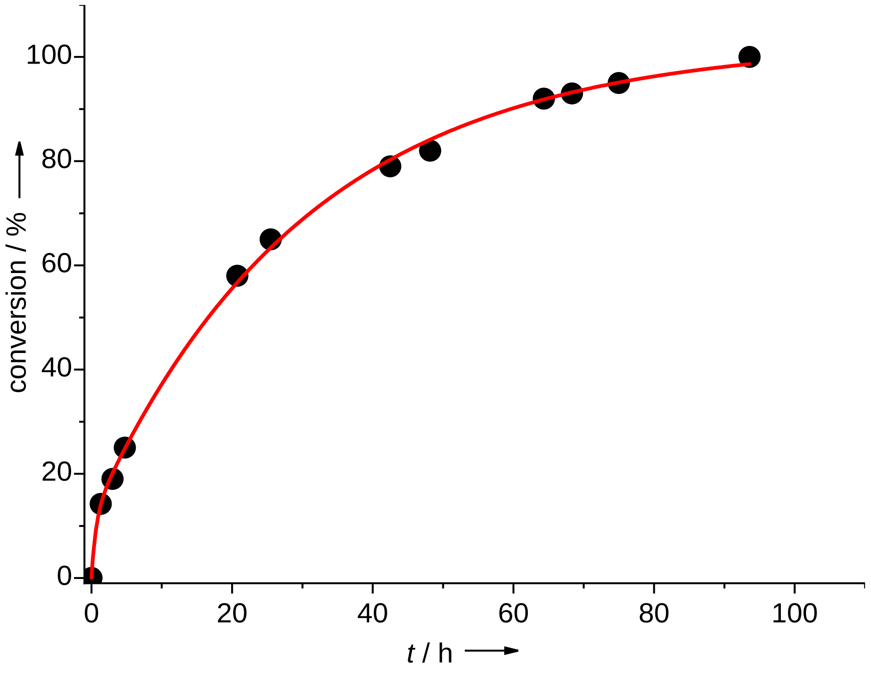

Oxidation of hydroquinones and derivatives to quinones

NMR experiments

Hydroquinone oxidation

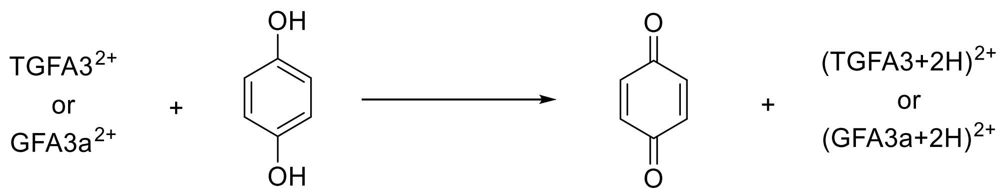

General protocol for hydroquinone oxidation:

The TGFA3(PF_6)$_2$ or GFA3a(PF_6)$_2$ and hydroquinone were added under an argon atmosphere in an NMR tube and dissolved in CD_3CN.

	TGFA3(PF_6)$_2$	TGFA3(PF_6)$_2$
TGFA(PF_6)$_2$ [mg]	5.26	4.782 + 2.16[c]
hydroquinone [mg]	0.546	0.714
CD_3CN [ml]	0.45	0.45
ratio TGFA3(PF_6)$_2$ / hydroquinone[a]	1.11 / 1	1.06 / 1
reaction time	25 min	see footnote c
benzoquinone [%][b]	95	> 99

[a] Estimated from the signals in the ^1H NMR spectra.
[b] Conversion estimated from the ratio of hydroquinone and quinone signals.
[c] A second portion of TGFA3(PF_6)$_2$ was added after 1 d.

Reaction between TGFA3(PF_6)$_2$ and hydroquinone (the colour brightened up immediately).

^1H NMR spectrum (400.20 MHz, CD_3CN, 298 K) after 25 min reaction time

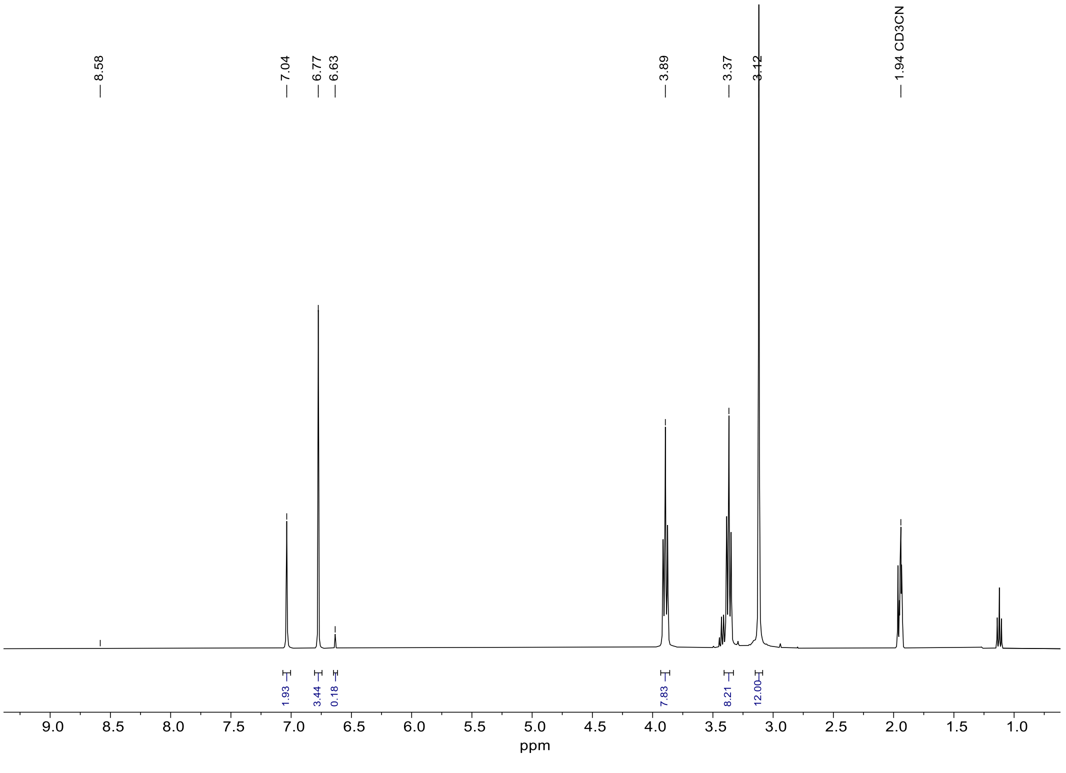

^{1}H NMR spectra (600.18 MHz, CD_3CN, 298 K) for the reaction between TGFA3$(PF_6)_2$ and hydroquinone (top); and upon addition of a second portion of TGFA3$(PF_6)_2$ after 1 d (bottom)

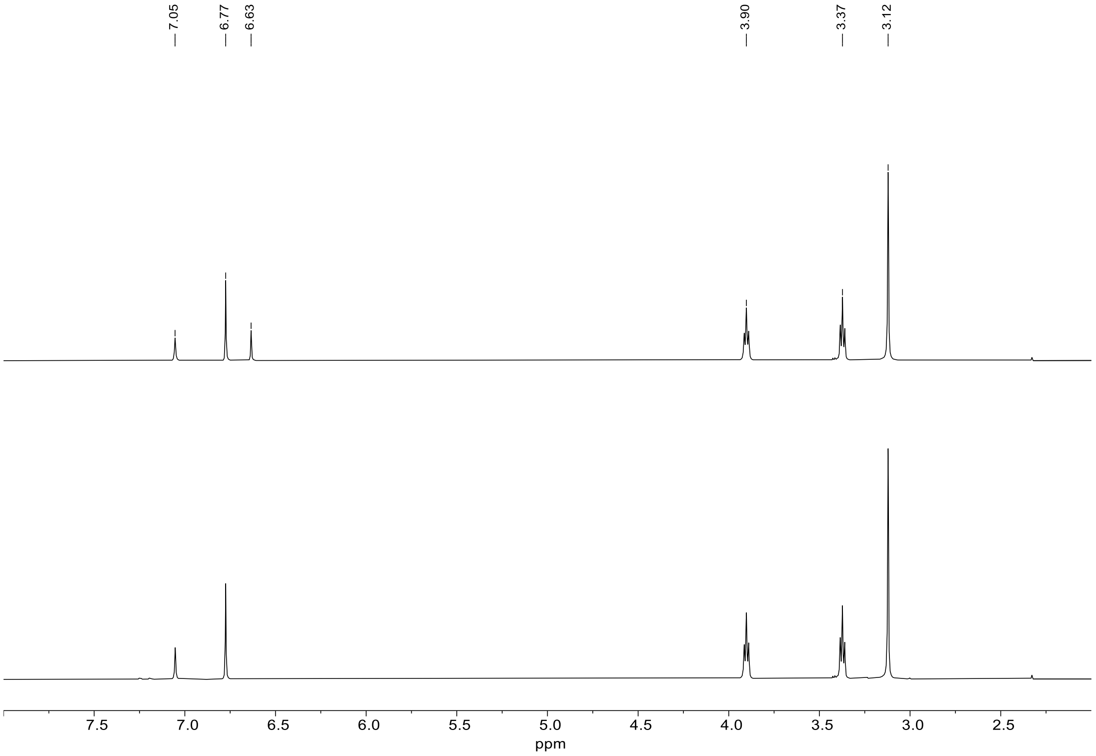

	GFA3a$(PF_6)_2$	GFA3a$(PF_6)_2$
GFA3a$(PF_6)_2$ [mg]	5.03	5.132 + 1.172[c]
hydroquinone [mg]	0.548	0.690
CD_3CN [ml]	0.45	0.45
ratio GFA3a$(PF_6)_2$ / hydroquinone[a]	1.52 / 1	1.56 / 1
reaction time	45 min	see footnote c
benzoquinone [%][b]	95	93

[a] Estimated from the signals in the ^{1}H NMR spectra.
[b] Conversion estimated from the ratio of hydroquinone and benzoquinone signals.
[c] A second portion of GFA3a$(PF_6)_2$ was added after 2 d.

Reaction between GFA3a(PF_6)$_2$ and hydroquinone

^{1}H NMR spectrum (400.20 MHz, CD_3CN, 298 K) after 45 min reaction time

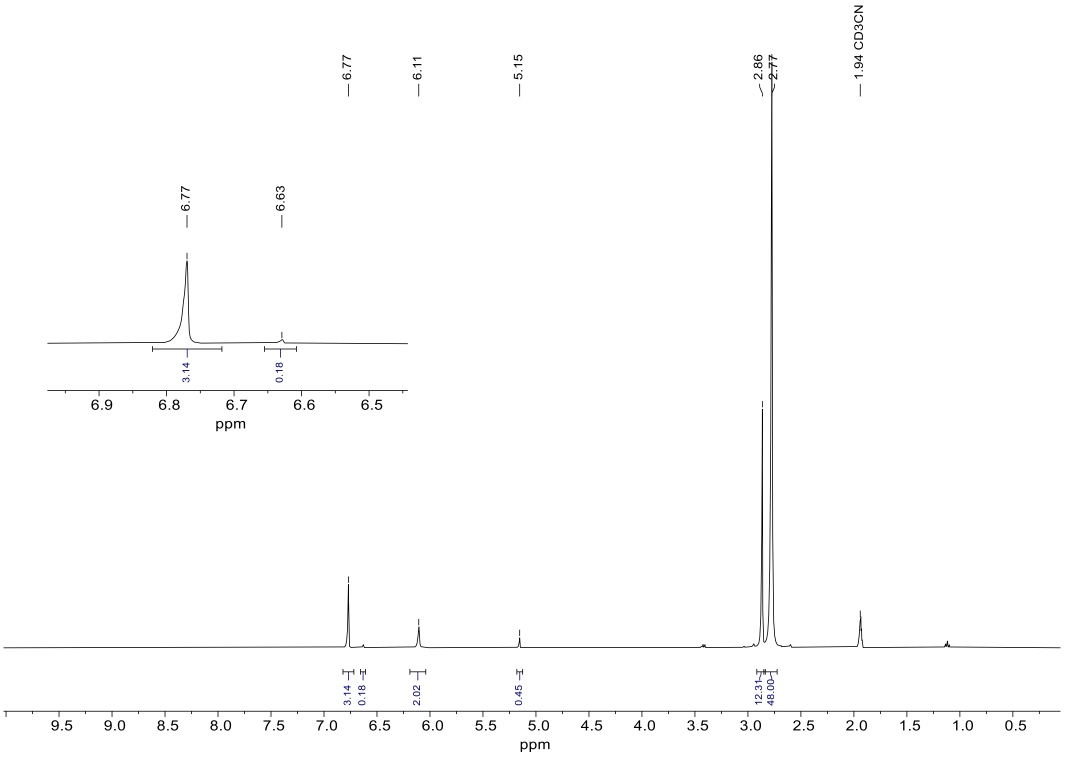

^{1}H NMR spectra (600.18 MHz, CD_3CN, 298 K) for the reaction between GFA3a(PF_6)$_2$ and hydroquinone (top), and upon addition of a second portion of GFA3a(PF_6)$_2$ after 2 d (bottom)

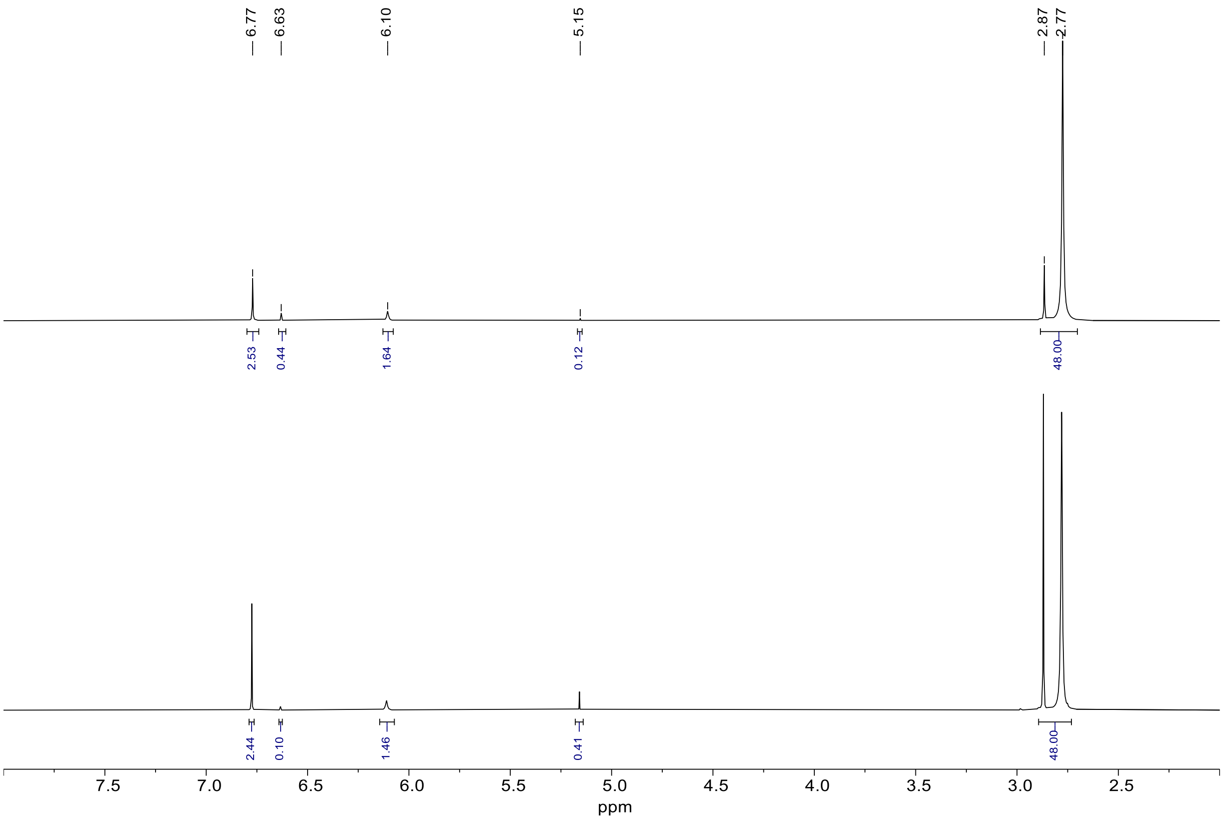

^{1}H NMR chemical shifts in CD_3CN

	δ [ppm]
(TGFA3+2H)$(PF_6)_2$	7.05 (s, 2 H), 3.90 (t, 8 H), 3.37 (t, 8 H), 3.12 (s, 12 H)
GFA3a$(PF_6)_2$	5.15 (s, 2 H), 2.87 (s, 48 H)
(GFA3a+2H)$(PF_6)_2$	6.10 (s, 2 H), 2.77 (s, 48 H)
hydroquinone	6.63 (s, 4 H)
benzoquinone	6.77 (s, 4 H)

2-Chlorobenzene-1,4-diol oxidation

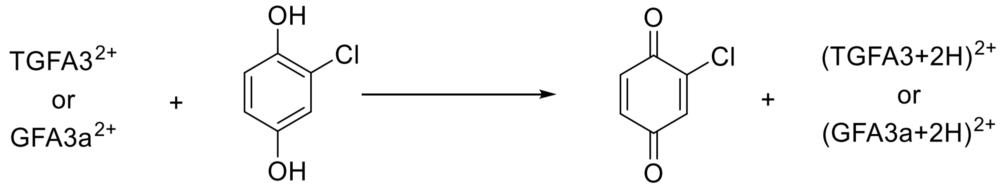

General protocol for 2-chlorobenzene-1,4-diol oxidation:

The TGFA3$(PF_6)_2$ or GFA3a$(PF_6)_2$ and 2-chlorobenzene-1,4-diol were added under an argon atmosphere in an NMR tube and dissolved in CD_3CN.

	TGFA3$(PF_6)_2$	GFA3a$(PF_6)_2$
TGFA3$(PF_6)_2$ / GFA3a$(PF_6)_2$ [mg]	4.942	5.124
2-chlorobenzene-1,4-diol [mg]	0.746	0.768
CD_3CN [ml]	0.45	0.45
ratio GFA$(PF_6)_2$[a] / 2-chlorobenzene-1,4-diol[b]	1 / 1	1.29 / 1
reaction time	54 min	50 min
2-chloro-1,4-benzoquinone [%][c]	84	85

[a] GFA$(PF_6)_2$ denotes TGFA3$(PF_6)_2$ or GFA3a$(PF_6)_2$.
[b] Estimated from the signals in the ^{1}H NMR spectra.
[c] Conversion estimated from the ratio of 2-chlorobenzene-1,4-diol and 2-chloro-1,4-benzoquinone signals.

Reaction between TGFA3$(PF_6)_2$ and 2-chlorobenzene-1,4-diol (the colour brightened up immediately).

^{1}H NMR spectrum (400.20 MHz, CD_3CN, 298 K) after 54 min reaction time

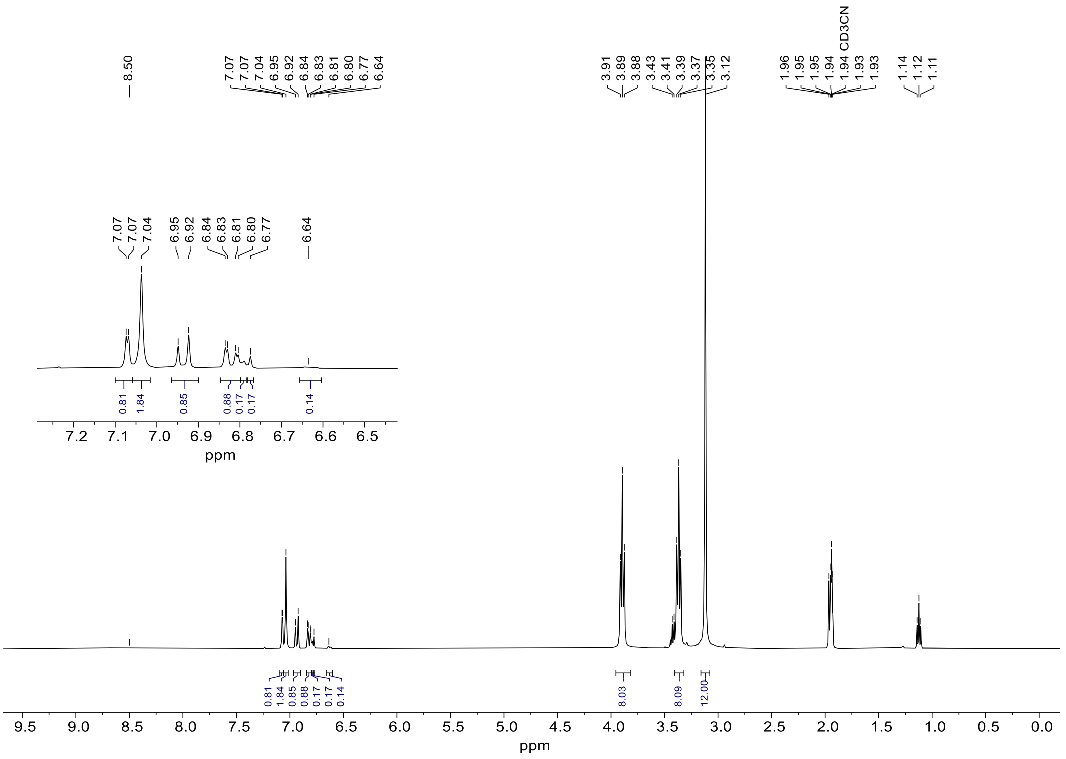

Reaction between GFA3a$(PF_6)_2$ and 2-chlorobenzene-1,4-diol

^{1}H NMR spectrum (400.20 MHz, CD_3CN, 298 K) after 50 min reaction time

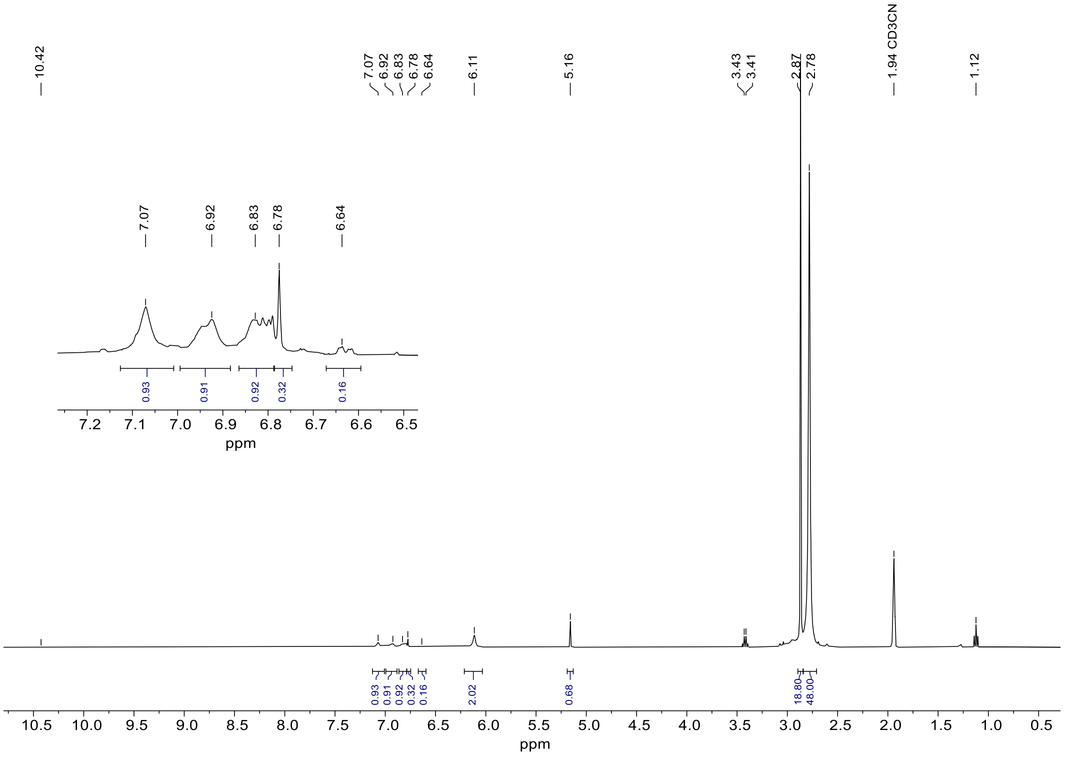

^{1}H NMR chemical shifts in CD_3CN

	δ [ppm]
(TGFA3+2H)(PF_6)$_2$	7.04 (s, 2 H), 3.89 (t, 8 H), 3.37 (t, 8 H), 3.12 (s, 12 H)
GFA3a(PF_6)$_2$	5.16 (s, 2 H), 2.87 (s, 48 H)
(GFA3a+2H)(PF_6)$_2$	6.11 (s, 2 H), 2.78 (s, 48 H)
2-chlorobenzene-1,4-diol	6.63 (dd, 1 H), 6.84 – 6.80 (m, 2 H)
2-chloro-1,4-benzoquinone	7.07 (d, 1 H), 6.94 (d, 1 H), 6.82 (dd, 1 H)

2-Bromobenzene-1,4-diol oxidation

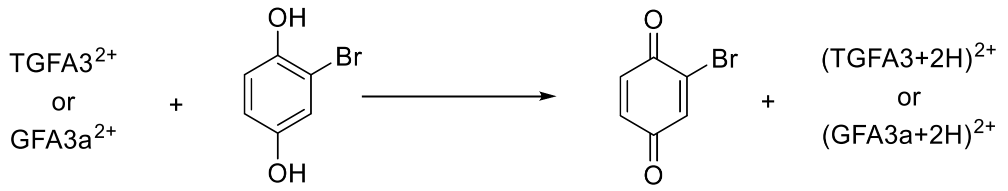

General protocol for 2-bromobenzene-1,4-diol oxidation:

The TGFA3(PF_6)$_2$ or GFA3a(PF_6)$_2$ and 2-bromobenzene-1,4-diol were added under an argon atmosphere in an NMR tube and dissolved in CD_3CN.

	TGFA3(PF_6)$_2$	GFA3a(PF_6)$_2$
TGFA3(PF_6)$_2$ / GFA3a(PF_6)$_2$ [mg]	5.076	5.066
2-bromobenzene-1,4-diol [mg]	1.054	1.072
CD_3CN [ml]	0.45	0.45
ratio GFA(PF_6)$_2$[a]/ 2- bromobenzene-1,4-diol[b]	0.96 / 1	1.30 / 1
reaction time	62 min	55 min
2-bromo-1,4-benzoquinone [%][c]	85	84

[a] GFA(PF_6)$_2$ denotes TGFA3(PF_6)$_2$ or GFA3a(PF_6)$_2$.
[b] Estimated from the signals in the ^{1}H NMR spectra.
[c] Conversion estimated from the ratio of 2-bromobenzene-1,4-diol and 2-bromo-1,4-benzoquinone signals.

Reaction between TGFA3(PF_6)$_2$ and 2-bromobenzene-1,4-diol (the colour brightened up immediately).

^{1}H NMR spectrum (400.20 MHz, CD_3CN, 298 K) after 62 min reaction time

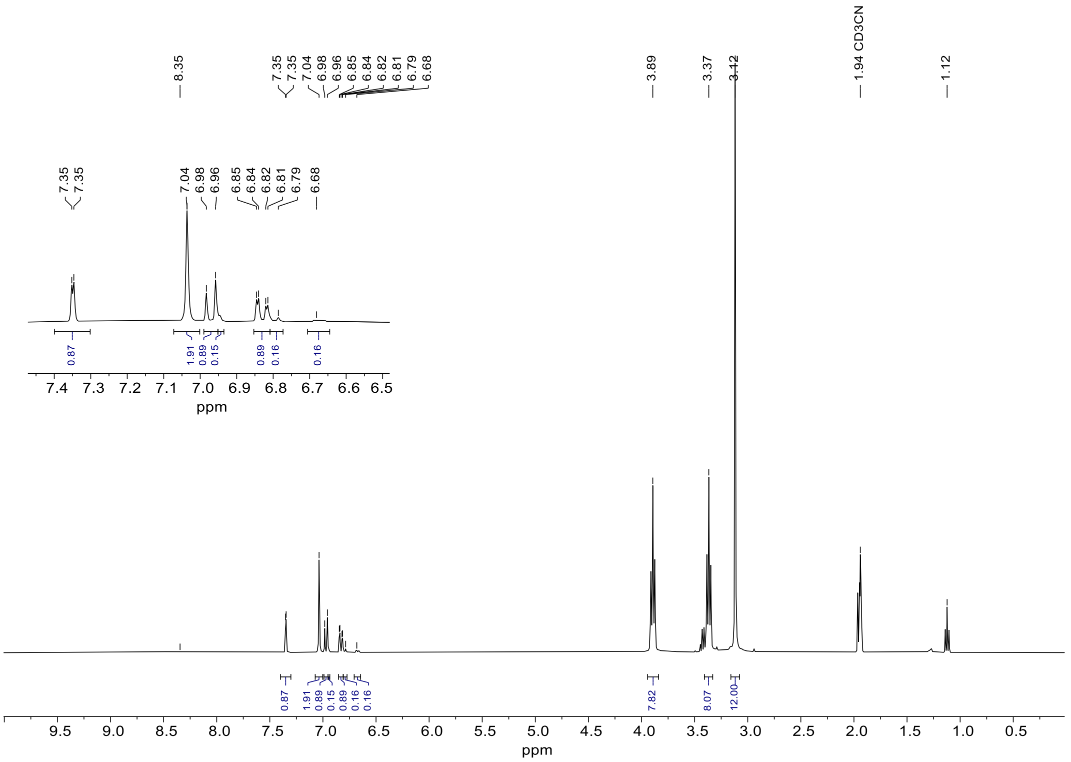

Reaction between GFA3a(PF_6)$_2$ and 2-bromobenzene-1,4-diol

^{1}H NMR spectrum (400.20 MHz, CD_3CN, 298 K) after 55 min reaction time

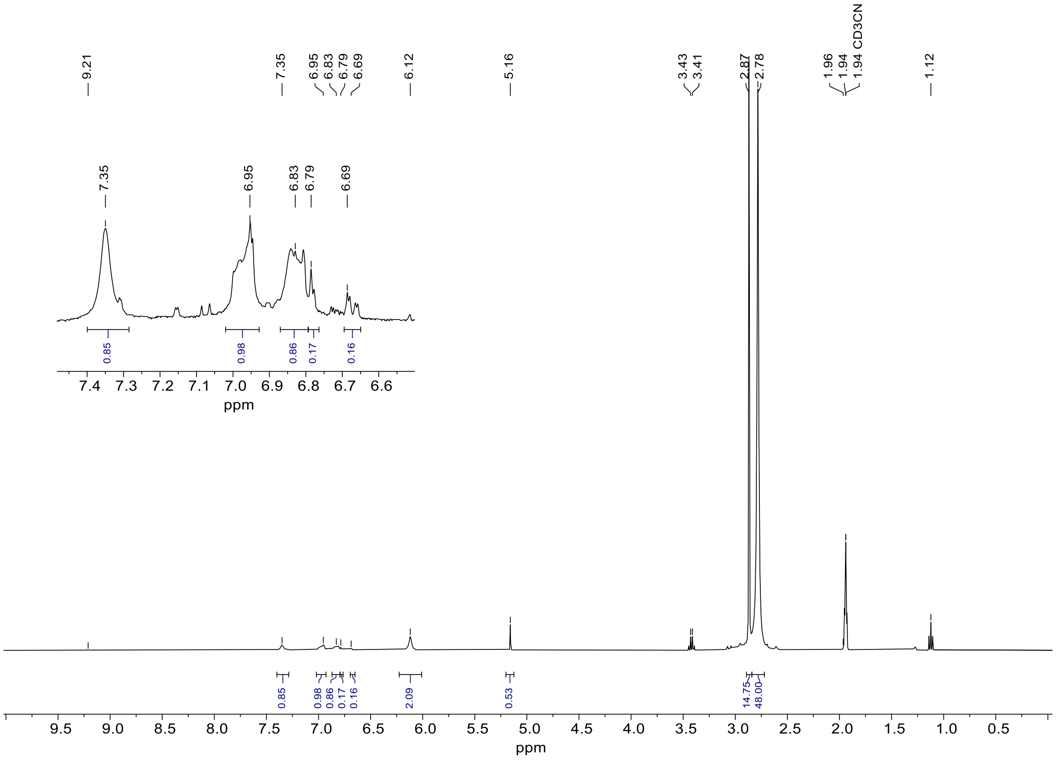

^1H NMR chemical shifts in CD_3CN

	δ [ppm]
$(TGFA3+2H)(PF_6)_2$	7.04 (s, 2 H), 3.89 (t, 8 H), 3.37 (t, 8 H), 3.12 (s, 12 H)
$GFA3a(PF_6)_2$	5.16 (s, 2 H), 2.87 (s, 48 H)
$(GFA3a+2H)(PF_6)_2$	6.12 (s, 2 H), 2.78 (s, 48 H)
2-bromobenzene-1,4-diol	6.95 (m, 1 H), 6.79 (m, 1 H), 6.68 (dd, 1 H)
2-bromo-1,4-benzoquinone	7.35 (d, 1 H), 6.97 (d, 1 H), 6.83 (dd, 1 H)

2,5-Dibromobenzene-1,4-diol oxidation

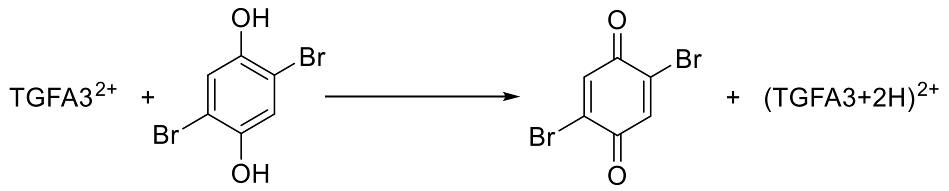

General protocol for 2,5-dibromobenzene-1,4-diol oxidation:

The $TGFA3(PF_6)_2$ and 2,5-dibromobenzene-1,4-diol were added under an argon atmosphere in an NMR tube and dissolved in CD_3CN.

	$TGFA3(PF_6)_2$
$TGFA3(PF_6)_2$ [mg]	4.948
2,5-dibromobenzene-1,4-diol [mg]	1.316
CD_3CN [ml]	0.45
ratio $TGFA3(PF_6)_2$ / 2,5-dibromobenzene-1,4-diol[a]	1.04 / 1
reaction time	60 min
2,5-dibromo-1,4-benzoquinone [%][b]	92

[a] Estimated from the signals in the ^1H NMR spectra.
[b] Conversion estimated from the ratio of 2,5-dibromobenzene-1,4-diol and 2,5-dibromo-1,4-benzoquinone signals.

Reaction between TGFA3$(PF_6)_2$ and 2,5-dibromobenzene-1,4-diol (the colour brightened up (yellow) immediately).

^{1}H NMR (400.20 MHz, CD_3CN, 298 K) after 60 min reaction time

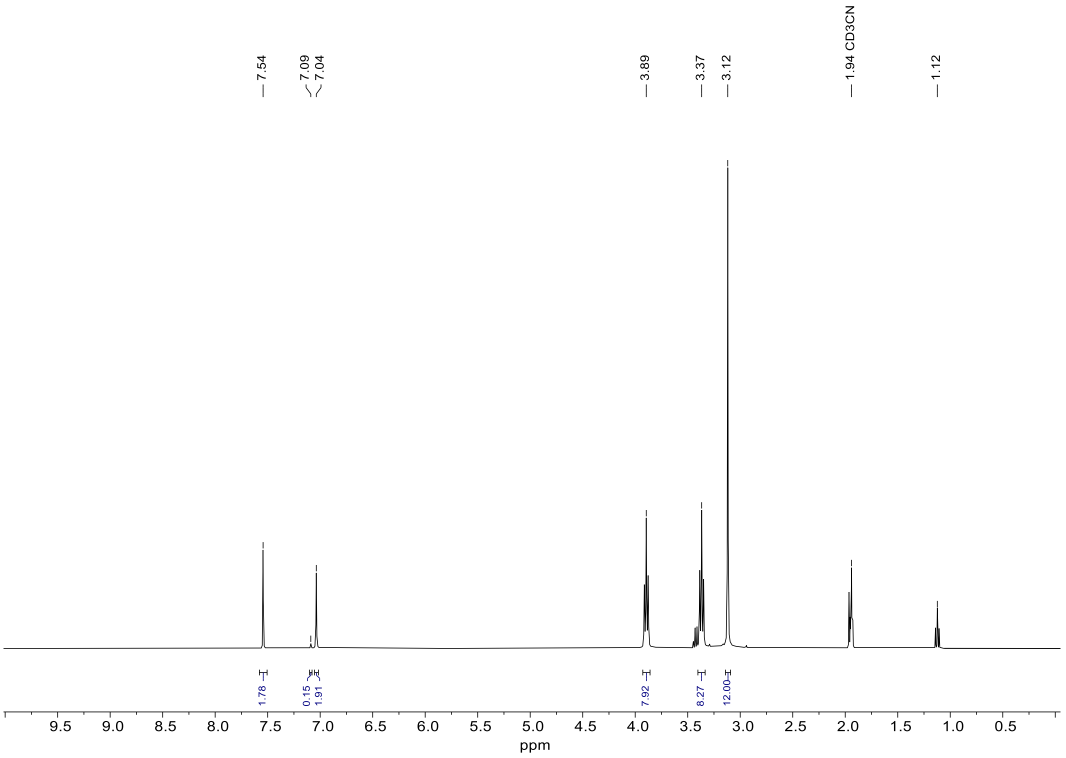

^{1}H NMR chemical shifts in CD_3CN

	δ [ppm]
(TGFA3+2H)$(PF_6)_2$	7.04 (s, 2 H), 3.89 (t, 8 H), 3.37 (t, 8 H), 3.12 (s, 12 H)
2,5-dibromobenzene-1,4-diol	7.09 (s, 2 H)
2,5-dibromo-1,4-benzoquinone	7.54 (s, 2 H)

UV-vis experiments

Hydroquinone oxidation

<u>With TGFA3(PF_6)$_2$</u>

Stoichiometric ratio

A solution of 0.598 mg of TGFA3(PF_6)$_2$ in 5 ml CH_3CN (c = $1.45\cdot10^{-4}$ M) and a solution of 0.356 mg of hydroquinone in 25 ml CH_3CN (c = $1.29\cdot10^{-4}$ M) were prepared. Under argon atmosphere a 1 cm quartz glass cuvette was filled with 1.4 ml (c = $6.49\cdot10^{-5}$ M [cuvette]) of the TGFA3(PF_6)$_2$ solution and with 1.53 ml (c = $6.32\cdot10^{-5}$ M [cuvette]) of the hydroquinone solution and 0.2 ml of CH_3CN.

UV-vis spectra for a 1:1 mixture of TGFA3(PF_6)$_2$ and hydroquinone in CH_3CN

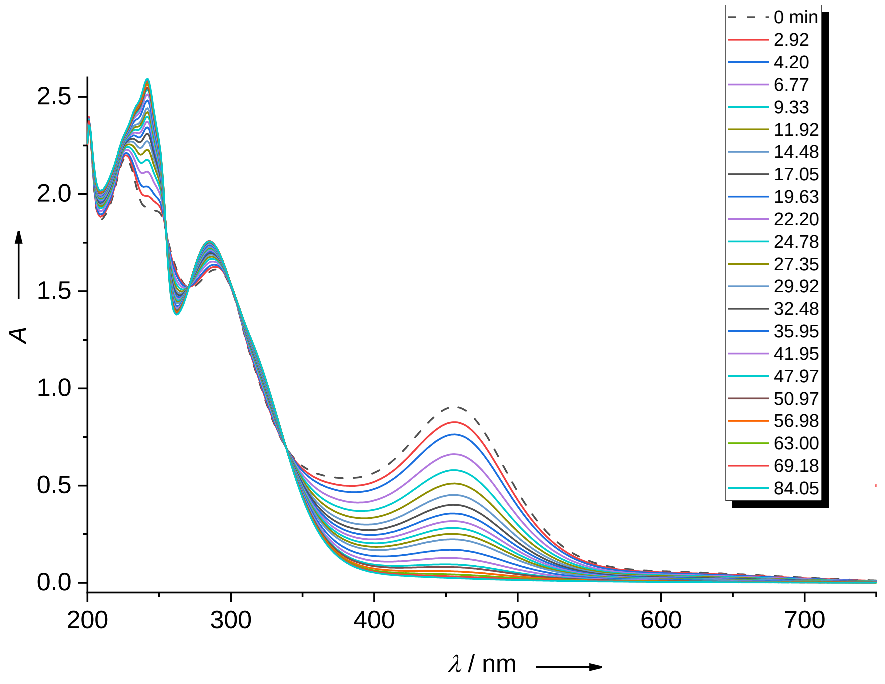

General protocol for the UV-vis kinetic studies:

Solution I: 1.476 mg of TGFA3(PF_6)$_2$ was dissolved in 25 ml CH_3CN, c = 7.16·10^{-5} M.

Solution II: 20.524 mg of hydroquinone was dissolved in 5 ml CH_3CN, 2 ml of this solution was diluted to 5 ml, c = 1.49·10^{-2} M.

	entry A	entry B	entry C	entry D
solution I [ml]	2.60	2.60	2.60	2.60
solution II [ml]	0.12	0.25	0.38	0.5
CH_3CN [ml]	0.48	0.35	0.22	0.1
TGFA3(PF_6)$_2$ [c, cuvette]	5.82·10^{-5} M	5.82·10^{-5} M	5.82·10^{-5} M	5.82·10^{-5} M
hydroquinone [c, cuvette]	5.59·10^{-4} M	1.16·10^{-3} M	1.77·10^{-3} M	2.33·10^{-3} M
equivalents of hydroquinone	9.6	20.0	30.4	40.1

UV-vis spectra recorded for the reaction of TGFA3(PF_6)$_2$ with 9.6 equivalents of hydroquinone in CH_3CN solution to give (TGFA3+2H)(PF_6)$_2$ and benzoquinone

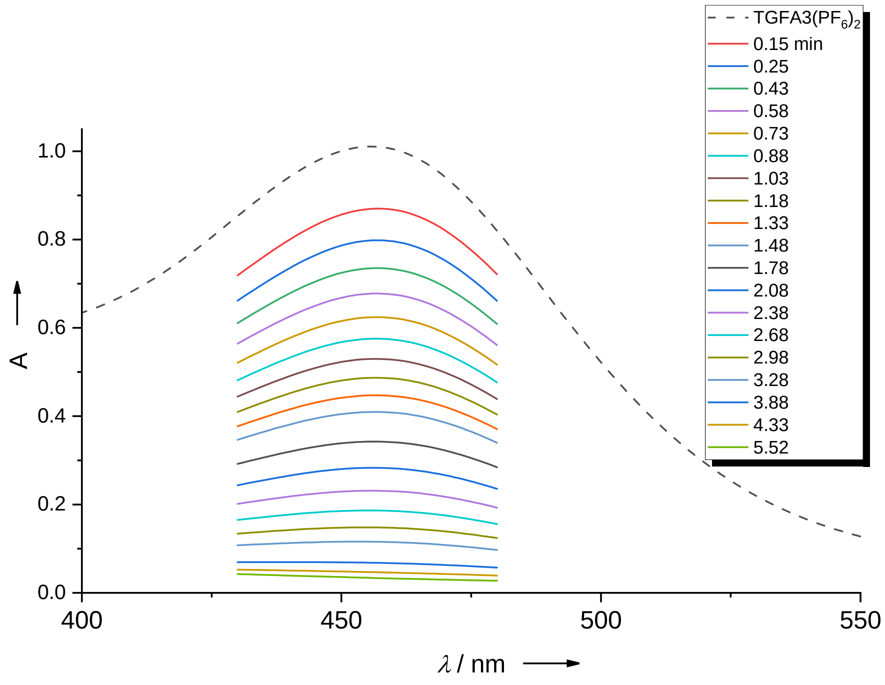

UV-vis spectra recorded for the reaction of TGFA3(PF_6)$_2$ with 40.1 equivalents of hydroquinone in CH_3CN solution to give (TGFA3+2H)(PF_6)$_2$ and benzoquinone

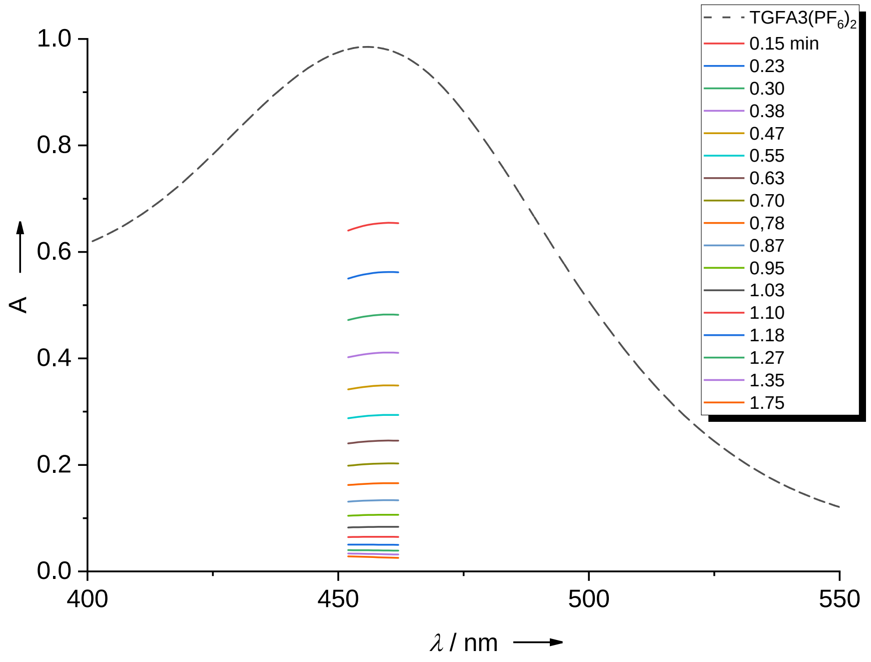

Plot of the absorption at 456 nm (band of the reactant (TGFA3)$^{2+}$) in dependence of the reaction time

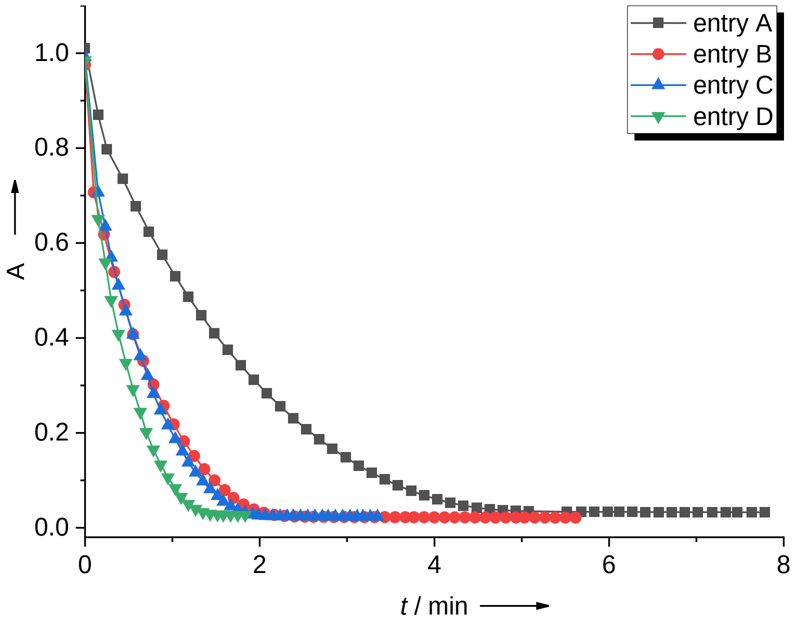

Second-order rate constant *k* obtained as the slope of the plot k_{obs} versus the hydroquinone concentration

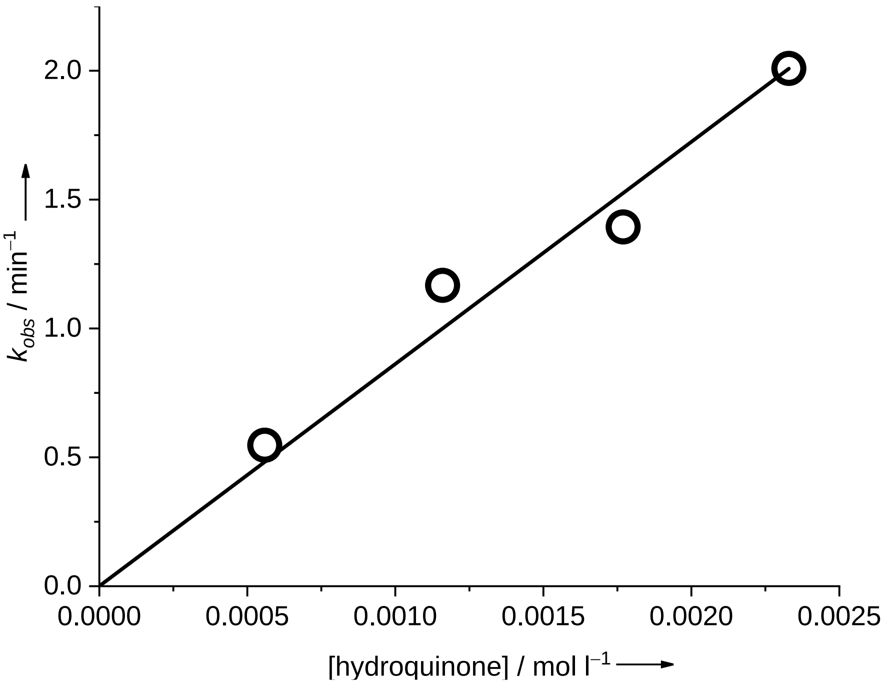

With GFA3a(PF_6)$_2$

Solution I: 1.436 mg of GFA3a(PF_6)$_2$ was dissolved in 25 ml CH_3CN, c = $7.00\cdot10^{-5}$ M.

Solution II: 20.524 mg of hydroquinone was dissolved in 5 ml CH_3CN, c = $3.73\cdot10^{-2}$ M.

	entry A	entry B	entry C	entry D
solution I [ml]	2.65	2.65	2.65	2.65
solution II [ml]	0.1	0.2	0.4	0.5
CH_3CN [ml]	0.45	0.35	0.15	0.05
GFA3a(PF_6)$_2$ [c, cuvette]	$5.80\cdot10^{-5}$ M	$5.80\cdot10^{-5}$ M	$5.80\cdot10^{-5}$ M	$5.80\cdot10^{-5}$ M
hydroquinone [c, cuvette]	$1.17\cdot10^{-3}$ M	$2.33\cdot10^{-3}$ M	$4.66\cdot10^{-3}$ M	$5.82\cdot10^{-3}$ M
equivalents of hydroquinone	20.1	40.2	80.3	100.4

UV-vis spectra recorded for the reaction of GFA3a(PF_6)$_2$ with 20.1 equivalents of hydroquinone in CH_3CN solution to give (GFA3a+2H)(PF_6)$_2$ and benzoquinone

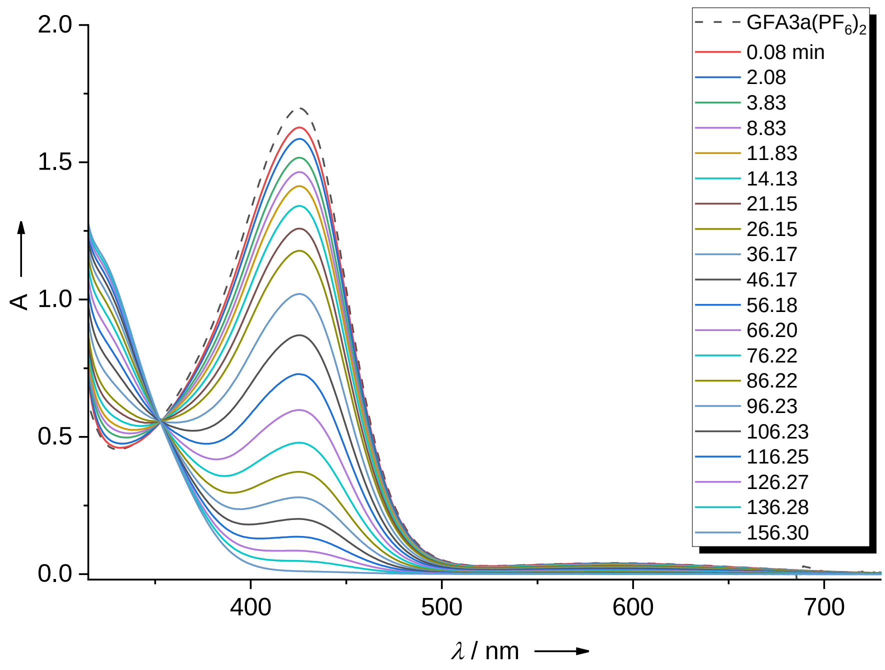

UV-vis spectra recorded for the reaction of GFA3a(PF_6)$_2$ with 100.4 equivalents of hydroquinone in CH_3CN solution to give (GFA3a+2H)(PF_6)$_2$ and benzoquinone

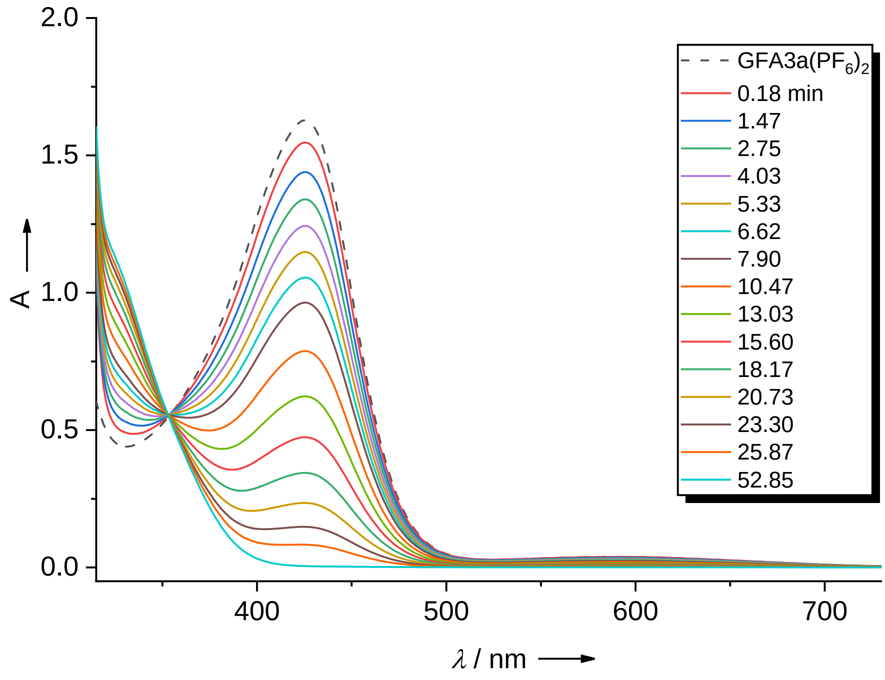

Plot of the absorption at 425 nm (band of the reactant $(GFA3a)^{2+}$) in dependence of the reaction time

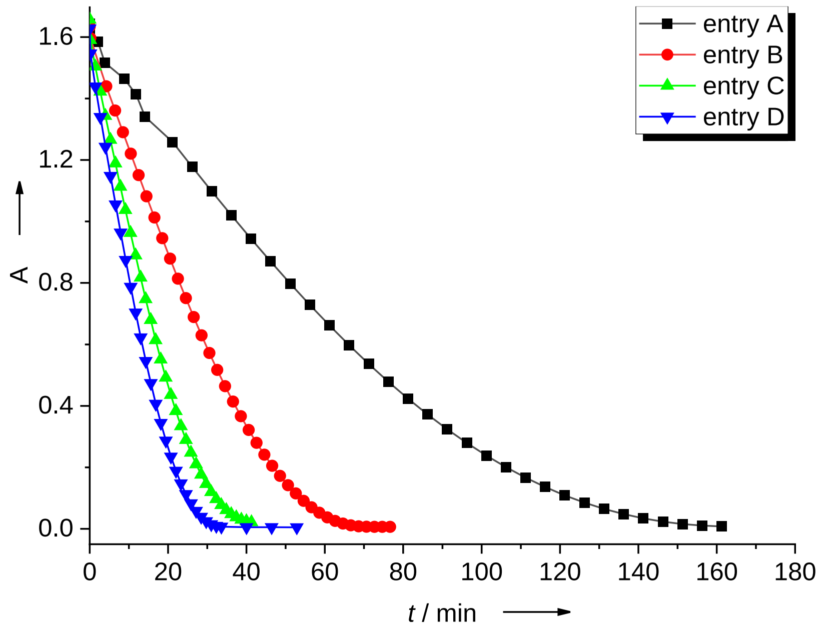

Second-order rate constant k obtained as the slope of the plot k_{obs} versus the hydroquinone concentration

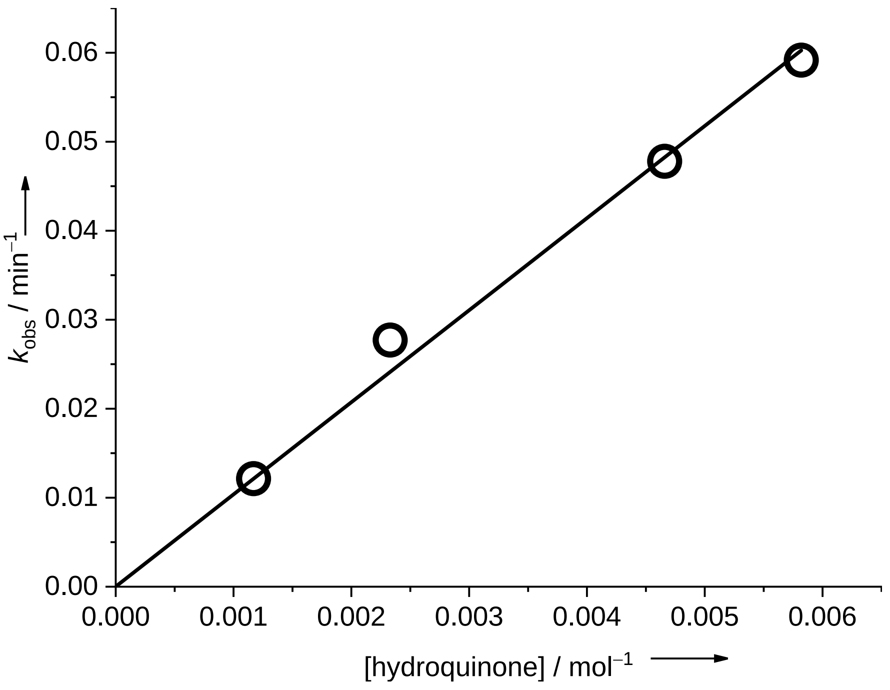

2-Chlorobenzene-1,4-diol oxidation

With $TGFA3(PF_6)_2$

Solution I: 1.420 mg of $TGFA3(PF_6)_2$ was dissolved in 25 ml CH_3CN, c = $6.87{\cdot}10^{-5}$ M.

Solution II: 27.008 mg of 2-chlorobenzene-1,4-diol was dissolved in 5 ml CH_3CN, 2 ml of this solution was diluted to 5 ml c = $1.31{\cdot}10^{-2}$ M.

	entry A	entry B	entry C	entry D
solution I [ml]	2.70	2.70	2.70	2.70
solution II [ml]	0.12	0.25	0.38	0.5
CH_3CN [ml]	0.39	0.25	0.12	-
$TGFA3(PF_6)_2$ [c, cuvette]	$5.81{\cdot}10^{-5}$ M	$5.81{\cdot}10^{-5}$ M	$5.81{\cdot}10^{-5}$ M	$5.81{\cdot}10^{-5}$ M
2-chlorobenzene-1,4-diol [c, cuvette]	$4.91{\cdot}10^{-4}$ M	$1.02{\cdot}10^{-3}$ M	$1.55{\cdot}10^{-3}$ M	$2.04{\cdot}10^{-3}$ M
equivalents of 2-chlorobenzene-1,4-diol	8.4	17.6	26.7	35.2

UV-vis spectra recorded for the reaction of $TGFA3(PF_6)_2$ with 8.4 equivalents of 2-chlorobenzene-1,4-diol in CH_3CN solution to give $(TGFA3{+}2H)(PF_6)_2$ and 2-chloro-1,4-benzoquinone

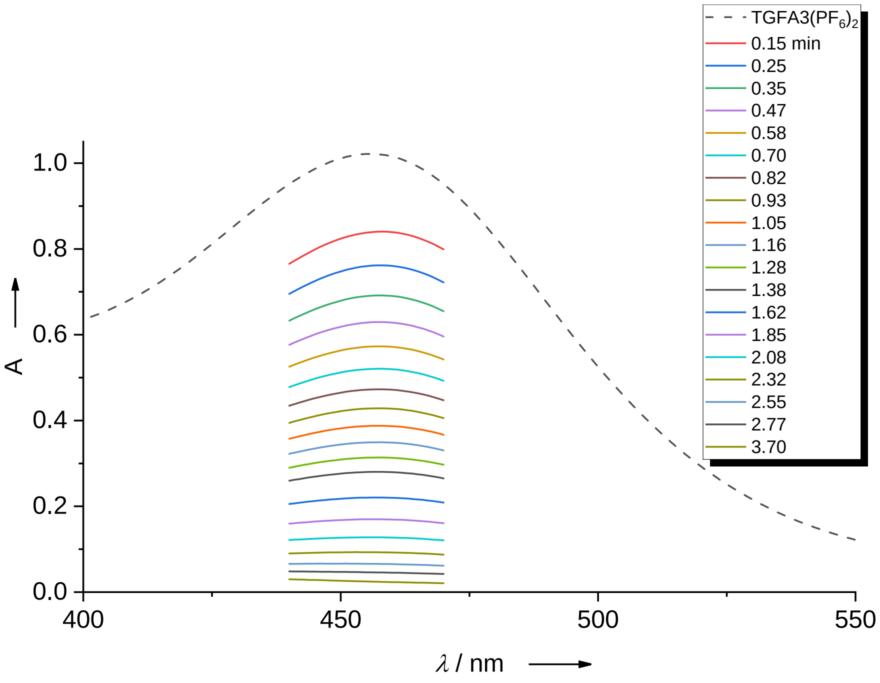

UV-vis spectra recorded for the reaction of TGFA3(PF_6)$_2$ with 35.2 equivalents of 2-chlorobenzene-1,4-diol in CH_3CN solution to give (TGFA3+2H)(PF_6)$_2$ and 2-chloro-1,4-benzoquinone

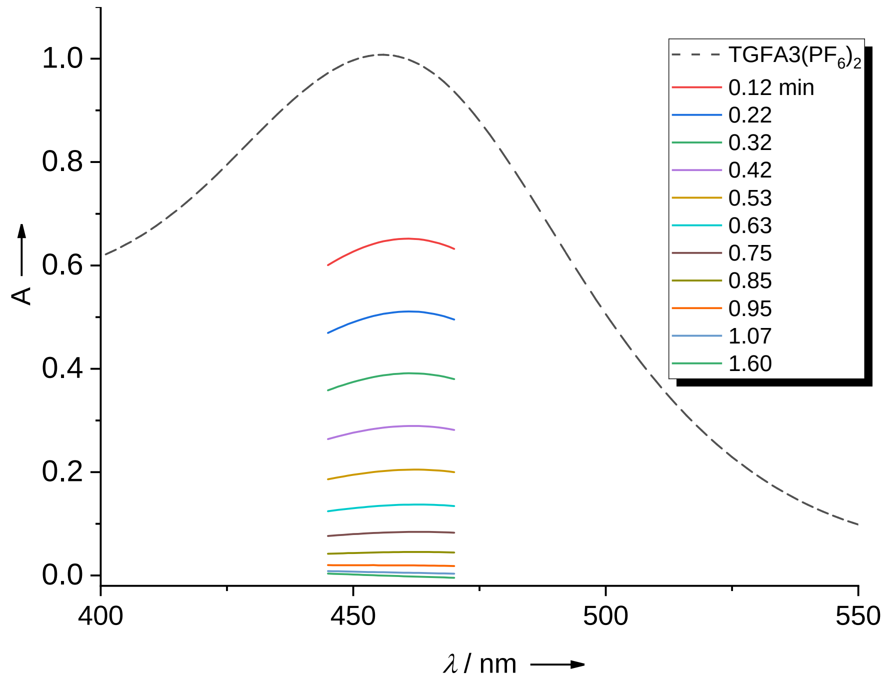

Plot of the absorption at 456 nm (band of the reactant (TGFA3)$^{2+}$) in dependence of the reaction time

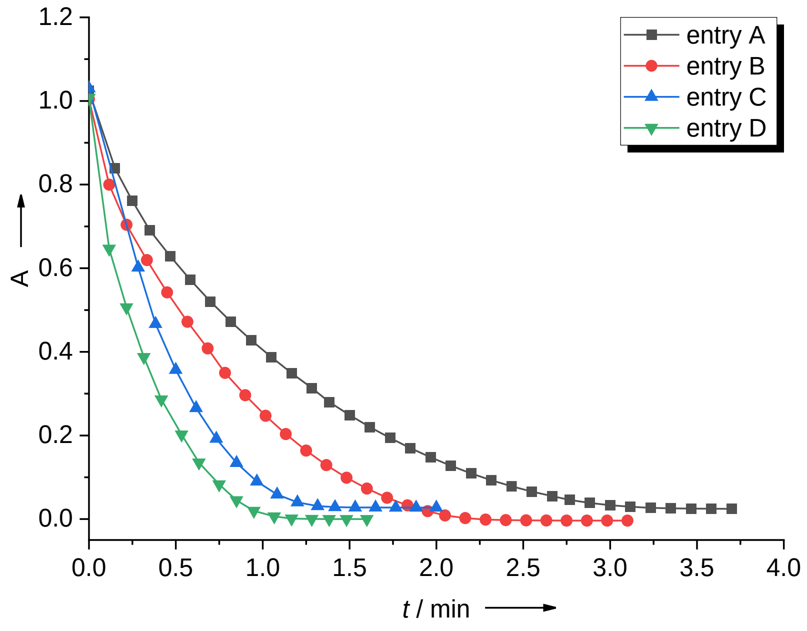

second-order rate constant k obtained as the slope of the plot k_{obs} versus the 2-chlorobenzene-1,4-diol concentration

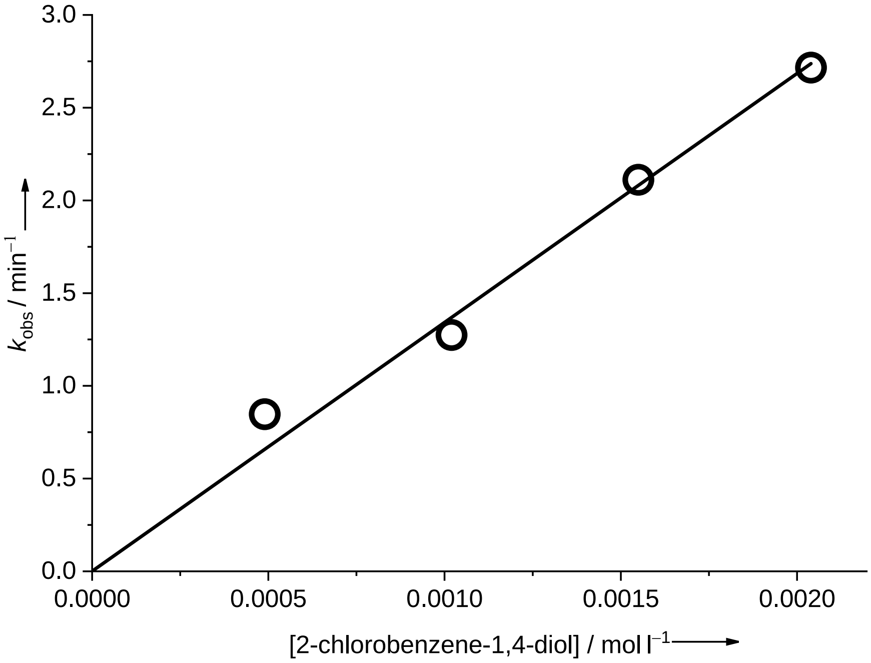

With GFA3a(PF_6)$_2$

Solution I: 1.496 mg of GFA3a(PF_6)$_2$ was dissolved in 25 ml CH_3CN, c = 7.29·10^{-5} M.

Solution II: 20.524 mg of 2-chlorobenzene-1,4-diol was dissolved in 5 ml CH_3CN, c = 3.27·10^{-2} M.

	entry A	entry B	entry C	entry D
solution I [ml]	2.55	2.55	2.55	2.55
solution II [ml]	0.1	0.2	0.4	0.5
CH_3CN [ml]	0.55	0.45	0.25	0.15
GFA3a(PF_6)$_2$ [c, cuvette]	5.81·10^{-5} M	5.81·10^{-5} M	5.81·10^{-5} M	5.81·10^{-5} M
2-chlorobenzene-1,4-diol [c, cuvette]	1.02·10^{-3} M	2.04·10^{-3} M	4.09·10^{-3} M	5.11·10^{-3} M
equivalents of 2-chlorobenzene-1,4-diol	17.6	35.2	70.3	87.9

UV-vis spectra recorded for the reaction of $GFA3a(PF_6)_2$ with 35.2 equivalents of 2-chlorobenzene-1,4-diol in CH_3CN solution to give $(GFA3a+2H)(PF_6)_2$ and 2-chloro-1,4-benzoquinone

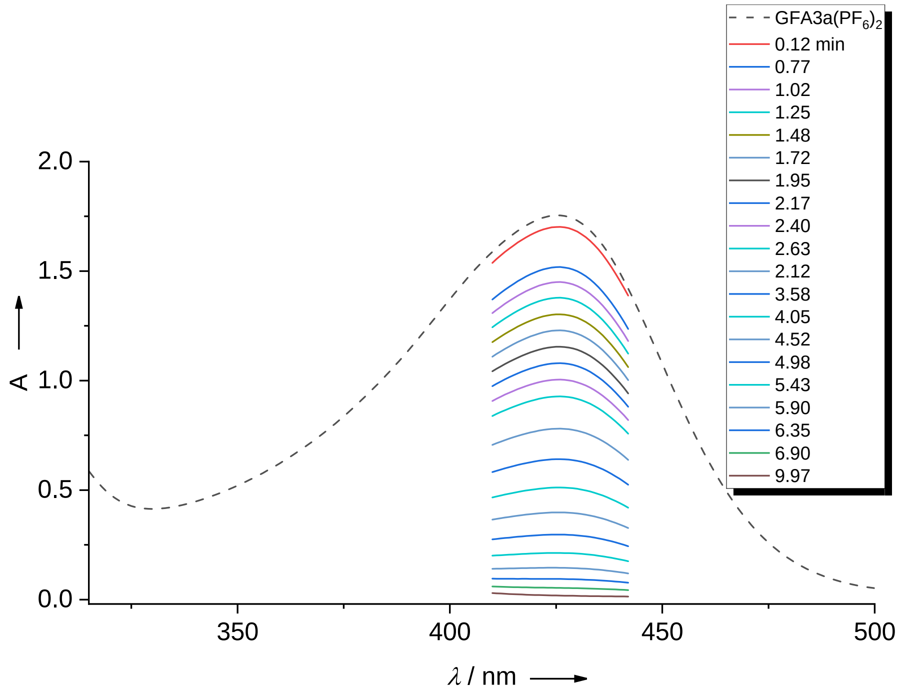

UV-vis spectra recorded for the reaction of $GFA3a(PF_6)_2$ with 87.9 equivalents of 2-chlorobenzene-1,4-diol in CH_3CN solution to give $(GFA3a+2H)(PF_6)_2$ and 2-chloro-1,4-benzoquinone

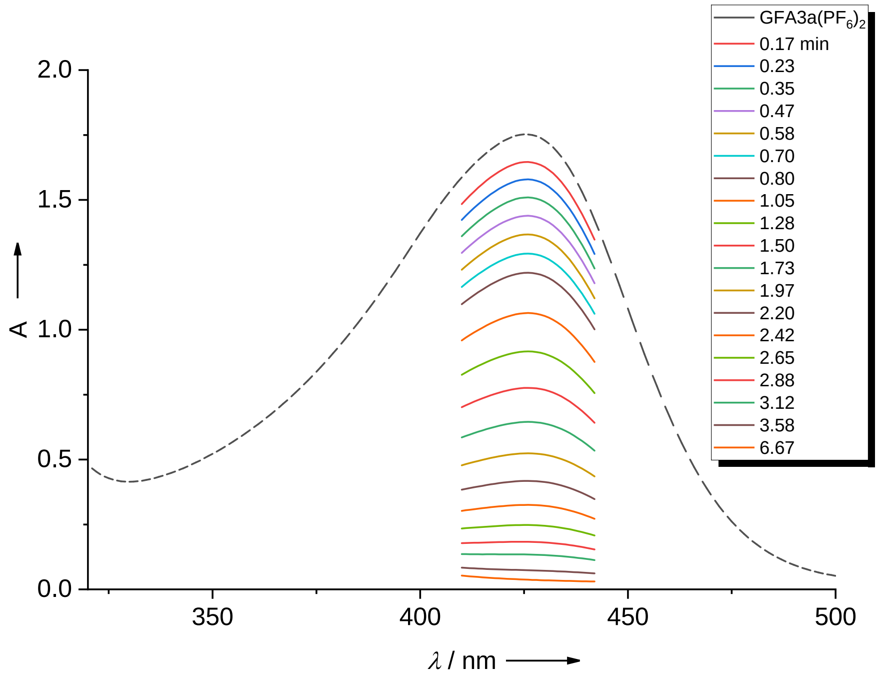

Plot of the absorption at 425 nm (band of the reactant $(GFA3a)^{2+}$) in dependence of the reaction time

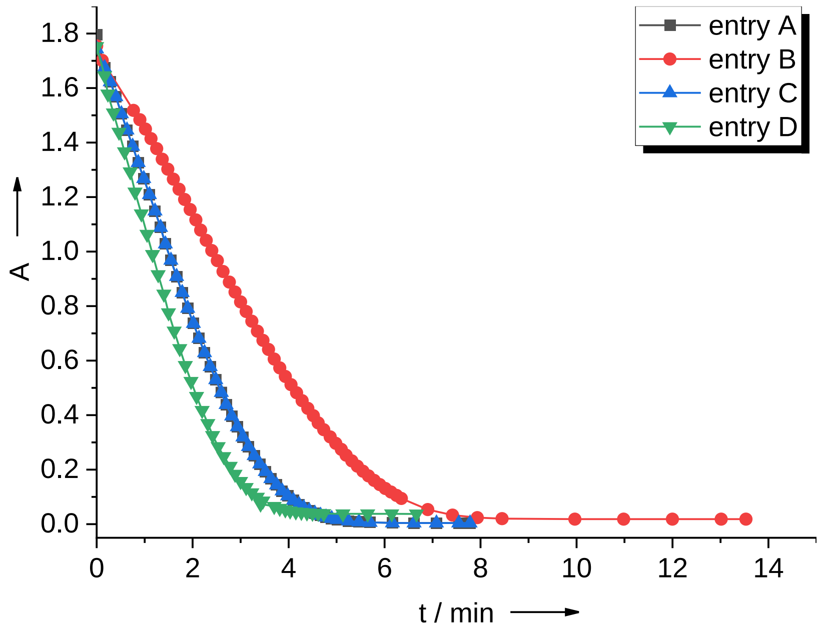

Second-order rate constant k obtained as the slope of the plot k_{obs} versus the 2-chlorobenzene-1,4-diol concentration

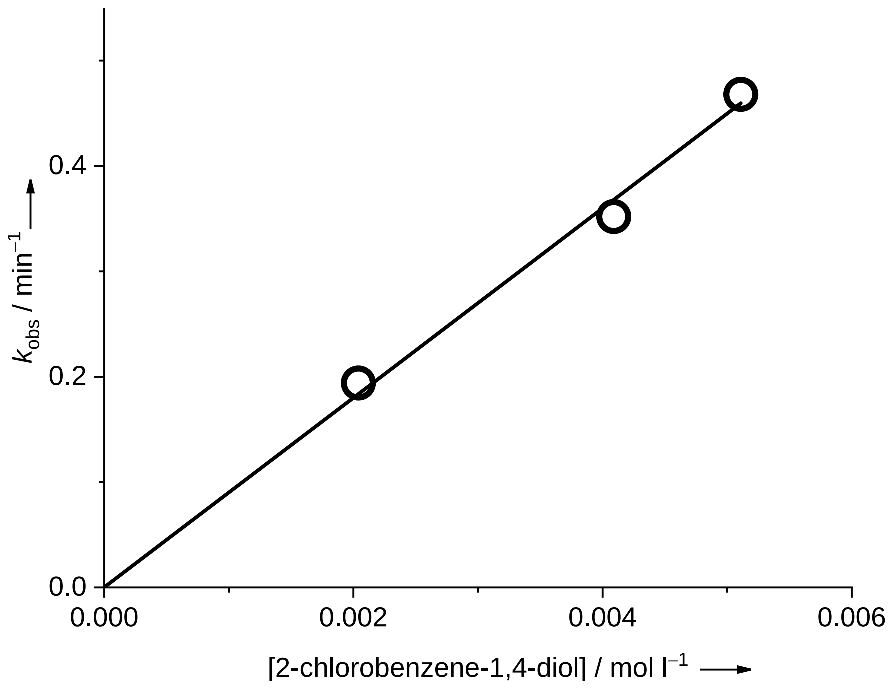

2-Bromobenzene-1,4-diol oxidation

With TGFA3(PF_6)$_2$

Solution I: 1.420 mg of TGFA3(PF_6)$_2$ was dissolved in 25 ml CH_3CN, c = $6.87 \cdot 10^{-5}$ M.

Solution Ib: 0.304 mg of TGFA3(PF_6)$_2$ was dissolved in 5 ml CH_3CN, c = $7.37 \cdot 10^{-5}$ M.

Solution II: 35.202 mg of 2-bromobenzene-1,4-diol was dissolved in 5 ml CH_3CN, 2 ml of this solution was diluted to 5 ml, c = $1.40 \cdot 10^{-2}$ M.

	entry A	entry B	entry C	entry D
solution I [ml]	2.70	2.70	2.70	-
solution Ib [ml]	-	-	-	2.53
solution II [ml]	0.12	0.25	0.38	0.5
CH_3CN [ml]	0.38	0.25	0.12	0.17
TGFA3(PF_6)$_2$ [c, cuvette]	$5.81 \cdot 10^{-5}$ M	$5.81 \cdot 10^{-5}$ M	$5.81 \cdot 10^{-5}$ M	$5.83 \cdot 10^{-5}$ M
2-bromobenzene-1,4-diol [c, cuvette]	$5.25 \cdot 10^{-4}$ M	$1.09 \cdot 10^{-3}$ M	$1.66 \cdot 10^{-3}$ M	$2.19 \cdot 10^{-3}$ M
equivalents of 2-bromobenzene-1,4-diol	9.0	18.8	28.6	37.5

UV-vis spectra recorded for the reaction of (TGFA3)(PF_6)$_2$ with 9.0 equivalents of 2-bromobenzene-1,4-diol in CH_3CN solution to give (TGFA3+2H)(PF_6)$_2$ and 2-bromo-1,4-benzoquinone

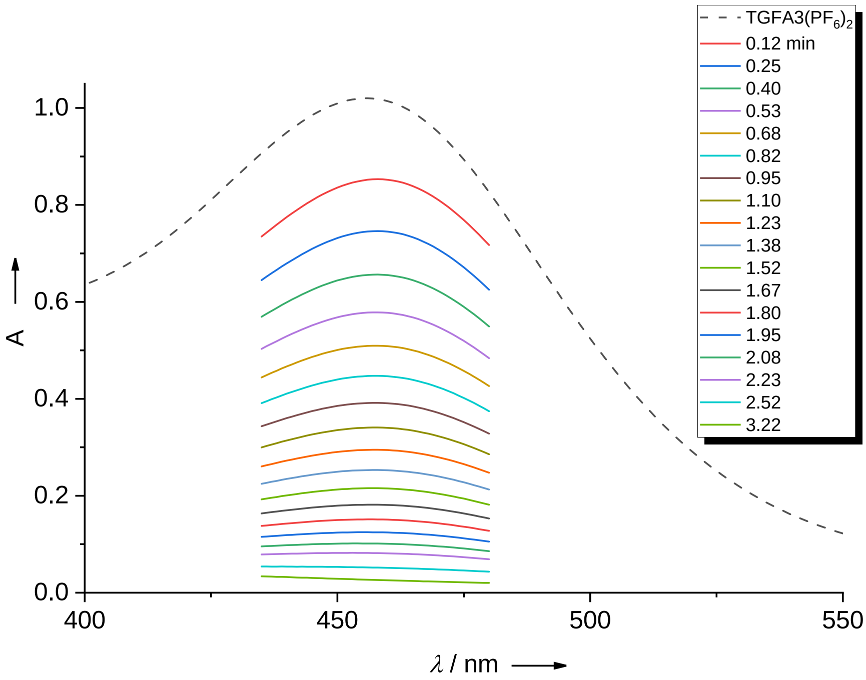

UV-vis spectra recorded for the reaction of $(TGFA3)(PF_6)_2$ with 37.5 equivalents of 2-bromobenzene-1,4-diol in CH_3CN solution to give $(TGFA3+2H)(PF_6)_2$ and 2-bromo-1,4-benzoquinone

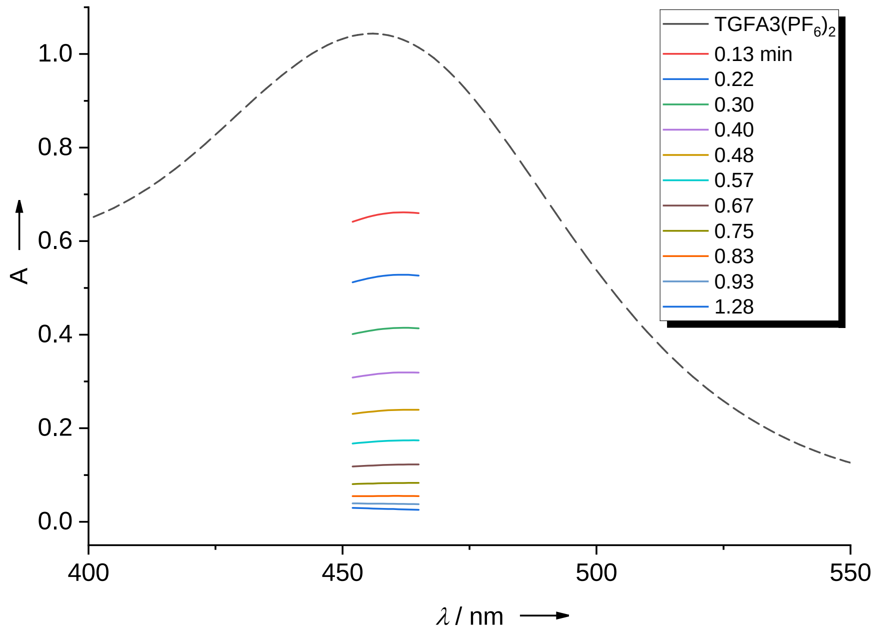

Plot of the absorption at 456 nm (band of the reactant $(TGFA3)^{2+}$) in dependence of the reaction time

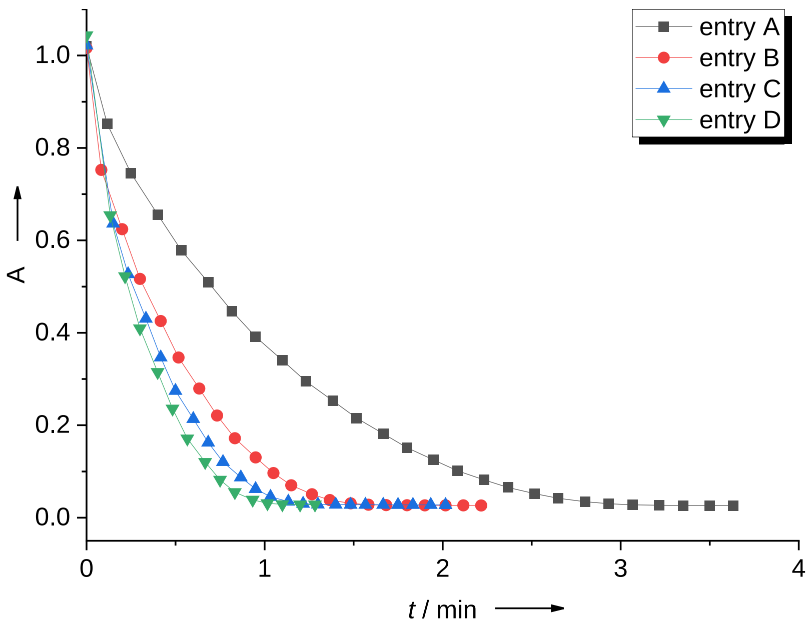

Second-order rate constant k obtained as the slope of the plot k_{obs} versus the 2-bromobenzene-1,4-diol concentration

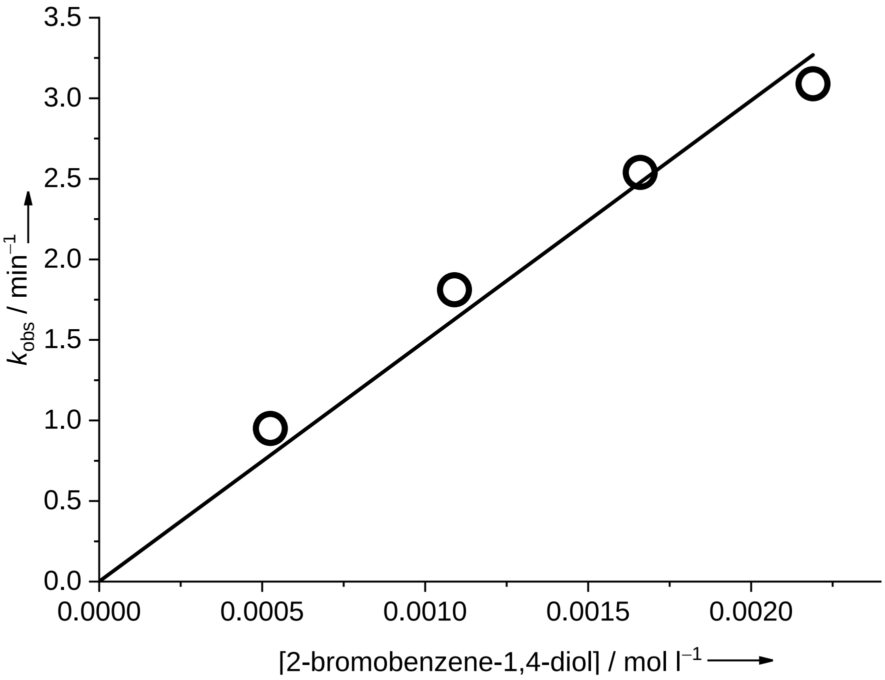

With GFA3a(PF_6)$_2$

Solution I: 1.496 mg of GFA3a(PF_6)$_2$ was dissolved in 25 ml CH_3CN, c = 7.29·10^{-5} M.

Solution II: 35.202 mg of 2-bromobenzene-1,4-diol was dissolved in 5 ml CH_3CN, c = 3.50·10^{-2} M.

	entry A	entry B	entry C	entry D
solution I [ml]	2.55	2.55	2.55	2.55
solution II [ml]	0.1	0.2	0.4	0.5
CH_3CN [ml]	0.55	0.45	0.25	0.15
GFA3a(PF_6)$_2$ [c, cuvette]	5.81·10^{-5} M	5.81·10^{-5} M	5.81·10^{-5} M	5.81·10^{-5} M
2-bromobenzene-1,4-diol [c, cuvette]	1.09·10^{-3} M	2.19·10^{-3} M	4.38·10^{-3} M	5.47·10^{-3} M
equivalents of 2-bromobenzene-1,4-diol	18.8	37.7	75.3	94.2

UV-vis spectra recorded for the reaction of GFA3a(PF_6)$_2$ with 18.8 equivalents of 2-bromobenzene-1,4-diol in CH_3CN solution to give (GFA3a+2H)(PF_6)$_2$ and 2-bromo-1,4-benzoquinone

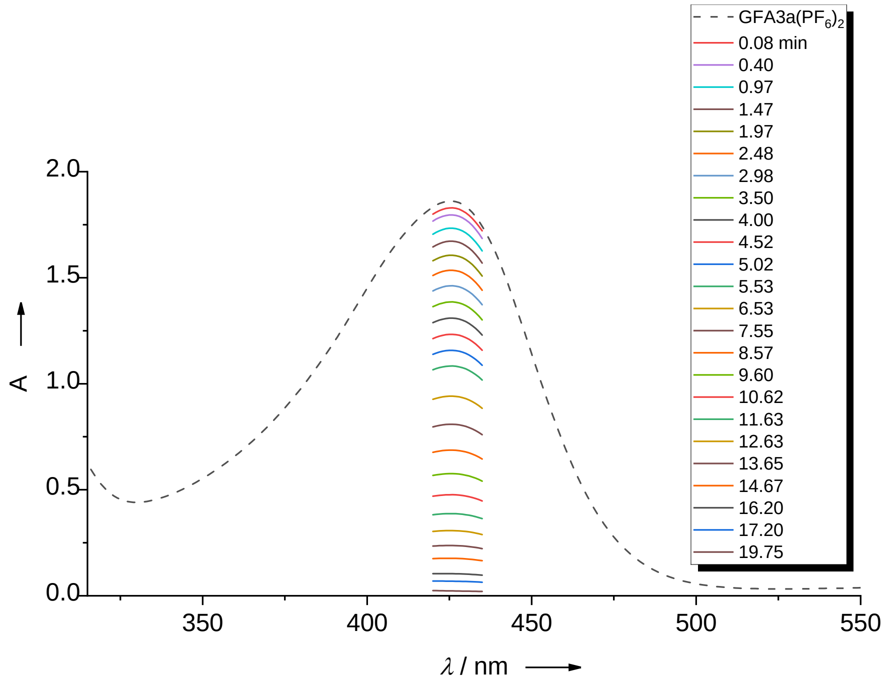

UV-vis spectra recorded for the reaction of GFA3a(PF_6)$_2$ with 94.2 equivalents of 2-bromobenzene-1,4-diol in CH_3CN solution to give (GFA3a+2H)(PF_6)$_2$ and 2-bromo-1,4-benzoquinone

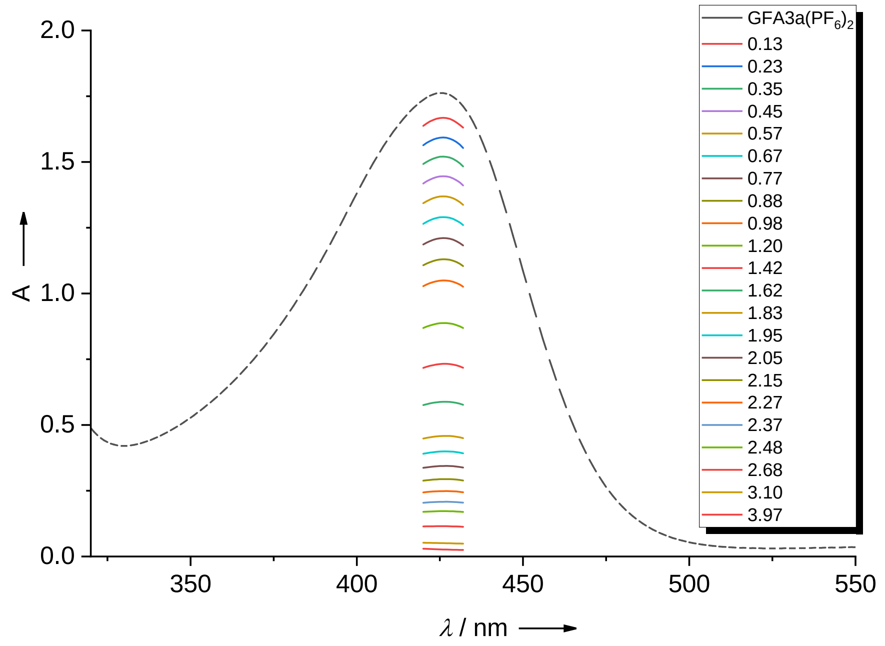

Plot of the absorption at 425 nm (band of the reactant $(GFA3a)^{2+}$) in dependence of the reaction time

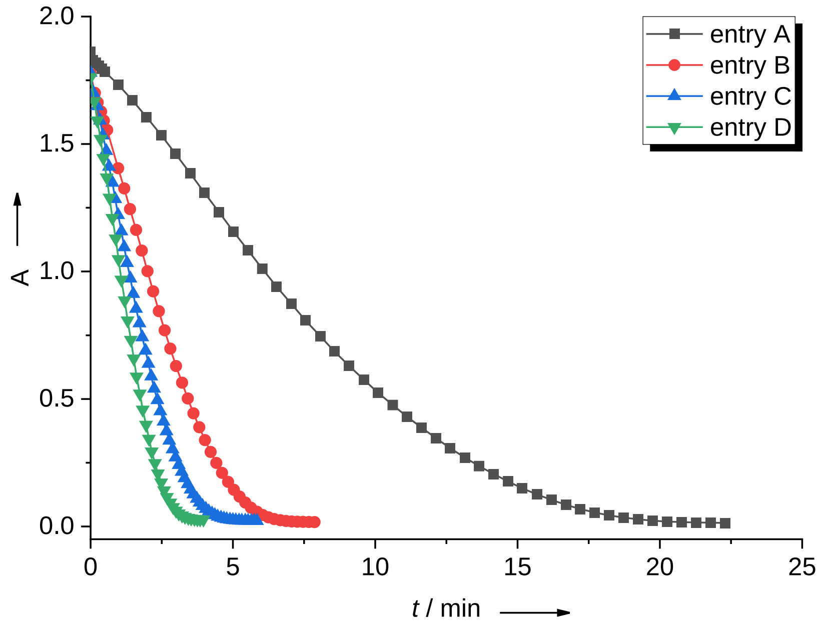

Second-order rate constant k obtained as the slope of the plot k_{obs} versus the 2-bromobenzene-1,4-diol concentration

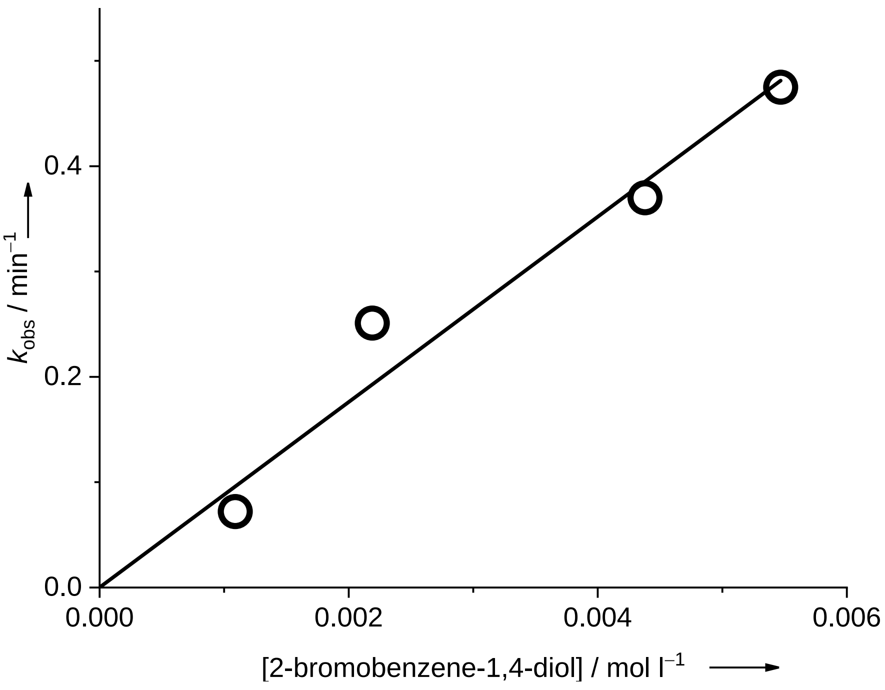

2,5-Dibromobenzene-1,4-diol oxidation

With TGFA3(PF_6)$_2$

Stoichiometric ratio

A solution of 0.542 mg TGFA3(PF_6)$_2$ in 5 ml CH_3CN (c = $1.31 \cdot 10^{-4}$ M) and a solution of 0.94 mg 2,5-dibromobenzene-1,4-diol in 25 ml CH_3CN (c = $1.36 \cdot 10^{-4}$ M) were prepared. Under argon atmosphere a 1 cm quartz glass cuvette was filled with 1.5 ml (c = $6.30 \cdot 10^{-5}$ M [cuvette]) of the TGFA3(PF_6)$_2$ solution and with 1.45 ml (c = $6.31 \cdot 10^{-5}$ M [cuvette]) of the 2,5-dibromobenzene-1,4-diol solution and 0.18 ml of CH_3CN.

UV-vis spectra for a 1:1 mixture of TGFA3(PF_6)$_2$ and 2,5-dibromobenzene-1,4-diol in CH_3CN

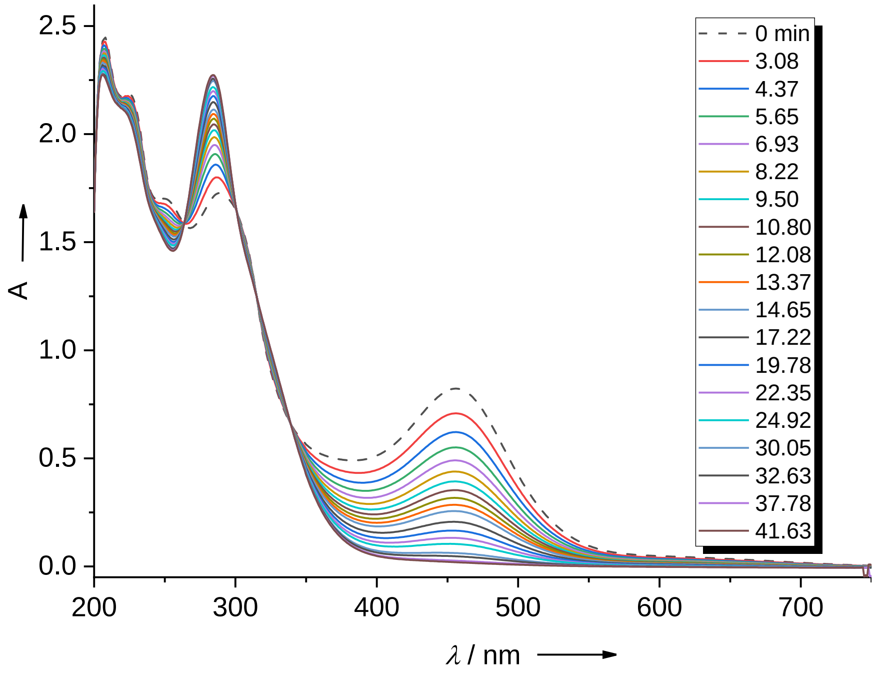

General protocol for the UV-vis kinetic study

Solution I: 1.476 mg of TGFA3(PF_6)$_2$ was dissolved in 25 ml CH_3CN, c = $7.16 \cdot 10^{-5}$ M.

Solution II: 49.606 mg of 2,5-dibromobenzene-1,4-diol was dissolved in 5 ml CH_3CN, 2 ml of this solution was diluted to 5 ml, c = $1.44 \cdot 10^{-2}$ M.

	entry A	entry B	entry C	entry D
solution I [ml]	2.60	2.60	2.60	2.60
solution II [ml]	0.13	0.25	0.38	0.5
CH_3CN [ml]	0.47	0.35	0.22	0.1
TGFA3(PF_6)$_2$ [c, cuvette]	$5.82 \cdot 10^{-5}$ M	$5.82 \cdot 10^{-5}$ M	$5.82 \cdot 10^{-5}$ M	$5.82 \cdot 10^{-5}$ M
2,5-dibromobenzene-1,4-diol [c, cuvette]	$5.84 \cdot 10^{-4}$ M	$1.12 \cdot 10^{-3}$ M	$1.71 \cdot 10^{-3}$ M	$2.25 \cdot 10^{-3}$ M
equivalents of 2,5-dibromobenzene-1,4-diol	10.0	19.3	29.3	38.6

UV-vis spectra recorded for the reaction of TGFA3(PF_6)$_2$ with 10.0 equivalents of 2,5-dibromobenzene-1,4-diol in CH_3CN solution to give (TGFA3+2H)(PF_6)$_2$ and 2,5-dibromo-1,4-benzoquinone

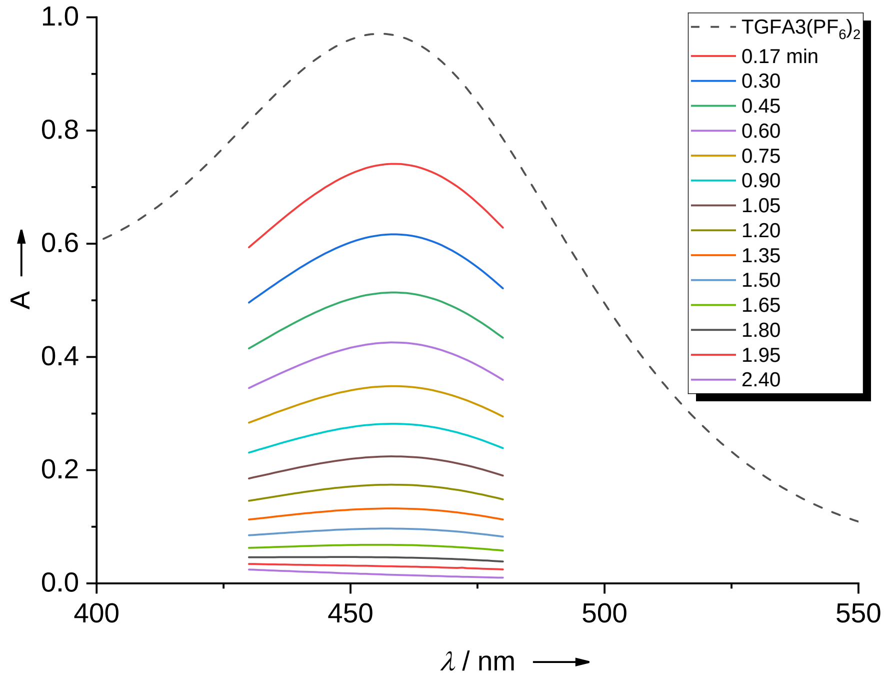

UV-vis spectra recorded for the reaction of TGFA3$(PF_6)_2$ with 38.6 equivalents of 2,5-dibromobenzene-1,4-diol in CH_3CN solution to give (TGFA3+2H)$(PF_6)_2$ and 2,5-dibromo-1,4-benzoquinone

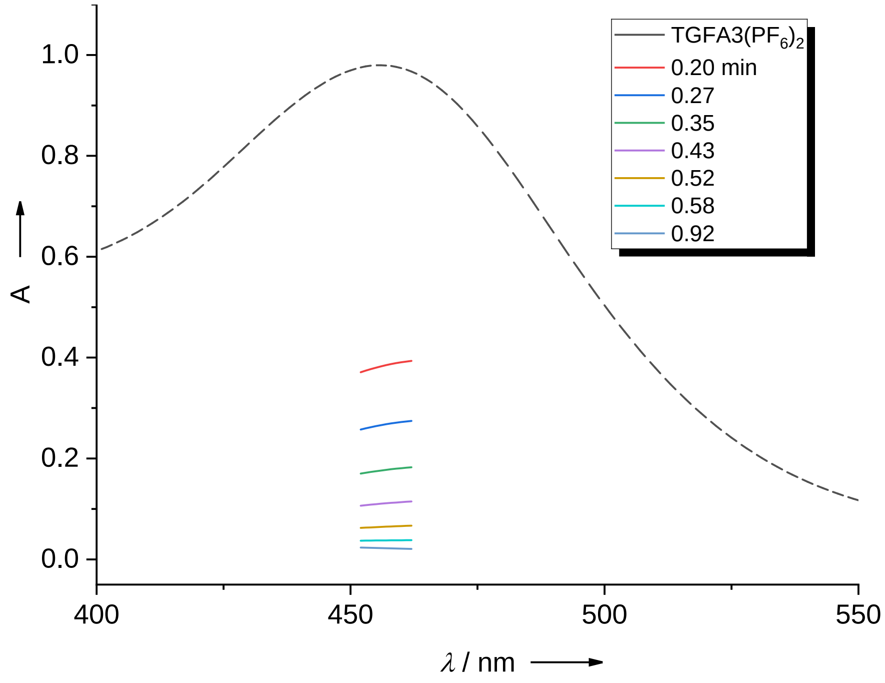

Plot of the absorption at 456 nm (band of the reactant $(TGFA3)^{2+}$ in dependence of the reaction time

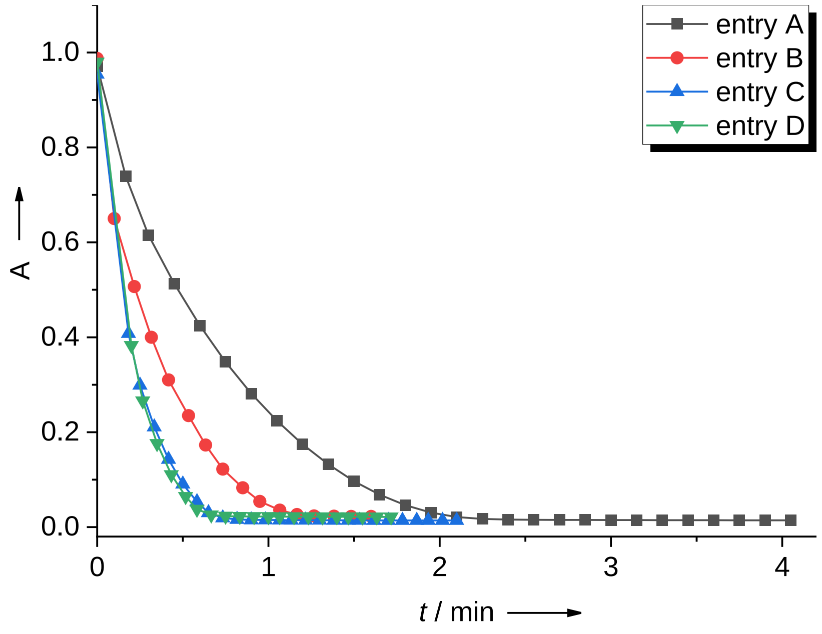

Second-order rate constant *k* obtained as the slope of the plot k_{obs} versus the 2,5-dibromobenzene-1,4-diol concentration

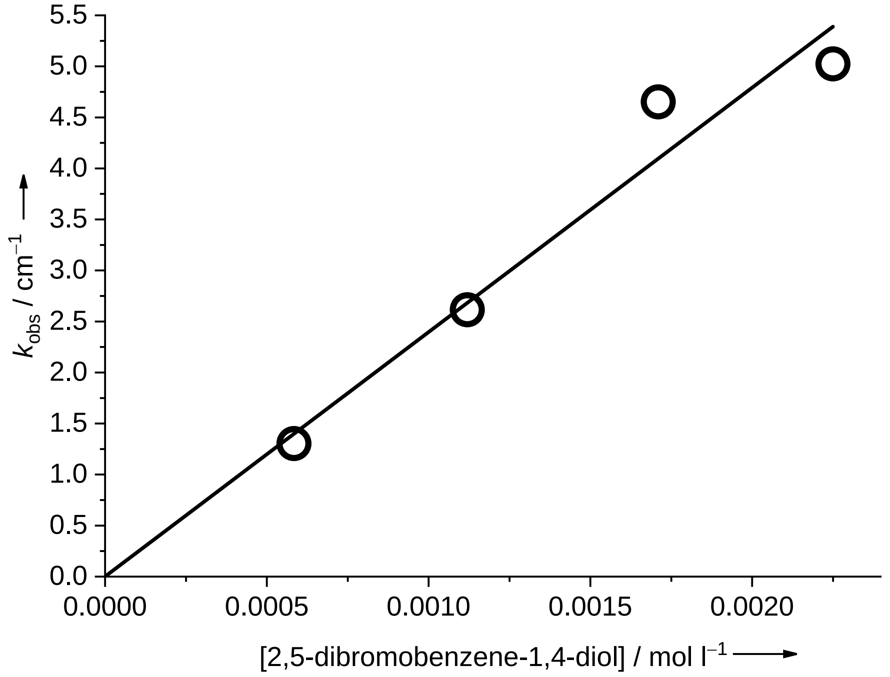

Second-order rate constants *k* obtained as the slope of the plot k_{obs} versus the different hydroquinone concentrations for the reactions with TGFA3$(PF_6)_2$

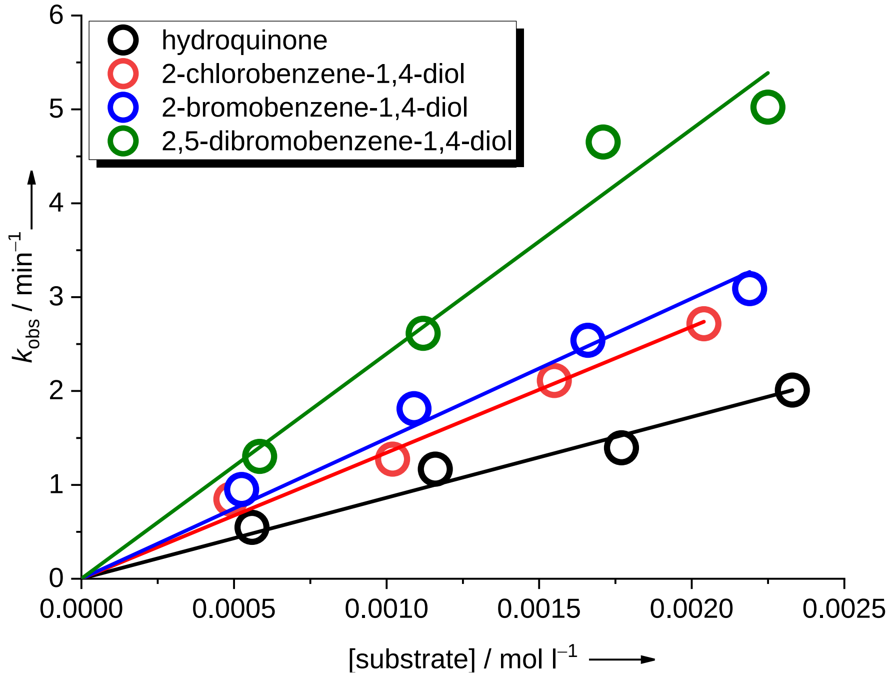

Second-order rate constants k obtained as the slope of the plot k_{obs} versus the different hydroquinone concentrations for the reactions with GFA3a$(PF_6)_2$

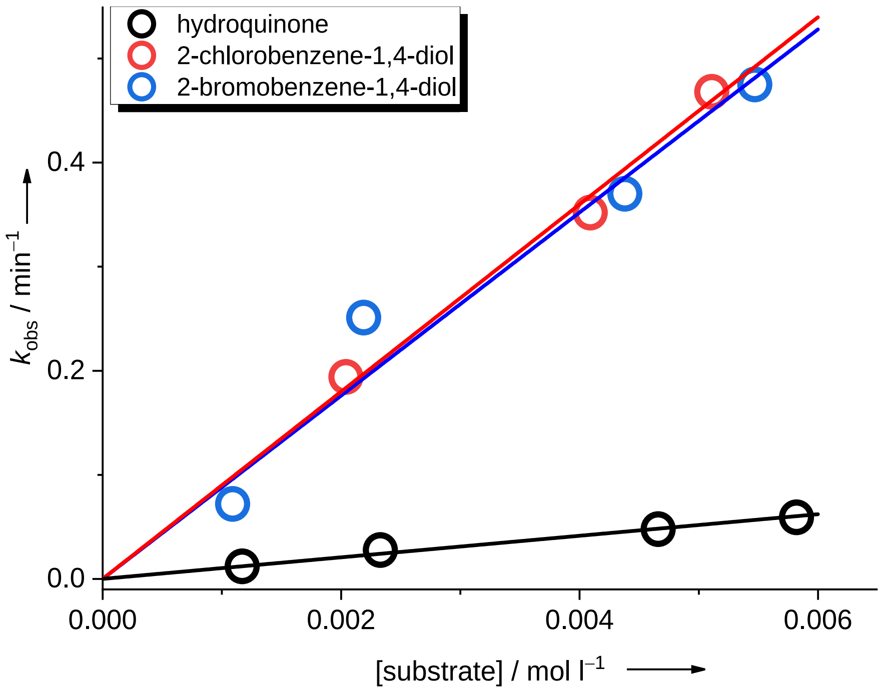

Details for the crystal structure determinations

	TGFA1	(TGFA1+2H)(PF_6)$_2$	TGFA2
CCDC-No.	2505945	2505950	2505946
Empirical formula	$C_{14}H_{18}N_4S_2$	$C_{14}H_{20}F_{12}N_4P_2S_2$	$C_{18}H_{24}N_6S_3$
Formula weight	306.44	598.40	420.61
Temperature/K	100.00	100.00	100.0
Crystal system	triclinic	monoclinic	monoclinic
Space group	P-1	$P2_1/n$	Cc
a/Å	5.8541(4)	5.9395(8)	18.9665(19)
b/Å	9.6007(6)	13.7513(19)	7.7571(7)
c/Å	13.3948(9)	14.021(2)	14.1086(12)
α/°	78.419(3)	90	90
β/°	82.521(3)	100.581(5)	103.030(6)
γ/°	82.257(3)	90	90
Volume/Å^3	726.65(8)	1125.7(3)	2022.3(3)
Z	2	2	4
ρ_{calc} g/cm^3	1.401	1.765	1.381
μ/mm^{-1}	0.362	0.489	0.383
F(000)	324.0	604.0	888.0
Crystal size/mm^3	0.307 × 0.205 × 0.116	0.163 × 0.13 × 0.09	0.242 × 0.238 × 0.223
Radiation	MoKα (λ = 0.71073)	MoKα (λ = 0.71073)	MoKα (λ = 0.71073)
2Θ range for data collection/°	4.358 to 54.462	4.184 to 53.946	4.408 to 55.25
Index ranges	-7 ≤ h ≤ 7, -12 ≤ k ≤ 12, -17 ≤ l ≤ 17	-7 ≤ h ≤ 7, -14 ≤ k ≤ 17, -17 ≤ l ≤ 17	-24 ≤ h ≤ 24, -10 ≤ k ≤ 10, -18 ≤ l ≤ 18
Reflections collected	19326	35262	43987
Independent reflections	3203 [R_{int} = 0.0668, R_{sigma} = 0.0496]	2454 [R_{int} = 0.0956, R_{sigma} = 0.0363]	4652 [R_{int} = 0.0851, R_{sigma} = 0.0444]
Data/restraints/ parameters	3203/0/192	2454/0/159	4652/2/248
Goodness-of-fit on F^2	1.029	1.058	1.090
Final R indexes [I>=2σ (I)]	R_1 = 0.0492, wR_2 = 0.1189	R_1 = 0.0389, wR_2 = 0.0964	R_1 = 0.0618, wR_2 = 0.1600
Final R indexes [all data]	R_1 = 0.0684, wR_2 = 0.1296	R_1 = 0.0491, wR_2 = 0.1013	R_1 = 0.0635, wR_2 = 0.1615
Largest diff. peak/hole / e Å^{-3}	0.61/-0.34	0.37/-0.38	1.42/-0.36
Flack parameter			0.41(15)

	**{[AgCH$_3$CN(TGFA2)](PF$_6$)}*$_n$***	**TGFA3**
CCDC-No.	2505949	2505947
Empirical formula	$C_{21.2}H_{28.8}AgF_6N_{7.6}PS_3$	$C_{22}H_{30}N_8S_4$
Formula weight	739.14	534.78
Temperature/K	100.00	100.00
Crystal system	monoclinic	monoclinic
Space group	$P2_1/c$	$P2_1/n$
a/Å	12.6849(16)	9.0778(15)
b/Å	7.9606(9)	12.547(2)
c/Å	29.205(3)	11.2877(18)
α/°	90	90
β/°	96.372(4)	98.670(6)
γ/°	90	90
Volume/Å^3	2930.9(6)	1271.0(4)
Z	4	2
ρ_{calc} g/cm^3	1.675	1.397
μ/mm^{-1}	1.022	0.402
F(000)	1493.0	564.0
Crystal size/mm^3	0.26 × 0.18 × 0.08	0.269 × 0.106 × 0.089
Radiation	MoKα (λ = 0.71073)	MoKα (λ = 0.71073)
2Θ range for data collection/°	4.038 to 54	4.886 to 54.516
Index ranges	$-16 \le h \le 16$, $-10 \le k \le 10$, $-37 \le l \le 37$	$-11 \le h \le 11$, $-16 \le k \le 16$, $-14 \le l \le 14$
Reflections collected	116779	31965
Independent reflections	6382 [R_{int} = 0.0728, R_{sigma} = 0.0237]	2840 [R_{int} = 0.1057, R_{sigma} = 0.0518]
Data/restraints/ parameters	6382/226/431	2840/0/156
Goodness-of-fit on F^2	1.058	1.097
Final R indexes [I>=2σ (I)]	R_1 = 0.0505, wR_2 = 0.1142	R_1 = 0.0552, wR_2 = 0.1391
Final R indexes [all data]	R_1 = 0.0686, wR_2 = 0.1319	R_1 = 0.0727, wR_2 = 0.1539
Largest diff. peak/hole / e Å^{-3}	0.83/-1.90	0.72/-0.42

	**(TGFA3+2H)(PF_6)$_2$**	**TGFA3(PF_6)$_2$**	**(TGFA3+2H)(BF_4)$_4$**
CCDC-No.	2505951	2505948	2505952
Empirical formula	$C_{26}H_{38}F_{12}N_{10}P_2S_4$	$C_{24}H_{33}F_{12}N_9P_2S_4$	$C_{22}H_{32}B_4N_8F_{16}S_4$
Formula weight	908.84	865.77	884.03
Temperature/K	100.00	100.00	105.00
Crystal system	orthorhombic	triclinic	monoclinic
Space group	$Pca2_1$	P-1	$P2_1/c$
a/Å	14.7970(9)	6.1409(6)	8.1246(5)
b/Å	21.0194(14)	15.4290(18)	11.0618(6)
c/Å	11.9428(8)	19.232(2)	19.0550(10)
α/°	90	96.945(6)	90
β/°	90	99.124(4)	96.487(2)
γ/°	90	101.234(5)	90
Volume/Å^3	3714.5(4)	1742.8(3)	1701.56(17)
Z	4	2	2
ρ_{calc} g/cm^3	1.625	1.650	1.725
μ/mm^{-1}	0.440	0.463	0.401
F(000)	1864.0	884.0	896.0
Crystal size/mm^3	0.33 × 0.11 × 0.07	0.28 × 0.095 × 0.065	0.15 × 0.14 × 0.068
Radiation	MoKα (λ = 0.71073)	MoKα (λ = 0.71073)	MoKα (λ = 0.71073)
2Θ range for data collection/°	3.876 to 52.996	4.344 to 54	4.264 to 52.998
Index ranges	-18 ≤ h ≤ 18, -26 ≤ k ≤ 26, -14 ≤ l ≤ 14	-7 ≤ h ≤ 7, -19 ≤ k ≤ 19, -24 ≤ l ≤ 24	-10 ≤ h ≤ 10, -13 ≤ k ≤ 13, -23 ≤ l ≤ 23
Reflections collected	83651	121961	40161
Independent reflections	7689 [R_{int} = 0.1167, R_{sigma} = 0.0476]	7606 [R_{int} = 0.0868, R_{sigma} = 0.0358]	3512 [R_{int} = 0.0775, R_{sigma} = 0.0320]
Data/restraints/ parameters	7689/111/537	7606/208/538	3512/1/250
Goodness-of-fit on F^2	1.025	1.089	1.053
Final R indexes [I>=2σ (I)]	R_1 = 0.0406, wR_2 = 0.0902	R_1 = 0.0494, wR_2 = 0.0994	R_1 = 0.0372, wR_2 = 0.0905
Final R indexes [all data]	R_1 = 0.0500, wR_2 = 0.0976	R_1 = 0.0559, wR_2 = 0.1033	R_1 = 0.0484, wR_2 = 0.0975
Largest diff. peak/hole / e Å^{-3}	0.41/-0.32	0.42/-0.54	0.51/-0.33
Flack parameter	0.46(5)		

Computational Details

The density functional calculations were performed with the program TURBOMOLE [15-18]. The B3LYP functional [19,20] was used in connection with the def2-TZVP basis set.[21] For the calculation of the two-electron integrals, the resolution-of-the-identity (RI) approximation [22] was used with the appropriate def2-TZVP auxiliary basis set.[23] The calculations included the D3 dispersion correction with Becke-Johnson damping[24,25]. Harmonic vibrational frequencies [26] were calculated.

Calculated electronic energies and thermodynamic contributions

Electronic energies by density functional calculations with the B3LYP functional and the def2-TZVP basis set (grid "m5") with inclusion of the D3(BJ) dispersion correction.

System	Name	Term	E_{el} / Hartree
GFA3a	tetrakis-tmgb	^1A_g	-1677.564856
$(GFA3a2)^+$	tetrakis-tmgb++	^1A_g	-1677.121648
$(GFA3a+H)^+$	tetrakis-tmgbH+	^1A	-1678.007510
$(GFA3a+2H)^{2+}$	tetrakis-tmgbH2++	^1A_g	-1678.361143
GFA1b	bis-dmegb	^1A	-952.497491
$(GFA1b)^{2+}$	bis-dmegb++	^1A	-951.976550
GFA2b	tris-dmegb	^1A	-1312.650191
$(GFA2b)^{2+}$	tris-dmegb++	^1A	-1312.173764
GFA3b	tetrakis-dmegb	^1A_g	-1672.802689
$(GFA3b)^{2+}$	tetrakis-dmegb++	^1A_g	-1672.367838
$(GFA3b+H)^+$	tetrakis-dmegbH+	^1A	-1673.241836
$(GFA3b+2H)^{2+}$	tetrakis-dmegbH2++	^1A_g	-1673.586190
TGFA1	bis-metgb	^1A	-1559.537274
$(TGFA1)^{2+}$	bis-metgb++	^1A	-1558.978075
TGFA2	tris-metgb	^1A	-2223.205807
$(TGFA2)^{2+}$	tris-metgb++	^1A	-2222.688544
TGFA3	tetrakis-metgb	^1A_g	-2886.873171
$(TGFA3)^{2+}$	tetrakis-metgb++	^1A_g	-2886.392341
$(TGFA3+H)^+$	tetrakis-metgbH+	^1A	-2887.296692
$(TGFA3+2H)^{2+}$	tetrakis-metgbH2++	^1A_g	-2887.633347
p-$C_6H_4O_2$	p-$C_6H_4O_2$	^1A_g	-381.418560
p-$C_6H_4(OH)_2$	p-$C_6H_4(OH)_2$	^1A_g	-382.653910

Thermodynamic contributions by density functional calculations with the B3LYP functional and the def2-TZVP basis set (grid "m5") with inclusion of the D3(BJ) dispersion correction.

System	Name	$E_{vib,0}$ / Hartree	μ_{298} / kJmol^{-1}
GFA3a	tetrakis-tmgb	0.769677	1809.94
$(GFA3a)^{2+}$	tetrakis-tmgb++	0.775483	1820.89
$(GFA3a+H)^{+}$	tetrakis-tmgbH+	0.784946	1851.62
$(GFA3a+2H)^{2+}$	tetrakis-tmgbH2++	0.799781	1892.13
GFA1b	bis-dmegb	0.394292	896.26
$GFA1b^{2+}$	bis-dmegb++	0.397002	897.97
GFA2b	tris-dmegb	0.541229	1253.02
$(GFA2b)^{2+}$	tris-dmegb++	0.544229	1251.57
GFA3b	tetrakis-dmegb	0.687857	1614.51
$(GFA3b)^{2+}$	tetrakis-dmegb++	0.692062	1614.05
$(GFA3b+H)^{+}$	tetrakis-dmegbH+	0.703337	1653.21
$(GFA3b+2H)^{2+}$	tetrakis-dmegbH2++	0.716673	1673.29
TGFA1	bis-metgb	0.307330	673.70
$(TGFA1)^{2+}$	bis-metgb++	0.309398	677.96
TGFA2	tris-metgb	0.410543	917.31
$(TGFA2)^{2+}$	tris-metgb++	0.413282	923.63
TGFA3	tetrakis-metgb	0.514071	1163.72
$(TGFA3)^{2+}$	tetrakis-metgb++	0.516556	1165.00
$(TGFA3+H)^{+}$	tetrakis-metgbH+	0.527886	1195.90
$(TGFA3+2H)^{2+}$	tetrakis-metgbH2++	0.541505	1230.70
$p\text{-}C_6H_4O_2$	$p\text{-}C_6H_4O_2$	0.084947	146.12
$p\text{-}C_6H_4(OH)_2$	$p\text{-}C_6H_4(OH)_2$	0.108160	205.22
H^{+}	H^{+}	0.0	-26.29

Cartesian coordinates of the optimized structures

GFA3a

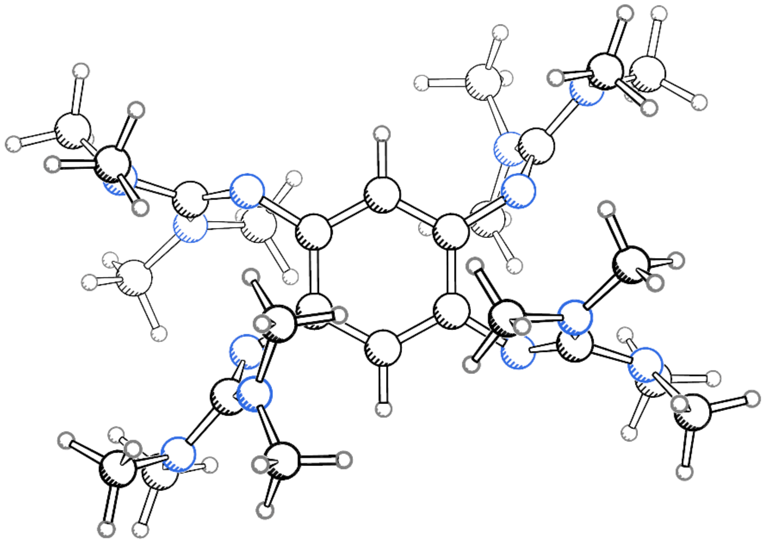

Energy = -1677.564856352

N	1.2956016	-0.4155352	-2.4618073
N	2.1119249	-2.6509474	-2.2035537
N	1.8638244	-1.6807846	-4.3022628
N	-0.9085539	-2.0812335	-1.6665952
N	-2.8544039	-0.9135154	-2.4159874
N	-2.6931588	-3.2302324	-2.5811240
C	0.6839660	-0.2777010	-1.2103302
C	-0.4534977	-1.0287428	-0.8514912
C	-1.0975145	-0.7406397	0.3519779
C	1.7204631	-1.5368016	-2.9257066
C	2.4846481	-2.5351784	-0.8108631
H	2.9101117	-1.5536934	-0.6213242
H	3.2411477	-3.2935826	-0.5881535
H	1.6354546	-2.6806399	-0.1345089
C	1.7129215	-3.9854910	-2.6111897
H	0.9542626	-4.3747839	-1.9261312
H	2.5704832	-4.6672230	-2.6102070
H	1.2864635	-3.9542255	-3.6092796
C	3.1024829	-2.2147406	-4.8448863
H	3.5799244	-2.8673532	-4.1206536
H	3.8048889	-1.4074802	-5.0958930
H	2.9030978	-2.7854039	-5.7553724
C	1.2411320	-0.6782202	-5.1431539
H	0.2402911	-0.4636830	-4.7790360
H	1.1799612	-1.0677664	-6.1619217
H	1.8034004	0.2637059	-5.1581781

C	-2.0848275	-2.0472309	-2.1826976
C	-2.2309173	0.3749474	-2.6438865
H	-2.1600799	0.9774218	-1.7335201
H	-2.8271139	0.9240825	-3.3795923
H	-1.2267038	0.2419760	-3.0369449
C	-4.2435650	-0.8605991	-2.0064841
H	-4.6053200	-1.8616008	-1.7875747
H	-4.8681422	-0.4244055	-2.7933635
H	-4.3582826	-0.2457400	-1.1040906
C	-3.3638788	-3.3108859	-3.8670816
H	-3.6817459	-2.3232455	-4.1864492
H	-4.2405831	-3.9599723	-3.7988809
H	-2.6947487	-3.7234881	-4.6348182
C	-2.1072484	-4.4793082	-2.1415095
H	-1.2790936	-4.7981046	-2.7865424
H	-2.8757476	-5.2559960	-2.1582122
H	-1.7226096	-4.3661771	-1.1318606
N	-1.2956016	0.4155352	2.4618073
N	-2.1119249	2.6509474	2.2035537
N	-1.8638244	1.6807846	4.3022628
N	0.9085539	2.0812335	1.6665952
N	2.8544039	0.9135154	2.4159874
N	2.6931588	3.2302324	2.5811240
C	-0.6839660	0.2777010	1.2103302
C	0.4534977	1.0287428	0.8514912
C	1.0975145	0.7406397	-0.3519779
C	-1.7204631	1.5368016	2.9257066
C	-2.4846481	2.5351784	0.8108631
H	-2.9101117	1.5536934	0.6213242
H	-3.2411477	3.2935826	0.5881535
H	-1.6354546	2.6806399	0.1345089
C	-1.7129215	3.9854910	2.6111897
H	-0.9542626	4.3747839	1.9261312
H	-2.5704832	4.6672230	2.6102070
H	-1.2864635	3.9542255	3.6092796
C	-3.1024829	2.2147406	4.8448863
H	-3.5799244	2.8673532	4.1206536
H	-3.8048889	1.4074802	5.0958930
H	-2.9030978	2.7854039	5.7553724
C	-1.2411320	0.6782202	5.1431539
H	-0.2402911	0.4636830	4.7790360
H	-1.1799612	1.0677664	6.1619217
H	-1.8034004	-0.2637059	5.1581781
C	2.0848275	2.0472309	2.1826976
C	2.2309173	-0.3749474	2.6438865
H	2.1600799	-0.9774218	1.7335201
H	2.8271139	-0.9240825	3.3795923
H	1.2267038	-0.2419760	3.0369449
C	4.2435650	0.8605991	2.0064841
H	4.6053200	1.8616008	1.7875747
H	4.8681422	0.4244055	2.7933635

H	4.3582826	0.2457400	1.1040906
C	3.3638788	3.3108859	3.8670816
H	3.6817459	2.3232455	4.1864492
H	4.2405831	3.9599723	3.7988809
H	2.6947487	3.7234881	4.6348182
C	2.1072484	4.4793082	2.1415095
H	1.2790936	4.7981046	2.7865424
H	2.8757476	5.2559960	2.1582122
H	1.7226096	4.3661771	1.1318606
H	1.9489752	1.3421053	-0.6468831
H	-1.9489752	-1.3421053	0.6468831

$(GFA3a)^{2+}$

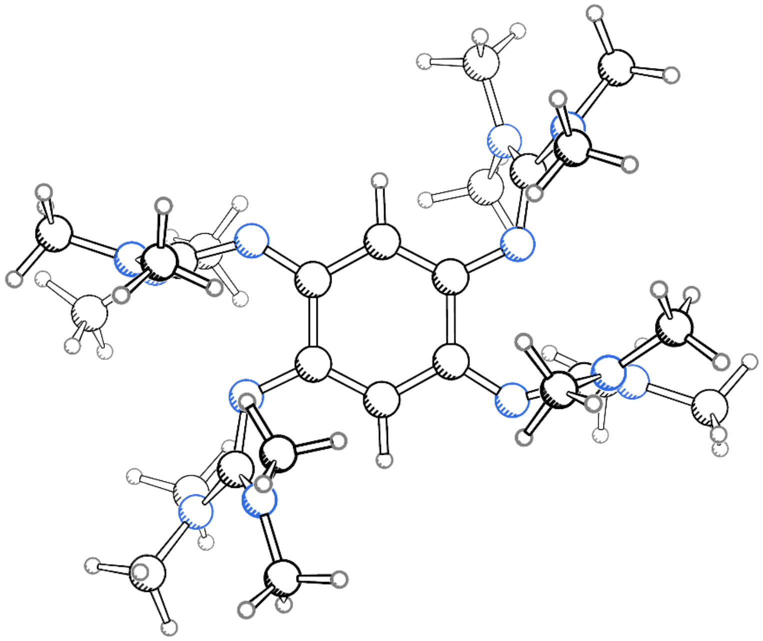

Energy = -1677.121648019

N	1.8737434	-0.8340162	-1.8042643
N	2.0919393	-3.0664964	-2.3952862
N	1.8623340	-1.4292732	-4.0458206
N	-0.7507930	-1.7562699	-2.0235096
N	-2.8626930	-1.0290225	-2.7822138
N	-2.3962452	-3.2946678	-2.5649415
C	0.9702573	-0.4558385	-0.9523356
C	-0.4665392	-0.8998412	-1.0397778
C	-1.3430390	-0.4357965	-0.0816874
C	1.8683590	-1.7870885	-2.7526610
C	2.4193302	-3.3868497	-1.0111820
H	3.0415832	-2.6045731	-0.5854231
H	2.9716036	-4.3249354	-0.9964757
H	1.5176183	-3.4997626	-0.4017036
C	1.6526045	-4.1946872	-3.2127571
H	1.1066538	-4.8855209	-2.5714600

H	2.4912022	-4.7318377	-3.6575796
H	0.9817367	-3.8531309	-3.9950188
C	2.6298826	-2.1335190	-5.0758296
H	3.3228988	-2.8340214	-4.6215290
H	3.2099934	-1.3952224	-5.6302736
H	1.9829599	-2.6610840	-5.7778691
C	1.4176700	-0.0949093	-4.4281013
H	0.7589153	0.3135967	-3.6713624
H	0.8786493	-0.1591248	-5.3735909
H	2.2699846	0.5775058	-4.5539386
C	-2.0032050	-2.0152776	-2.4248120
C	-2.3893874	0.3054216	-3.1147443
H	-2.4125214	0.9686986	-2.2454155
H	-3.0336539	0.7197256	-3.8900975
H	-1.3745402	0.2550353	-3.4945398
C	-4.3089212	-1.1327013	-2.5926784
H	-4.5574508	-2.0351750	-2.0439086
H	-4.8463194	-1.1266609	-3.5423281
H	-4.6403363	-0.2737655	-2.0050611
C	-3.3390390	-3.7440965	-3.5866022
H	-3.5164930	-2.9544152	-4.3097862
H	-4.2897822	-4.0623911	-3.1557445
H	-2.8993414	-4.5970425	-4.1062316
C	-1.7648194	-4.3597108	-1.7981766
H	-1.2018130	-5.0240385	-2.4562575
H	-2.5342528	-4.9490493	-1.2956165
H	-1.0966563	-3.9344291	-1.0571360
N	-1.8737434	0.8340162	1.8042643
N	-2.0919393	3.0664964	2.3952862
N	-1.8623340	1.4292732	4.0458206
N	0.7507930	1.7562699	2.0235096
N	2.8626930	1.0290225	2.7822138
N	2.3962452	3.2946678	2.5649415
C	-0.9702573	0.4558385	0.9523356
C	0.4665392	0.8998412	1.0397778
C	1.3430390	0.4357965	0.0816874
C	-1.8683590	1.7870885	2.7526610
C	-2.4193302	3.3868497	1.0111820
H	-3.0415832	2.6045731	0.5854231
H	-2.9716036	4.3249354	0.9964757
H	-1.5176183	3.4997626	0.4017036
C	-1.6526045	4.1946872	3.2127571
H	-1.1066538	4.8855209	2.5714600
H	-2.4912022	4.7318377	3.6575796
H	-0.9817367	3.8531309	3.9950188
C	-2.6298826	2.1335190	5.0758296
H	-3.3228988	2.8340214	4.6215290
H	-3.2099934	1.3952224	5.6302736
H	-1.9829599	2.6610840	5.7778691
C	-1.4176700	0.0949093	4.4281013
H	-0.7589153	-0.3135967	3.6713624

H	-0.8786493	0.1591248	5.3735909
H	-2.2699846	-0.5775058	4.5539386
C	2.0032050	2.0152776	2.4248120
C	2.3893874	-0.3054216	3.1147443
H	2.4125214	-0.9686986	2.2454155
H	3.0336539	-0.7197256	3.8900975
H	1.3745402	-0.2550353	3.4945398
C	4.3089212	1.1327013	2.5926784
H	4.5574508	2.0351750	2.0439086
H	4.8463194	1.1266609	3.5423281
H	4.6403363	0.2737655	2.0050611
C	3.3390390	3.7440965	3.5866022
H	3.5164930	2.9544152	4.3097862
H	4.2897822	4.0623911	3.1557445
H	2.8993414	4.5970425	4.1062316
C	1.7648194	4.3597108	1.7981766
H	1.2018130	5.0240385	2.4562575
H	2.5342528	4.9490493	1.2956165
H	1.0966563	3.9344291	1.0571360
H	2.3757784	0.7553876	0.0915299
H	-2.3757784	-0.7553876	-0.0915299

(GFA3a+H)$^{+}$

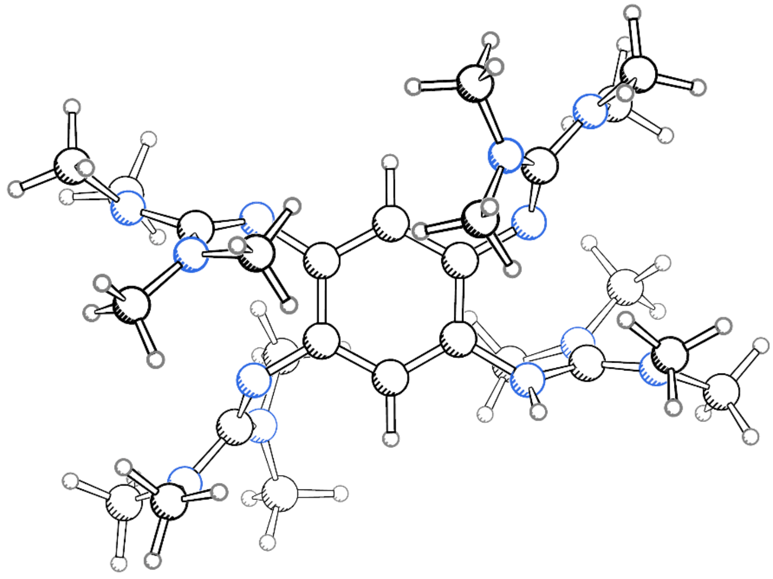

Energy = -1678.007510259

N	1.5687357	-0.3472898	-2.4795288
N	2.2795049	-2.5419816	-2.1434987
N	1.8338052	-1.7647274	-4.2919137
N	-0.7659031	-1.8283937	-2.0066313
N	-2.9109918	-0.8661540	-2.4139229
N	-2.5065194	-3.1179306	-2.8001079

C	0.8142374	-0.1358229	-1.2798637
C	-0.3669075	-0.8606871	-1.0790905
C	-1.0626463	-0.6165997	0.1096225
C	1.8778985	-1.5608093	-2.9616114
C	2.8174956	-2.2649515	-0.8177737
H	3.2727183	-1.2799165	-0.7966520
H	3.5777458	-3.0145457	-0.5963630
H	2.0345952	-2.3095286	-0.0585939
C	1.8957550	-3.9295248	-2.3790663
H	1.3658561	-4.2958489	-1.4993042
H	2.7707540	-4.5584444	-2.5535021
H	1.2205987	-3.9894454	-3.2248049
C	2.7603151	-2.6560816	-4.9792119
H	3.5579707	-2.9548929	-4.3056979
H	3.2003970	-2.1240949	-5.8246124
H	2.2558553	-3.5478677	-5.3576222
C	0.8992468	-1.0324282	-5.1378678
H	0.0539621	-0.7005913	-4.5410426
H	0.5299242	-1.7058101	-5.9123249
H	1.3723224	-0.1772568	-5.6293829
C	-2.0127492	-1.9133251	-2.3724991
C	-2.4616821	0.4982314	-2.6049902
H	-2.3387259	1.0376309	-1.6625074
H	-3.1949470	1.0263186	-3.2192762
H	-1.5065945	0.5050258	-3.1239381
C	-4.2643629	-1.0008143	-1.8990840
H	-4.4704429	-2.0384828	-1.6537281
H	-5.0031347	-0.6565014	-2.6280235
H	-4.3782835	-0.4039124	-0.9873993
C	-3.4694976	-3.2280352	-3.8838051
H	-3.7671668	-2.2405099	-4.2222211
H	-4.3605911	-3.7778112	-3.5701386
H	-3.0217601	-3.7646046	-4.7270280
C	-1.8101651	-4.3433646	-2.4686905
H	-1.1089460	-4.6414285	-3.2574133
H	-2.5384512	-5.1472954	-2.3436332
H	-1.2558054	-4.2078755	-1.5451961
N	-1.3486592	0.4212414	2.2447906
N	-2.0621298	2.6941105	2.1806496
N	-1.8845956	1.5361376	4.1844173
N	0.9147511	2.0994574	1.6771123
N	2.8171605	0.9413079	2.5170363
N	2.6274551	3.2517237	2.7095524
C	-0.6702299	0.3432359	1.0457662
C	0.5177322	1.0917956	0.7997842
C	1.2397672	0.8151448	-0.3573631
C	-1.7254351	1.5213274	2.8179879
C	-2.4027455	2.7010952	0.7757552
H	-2.8650582	1.7556546	0.5046315
H	-3.1216975	3.5033803	0.5935955
H	-1.5302612	2.8658930	0.1334691

C	-1.6857793	3.9889493	2.7227956
H	-0.9603529	4.4655545	2.0589261
H	-2.5550573	4.6459673	2.8218260
H	-1.2233865	3.8629736	3.6967369
C	-3.0493425	2.1579300	4.8002374
H	-3.5671346	2.7854448	4.0823399
H	-3.7478237	1.3895117	5.1505874
H	-2.7568518	2.7687650	5.6575001
C	-1.3137289	0.4481459	4.9559812
H	-0.3236658	0.2067209	4.5812898
H	-1.2341228	0.7651507	5.9970012
H	-1.9276025	-0.4581904	4.9086405
C	2.0655637	2.0765211	2.2644850
C	2.1904724	-0.3476374	2.7172886
H	2.2552732	-0.9845808	1.8291436
H	2.6885917	-0.8620005	3.5443407
H	1.1408301	-0.2232221	2.9661072
C	4.2552425	0.9145859	2.3372279
H	4.6180664	1.9046916	2.0765949
H	4.7695636	0.5791668	3.2435438
H	4.5212129	0.2237874	1.5271593
C	3.2672016	3.3346259	4.0141948
H	3.4618736	2.3406768	4.4041570
H	4.2111837	3.8808798	3.9498380
H	2.6175373	3.8605204	4.7233448
C	2.1010894	4.5113513	2.2212332
H	1.2647956	4.8696324	2.8315437
H	2.8937933	5.2616972	2.2508324
H	1.7503156	4.3907493	1.2004525
H	2.1555010	1.3635557	-0.5451760
H	-1.9465595	-1.2003191	0.3322453
H	1.6246248	0.4168992	-3.1358936

(GFA3a+2H)$^{2+}$

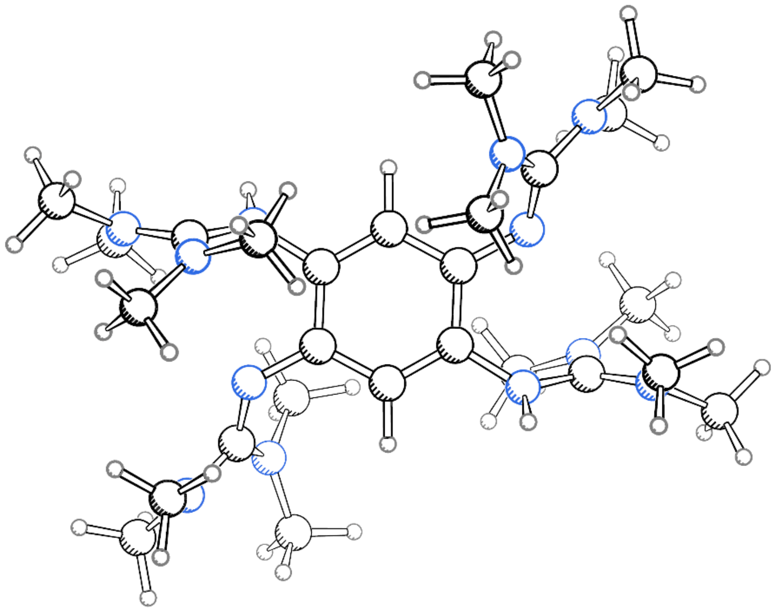

Energy = -1678.361142912

N	1.5955521	-0.4118544	-2.2714863
N	2.1717551	-2.6687089	-2.1387590
N	1.9297515	-1.6334776	-4.2089636
N	-0.8007786	-1.8582333	-1.9704520
N	-2.9103678	-0.8907858	-2.4875372
N	-2.5075157	-3.1495491	-2.8336861
C	0.7868366	-0.2429356	-1.1060351
C	-0.4387411	-0.9322909	-1.0082424
C	-1.2017399	-0.6580650	0.1366031
C	1.8789855	-1.5899924	-2.8697181
C	2.6402020	-2.5676197	-0.7627511
H	3.1281541	-1.6111490	-0.6037094
H	3.3621484	-3.3651100	-0.5882036
H	1.8139515	-2.6748435	-0.0566214
C	1.8037795	-4.0133982	-2.5746110
H	1.2393173	-4.4896244	-1.7735236
H	2.6860814	-4.6169354	-2.7918376
H	1.1678810	-3.9575294	-3.4507836
C	2.8670617	-2.4915566	-4.9337170
H	3.6039175	-2.9018903	-4.2502625
H	3.3846116	-1.8866259	-5.6791512
H	2.3519522	-3.3059486	-5.4454159
C	1.1328199	-0.7283396	-5.0328555
H	0.2585198	-0.4000859	-4.4779525
H	0.7974211	-1.2701150	-5.9170113
H	1.7129494	0.1368089	-5.3652800
C	-2.0374195	-1.9503799	-2.3981747
C	-2.4375598	0.4625096	-2.6958779
H	-2.4227396	1.0458144	-1.7705357

H	-3.0937471	0.9646208	-3.4100074
H	-1.4304890	0.4468285	-3.1029277
C	-4.3248884	-1.0119906	-2.1670111
H	-4.5529302	-2.0248293	-1.8489167
H	-4.9595982	-0.7575945	-3.0194535
H	-4.5721379	-0.3301008	-1.3466136
C	-3.4347854	-3.2773788	-3.9519443
H	-3.6430640	-2.3035234	-4.3837491
H	-4.3750198	-3.7399126	-3.6440952
H	-2.9842365	-3.9079055	-4.7225668
C	-1.8671744	-4.3859371	-2.4227703
H	-1.1668230	-4.7475967	-3.1820254
H	-2.6303291	-5.1519428	-2.2749853
H	-1.3279039	-4.2270447	-1.4943589
N	-1.5955521	0.4118544	2.2714863
N	-2.1717551	2.6687089	2.1387590
N	-1.9297515	1.6334776	4.2089636
N	0.8007786	1.8582333	1.9704520
N	2.9103678	0.8907858	2.4875372
N	2.5075157	3.1495491	2.8336861
C	-0.7868366	0.2429356	1.1060351
C	0.4387411	0.9322909	1.0082424
C	1.2017399	0.6580650	-0.1366031
C	-1.8789855	1.5899924	2.8697181
C	-2.6402020	2.5676197	0.7627511
H	-3.1281541	1.6111490	0.6037094
H	-3.3621484	3.3651100	0.5882036
H	-1.8139515	2.6748435	0.0566214
C	-1.8037795	4.0133982	2.5746110
H	-1.2393173	4.4896244	1.7735236
H	-2.6860814	4.6169354	2.7918376
H	-1.1678810	3.9575294	3.4507836
C	-2.8670617	2.4915566	4.9337170
H	-3.6039175	2.9018903	4.2502625
H	-3.3846116	1.8866259	5.6791512
H	-2.3519522	3.3059486	5.4454159
C	-1.1328199	0.7283396	5.0328555
H	-0.2585198	0.4000859	4.4779525
H	-0.7974211	1.2701150	5.9170113
H	-1.7129494	-0.1368089	5.3652800
C	2.0374195	1.9503799	2.3981747
C	2.4375598	-0.4625096	2.6958779
H	2.4227396	-1.0458144	1.7705357
H	3.0937471	-0.9646208	3.4100074
H	1.4304890	-0.4468285	3.1029277
C	4.3248884	1.0119906	2.1670111
H	4.5529302	2.0248293	1.8489167
H	4.9595982	0.7575945	3.0194535
H	4.5721379	0.3301008	1.3466136
C	3.4347854	3.2773788	3.9519443
H	3.6430640	2.3035234	4.3837491

H	4.3750198	3.7399126	3.6440952
H	2.9842365	3.9079055	4.7225668
C	1.8671744	4.3859371	2.4227703
H	1.1668230	4.7475967	3.1820254
H	2.6303291	5.1519428	2.2749853
H	1.3279039	4.2270447	1.4943589
H	2.1512599	1.1628782	-0.2629987
H	-2.1512599	-1.1628782	0.2629987
H	1.7935963	0.4171831	-2.8100912
H	-1.7935963	-0.4171831	2.8100912

GFA1b

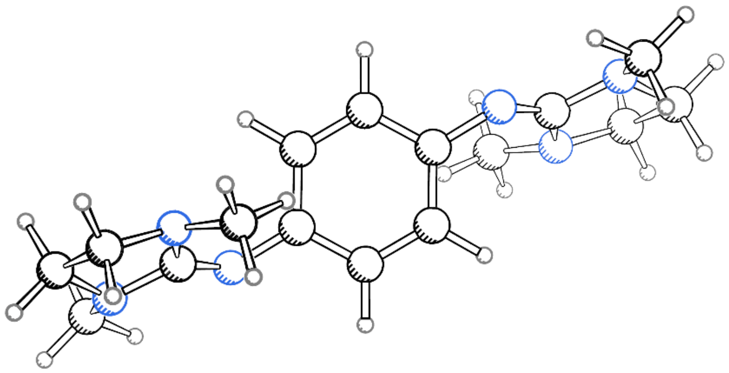

Energy = -952.4974909922

N	2.1379342	-0.6541891	-1.7092692
C	1.0658914	-0.3657226	-0.8622732
C	2.5494942	-1.8276818	-1.9938418
C	0.0019334	0.4188194	-1.3192417
C	1.0478093	-0.7650225	0.4806723
N	3.7272209	-2.0290484	-2.7048138
H	0.0057561	0.7440662	-2.3521314
C	-1.0468388	0.7657651	-0.4805220
C	-0.0008514	-0.4178882	1.3194538
C	-1.0649774	0.3663720	0.8623938
H	-1.8655687	1.3653352	-0.8591614
H	-0.0043247	-0.7425407	2.3525312
N	-2.1372924	0.6543158	1.7092131
C	-2.5494097	1.8276124	1.9937742
N	-2.0070988	3.0837124	1.7101060
N	-3.7273064	2.0284351	2.7046188
C	-4.1283251	3.4181548	2.6030607

C	-4.7413266	1.0009075	2.7305467
C	-2.7847963	4.1055759	2.4036293
C	-0.5723456	3.3058774	1.6735912
C	4.1277217	-3.4189065	-2.6031084
C	4.7416287	-1.0019096	-2.7310157
C	2.7839479	-4.1057856	-2.4034627
N	2.0066976	-3.0835357	-1.7100076
H	1.8664822	-1.3646893	0.8592821
H	-4.6489577	3.7527768	3.5014190
H	-4.7902662	3.5804741	1.7388617
H	-5.3572367	1.0088749	1.8202691
H	-5.3913382	1.1535199	3.5935779
H	-4.2541957	0.0325988	2.8083339
H	-2.8522104	5.0192735	1.8115076
H	-2.3256484	4.3515865	3.3716370
H	-0.0818547	2.5760706	1.0393320
H	-0.1308538	3.2563898	2.6780221
H	-0.3860717	4.2993439	1.2632016
H	4.6481431	-3.7538451	-3.5014706
H	4.7896802	-3.5813631	-1.7389490
H	5.3576528	-1.0099845	-1.8208163
H	5.3914705	-1.1548906	-3.5941088
H	4.2548560	-0.0334257	-2.8088764
H	2.8510662	-5.0194287	-1.8112244
H	2.3246193	-4.3517516	-3.3713955
C	0.5718572	-3.3051298	-1.6734079
H	0.0816805	-2.5750668	-1.0392022
H	0.1303422	-3.2555706	-2.6778252
H	0.3852097	-4.2984815	-1.2629100

(GFA1b)$^{2+}$

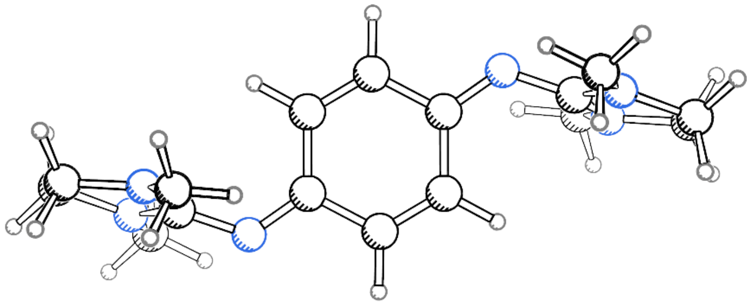

Energy = -951.9765495841

N	1.3047803	-1.0863484	-2.1099647
C	0.7054276	-0.6041415	-1.0779591
C	2.1650790	-2.1427782	-2.1132423
C	-0.2247506	0.5041012	-1.3065346
C	0.8909256	-1.0744763	0.2937667
N	3.4624370	-2.0041465	-2.3415092
H	-0.3462090	0.8320970	-2.3305249
C	-0.8935231	1.0723533	-0.2942296
C	0.2222747	-0.5061108	1.3060931
C	-0.7076058	0.6023956	1.0775843
H	-1.5822386	1.8879589	-0.4725595
H	0.3436645	-0.8341730	2.3300698
N	-1.3062675	1.0851280	2.1097905
C	-2.1654740	2.1423739	2.1134828
N	-1.7916564	3.4201708	2.0220027
N	-3.4616124	2.0052714	2.3496716
C	-4.1200732	3.3229043	2.3582811
C	-4.2054718	0.7578465	2.4125891
C	-2.9255801	4.2899046	2.3957793
C	-0.4290721	3.9293371	2.0525045
C	4.1213491	-3.3215556	-2.3532435
C	4.2063004	-0.7563490	-2.3954262
C	2.9274055	-4.2888434	-2.4007718
N	1.7911952	-3.4210193	-2.0295085
H	1.5794328	-1.8902876	0.4720363
H	-4.7689940	3.4148164	3.2272990
H	-4.7241844	3.4364280	1.4558179
H	-4.7801996	0.6030303	1.4971522
H	-4.8907949	0.7954689	3.2583819

H	-3.5140143	-0.0685104	2.5563051
H	-3.0198188	5.1130686	1.6905044
H	-2.7519256	4.6988457	3.3936992
H	0.2719067	3.1418926	1.7921348
H	-0.1870274	4.3049480	3.0492605
H	-0.3332740	4.7422375	1.3339302
H	4.7742802	-3.4092120	-3.2196909
H	4.7213745	-3.4390584	-1.4485782
H	4.7785100	-0.6067747	-1.4775591
H	4.8939574	-0.7889560	-3.2395414
H	3.5152420	0.0708124	-2.5362761
H	3.0182661	-5.1147398	-1.6982402
H	2.7591432	-4.6939289	-3.4011829
C	0.4288341	-3.9302469	-2.0691985
H	-0.2734337	-3.1441475	-1.8081742
H	0.1916689	-4.3008040	-3.0690197
H	0.3297464	-4.7467829	-1.3552021

GFA2b

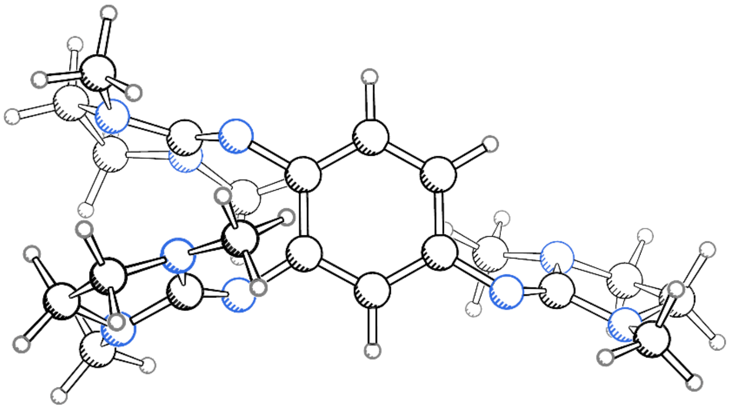

Energy = -1312.650191316

N	3.5005462	0.5156383	1.1895992
C	2.2076604	1.0158693	1.3700640
C	4.0458180	-0.3704288	1.9277768
C	1.0792552	0.1809585	1.1689679
C	1.9912583	2.3719402	1.6109519
N	3.7846035	-0.7863464	3.2223924
N	5.1521607	-1.0949870	1.4872101
N	1.2183116	-1.1962299	0.9833863
C	-0.1995466	0.7269541	1.2816991
C	0.7122547	2.9047988	1.7079238

C	4.6312354	-1.9114442	3.5763345
C	2.5542394	-0.5638373	3.9502165
C	5.8018042	-1.7392128	2.6112606
C	5.9313725	-0.6221481	0.3710933
C	1.8595910	-1.7640184	0.0348369
C	-0.4074149	2.0786741	1.5616414
N	2.4457902	-1.2585504	-1.1120508
N	2.0679216	-3.1434665	0.0334096
N	-1.7098549	2.5897315	1.5991770
C	2.9270864	-2.3464835	-1.9484440
C	2.0020885	-0.0539154	-1.7789682
C	3.0897966	-3.4766813	-0.9405214
C	2.0095806	-3.8879066	1.2656314
C	-2.2425905	3.1464518	2.6134278
N	-1.7900618	3.2853102	3.9297665
N	-3.4884079	3.7629879	2.5325912
C	-2.8561681	3.8612353	4.7416873
C	-0.9915540	2.2592509	4.5744345
C	-3.7008454	4.5885933	3.7049483
C	-3.9886236	4.2107632	1.2545176
H	2.8578721	3.0121321	1.7229384
H	-1.0544738	0.0773424	1.1471531
H	0.5721546	3.9600903	1.9039870
H	4.9327802	-1.8581211	4.6238009
H	4.1196942	-2.8698019	3.4139823
H	1.7553687	-1.2397618	3.6302076
H	2.7536916	-0.7179162	5.0123397
H	2.2160988	0.4578726	3.8084395
H	6.2565838	-2.6868558	2.3190759
H	6.5831968	-1.0990253	3.0485498
H	6.6522545	0.1535369	0.6654960
H	6.4786227	-1.4567965	-0.0714906
H	5.2502947	-0.1985132	-0.3628927
H	2.1883609	-2.6069870	-2.7201788
H	3.8615523	-2.0768958	-2.4435697
H	2.7983792	0.2944048	-2.4385327
H	1.1029609	-0.2347381	-2.3831154
H	1.7901446	0.7286962	-1.0601041
H	4.0907593	-3.4560027	-0.4837820
H	2.9246659	-4.4639391	-1.3743625
H	1.7764212	-4.9330091	1.0536905
H	2.9618171	-3.8480057	1.8128527
H	1.2294923	-3.4626103	1.8917686
H	-3.4405651	3.0710951	5.2348298
H	-2.4533587	4.5230795	5.5098814
H	-0.1458632	1.9847921	3.9538963
H	-0.6150816	2.6557017	5.5188267
H	-1.5828574	1.3580372	4.7862049
H	-3.3352555	5.6144481	3.5441604
H	-4.7567235	4.6391622	3.9749450
H	-3.7336985	3.4692193	0.5020496

H	-5.0730361	4.3212436	1.3067594
H	-3.5505675	5.1738143	0.9561628

(GFA2b)$^{2+}$

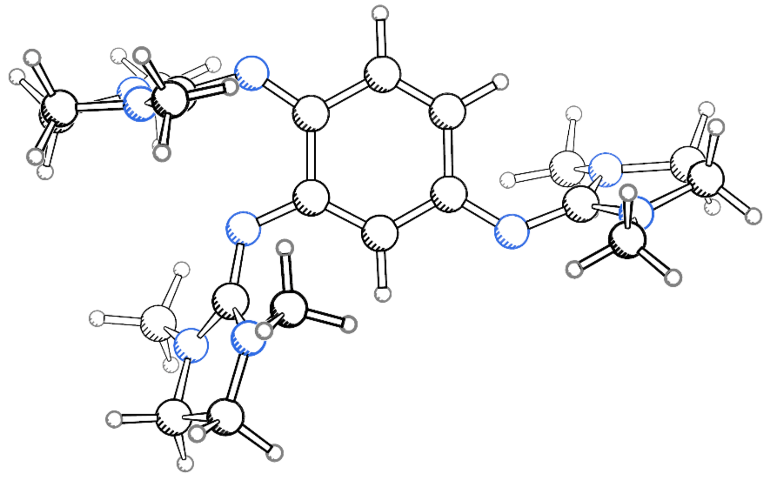

Energy = -1312.173763952

N	3.7672891	0.7857630	2.9252700
C	2.5405167	1.0092842	2.6362963
C	4.5710903	-0.2318257	2.5298869
C	1.7011322	0.1331934	1.7536395
C	1.9402299	2.2141785	3.2020428
N	4.8585277	-1.2676094	3.3141672
N	5.3493828	-0.1879414	1.4526669
N	2.3054121	-0.9789235	1.3396079
C	0.3998798	0.5284020	1.5081580
C	0.6733498	2.5439788	2.9373329
C	5.8247663	-2.1506924	2.6514984
C	4.1422024	-1.6544719	4.5114152
C	6.3285008	-1.2830420	1.4846333
C	5.4017471	0.8663606	0.4627941
C	1.8400603	-1.7534242	0.3574215
C	-0.1656125	1.6992633	2.0699378
N	1.5697072	-1.3725995	-0.9045001
N	1.6850892	-3.0789104	0.4861943
N	-1.4021762	2.0166197	1.7848426
C	1.0770921	-2.5030901	-1.7002824
C	1.6620733	-0.0351480	-1.4442151
C	1.4050762	-3.7102607	-0.8058395
C	1.9698066	-3.8506071	1.6749040
C	-2.1345103	2.9870781	2.3489131
N	-2.4795774	3.0620354	3.6487620

N	-2.7212143	3.9491960	1.6359656
C	-3.5423661	4.0671137	3.8208809
C	-2.3367246	1.9949395	4.6240762
C	-3.4719176	4.8587456	2.5080363
C	-2.4341259	4.2646665	0.2499046
H	2.5767375	2.8217230	3.8309801
H	-0.2485496	-0.0847052	0.8978617
H	0.2392217	3.4437695	3.3523197
H	6.6178235	-2.4346032	3.3412236
H	5.3207846	-3.0570379	2.3064080
H	3.4168159	-2.4436501	4.3000876
H	4.8528824	-2.0181901	5.2525178
H	3.6250867	-0.7901428	4.9204657
H	6.3365215	-1.8165490	0.5351265
H	7.3269670	-0.8787510	1.6653787
H	6.4064462	1.2908740	0.4209183
H	5.1451166	0.4738092	-0.5228971
H	4.7033828	1.6551677	0.7288352
H	0.0038952	-2.3990891	-1.8784851
H	1.5843711	-2.5419386	-2.6631489
H	2.0670073	-0.0861919	-2.4549506
H	0.6859542	0.4554574	-1.4812567
H	2.3303357	0.5619038	-0.8292279
H	2.2838032	-4.2602783	-1.1541140
H	0.5739952	-4.4083240	-0.7202496
H	1.1440667	-4.5313546	1.8840503
H	2.8819515	-4.4407822	1.5472054
H	2.0919164	-3.1699987	2.5122833
H	-4.5034583	3.5608182	3.9456273
H	-3.3541701	4.6799137	4.7002630
H	-1.5925706	1.2783122	4.2905119
H	-2.0216932	2.4139365	5.5792990
H	-3.2891339	1.4764531	4.7627780
H	-2.9267918	5.7997422	2.6184389
H	-4.4530279	5.0732540	2.0876875
H	-1.9778917	3.3985325	-0.2224414
H	-3.3646836	4.5040855	-0.2634846
H	-1.7588263	5.1207517	0.1745091

GFA3b

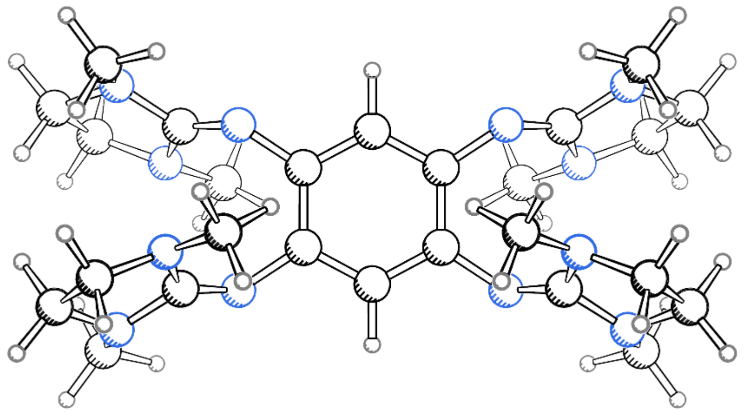

Energy = -1672.802689185

N	1.4248036	-0.4032104	-2.3896827
N	1.9496161	-1.0256640	-4.5642316
N	0.1204111	-1.9894365	-3.6952265
N	1.4248036	-0.4032104	2.3896827
N	1.9496161	-1.0256640	4.5642316
N	0.1204111	-1.9894365	3.6952265
C	0.6646739	-0.2458245	-1.2298305
C	1.2917652	-0.4473449	0.0000000
H	2.3211890	-0.7810186	0.0000000
C	0.6646739	-0.2458245	1.2298305
C	1.1461831	-1.0909882	-3.4233613
C	0.3805570	-2.6642534	-4.9574779
H	-0.5482884	-2.8678024	-5.4936261
H	0.9012028	-3.6191477	-4.7946685
C	1.2810014	-1.6679034	-5.6763583
H	1.9916283	-2.1464020	-6.3524332
H	0.6866205	-0.9461751	-6.2580139
C	-0.5363500	-2.7612013	-2.6619674
H	0.0957675	-3.5886713	-2.3117389
H	-1.4585544	-3.1773921	-3.0701175
H	-0.7920490	-2.1303809	-1.8186904
C	2.7685985	0.1356429	-4.8079118
H	2.1990077	0.9467426	-5.2826697
H	3.6042134	-0.1327074	-5.4571504
H	3.1482217	0.4944187	-3.8548625
C	1.1461831	-1.0909882	3.4233613
C	1.2810014	-1.6679034	5.6763583
H	0.6866205	-0.9461751	6.2580139
H	1.9916283	-2.1464020	6.3524332

C	0.3805570	-2.6642534	4.9574779
H	0.9012028	-3.6191477	4.7946685
H	-0.5482884	-2.8678024	5.4936261
C	2.7685985	0.1356429	4.8079118
H	3.1482217	0.4944187	3.8548625
H	3.6042134	-0.1327074	5.4571504
H	2.1990077	0.9467426	5.2826697
C	-0.5363500	-2.7612013	2.6619674
H	-0.7920490	-2.1303809	1.8186904
H	-1.4585544	-3.1773921	3.0701175
H	0.0957675	-3.5886713	2.3117389
C	-0.6646739	0.2458245	-1.2298305
C	-0.6646739	0.2458245	1.2298305
N	-1.4248036	0.4032104	-2.3896827
C	-1.2917652	0.4473449	0.0000000
N	-1.4248036	0.4032104	2.3896827
C	-1.1461831	1.0909882	-3.4233613
H	-2.3211890	0.7810186	0.0000000
C	-1.1461831	1.0909882	3.4233613
N	-1.9496161	1.0256640	-4.5642316
N	-0.1204111	1.9894365	-3.6952265
N	-1.9496161	1.0256640	4.5642316
N	-0.1204111	1.9894365	3.6952265
C	-1.2810014	1.6679034	-5.6763583
C	-2.7685985	-0.1356429	-4.8079118
C	-0.3805570	2.6642534	-4.9574779
C	0.5363500	2.7612013	-2.6619674
C	-1.2810014	1.6679034	5.6763583
C	-2.7685985	-0.1356429	4.8079118
C	-0.3805570	2.6642534	4.9574779
C	0.5363500	2.7612013	2.6619674
H	-0.6866205	0.9461751	-6.2580139
H	-1.9916283	2.1464020	-6.3524332
H	-3.1482217	-0.4944187	-3.8548625
H	-3.6042134	0.1327074	-5.4571504
H	-2.1990077	-0.9467426	-5.2826697
H	-0.9012028	3.6191477	-4.7946685
H	0.5482884	2.8678024	-5.4936261
H	0.7920490	2.1303809	-1.8186904
H	1.4585544	3.1773921	-3.0701175
H	-0.0957675	3.5886713	-2.3117389
H	-1.9916283	2.1464020	6.3524332
H	-0.6866205	0.9461751	6.2580139
H	-2.1990077	-0.9467426	5.2826697
H	-3.6042134	0.1327074	5.4571504
H	-3.1482217	-0.4944187	3.8548625
H	0.5482884	2.8678024	5.4936261
H	-0.9012028	3.6191477	4.7946685
H	-0.0957675	3.5886713	2.3117389
H	1.4585544	3.1773921	3.0701175
H	0.7920490	2.1303809	1.8186904

(GFA3b)$^{2+}$

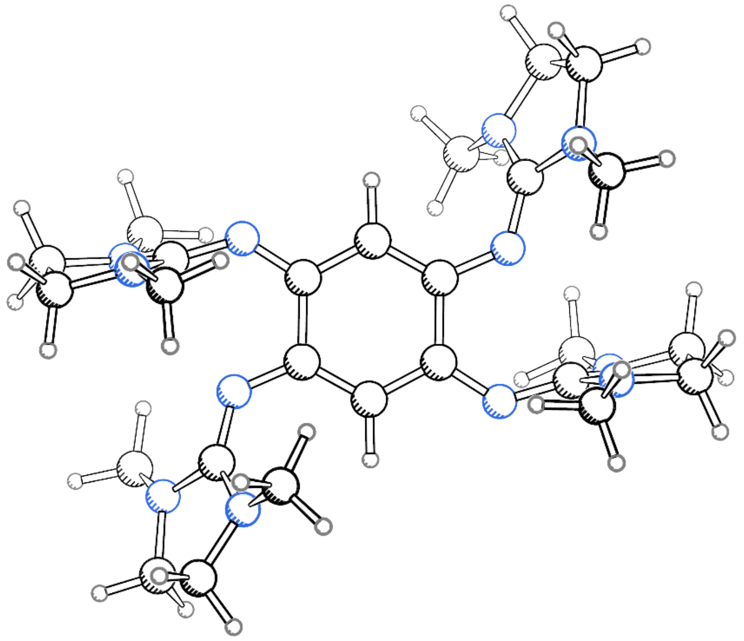

Energy = -1672.367838128

N	1.1385120	1.7524129	1.8190058
N	1.1172387	2.7006959	3.9466449
N	-0.4669425	3.3897008	2.5597029
N	2.6560719	0.6148134	-0.1710946
N	3.6324315	2.7270838	0.2703020
N	4.2608120	1.0106057	1.5121290
C	0.5085670	0.9367559	0.9790278
C	1.3877621	0.3410409	-0.0923802
C	0.8166418	-0.5549167	-1.0230309
H	1.4720559	-0.9499982	-1.7860525
C	0.5875073	2.5539301	2.7167855
C	2.0577527	1.7730699	4.5411864
H	2.5858534	1.2563495	3.7443035
H	1.5460769	1.0398125	5.1713848
H	2.7694498	2.3239514	5.1560610
C	-0.9799852	3.8434824	1.2809462
H	-0.5491500	4.8147513	1.0183154
H	-2.0631277	3.9502460	1.3406371
H	-0.7452773	3.1243733	0.5041709
C	0.2815522	3.5729834	4.7719629
H	0.8895969	4.2588422	5.3598634
H	-0.3224197	2.9702448	5.4569240
C	-0.5769104	4.2829559	3.7211456
H	-1.6152842	4.4003513	4.0257393
H	-0.1759233	5.2685513	3.4642945
C	3.4186764	1.4324166	0.5550072
C	4.5545061	3.3084850	1.2471204
H	3.9900183	3.8623961	2.0047592
H	5.2562869	3.9875463	0.7665670

C	5.2285419	2.0632566	1.8345529
H	6.1872330	1.8492191	1.3524951
H	5.3875910	2.1321775	2.9094222
C	2.7432187	3.5514327	-0.5147200
H	2.2312035	2.9328840	-1.2479642
H	3.3230371	4.3089726	-1.0403197
H	2.0015504	4.0467194	0.1193626
C	4.5199561	-0.3780364	1.8306543
H	3.6850921	-0.9859450	1.4940712
H	4.6326512	-0.4930870	2.9088854
H	5.4348035	-0.7268401	1.3431613
C	-0.8166418	0.5549167	1.0230309
C	-0.5085670	-0.9367559	-0.9790278
C	-1.3877621	-0.3410409	0.0923802
H	-1.4720559	0.9499982	1.7860525
N	-1.1385120	-1.7524129	-1.8190058
N	-2.6560719	-0.6148134	0.1710946
C	-0.5875073	-2.5539301	-2.7167855
C	-3.4186764	-1.4324166	-0.5550072
N	-1.1172387	-2.7006959	-3.9466449
N	0.4669425	-3.3897008	-2.5597029
N	-3.6324315	-2.7270838	-0.2703020
N	-4.2608120	-1.0106057	-1.5121290
C	-2.0577527	-1.7730699	-4.5411864
C	-0.2815522	-3.5729834	-4.7719629
C	0.9799852	-3.8434824	-1.2809462
C	0.5769104	-4.2829559	-3.7211456
C	-4.5545061	-3.3084850	-1.2471204
C	-2.7432187	-3.5514327	0.5147200
C	-5.2285419	-2.0632566	-1.8345529
C	-4.5199561	0.3780364	-1.8306543
H	-2.5858534	-1.2563495	-3.7443035
H	-1.5460769	-1.0398125	-5.1713848
H	-2.7694498	-2.3239514	-5.1560610
H	-0.8895969	-4.2588422	-5.3598634
H	0.3224197	-2.9702448	-5.4569240
H	0.5491500	-4.8147513	-1.0183154
H	2.0631277	-3.9502460	-1.3406371
H	0.7452773	-3.1243733	-0.5041709
H	1.6152842	-4.4003513	-4.0257393
H	0.1759233	-5.2685513	-3.4642945
H	-3.9900183	-3.8623961	-2.0047592
H	-5.2562869	-3.9875463	-0.7665670
H	-2.2312035	-2.9328840	1.2479642
H	-3.3230371	-4.3089726	1.0403197
H	-2.0015504	-4.0467194	-0.1193626
H	-6.1872330	-1.8492191	-1.3524951
H	-5.3875910	-2.1321775	-2.9094222
H	-3.6850921	0.9859450	-1.4940712
H	-4.6326512	0.4930870	-2.9088854
H	-5.4348035	0.7268401	-1.3431613

(GFA3b+H)$^+$

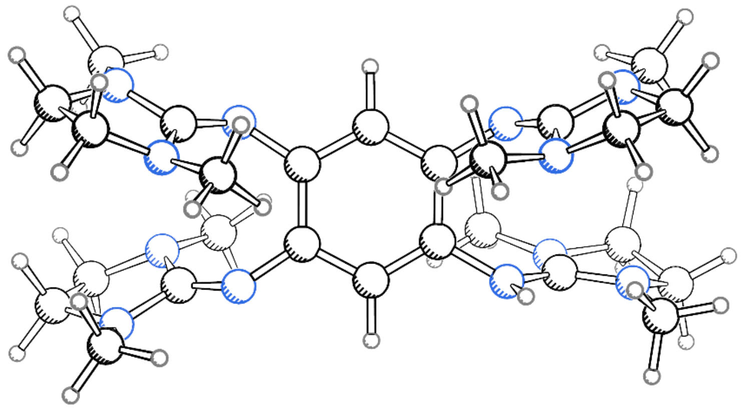

Energy = -1673.241836122

N	6.7822347	5.3347148	-2.2430084
N	6.5217987	6.3750784	-4.3339881
N	5.6021366	4.3752252	-4.0828926
N	7.0653346	2.1954998	1.4081324
N	7.6889147	0.3726934	2.6939070
N	5.8864575	0.0699402	1.4076364
C	6.1396345	4.6606751	-1.1457671
C	6.8431632	3.7166916	-0.4124770
H	7.8691374	3.4895971	-0.6717016
C	6.2634845	3.0311430	0.6564501
C	6.3015192	5.3465969	-3.4844947
C	5.1571021	4.8342440	-5.4022629
H	4.1173746	5.1691106	-5.3510900
H	5.2338939	4.0278441	-6.1288135
C	6.1184311	5.9883554	-5.6874402
H	6.9865424	5.6634710	-6.2704408
H	5.6426118	6.8230756	-6.1990830
C	4.9839756	3.2033428	-3.4784361
H	5.6420278	2.7665447	-2.7351874
H	4.8166341	2.4771217	-4.2724118
H	4.0334736	3.4622988	-3.0093572
C	7.4699400	7.4451284	-4.1008837
H	7.3282719	7.8601060	-3.1028601
H	7.2727573	8.2401879	-4.8174413
H	8.5071958	7.1161502	-4.2215574
C	6.8543141	0.9882632	1.7811077
C	7.1343340	-0.9008092	3.1106774
H	6.5477150	-0.7936558	4.0342351
H	7.9156060	-1.6404429	3.2872204

C	6.2427850	-1.2485221	1.9251547
H	6.7953510	-1.8253166	1.1718487
H	5.3532645	-1.8125924	2.2066493
C	8.5078484	1.1371460	3.6028131
H	8.8256081	2.0473563	3.1017613
H	9.3846085	0.5532779	3.8856242
H	7.9580693	1.4105271	4.5125091
C	5.2266603	0.1129608	0.1194477
H	4.8180336	1.0990055	-0.0695726
H	4.3994901	-0.5967367	0.1315153
H	5.9084689	-0.1620132	-0.6960892
C	4.8152847	4.9912835	-0.8303091
C	4.9078881	3.3478611	0.9929024
N	4.0749933	5.8910156	-1.6041818
C	4.2244520	4.2962080	0.2208812
N	4.1923580	2.6664708	1.9477659
C	4.3292984	7.1428812	-1.6962533
H	3.1922941	4.5038194	0.4681122
C	4.4720817	2.4288727	3.1755153
N	3.6249219	7.9641710	-2.5500010
N	5.3015062	7.9233138	-1.0538590
N	3.7171348	1.5445183	3.9174433
N	5.4476237	2.9531286	4.0049416
C	4.2763351	9.2519581	-2.6806624
C	2.8206641	7.4401636	-3.6240958
C	5.0429783	9.3340013	-1.3680616
C	5.6657799	7.6736727	0.3380872
C	4.3328135	1.3068407	5.2086363
C	2.9102190	0.5247002	3.2941247
C	5.1677836	2.5696226	5.3862848
C	6.0372389	4.2575193	3.7860220
H	4.9525323	9.2691598	-3.5482333
H	3.5521786	10.0577550	-2.7999659
H	2.4201189	6.4761401	-3.3221807
H	1.9976101	8.1230637	-3.8363335
H	3.4073176	7.3125339	-4.5448503
H	4.4245703	9.7909673	-0.5861320
H	5.9736843	9.8958902	-1.4436595
H	5.9770435	6.6450916	0.4838946
H	6.4962740	8.3306856	0.5962346
H	4.8310430	7.8858787	1.0158817
H	3.5853703	1.1772757	5.9916762
H	4.9656840	0.4083907	5.1856297
H	3.4866963	-0.3893577	3.1041077
H	2.0663374	0.2785888	3.9398459
H	2.5402626	0.9070765	2.3465952
H	6.0912271	2.3999181	5.9403439
H	4.5943542	3.3532269	5.8984736
H	5.3214832	5.0660213	3.9822311
H	6.8831062	4.3714022	4.4637924
H	6.4084523	4.3435746	2.7710922

H	7.3241119	6.1511432	-1.9984831

(GFA3b+2H)$^{2+}$

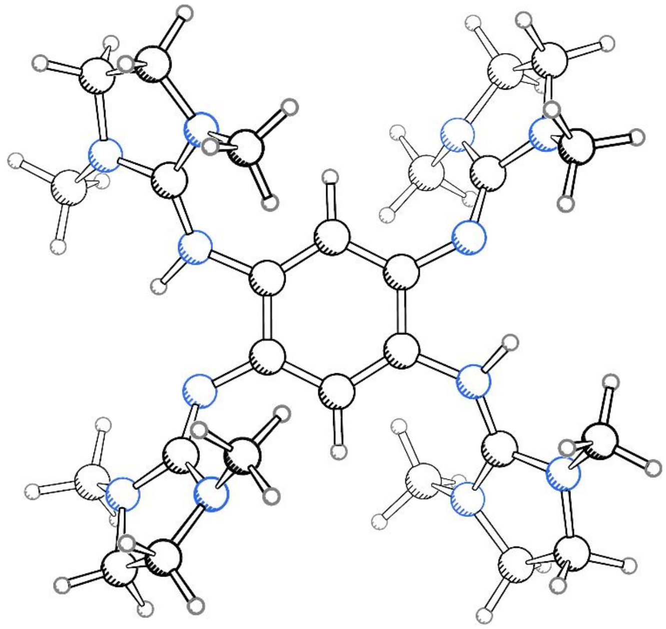

Energy = -1673.586190474

N	0.4495803	1.3237956	2.4104291
N	0.0360622	2.2126220	4.5271466
N	-1.4287706	2.7156467	2.9229245
N	2.5301847	0.6522713	1.0030356
N	3.8056862	1.4853046	-0.8803000
N	4.8474320	0.8279621	0.9853570
C	0.1597847	0.6967902	1.1763340
C	1.3112496	0.3782737	0.4212655
C	1.1128864	-0.3176307	-0.7777582
H	1.9718384	-0.5557148	-1.3883655
C	-0.2934259	2.0608046	3.2330441
C	0.9842592	1.3649743	5.2339838
H	1.9484198	1.3548066	4.7279079
H	0.6143809	0.3408012	5.3342973
H	1.1406310	1.7846461	6.2248093
C	-1.7078909	3.3159255	1.6214994
H	-1.4326397	4.3736994	1.6398030
H	-2.7720453	3.2331432	1.4034126
H	-1.1503475	2.8172330	0.8380352
C	-1.0663533	2.8607955	5.2492594
H	-0.6912094	3.6155629	5.9376350
H	-1.6213559	2.1105012	5.8191944
C	-1.9019832	3.4512951	4.1112987
H	-2.9724660	3.3145750	4.2512782
H	-1.7015006	4.5168031	3.9727002
C	3.6315835	0.9646013	0.3877331

C	5.2122802	1.8832925	-1.0427696
H	5.3161104	2.9584879	-0.8593771
H	5.5672936	1.6680994	-2.0492733
C	5.9236655	1.0712484	0.0332855
H	6.3067334	0.1208926	-0.3573282
H	6.7504602	1.6095217	0.4950895
C	2.8336826	2.3958050	-1.4677185
H	1.8295596	1.9899652	-1.4067407
H	3.0837482	2.5390894	-2.5183578
H	2.8545329	3.3731793	-0.9716754
C	5.0786308	-0.0247390	2.1312157
H	4.1501474	-0.1357582	2.6832675
H	5.8339751	0.4235547	2.7768169
H	5.4283176	-1.0165490	1.8233616
C	-1.1128864	0.3176307	0.7777582
C	-0.1597847	-0.6967902	-1.1763340
C	-1.3112496	-0.3782737	-0.4212655
H	-1.9718384	0.5557148	1.3883655
N	-0.4495803	-1.3237956	-2.4104291
N	-2.5301847	-0.6522713	-1.0030356
C	0.2934259	-2.0608046	-3.2330441
C	-3.6315835	-0.9646013	-0.3877331
N	-0.0360622	-2.2126220	-4.5271466
N	1.4287706	-2.7156467	-2.9229245
N	-3.8056862	-1.4853046	0.8803000
N	-4.8474320	-0.8279621	-0.9853570
C	-0.9842592	-1.3649743	-5.2339838
C	1.0663533	-2.8607955	-5.2492594
C	1.7078909	-3.3159255	-1.6214994
C	1.9019832	-3.4512951	-4.1112987
C	-5.2122802	-1.8832925	1.0427696
C	-2.8336826	-2.3958050	1.4677185
C	-5.9236655	-1.0712484	-0.0332855
C	-5.0786308	0.0247390	-2.1312157
H	-1.9484198	-1.3548066	-4.7279079
H	-0.6143809	-0.3408012	-5.3342973
H	-1.1406310	-1.7846461	-6.2248093
H	0.6912094	-3.6155629	-5.9376350
H	1.6213559	-2.1105012	-5.8191944
H	1.4326397	-4.3736994	-1.6398030
H	2.7720453	-3.2331432	-1.4034126
H	1.1503475	-2.8172330	-0.8380352
H	2.9724660	-3.3145750	-4.2512782
H	1.7015006	-4.5168031	-3.9727002
H	-5.3161104	-2.9584879	0.8593771
H	-5.5672936	-1.6680994	2.0492733
H	-1.8295596	-1.9899652	1.4067407
H	-3.0837482	-2.5390894	2.5183578
H	-2.8545329	-3.3731793	0.9716754
H	-6.3067334	-0.1208926	0.3573282
H	-6.7504602	-1.6095217	-0.4950895

H	-4.1501474	0.1357582	-2.6832675
H	-5.8339751	-0.4235547	-2.7768169
H	-5.4283176	1.0165490	-1.8233616
H	1.4289394	1.1724843	2.6532401
H	-1.4289394	-1.1724843	-2.6532401

TGFA1

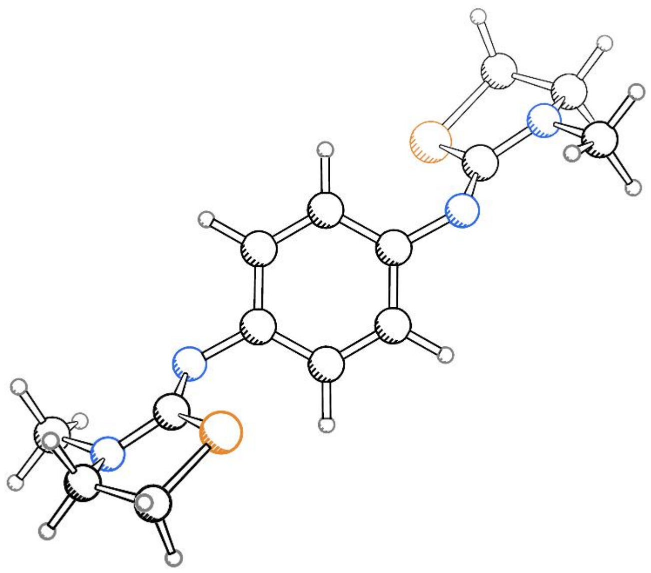

Energy = -1559.537273880

N	0.3264652	7.1554826	4.6414746
C	-0.5449928	6.6051637	5.5911257
C	0.6139948	8.3904424	4.6218092
C	-1.8570764	7.0619856	5.7611718
C	-0.1161119	5.5217798	6.3616983
S	0.1819304	9.6154676	5.8662062
N	1.3643899	8.9741158	3.6247462
C	-2.7005715	6.4678864	6.6865085
C	-0.9596183	4.9277171	7.2870536
C	1.0041616	10.9376077	4.9130552
C	2.0024883	10.2163691	4.0186220
C	2.0521039	8.1192187	2.6780372
C	-2.2716591	5.3845460	7.4570783
N	-3.1431018	4.8342661	8.4067045
C	-3.4310114	3.5994013	8.4260289
S	-2.9996842	2.3746154	7.1812085
N	-4.1813887	3.0156655	9.4231000
C	-3.8220703	1.0524484	8.1342001
C	-4.8200488	1.7737676	9.0289617
C	-4.8687261	3.8705652	10.3700826
H	-3.0769812	0.5185226	8.7232746
H	-4.3065788	0.3592599	7.4502402

H	-5.0579946	1.1847652	9.9168066
H	-5.7563722	1.9659945	8.4845375
H	-5.7828578	4.3010901	9.9415495
H	-5.1311288	3.2866993	11.2528627
H	-4.2093535	4.6868643	10.6519079
H	-2.2143728	7.8845003	5.1558892
H	0.8953053	5.1593020	6.2300754
H	-3.7119906	6.8303361	6.8181299
H	-0.6022995	4.1052758	7.8924181
H	0.2590299	11.4711990	4.3237337
H	1.4883533	11.6310768	5.5969586
H	2.2403176	10.8052015	3.1306371
H	2.9388272	10.0246866	4.5632112
H	2.9663622	7.6891334	3.1067393
H	2.3143651	8.7029447	1.7951260
H	1.3930566	7.3026059	2.3963692

$(TGFA1)^{2+}$

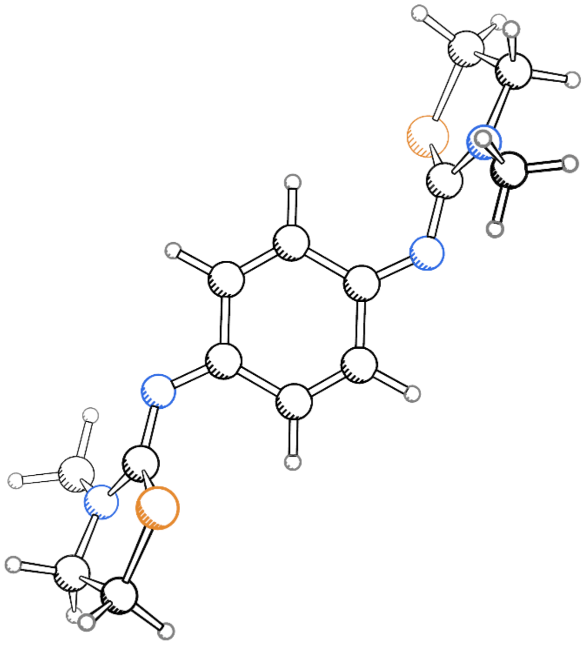

Energy = -1558.978074708

N	0.7897920	7.2940540	5.6204945
C	-0.2846791	6.7184689	6.0439885
C	0.9434140	8.5655698	5.1973233
C	-1.6280542	7.2831863	5.9502385
C	-0.1314761	5.3907317	6.6377626
S	0.6938591	9.9138600	6.2605009
N	1.4538780	8.8653214	4.0268221
C	-2.6850454	6.5996580	6.4112161
C	-1.1884674	4.7072025	7.0987456
C	1.0848504	11.0891051	4.9007423
C	1.8525520	10.2747942	3.8611890

C	1.8309432	7.8858201	3.0089782
C	-2.5318606	5.2718550	7.0048503
N	-3.6064237	4.6960675	7.4278850
C	-3.7600570	3.4244323	7.8507153
S	-3.5094912	2.0763813	6.7874754
N	-4.2713465	3.1243712	9.0207740
C	-3.9009852	0.9008505	8.1468388
C	-4.6698450	1.7147858	9.1858417
C	-4.6495809	4.1036318	10.0384161
H	-2.9598463	0.5141984	8.5320731
H	-4.4917253	0.0831156	7.7431216
H	-4.4381899	1.3887837	10.1986173
H	-5.7510726	1.6535715	9.0379313
H	-5.6913952	3.9391936	10.3140812
H	-4.0220191	3.9795097	10.9210949
H	-4.5324230	5.1065728	9.6387681
H	-1.7522493	8.2579654	5.4993426
H	0.8745228	4.9971426	6.6980631
H	-3.6910503	6.9932321	6.3508970
H	-1.0642715	3.7324172	7.5496350
H	0.1435928	11.4761537	4.5162095
H	1.6762584	11.9066042	5.3039571
H	1.6200587	10.6006897	2.8485729
H	2.9339296	10.3357737	4.0081037
H	2.8725145	8.0500271	2.7322733
H	1.2025486	8.0099003	2.1268879
H	1.7140007	6.8829713	3.4089121

TGFA2

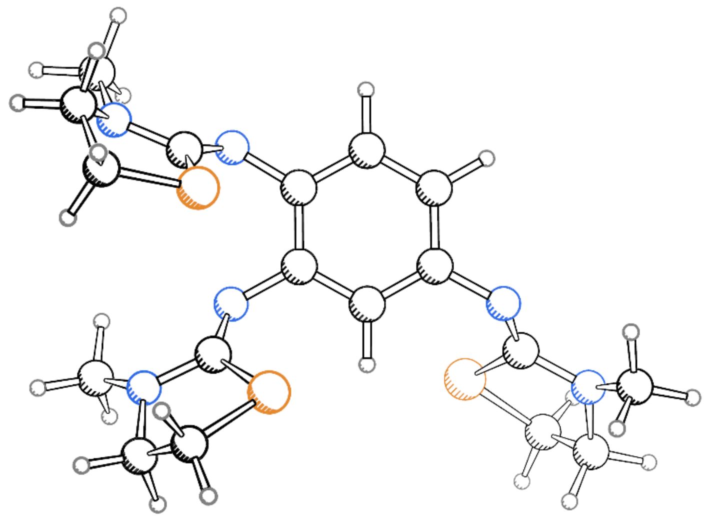

Energy = -2223.205806912

C	6.0669437	0.4687054	8.5572780
N	4.7093765	0.5687333	8.8910710
C	6.6583898	1.3084190	7.6118929
C	6.8675260	-0.4635601	9.2208078
C	3.7871159	0.4683344	8.0278638
C	8.0202141	1.2269661	7.3122923
C	8.2125359	-0.5734528	8.9081342
S	3.9834973	0.1794846	6.2613515
N	2.4480406	0.5470224	8.3458411
N	8.6084376	2.0209446	6.3256560
N	9.1980301	4.0600247	5.3872622
C	8.4320964	5.8289765	6.7620308
C	8.7599571	5.4386345	5.3283055
C	8.8154226	0.2607756	7.9660515
C	2.1817918	-0.0386471	6.0627633
C	1.5685134	0.7466808	7.2122217
C	2.0565967	1.0826517	9.6328948
C	8.5441605	3.2854325	6.3209475
C	9.7345730	3.4348492	4.1972543
S	7.6641548	4.3391871	7.4824922
N	10.1626922	0.0794108	7.6342019
C	11.0322079	0.9791443	7.8306389
S	10.8257893	2.4673470	8.8137117
N	12.3178265	0.9100622	7.3405699
C	12.5069866	3.0301617	8.3856597
C	13.2657251	1.7571612	8.0366388
C	12.7842290	-0.3356022	6.7686068
H	12.4526050	3.7026677	7.5294654
H	12.9450545	3.5540006	9.2323603
H	14.1186832	1.9659015	7.3873991
H	13.6409680	1.2705718	8.9492687
H	13.0803043	-1.0553842	7.5426907
H	13.6412857	-0.1365412	6.1239437
H	11.9788387	-0.7753723	6.1863400
H	7.7331198	6.6604996	6.8169500
H	9.3359825	6.0755318	7.3193414
H	7.8750742	5.5514805	4.6842178
H	9.5587337	6.0611142	4.9192337
H	10.1341084	2.4607596	4.4671750
H	10.5292591	4.0604813	3.7885042
H	8.9650833	3.2910767	3.4277773
H	1.8758883	0.3427000	5.0911100
H	1.9343426	-1.0977371	6.1317846
H	1.4890452	1.8131892	6.9530095
H	0.5681449	0.3812465	7.4539477
H	2.0545473	2.1805321	9.6337857
H	2.7641927	0.7418219	10.3837702
H	1.0557591	0.7276069	9.8817693
H	6.0507415	2.0362129	7.0918141
H	6.4172733	-1.1080722	9.9639312
H	8.8294645	-1.3148754	9.3995904

$(TGFA2)^{2+}$

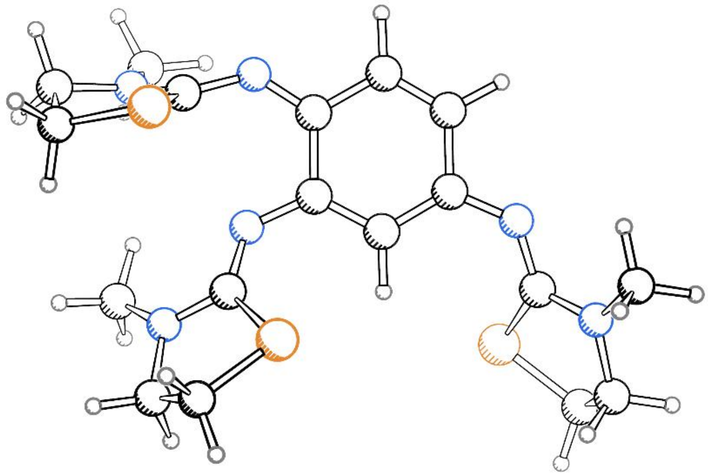

Energy = -2222.688544066

C	6.0959976	0.1504029	8.5141996
N	4.8010728	-0.0384048	8.5571628
C	6.7714549	1.1262918	7.7302534
C	6.8747450	-0.7324994	9.3859777
C	3.8591858	0.3719263	7.7182128
C	8.1426816	1.2487379	7.7502046
C	8.2097522	-0.6649217	9.4270151
S	4.0268531	0.4262715	5.9744993
N	2.6370972	0.6559025	8.1381880
N	8.8947997	2.0748700	7.0121565
N	9.2516566	3.9177007	5.6624211
C	7.9232143	5.7518837	6.4356988
C	8.7124949	5.2143979	5.2508492
C	8.9217908	0.3301116	8.6441300
C	2.2072167	0.5440468	5.7790608
C	1.7047112	1.1113477	7.1016705
C	2.2522505	0.7087261	9.5446893
C	8.5556804	3.2713777	6.6052207
C	10.2679364	3.2769453	4.8435740
S	7.2577526	4.2646522	7.2711619
N	10.1947783	0.3916538	8.7712831
C	11.1030627	1.2669128	8.3343448
S	11.3801881	2.7547394	9.1980718
N	12.0115068	0.9851506	7.4243250
C	12.6978895	3.2144289	8.0071341
C	13.1617688	1.9004363	7.3890500
C	12.0601477	-0.2633009	6.6710743

H	12.2684008	3.8880807	7.2685106
H	13.4950706	3.7232344	8.5424752
H	13.4882637	2.0349316	6.3585012
H	13.9804559	1.4497797	7.9563218
H	12.9833697	-0.7939779	6.9084281
H	12.0366628	-0.0504496	5.6022922
H	11.2106558	-0.8847784	6.9360668
H	7.0971725	6.3883594	6.1305991
H	8.5534842	6.2888481	7.1421140
H	8.0774972	5.0790839	4.3695229
H	9.5313353	5.8819162	4.9830880
H	10.5669091	2.3481023	5.3187298
H	11.1255245	3.9420445	4.7395751
H	9.8746631	3.0587173	3.8475509
H	1.9801851	1.1909029	4.9360772
H	1.8252758	-0.4563815	5.5849120
H	1.6876490	2.2049507	7.0951477
H	0.7033147	0.7527400	7.3364436
H	2.2048754	1.7454032	9.8842977
H	2.9873859	0.1673592	10.1328501
H	1.2717017	0.2500116	9.6596510
H	6.1907854	1.7665413	7.0824998
H	6.3197708	-1.4493795	9.9753324
H	8.8032346	-1.3185674	10.0510039

TGFA3

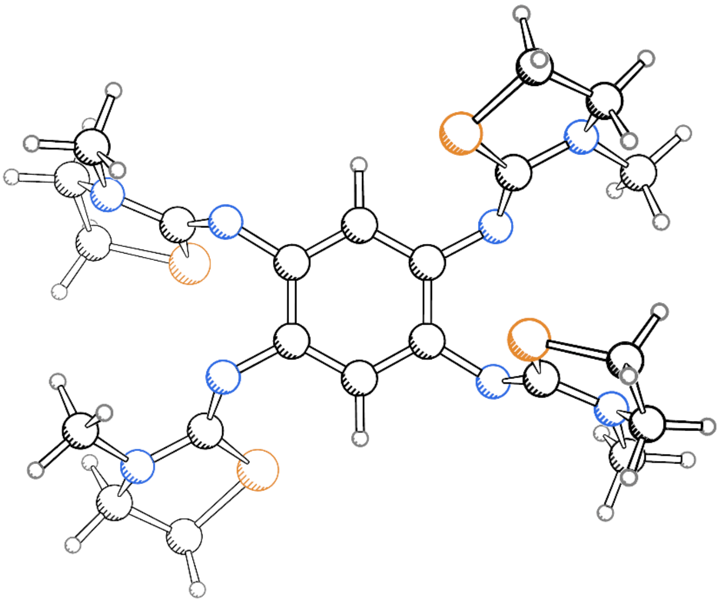

Energy = -2886.873170689

C	-0.6246195	-1.2116128	0.3538431
N	-1.2734057	-2.4207106	0.6298708
C	0.7850549	-1.1193213	0.3472150

C	-1.3770358	-0.0930665	0.0077854
C	-1.1815119	-2.9957202	1.7539363
N	1.5204638	-2.2726148	0.6285591
C	1.3770358	0.0930665	-0.0077854
C	-0.7850549	1.1193213	-0.3472150
S	-0.5259572	-2.2870966	3.2677415
N	-1.6359685	-4.2757426	1.9893294
C	2.4946596	-2.3100440	1.4348228
C	0.6246195	1.2116128	-0.3538431
N	-1.5204638	2.2726148	-0.6285591
C	-0.7942456	-3.8518500	4.1653302
C	-1.8908929	-4.5653200	3.3863998
C	-2.4536289	-4.9216984	0.9839271
S	3.1091786	-0.9795507	2.4756103
N	3.2200048	-3.4667289	1.6652318
N	1.2734057	2.4207106	-0.6298708
C	-2.4946596	2.3100440	-1.4348228
C	4.3871298	-2.0686065	3.1916116
C	3.8742762	-3.4819748	2.9602730
C	2.6604928	-4.7153772	1.1843272
C	1.1815119	2.9957202	-1.7539363
S	-3.1091786	0.9795507	-2.4756103
N	-3.2200048	3.4667289	-1.6652318
S	0.5259572	2.2870966	-3.2677415
N	1.6359685	4.2757426	-1.9893294
C	-4.3871298	2.0686065	-3.1916116
C	-3.8742762	3.4819748	-2.9602730
C	-2.6604928	4.7153772	-1.1843272
C	0.7942456	3.8518500	-4.1653302
C	1.8908929	4.5653200	-3.3863998
C	2.4536289	4.9216984	-0.9839271
H	0.1288676	-4.4325536	4.1642060
H	-1.0857949	-3.6403714	5.1917001
H	-1.8600695	-5.6445624	3.5502672
H	-2.8815221	-4.1999856	3.6958723
H	-3.4937159	-4.5721633	1.0160648
H	-2.4367535	-6.0001730	1.1461709
H	-2.0480867	-4.6895158	0.0027634
H	3.1679165	-3.7667053	3.7554485
H	4.6899442	-4.2076512	2.9536098
H	2.3279041	-4.5847199	0.1584312
H	3.4276508	-5.4888625	1.2290727
H	1.7936337	-5.0297045	1.7809986
H	4.5081961	-1.8418452	4.2486945
H	5.3323575	-1.9022836	2.6754926
H	-2.4547458	-0.1733992	0.0394792
H	2.4547458	0.1733992	-0.0394792
H	-0.1288676	4.4325536	-4.1642060
H	1.0857949	3.6403714	-5.1917001
H	1.8600695	5.6445624	-3.5502672
H	2.8815221	4.1999856	-3.6958723

H	3.4937159	4.5721633	-1.0160648
H	2.4367535	6.0001730	-1.1461709
H	2.0480867	4.6895158	-0.0027634
H	-4.5081961	1.8418452	-4.2486945
H	-5.3323575	1.9022836	-2.6754926
H	-3.1679165	3.7667053	-3.7554485
H	-4.6899442	4.2076512	-2.9536098
H	-2.3279041	4.5847199	-0.1584312
H	-3.4276508	5.4888625	-1.2290727
H	-1.7936337	5.0297045	-1.7809986

$(TGFA3)^{2+}$

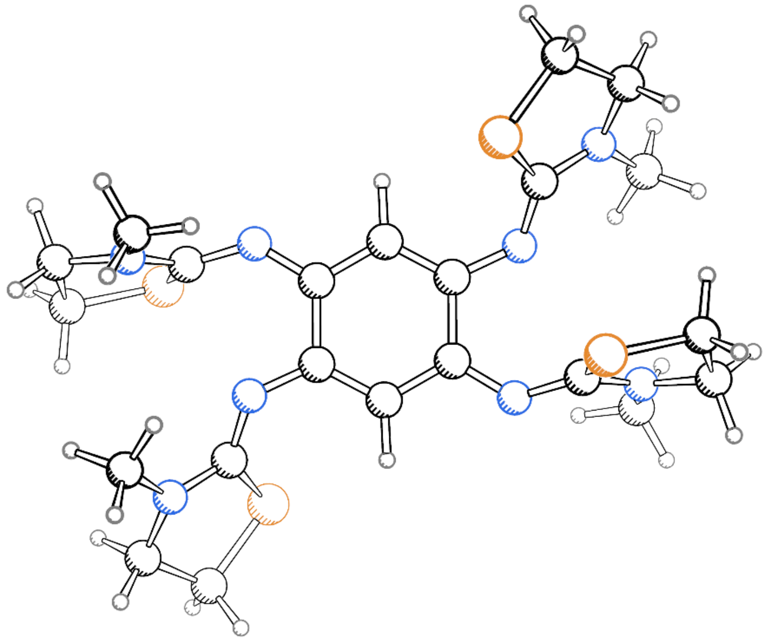

Energy = -2886.392341129

C	-0.8280905	-0.9659436	0.6253483
N	-1.5929398	-1.8300368	1.2213382
C	0.6684309	-1.0920779	0.6730114
C	-1.4199517	0.1105965	-0.0802967
C	-1.3322598	-2.7174046	2.1645775
N	1.1206291	-2.2288239	1.2261446
C	1.4199517	-0.1105965	0.0802967
C	-0.6684309	1.0920779	-0.6730114
S	-0.9559971	-2.2251481	3.8058216
N	-1.5622380	-4.0139713	2.0450504
C	2.2475990	-2.3783572	1.8681128
C	0.8280905	0.9659436	-0.6253483
N	-1.1206291	2.2288239	-1.2261446
C	-0.7582269	-3.9748372	4.3048789
C	-1.5834731	-4.7708513	3.2999543
C	-1.9927614	-4.6478459	0.8075528
S	3.1186193	-1.1033878	2.7223783

N	2.8076234	-3.5803683	2.0520643
N	1.5929398	1.8300368	-1.2213382
C	-2.2475990	2.3783572	-1.8681128
C	4.4803613	-2.2525937	3.1388437
C	3.8666083	-3.6446428	3.0575009
C	2.2367358	-4.8113518	1.5345441
C	1.3322598	2.7174046	-2.1645775
S	-3.1186193	1.1033878	-2.7223783
N	-2.8076234	3.5803683	-2.0520643
S	0.9559971	2.2251481	-3.8058216
N	1.5622380	4.0139713	-2.0450504
C	-4.4803613	2.2525937	-3.1388437
C	-3.8666083	3.6446428	-3.0575009
C	-2.2367358	4.8113518	-1.5345441
C	0.7582269	3.9748372	-4.3048789
C	1.5834731	4.7708513	-3.2999543
C	1.9927614	4.6478459	-0.8075528
H	0.2993052	-4.2299887	4.2575829
H	-1.1146298	-4.0998471	5.3237577
H	-1.1636589	-5.7627508	3.1344786
H	-2.6204815	-4.8853767	3.6283391
H	-2.9804831	-5.0910548	0.9458223
H	-1.2885764	-5.4321129	0.5277604
H	-2.0393548	-3.9024894	0.0196420
H	3.4379429	-3.9488985	4.0186521
H	4.6061492	-4.3886993	2.7628174
H	1.4695413	-4.5659632	0.8072872
H	3.0154937	-5.4074689	1.0590623
H	1.7986565	-5.3982827	2.3475940
H	4.8513391	-2.0208733	4.1334929
H	5.2796523	-2.1158521	2.4130759
H	-2.4975372	0.1763196	-0.0631013
H	2.4975372	-0.1763196	0.0631013
H	-0.2993052	4.2299887	-4.2575829
H	1.1146298	4.0998471	-5.3237577
H	1.1636589	5.7627508	-3.1344786
H	2.6204815	4.8853767	-3.6283391
H	2.9804831	5.0910548	-0.9458223
H	1.2885764	5.4321129	-0.5277604
H	2.0393548	3.9024894	-0.0196420
H	-4.8513391	2.0208733	-4.1334929
H	-5.2796523	2.1158521	-2.4130759
H	-3.4379429	3.9488985	-4.0186521
H	-4.6061492	4.3886993	-2.7628174
H	-1.4695413	4.5659632	-0.8072872
H	-3.0154937	5.4074689	-1.0590623
H	-1.7986565	5.3982827	-2.3475940

(TGFA3+H)$^{+}$

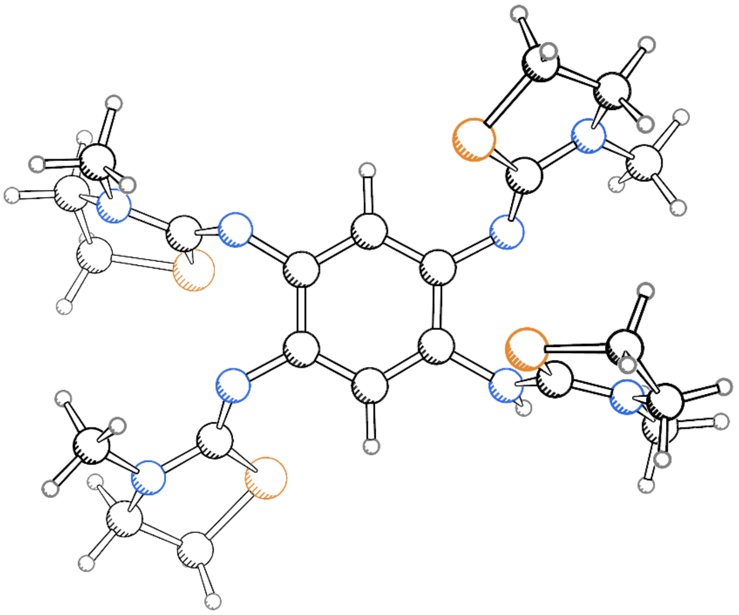

Energy = -2887.296692096

C	7.4784801	5.0972924	5.8939746
N	6.7966906	3.8803095	6.2351802
C	8.8819817	5.1051109	5.9296610
C	6.7446396	6.2157356	5.5268862
C	6.8919484	3.3382164	7.4317994
N	9.5453322	3.9179014	6.2341908
C	9.5170415	6.2961902	5.5779538
C	7.3822746	7.3992374	5.1619507
S	7.3626979	4.2892066	8.8073964
N	6.6004405	2.0690978	7.7102904
C	10.5380498	3.8580067	7.0374422
C	8.8060736	7.4346467	5.1956524
N	6.7035810	8.5700010	4.8622821
C	7.3995257	2.7763799	9.8414617
C	6.4760630	1.7869128	9.1438369
C	6.1252558	1.1247724	6.7113919
S	11.1168440	5.1433800	8.1372747
N	11.2758929	2.7186620	7.2038357
N	9.4947803	8.6031280	4.9374897
C	5.7402896	8.6439540	4.0348675
C	12.4998781	4.0949968	8.7063216
C	12.0493781	2.6679103	8.4322863
C	10.8415682	1.4812684	6.5910868
C	9.4387886	9.2233586	3.8252932
S	5.1308685	7.3428266	2.9598737
N	5.0463771	9.8053884	3.8156726
S	8.8454672	8.5563537	2.2770448
N	9.9065539	10.4928396	3.6510436
C	3.8315518	8.4387415	2.2926525

C	4.3443182	9.8491548	2.5443400
C	5.5693743	11.0462554	4.3573081
C	9.0923773	10.1605045	1.4436911
C	10.1604052	10.8644904	2.2701944
C	10.6468328	11.1465409	4.7124956
H	8.4267564	2.4163026	9.8802347
H	7.0621670	3.0237023	10.8442610
H	6.7707750	0.7557740	9.3356917
H	5.4367694	1.9193468	9.4600103
H	5.0749135	1.2949071	6.4583904
H	6.2296836	0.1173942	7.1071044
H	6.7372362	1.1994082	5.8121509
H	11.4380734	2.2877730	9.2640839
H	12.9005609	1.9980071	8.3049341
H	10.4769258	1.6889442	5.5889365
H	11.6834994	0.7922621	6.5384305
H	10.0327120	1.0067653	7.1636264
H	12.6825117	4.2785901	9.7621195
H	13.3919527	4.3475287	8.1352531
H	5.6643096	6.1742113	5.5483895
H	10.5960690	6.3483992	5.5717301
H	8.1547802	10.7162201	1.4511059
H	9.4056412	9.9931412	0.4162144
H	10.0951655	11.9483099	2.1632716
H	11.1644302	10.5483557	1.9568679
H	11.7029503	10.8531308	4.7078859
H	10.5783993	12.2266779	4.5844934
H	10.2142913	10.8623938	5.6678100
H	3.6926804	8.2317304	1.2343446
H	2.8996146	8.2529697	2.8246019
H	5.0201061	10.1633873	1.7363216
H	3.5239161	10.5658697	2.6003736
H	5.9048811	10.8774244	5.3764185
H	4.7809373	11.7979138	4.3488095
H	6.4230392	11.4123339	3.7735024
H	6.4294873	3.3154238	5.4818129

(TGFA3+2H)$^{2+}$

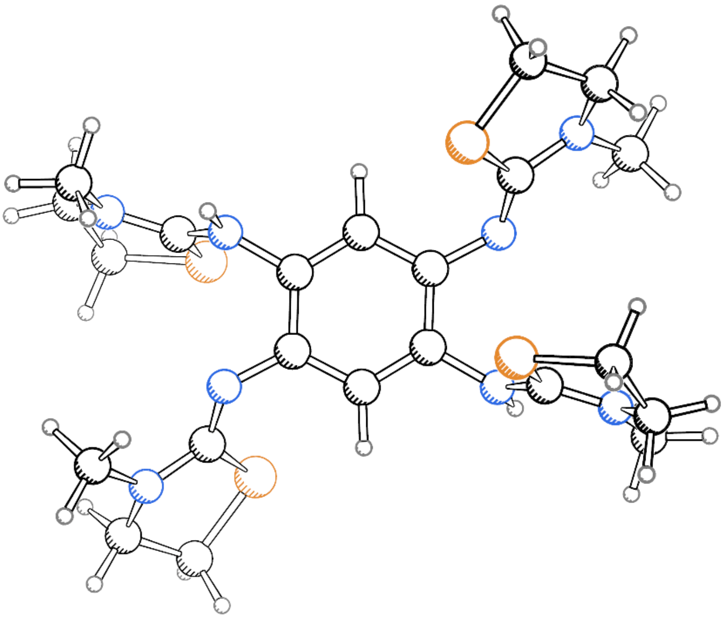

Energy = -2887.633346659

C	-0.6686489	-1.1512603	0.3061326
N	-1.3818447	-2.3595930	0.6078528
C	0.7370696	-1.1644723	0.4106385
C	-1.3860508	-0.0345864	-0.0857973
C	-1.4013996	-2.8976454	1.8180200
N	1.3787405	-2.3471844	0.7143552
C	1.3860508	0.0345864	0.0857973
C	-0.7370696	1.1644723	-0.4106385
S	-0.9875569	-1.9650117	3.2220837
N	-1.7855997	-4.1406360	2.0715875
C	2.3754692	-2.4284868	1.5260210
C	0.6686489	1.1512603	-0.3061326
N	-1.3787405	2.3471844	-0.7143552
C	-1.1021947	-3.4714205	4.2621626
C	-2.0173382	-4.4181121	3.4969044
C	-2.2476998	-5.0644562	1.0424589
S	2.8847586	-1.1918307	2.7092618
N	3.1432695	-3.5369854	1.6315059
N	1.3818447	2.3595930	-0.6078528
C	-2.3754692	2.4284868	-1.5260210
C	4.3434007	-2.1845653	3.1866713
C	3.9736223	-3.6166407	2.8256172
C	2.8341076	-4.7448768	0.8893445
C	1.4013996	2.8976454	-1.8180200
S	-2.8847586	1.1918307	-2.7092618
N	-3.1432695	3.5369854	-1.6315059
S	0.9875569	1.9650117	-3.2220837
N	1.7855997	4.1406360	-2.0715875
C	-4.3434007	2.1845653	-3.1866713

C	-3.9736223	3.6166407	-2.8256172
C	-2.8341076	4.7448768	-0.8893445
C	1.1021947	3.4714205	-4.2621626
C	2.0173382	4.4181121	-3.4969044
C	2.2476998	5.0644562	-1.0424589
H	-0.0993138	-3.8769399	4.3831893
H	-1.5053603	-3.1978665	5.2333162
H	-1.7801225	-5.4602800	3.7049047
H	-3.0697928	-4.2425149	3.7363015
H	-3.2643061	-4.8273340	0.7182451
H	-2.2379577	-6.0718225	1.4503361
H	-1.5694397	-5.0404556	0.1893039
H	3.4232692	-4.0981798	3.6436001
H	4.8610046	-4.2138494	2.6160548
H	2.4016471	-4.4723610	-0.0689188
H	3.7500959	-5.3113298	0.7287723
H	2.1217242	-5.3757640	1.4331231
H	4.5313430	-2.0581579	4.2495579
H	5.2058138	-1.8361535	2.6214008
H	-2.4652989	-0.0807167	-0.1096037
H	2.4652989	0.0807167	0.1096037
H	0.0993138	3.8769399	-4.3831893
H	1.5053603	3.1978665	-5.2333162
H	1.7801225	5.4602800	-3.7049047
H	3.0697928	4.2425149	-3.7363015
H	3.2643061	4.8273340	-0.7182451
H	2.2379577	6.0718225	-1.4503361
H	1.5694397	5.0404556	-0.1893039
H	-4.5313430	2.0581579	-4.2495579
H	-5.2058138	1.8361535	-2.6214008
H	-3.4232692	4.0981798	-3.6436001
H	-4.8610046	4.2138494	-2.6160548
H	-2.4016471	4.4723610	0.0689188
H	-3.7500959	5.3113298	-0.7287723
H	-2.1217242	5.3757640	-1.4331231
H	-1.7138653	-2.9142220	-0.1684713
H	1.7138653	2.9142220	0.1684713

p-$C_6H_4O_2$

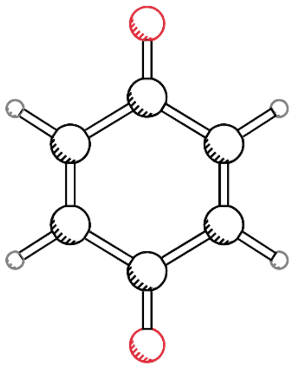

Energy = -381.4185597195

C	1.4382240	0.0000000	0.0000000
C	0.6678719	0.0000000	1.2653161
C	-0.6678719	0.0000000	1.2653161
C	-1.4382240	0.0000000	0.0000000
C	-0.6678719	0.0000000	-1.2653161
C	0.6678719	0.0000000	-1.2653161
O	2.6559836	0.0000000	0.0000000
H	1.2537760	0.0000000	2.1761039
H	-1.2537760	0.0000000	2.1761039
O	-2.6559836	0.0000000	0.0000000
H	-1.2537760	0.0000000	-2.1761039
H	1.2537760	0.0000000	-2.1761039

p-$C_6H_4(OH)_2$

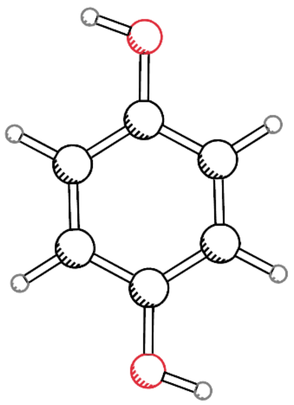

Energy = -382.6539095857

C	1.3948834	-0.0297794	0.0000000
C	0.7162126	1.1855683	0.0000000
C	-0.6720935	1.2168373	0.0000000
C	-1.3948834	0.0297794	0.0000000
C	-0.7162126	-1.1855683	0.0000000
C	0.6720935	-1.2168373	0.0000000

O	2.7634000	-0.1165039	0.0000000
H	1.2722099	2.1168240	0.0000000
H	-1.2042161	2.1585829	0.0000000
O	-2.7634000	0.1165039	0.0000000
H	-1.2722099	-2.1168240	0.0000000
H	1.2042161	-2.1585829	0.0000000
H	3.1410770	0.7696719	0.0000000
H	-3.1410770	-0.7696719	0.0000000

[1] U. Jahn, P. Hartmann, I. Dix, P.G. Jones, *Eur. J. Org. Chem.* **2001**, *17*, 3333–3355.

[2] N. G. Conelly, W. Geiger, *Chem. Rev.* **1996**, *96*, *2*, 877–910.

[3] U. Wild, S. Federle, A. Wagner, E. Kaifer, H.-J. Himmel, *Chem. Eur. J.* **2016**, *22*, 11971–11976.

[4] A. Peters, H. Herrmann, M. Magg, E. Kaifer, H.-J. Himmel, *Eur. J. Inorg. Chem.* **2012**, 1620–1631.

[5] H. Eilingsfeld, L. Möbius, *Chem. Ber*. **1965**, *98*, 1293–1307.

[6] SAINT (APEX III/IV) Bruker AXS GmbH, Karlsruhe, Germany **2016/2021**.

[7] a) G. M. Sheldrick, SADABS, Bruker AXS GmbH, Karlsruhe, Germany **2004-2014**; b) L. Krause, R. Herbst-Irmer, G. M. Sheldrick, D. Stalke, *J. Appl. Cryst*. **2015**, *48*, 3–10.

[8] a) G. M. Sheldrick, SHELXT, Program for Crystal Structure Solution, University of Göttingen, Germany **2014-2018**; b) G. M. Sheldrick, *Acta Cryst*. **2015**, *C71*, 3–8.

[9] a) G. M. Sheldrick, SHELXL-20xx, University of Göttingen and Bruker AXS GmbH, Karlsruhe, Germany **2012-2018**; b) W. Robinson, G. M. Sheldrick in: N. W. Isaaks, M. R. Taylor (eds.) *„Crystallographic Computing 4*“, Ch. 22, IUCr and Oxford University Press, Oxford, UK, **1988**; c) G. M. Sheldrick*, Acta Cryst.* **2008**, *A64*, 112–122.

[10] O. V. Dolomanov, L. J. Bourhis, R. J. Gildea, J. A. K. Howard, H. Puschmann, *J. Appl. Cryst.* **2009**, *42*, 339–341.

[11] A. Thorn, B. Dittrich, G. M. Sheldrick, *Acta Cryst.* **2012**, *A68*, 448–451.

[12] a) P. v. d. Sluis, A. L. Spek*, Acta Cryst.* **1990**, *A46*, 194–201; b) A. L. Spek, Acta Cryst. **2015**, *C71*, 9–18.

[13] A. L. Spek, PLATON, Utrecht University, The Netherlands; b) A. L. Spek, *J. Appl. Cryst.* **2003**, *36*, 7–13.

[14] U. Wild, F. Schön, H.-J. Himmel, *Angew. Chem.* **2017**, *129*, 16630-16633; *Angew. Chem. Int. Ed.* **2017**,*56*, 16410-16413.

[15] TURBOMOLE V7.7 2022, a development of University of Karlsruhe and Forschungszentrum Karlsruhe GmbH, 1989-2007, TURBOMOLE GmbH, since 2007; available from https://www.turbomole.org.

[16] S. G. Balasubramani, G. P. Chen, S. Coriani, M. Diedenhofen, M. S. Frank, Y. J. Franzke, F. Furche, R. Grotjahn, M. E. Harding, C. Hättig, A. Hellweg, B. Helmich-Paris, C. Holzer, U. Huniar, M. Kaupp, A. Marefat Khah, S. Karbalaei Khani, T. Müller, F. Mack, B. D. Nguyen, S. M. Parker, E. Perlt, D. Rappoport, K. Reiter, S. Roy, M. Rückert, G. Schmitz, M. Sierka, E. Tapavicza, D. P. Tew, C. van Wüllen, V. K. Voora, F. Weigend, A. Wodyński, J. M. Yu, *J. Chem. Phys.* **2020,** *152,* 184107.

[17] R. Ahlrichs, M. Bär, M. Häser, H. Horn, C. Kölmel, *Chem. Phys. Lett.* **1989,** *162,* 165–169.

[18] O. Treutler, R. Ahlrichs, *J. Chem. Phys.* **1995,** *102,* 346–354.

[19] A. D. Becke, *J. Chem. Phys.* **1993,** *98,* 5648–5652.

[20] P. J. Stephens, F. J. Devlin, C. F. Chabalowski, M. J. Frisch, *J. Phys. Chem.* **1994,** *98,* 11623–11627.
[21] F. Weigend, R. Ahlrichs, *Phys. Chem. Chem. Phys.* **2005,** *7,* 3297–3305.
[22] K. Eichkorn, O. Treutler, H. Öhm, M. Häser, R. Ahlrichs, *Chem. Phys. Lett.* **1995,** *242,* 652–660.
[23] F. Weigend, *Phys. Chem. Chem. Phys.* **2006,** *8,* 1057–1065.
[24] S. Grimme, J. Antony, S. Ehrlich, H. Krieg, *J. Chem. Phys.* **2010,** *132,* 154104.
[25] S. Grimme, S. Ehrlich, L. Goerigk, *J. Comput. Chem.* **2011,** *32,* 1456–1465.
[26] P. Deglmann, F. Furche, R. Ahlrichs, *Chem. Phys. Lett.* **2002,** *362,* 511–518.
